# Phenylpyridine-Based Boron Azides: Tuning Reactivity and Accessing Fluorescent Triazoles

**DOI:** 10.1021/acs.inorgchem.5c03363

**Published:** 2025-09-05

**Authors:** Karel Škoch, Michaela Buziková, Drahomír Hnyk, Miroslava Litecká, Anna Vykydalová, Dmytro Bavol, Kamil Lang, Kaplan Kirakci

**Affiliations:** † 112895 Institute of Inorganic Chemistry of Czech Academy of Sciences, Husinec-Řež 1001, 250 68, Czech Republic; b Polymer Institute, Slovak Academy of Sciences, Dúbravská cesta 9, 845 41 Bratislava, Slovakia

## Abstract

We report the synthesis and reactivity of phenylpyridine-based boron azides readily accessible via nucleophilic substitution from *in situ* generated borenium-type precursors. Three azides were obtained: a hydridic species (L^NC^)­BHN_3_ (L^NC^ = 2-phenylpyridine), a cyclopentyl-substituted analogue (L^NC^)­B­(cyclopentyl)­N_3_, and a boron diazide (L^NC^)­B­(N_3_)_2_ obtained as a byproduct from the synthesis of (L^NC^)­BHN_3_. The prepared borane azides exhibit notable thermal and photochemical robustness, with decomposition temperatures around 140 °C in mesitylene solution and above 170 °C in the solid state, as evidenced by DSC/TGA analysis. Reactivity studies revealed weak electrophilic character compared to organic azides, evidenced by their reluctance to undergo Staudinger-type reactions and relatively high activation barriers in [3 + 2] cycloadditions, even with activated or strained alkynes. In contrast, reactions with nucleophiles afforded unusual head-to-head azide-bridged species. Attempts to reduce the azide functionality to primary amines generally led to elimination and formation of borinic acid. Computational analysis supported the experimental findings and provided insight into frontier orbital interactions and energy profiles during the [3 + 2] cycloadditions. Selected boron-triazole products displayed UV-A/deep-blue fluorescence, with emission maxima at 372–385 nm in solution and 397–438 nm in the solid state, and high quantum yields up to 0.74 (in dichloromethane solution) and 0.48 (in solid state), suggesting their potential as UV-A or deep-blue-emitting fluorophores.

## Graphical abstract

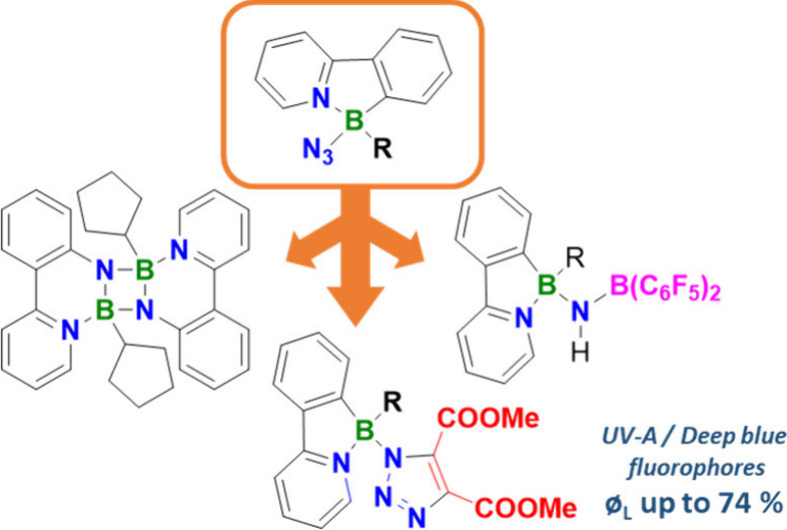

## Introduction

Organic azides represent an important class of compounds, which continuously inspires chemists to explore analogous structures involving other main-group elements.^1^ Since the synthesis of a simple boron triazide, B­(N_3_)_3_, in 1954,^2^ a wide array of boron azide derivatives has been described. In many regards, their reactivity mirrors that of their carbon counterparts. For example, due to the dipolar nature of the azide group, these compounds are commonly employed in various 3 + 2 Huisgen-type cycloadditions, enabling the attachment of boron moieties into larger molecular assemblies.^3^ A Staudinger-type reaction of borane azides has been reported, leading to the formation of borane-substituted iminophosphoranes,^4^ some of which were reported to exhibit blue emission with good fluorescence quantum yields.^5^ Similarly to organic azides, they can undergo thermo- or photolysis to generate elusive nitrene-like species, which readily engage in further transformations.^6^ In the absence of other reactive groups, these processes often lead to the formation of aminoboranes (R_2_B = NR_2_) or cyclic borazines, (RB–NR’)_*n*_.^7^ Distinct reactivity is the ability of insertion of the azide into a C–B bond, resulting in the formation of triazene-like compounds,^8^ 1,2 azaborinine^9^ or other BN-doped (poly)­aromatics,^10^ which are highly valued for their photophysical properties.^11^

We have recently reported the synthesis of highly luminescent borenium-type Lewis (super)­acids derived from arylpyridines,^12^ a class of compounds that combines synthetic accessibility, structural diversity, and intriguing photophysical properties resulting from the participation of boron in the extended π-system. While borenium-type Lewis superacids are typically stabilized by N-heterocyclic carbene (NHC) donors,^13^ arylpyridine offer a versatile alternative scaffold. Moreover, a variety of dialkyl or diaryl arylpyridine boranes have already been proven to have luminiscent properties.^14^ Motivated by the availability of arylpyridine boranes, we set out to explore nucleophilic substitution at the boron center to access a new class of boron azides and investigate their reactivity and potential photophysical behavior. CAUTION: Many azide compounds are toxic and potentially unstable or explosive. Although none of these hazardous properties were observed during our study, we still recommend handling them with appropriate caution.

## Results and Discussion

As an entry point for our investigations, phenylpyridine borane **1** was employed. It can be obtained in two steps by electrophilic borylation of phenylpyridine.^15,16^ Borane **1** was then treated with triflimic acid (a strong Brønsted acid), leading to hydrogen elimination and formation of reactive borenium-type compound **2a** featuring a weakly coordinating triflimide group. This species is prone to nucleophilic substitution as previously reported by Lacôte and Curran for nucleophilic substitutions of NHC-based boron compounds^17^ (see Scheme 1). The *in situ* generated arylborane **2a** was thus reacted with NaN_3_ in tetrahydrofuran to provide boron azide **3a** in good yield (up to 79% isolated yield). A small amount (<10%) of diazide **3b** was also isolated from this reaction mixture presumably as a result of anion scrambling in the reaction process. During the investigation of the reactivity of **3a**, it became evident that the reactive B–H group often interferes with the azide reactivity and can lead to various side reactions or mixture formation. To address this issue, we prepared a cyclopentyl-substituted azide **3c** in an analogous manner as it can be prepared from **1** by incorporating hydroboration of cyclopentene as an additional reaction step into the one pot reaction sequence (for reaction details see SI). Cyclopentyl substituted boron azide was, in this case, isolated as a sole product in 75% yield. All three boron azide compounds **3a**–**c** were suitable for column chromatography purification and were characterized by NMR, IR, elemental analysis and SC-XRD (see SI and Figure 1). The ^11^B NMR spectra of the compounds show chemical shifts of δ_B_ = −0.4 for **3a** (doublet with ^1^ *J*_BH_ ≈ 110 Hz), 3.5 for **3b**, and 4.6 for **3c**, which are slightly deshielded but still fall within the typical range for neutral tetracoordinated boron complexes. Notably, the azide group vibrations were observed within a narrow range of 2094–2096 cm^–1^, which is higher than for organic (aliphatic) azides (i.e., benzyl azide υ_N_3__ = 2088 cm^–1^),^18^ but comparable to values observed for carbene stabilized borane azides (IPrBH_2_N_3_: υ_N_3__ = 2096 cm^–1^, IPr = 1,3-bis­(2,6-diisopropylphenyl)­imidazol-2-ylidene).^19^ Crystal structures of **3a**–**c** and selected bond lengths and angles are depicted in Figure 1 and Table 1.

**Scheme 1:**
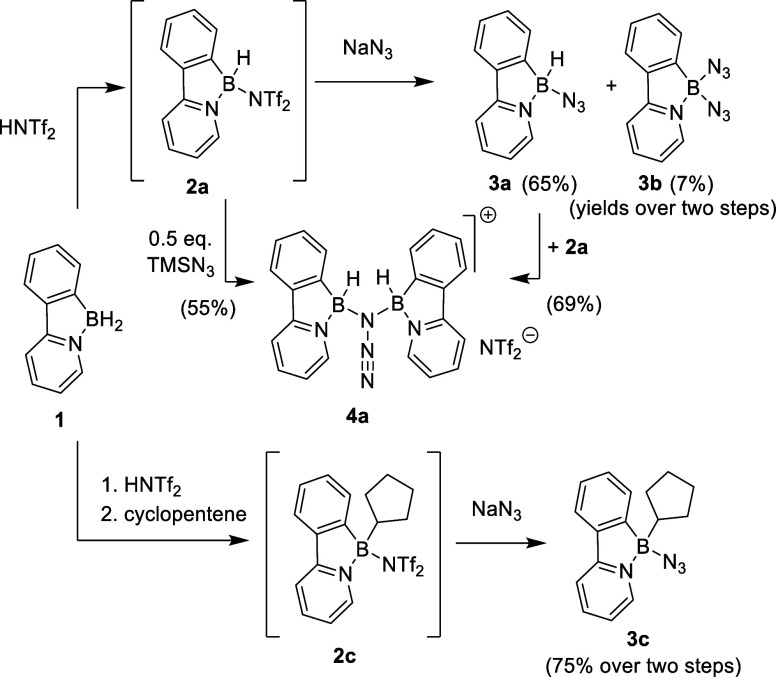
Preparation of Boron Azides

**Figure 1:**
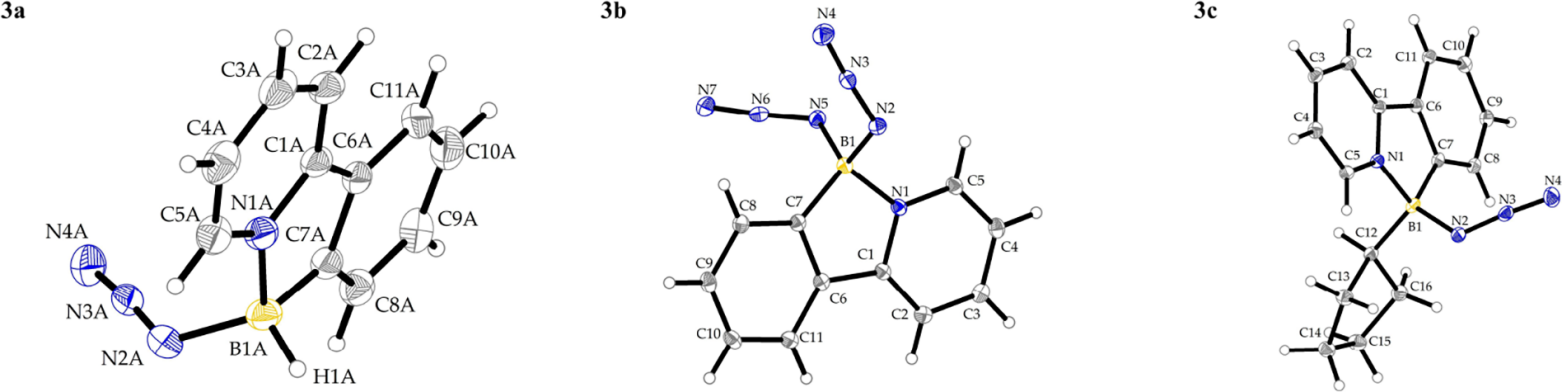
View of the molecular structures of boron azides **3a**–**c** with thermal ellipsoids shown at the 30% probability level. For compound **3a**, only one of the two independent molecules is shown.

**Table 1:** Geometrical Parameters of Selected Bonds Lengths [Å] and Angles [°] in Boron Azides **3a**–**c**

	**Bond length [Å]/** angle [°]				
Bond	**3a** ^a^		**3b** ^b^		**3c**
B1–N1	1.605(4)	1.609(5)	1.585(2)		1.625(2)
B1–C7	1.608(5)	1.593(5)	1.600(2)		1.609(2)
B1–N2	1.550(4)	1.535(5)	1.544(2)	1.554(2)	1.566(2)
N2–N3	1.216(4)	1.211(4)	1.224(2)	1.222(2)	1.216(2)
N3–N4	1.128(4)	1.141(5)	1.139(2)	1.138(2)	1.142(2)
N1–B1–C7	97.9(2)	98.3(3)	98.8(1)		97.3(1)
B1–N2–N3	119.2(3)	120.2(3)	116.0(1)	119.9(1)	119.7(1)
N2–N3–N4	175.2(3)	174.6(3)	175.6(1)	174.9(1)	175.3(2)

a For compound **3a**, there are two independent molecules in the crystal structure.

b For compound **3b**, values refer to both chemically equivalent azide fragments.

The synthesized compounds **3a–c** exhibit a high photochemical and thermal stability. Neither prolonged irradiation with a mercury UV lamp (6 h) nor heating in toluene at 100 °C for 1 week resulted in detectable decomposition. In additional thermal stability tests in mesitylene, compounds **3a** and **3b** underwent nonselective decomposition only after heating at 140 °C for 20 h, whereas compound **3c** remained almost intact even after 5 days of heating at 160 °C, indicating enhanced thermal robustness due to the presence of cyclopentyl moiety. To further investigate their thermal stability, simultaneous thermogravimetric analysis and differential scanning calorimetry (TGA/DSC) analyses were performed. These studies revealed that thermolysis, accompanied by nitrogen elimination, occurs for solid samples only at elevated temperatures above 170 °C. This decomposition appears to proceed in a nonselective manner, as no defined products could be isolated from the thermolyzed mixtures (for details on thermolytic and photolytic experiments, see SI).

The reaction of phenylpyridylborane **2a** with one equivalent of trimethylsilyl azide (TMSN_3_) led to the formation of compound **4** in which azide unit interconnects two phenylpyrineborane fragments as a head-to-head bridge. Reaction byproduct (TMSNTf_2_) was removed by washing with hexane and product **4a** was obtained as a mixture of *rac*- and *meso*-isomers (approximate ratio was determined to be 2:1 by ^1^H NMR). In this case, the infrared vibration of the azide group is shifted to higher wavenumbers (2184 cm^–1^), representing a significantly greater increase compared to other structurally related motifs featuring bridging azides (e.g., for IPr-BH_2_N_3_–B­(Ar^F^)_3_: υ_N_3__ = 2134 cm^–1^)^19^ and comparable for cationic bis-silyl azide (Me_3_Si)_2_N_3_^+^ B­(C_6_F_5_)_4_^–^ (υ_N_3__= 2189 and 2175 cm^–1^).^20^ The identity of **4a** was confirmed by single crystal X-ray diffraction analysis (SC-XRD, Figure 2). We analyzed the bond lengths and angles, observing that, compared to the parent azide **3a**, the length of nitrogen–nitrogen bond proximal to boron (B) N–N was significantly elongated (from 1.216(4) and 1.211(4) Å in **3a** to 1.272(7) Å in **4a**), while the terminal N≡N bond was shortened (from 1.128(4) and 1.141(5) Å in **3a** to 1.110(8) Å in **4a**) suggesting a stronger polarization of the activated azide moiety.

**Figure 2:**
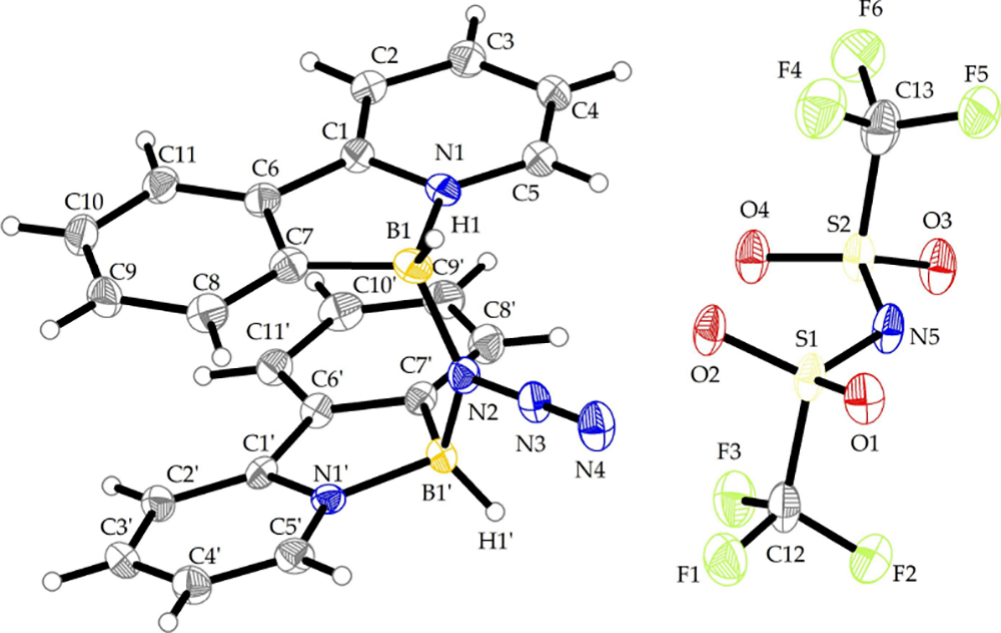
View of the molecular structure of compound **4a** with thermal ellipsoids shown at the 30% probability level. Selected bond lengths (Å) and angles (°): B1–N1 1.601(9), B1–C7 1.609 (10), B1–N2 1.595(9), N2–N3 1.272(7), N3–N4 1.110(8), N1–B1–C7 98.1(5), B1–N2–B1́ 131.1(5), B1–N2–N3 113.4(5), N2–N3–N4 179.5(7), ΣN2_BBN_ = 360°.

The cationic part of **4a** [(L^NC^)­BH]_2_N_3_^+^ structurally resembles the hydride-bridged cationic species [(L^NC^)­BH]_2_H^+^, previously observed upon hydride abstraction from **1** using sub stoichiometric amounts of trityl borate.^12^ The geometry of azide bridged compound **4a** suggests more effective π–π stacking interactions between the arylpyridine units, as indicated by the narrower angle between azaborole rings (13° in **4a**, compared to 20° in [(L^NC^)­BH]_2_H^+^), and shorter centroid-to-centroid distances between the corresponding aromatic rings (3.698 and 3.643 Å in **4a** vs significantly larger values 4.140 and 4.297 Å in the more twisted molecular assembly of [(L^NC^)­BH]_2_H^+^).

Although the Staudinger-type reaction of boron azides has been reported several times,^4^ it proceeds poorly with our compounds. Reactions with different phosphorus nucleophiles (P­(OEt)_3_, PPh_3_ or *n*-Bu_3_P), even under harsh conditions (*d*_8_-toluene 100 °C or 140 °C in mesitylene) occur very slowly (Scheme 2). Even after 2 weeks of heating, direct monitoring by ^1^H, ^11^B and ^31^P­{^1^H} NMR showed incomplete conversions to the corresponding iminophosphoranes **5.** These compounds were not isolated due to their reactive nature, but their formation was supported by NMR and high resolution mass spectrometry (HRMS, for details see SI). In the reaction of **3c** with tributylphosphine, several crystals of the dimeric dibenzo-1,3,2-diazaborinine **6c** were isolated from the reaction mixture as an unexpected product of nitrene insertion to the B–C bond. Even though thermally initiated nitrene insertions into B–C or B–B bonds are known,^7,21^ we did not observe any signs of this reactivity under analogous conditions neither in the absence, nor in the presence of a catalytic amount (20 mol %) of tri-*n*-butyl phosphine. We are currently unable to explain the role of phosphine in this reaction. Compound **6c** crystallizes as a centrosymmetric dimer featuring a nearly perfect square N_2_B_2_ motif in which the cyclopentyl fragments lie above and below the molecular axis. Due to the tetracoordinate environment of the boron atom, the nitrogen originated from the azide exhibits pronounced pyramidalization and the whole aryl-pyridine unit is distorted. The aromatic rings in the phenylpyridine ligands are significantly bent, enclosing an angle of 31.1°. For the depiction of molecular structure, see Figure 3.

**Scheme 2:**
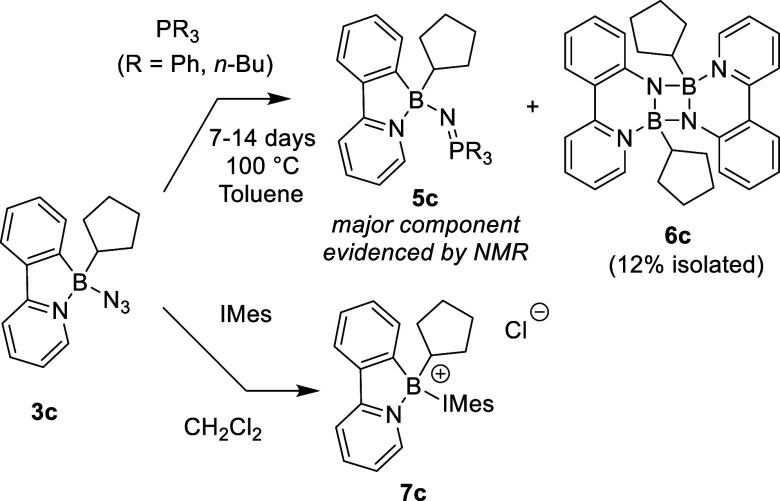
Reaction of Azide **3c** Toward Nucleophiles

**Figure 3:**
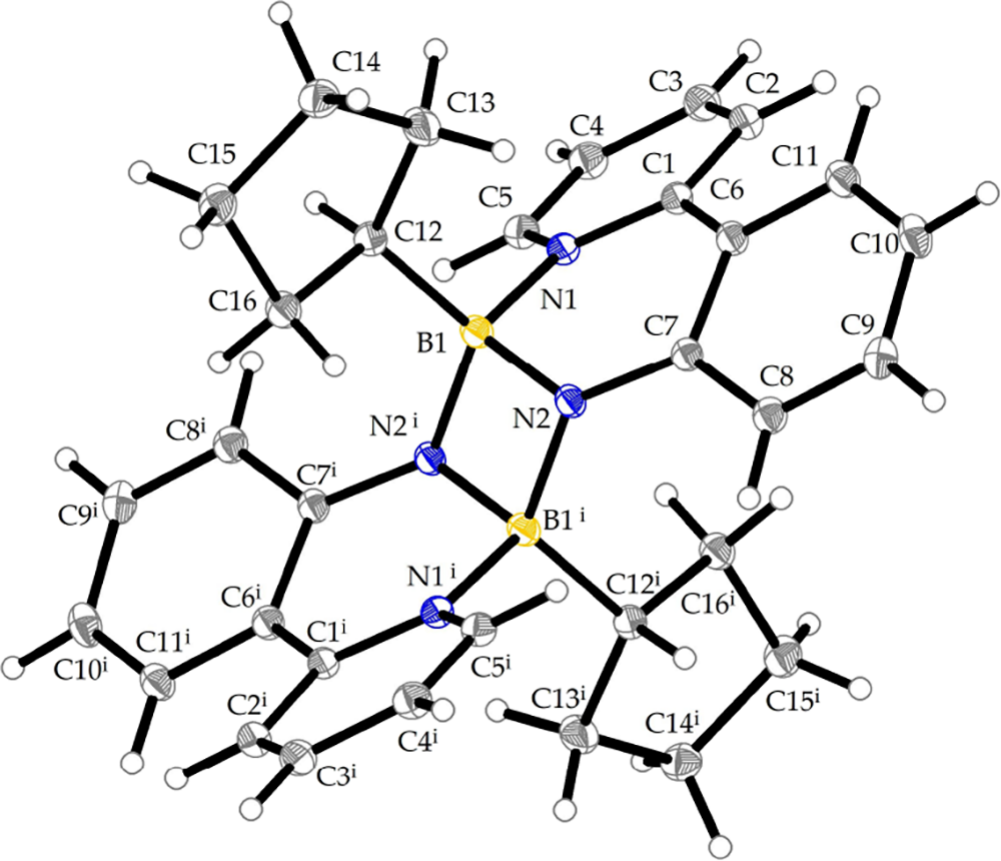
View of the molecular structure of the dimeric dibenzo-1,3,2-diazaborinine **6c** with thermal ellipsoids shown at the 30% probability level. Selected bond lengths (Å) and angles (°): B1–N1 1.618(2), B1–N2 1.532(2), B1–N2́ 1.544(2), N1–B1–N2 106.1(1), B1–N2–C7 123.4(1), N2–B1–N2́ 90.71(9), B1–N2–B1́ 89.29(9), ΣN2_BBC_ = 346.6°.

The reaction of azides with the carbon nucleophile, 1,3-dimesitylimidazol-2-ylidene (IMes carbene), typically leads to the formation of triazenes.^22^ In our case, this resulted in the rapid elimination of the azide anion and the formation of an adduct between the Lewis acidic fragment and the IMes carbene **7c**. As proved by SC-XRD under crystallization conditions, exchange of the counterion to chloride occurred, with the chloride presumably originating from dichloromethane as the solvent (for more details and crystal structure of **7c** see SI). Attempted reaction in *d*_*6*_-benzene or *d*_*8*_-toluene resulted in immediate darkening of the reaction mixture and the formation of complex mixture of products (for *in situ* experiments see SI).

Reduction of azides is a well-established method for preparation of primary amines, and we explored this approach as a potential route to obtain an amine-substituted borane (see Scheme 3). In our case, no reaction occurred upon treatment of **3a** or **3c** using either NaBH_4_ or hydrogen gas over palladium on carbon. Conversely, complete elimination of the azide and its replacement by a hydride group were observed when using the more reactive reducing agent, LiAlH_4_. Metal-assisted reduction with the NiCl_2_/NaBH_4_ system^23^ under optimized conditions (5 eq. NaBH_4_, 0.1 eq. NiCl_2_) resulted in the formation of borinic acid **8c** in 76% yield, presumably due to a rapid hydrolysis of the generated amine. Compound **8c** is sufficiently stable to be purified on silica gel, and it was obtained as colorless oil, which solidified over time. Results from HRMS were found inconclusive, as signals referring to both the borinic acid B–OH and its B–O–B anhydride were present. However, ^1^H NMR measurement showed a broad signal at δ_H_ 4.11 and IR revealed a broad vibration in IR υ_OH_ 3392 cm^–1^, both corroborating the presence of the −OH functionality and confirming the product identity as the monomer. As further evidenced by SC-XRD, in the solid state, compound **8c** adopts a tetrameric supramolecular arrangement supported by formation of hydrogen bonds (see Figure 4 and Table 2). Attempted reduction of **3a** under the same conditions suggested that the hydride moiety is incompatible with the reaction conditions and the corresponding borinic acid was not obtained.

**Scheme 3:**
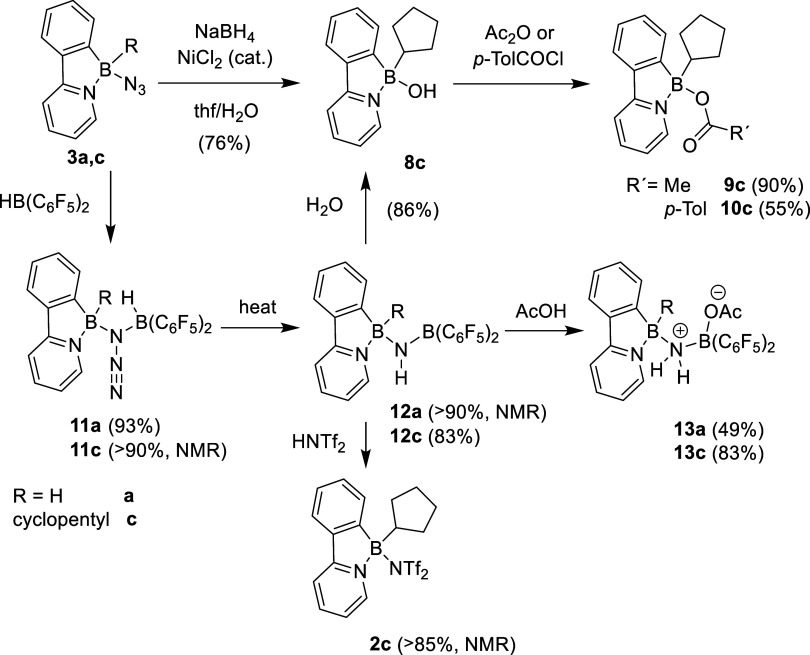
Reactivity of Azides **3a** and **3c** toward Boron Hydrides and Follow-up Transformations

**Figure 4:**
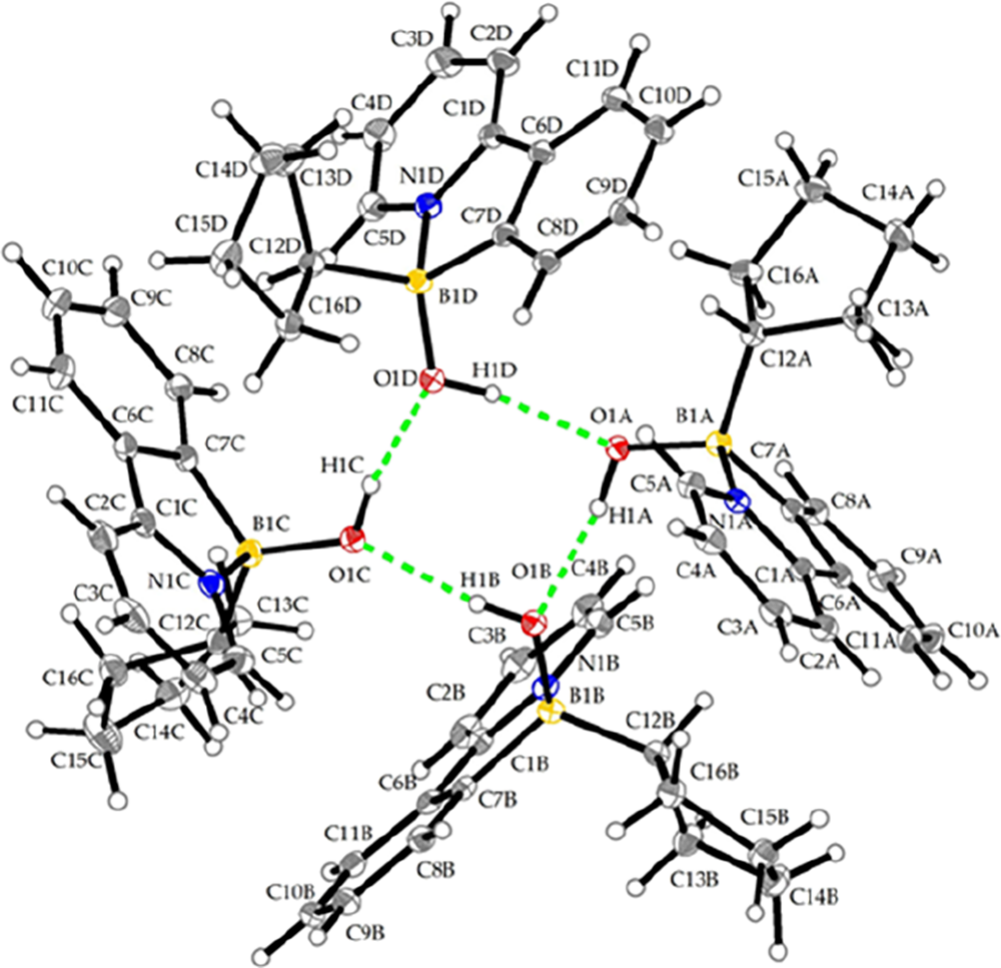
Supramolecular assembly observed in crystal structure of **8c** with thermal ellipsoids shown at the 30% probability level. Solvate molecule (hexane) is omitted for clarity.

**Table 2:** Geometrical Parameters of the Hydrogen Bonds in **8c** [Å, °]

D–H···A	d(D–H)	d(H···A)	d(D···A)	∠(DHA)
O1A–H1A···O1B	0.91(2)	1.86(2)	2.756(2)	167(2)
O1B–H1B···O1C	0.84(3)	1.96(3)	2.797(2)	172(2)
O1C–H1C···O1D	0.88(3)	1.91(3)	2.771(2)	168(2)
O1D–H1D···O1A	0.86(2)	1.95(2)	2.802(2)	169(2)

The reactivity of borinic acid **8c** was briefly investigated in a reaction with acetic anhydride or 4-methylbenzoyl chloride, giving the corresponding mixed-anhydrides in 90% yield (for acetyl derivative **9c**) and 55% yield (for 4-methylbenzoyl derivative **10c**) as bench stable solids (for experimental details and crystal structure of **9c** see SI).

As an alternative method for the azide reduction, we investigated the use of Piers’ borane, HB­(C_6_F_5_)_2_, as a strongly electrophilic hydride source.^24^ Upon mixing **3a** with with HB­(C_6_F_5_)_2_ in dichloromethane, solvent removal and brief washing with hexane to solidify the product, compound **11a** was obtained in nearly quantitative yield. The NMR analysis revealed the presence of two distinct B–H motifs featuring broad quartet-like signals at δ_H_ = 3.92 and 3.37 ppm in the ^1^H NMR and two signals in ^11^B NMR (δ_B_ = 0.8 and –12.0 ppm), significantly broadened when comparing ^11^B­{^1^H} and ^11^B NMR spectra. IR analysis also supported the presence of two B–H bonds by distinct B–H vibrations (υ_BH_ = 2451 and 2418 cm^–1^) accompanied by a sharp azide vibration peak exhibiting a shift to a higher wavenumber (υ_N_3__ = 2164 cm^–1^) compared to the parent boron azide. The obtained product proved to be an azide bridged adduct of HB­(C_6_F_5_)_2_ with **3a**, further demonstrating the nucleophilic nature of the boron azides. SC-XRD confirmed the molecular structure (Figure 5). Compound **11a** represents an example of a Lewis acid- boron azide adduct analogous to our compound **4a**, or IPr-BH_2_N_3_–BCF and 9-Ph-borafuorene-N_3_–AuPPh_3_ complexes previously described by Rivard^19^ and Martin.^25^

**Figure 5:**
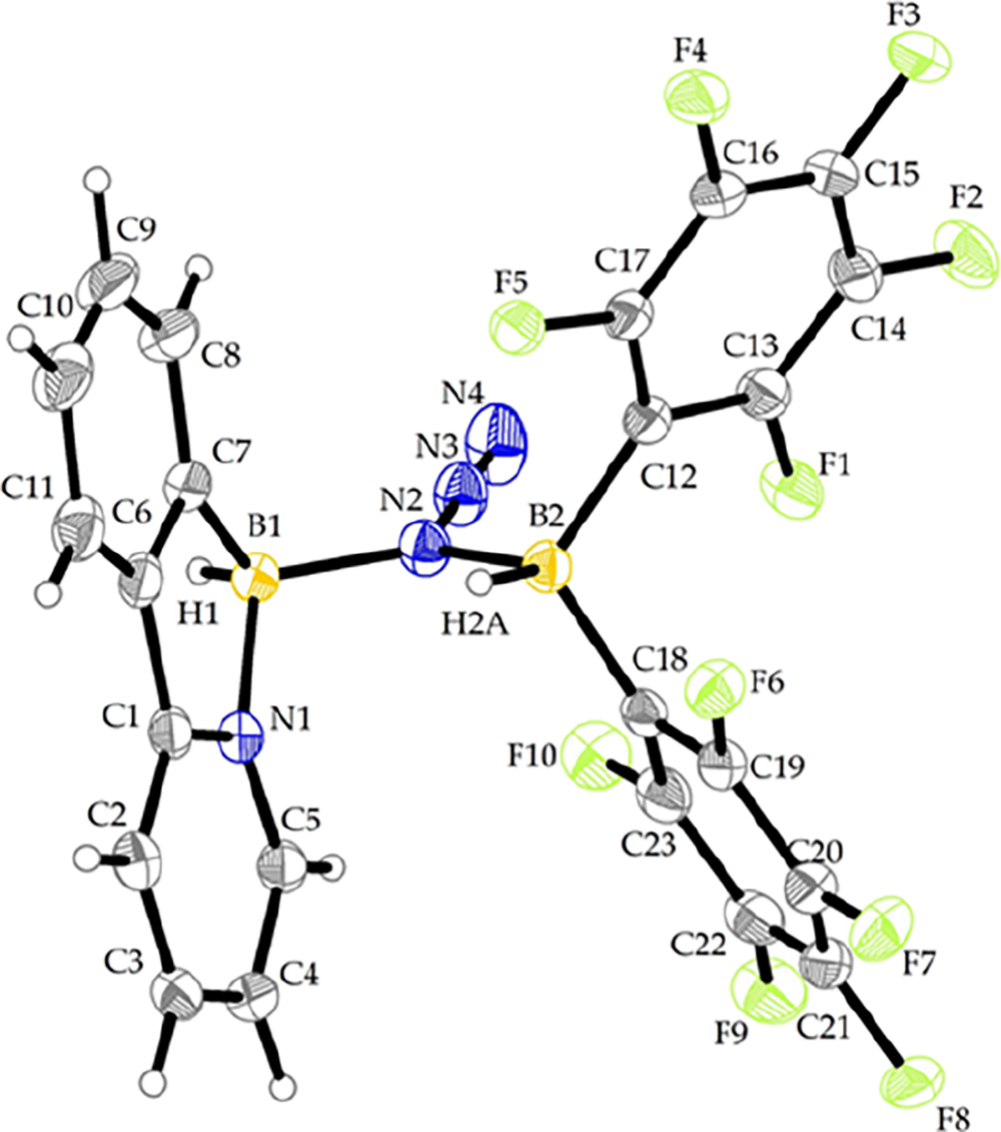
View of the molecular structure of **11a**. Selected bond lengths (Å) and angles (°): B1–N1 1.597(3), B1–N2 1.587(3), B2–N2 1.600(3), N2–N3 1.256(3), N3–N4 1.126(3), N1–B1–C7 98.6(2), B1–N2–B2 124.8(2), B1–N2–N3 116.7(2), B2–N2–N3 118.2(2), N2–N3–N4 178.1(3), ∑N2_BBN_ = 359.7°.

Compound **11a** was thermolyzed for 1 h at 80 °C in *d*_*6*_-benzene. NMR and HRMS analyses conclusively showed the elimination of a nitrogen molecule and a hydride shift, giving rise to diboronamine **12a**. While the formation of the hydride-substituted diboronamine **12a** was accompanied by degradation of the formed material and was therefore only characterized *in situ*, the analogous reaction sequence of cyclopentyl-substituted boron azide **3c** with HB­(C_6_F_5_)_2_ proceeded cleanly. The reduced diboronamine **12c** was conveniently isolated by removal of reaction solvent, extraction of product into hexane, filtration and solvent removal and compound **12c** was isolated as stable solid in nearly quantitative yield. The identity of diaminoborane was confirmed by the shift of one ^11^B NMR signal to the region typical for tricoordinated boranes (δ_B_ = 35.7 for **12a** and 34.5 for **12c**) and the appearance of a ^1^H NMR signal corresponding to the NH group (δ_H_ = 5.13 for **12a** and 5.98 for **12c**), as well as by mass spectrometry and IR spectroscopy.

We investigated the possibility of hydrolyzing compounds **12a** and **12c** as an alternate pathway to establish corresponding B-NH_2_ species. However, treatment of **12c** with water resulted in the formation of borinic acid **8c** in nearly quantitative yield, obtaining the same product as in direct azide reduction with the NaBH_4_/NiCl_2_ system. Reacting boron hydride **12a** under these conditions again led to complete decomposition, presumably due to the incompatibility of the functional groups in the expected product. When **12a** and **12c** were treated with acetic acid (a mild Brønsted acid), the reaction resulted in the formation of boron acetate zwitterionic species **13**, sufficiently robust and compatible with chromatographic purification. Formation of the acetate adduct was manifested by an upfield shift of both ^11^B NMR signals into the region characteristic for tetracoordinated boron species (δ_B_ for **13a** –1.2/–2.6 and for **13c** 2.6/–1.6, respectively), and by the appearance of two distinct peaks of NH_2_^+^ moiety within the broad range of 4.3–5.1 ppm, accompanying the characteristic sharp signal of the acetate fragment in ^1^H NMR (δ_H_ 1.94 for **13a** and 1.76 for **13c**) (for details see SI). The molecular structure of **13c** was additionally confirmed by SC-XRD (see the SI).

Reaction with bis­(trifluoromethylsulfonyl)­amide (triflimide, a strong Brønsted acid) led to the cleavage of the B–N bond proximal to the phenylpyridine moiety and the formation of **2c** as the major species, which was not isolated but its identity was confirmed by comparison of the *in situ* measured NMR spectra with an authentic sample. For detailed experimental procedures and characterization data, see SI.

Azide–alkyne [3 + 2] cycloaddition—often referred as the Huisgen or “click reaction”—is a typical transformation that exploits the dipolar nature of azides and has been employed in the synthesis of diverse boron substituted triazoles.^3,26^ However, in our case, even under rather forcing conditions (**3a** or **3c**, 1.5 equiv of PhC≡CH, 20 h, 100 °C in toluene, with or without 10 mol % CuI and *i*-Pr_2_NH), no triazole formation was observed, further highlighting the nonelectrophilic nature of our boron azides. We turned to a reactive strained cyclooctyne (Scheme 4), which typically undergoes rapid copper-free click reaction even at room temperature.^27^ In this case, boron azides **3a** and **3c** required heating to 60 °C overnight to achieve full conversion to triazoles **14a** and **14c**. In contrast, reaction with an electron-deficient alkyne dimethyl acetylenedicarboxylate (DMAD) proceeded somewhat faster under identical conditions, with complete conversion to **15a** and **15c** achieved within several hours. Reaction kinetics were monitored using ^1^H NMR, and the individual reaction profiles are summarized in Figure 6. Based on the obtained data, these reactions appear to follow the pseudo-first-order kinetics, with the observed rate constant *k* ´ decreasing in the order **15a** (**3a**/DMAD) > **15c** (**3c**/DMAD) > **14a** (**3a**/cyclooctyne) > **14c** (**3c**/cyclooctyne). These results confirm that the electronic activation of the alkyne plays a more important role in the reaction course than the steric hindrance of the boron azide. For completeness, bis-triazole derivatives **14b** and **15b** were also prepared from boron diazide **3b**. Formation of triazole products was evidenced by NMR, IR and HRMS and for **14a**–**c** and **15b** also by SC-XRD (for depiction of **14a**–**c** see Figure 7, for **15b** see SI). Additionally, triazoles **15a**–**c** exhibited a deep blue luminescence under UV irradiation; for discussion of their photophysical properties, see below.

**Scheme 4:**
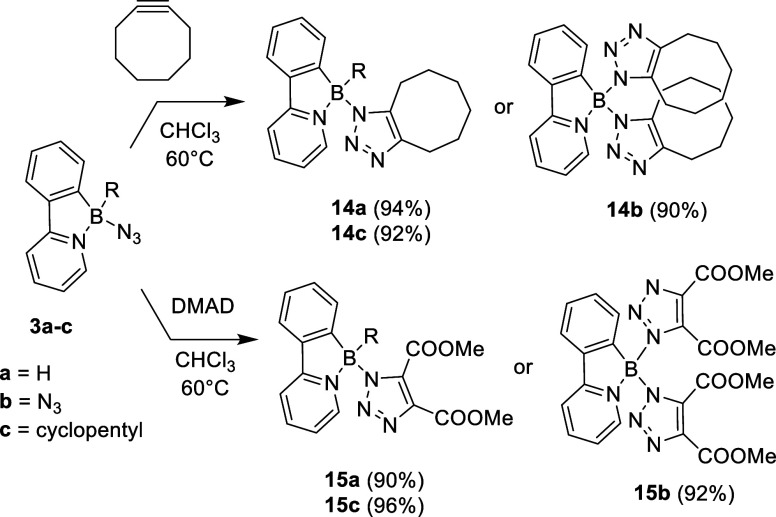
Cycloaddition Reactions of Azides **3a**–**c** with Cyclooctyne and Dimethyl Acetylenedicarboxylate (DMAD)

**Figure 6:**
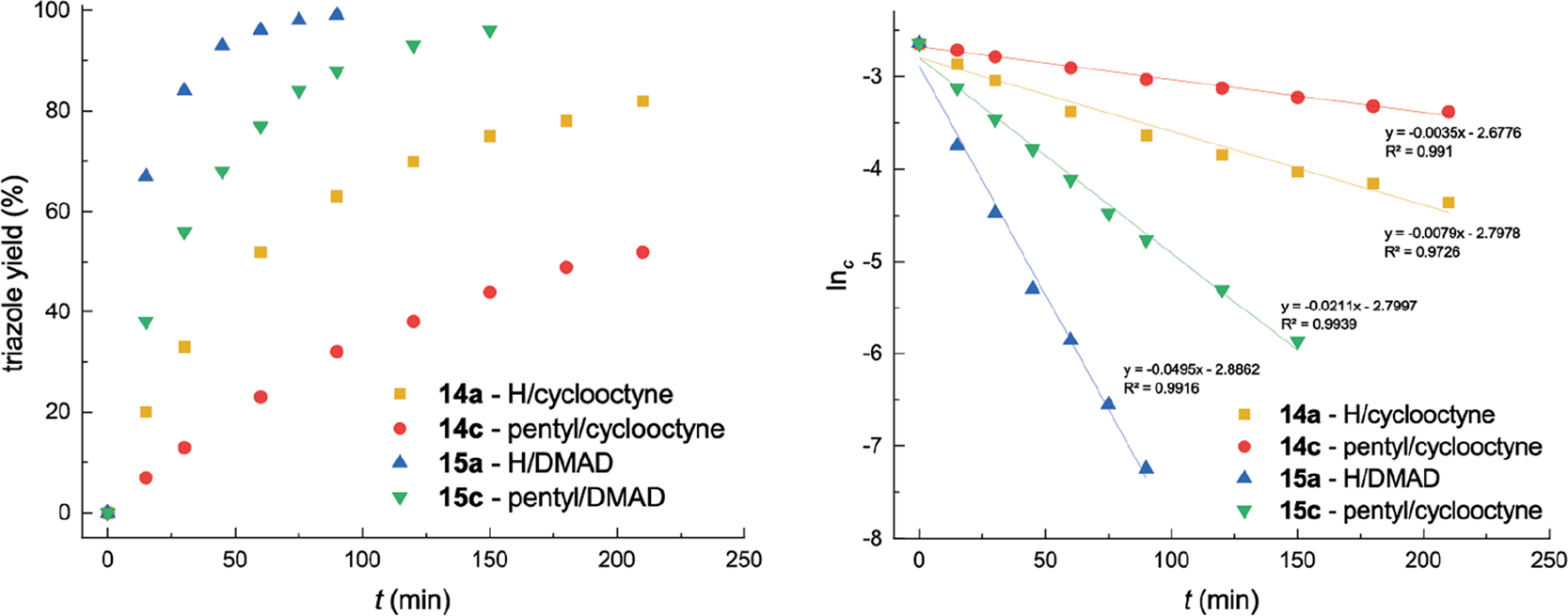
Kinetic profile of the reactivity of **3a** and **3c** toward cyclooctyne and dimethyl acetylenedicarboxylate (DMAD). Reaction conditions: 0.5 mmol of azide, 0.75 mmol of alkyne, 60 °C, CDCl_3_ (0.6 mL), triazole yield estimated by ^1^H NMR.

**Figure 7:**
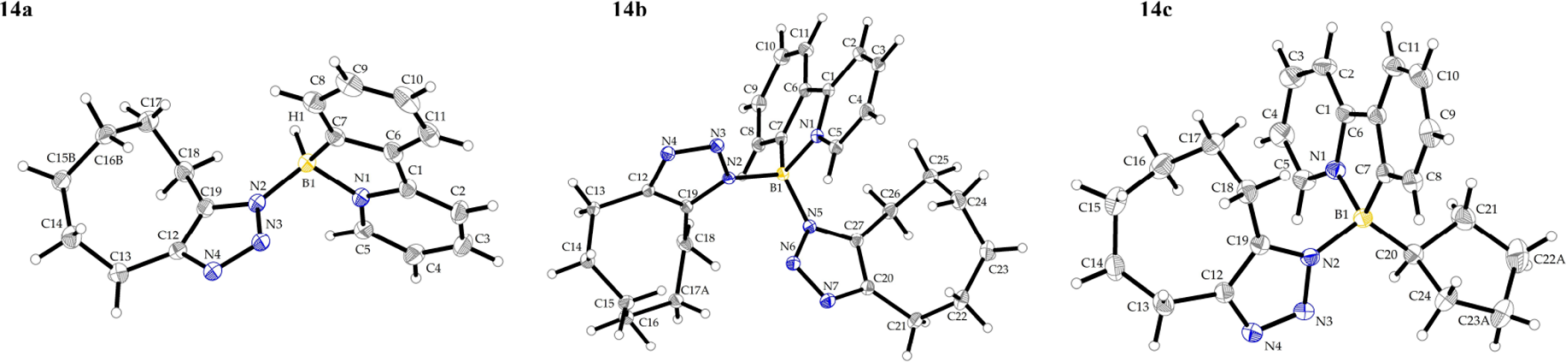
View of the molecular structure of **14a**–**c**. Selected bond lengths (Å) and angles (°) for **14a**: B1–N1 1.605(2), B1–C7 1.592(2), B1–N2 1.539(2), ΣN2_CNB_ 359.99; for **14b**: B1–N1 1.596(2), B1–C7 1.621(2), B1–N2 1.548(2), B1–N5 1.536(2), ΣN2_CNB_ 359.65, ΣN5_CNB_ 357.99; for **14c**: B1–N1 1.622(2), B1–C7 1.608(2), B1–N2 1.560(2), ΣN2_CNB_ 359.01.

The 1,3-dipolar cycloaddition also proceeds with an activated alkene, as manifested by the reaction of azide **3c** with succinimide (80 °C in toluene overnight, Scheme 5). HRMS analysis identified the product as bicyclic triazoline **16**. The reaction exhibits limited stereoselectivity, and compound **16** was obtained as a 2:1 mixture of exo- and endo- isomer (ratio estimated by ^1^H NMR) formed via syn-addition of the olefin to the boron-azide. The formation of the triazoline ring was evidenced by two sets of distinct aliphatic doublets in ^1^H NMR spectrum, each with a coupling constant ^3^ *J*_HH_ = 11 Hz, typical for *cis*-arrangement.^28^ A similar reaction was observed for azide **3a**; however, in this case, the outcome was compromised by the formation of undefined side reactions and gradual product decomposition (for details, see SI).

**Scheme 5:**
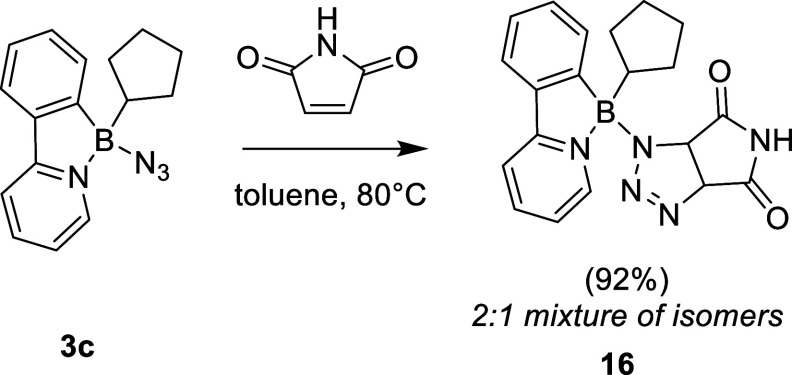
Cycloaddition of **3c** toward an Electron-Deficient Alkene

## Computational Investigations

We also employed computational approaches using the DFT model (see SI for details) in the investigation of the reactivity of prepared borane azides. The simplest borane azide **3a** was chosen as a model compound for this study. Notably, analysis of the frontier molecular orbitals revealed no significant contribution from the azide fragment from intrinsic bond orbital (IBO) derived HOMO nor from the corresponding LUMO (Figure 8), which correlates with the observed thermal and photochemical stability of reported azide compounds. However, for the cycloaddition pathway, a significant overlap was observed between LUMO+1 of **3a** and HOMO–1 of cyclooctyne and HOMO–2 of DMAD, respectively. Note that HOMO and other high-lying canonical orbitals are of the antibonding nature, i.e., **3a** acts as an electron acceptor (electron-poor 1,3-dipole), which is consistent both with the experiments and with the observation for boryl azides.^26b^ Moreover, LUMO of **3a** exhibits negligible contributions to it from-N_3_ and, e.g., HOMO of cyclooctyne is not “perpendicular” to its ethyne moiety of the C8-chain. In HOMO and HOMO–1 there is no contribution of the alkyne carbons to them in DMAD. On that basis, the most suitable orbital pairs responsible for the initial contact of **3a** with cyclooctyne and DMAD are LUMO+1 vs HOMO–1 and LUMO+1 and HOMO–2, respectively (see Figure 9), with the corresponding energy gaps of 1.5 and 2.0 eV. An analogous orbital analysis performed for the benchmark phenyl azide revealed that its high-lying occupied and low-lying unoccupied canonical IBO orbitals are of roughly similar shape, suggesting that the phenyl azide can act as an ambiphilic agent, with the nature of a dipolarophile being the decisive factor for the electronic initiation of the cycloaddition.^28^ Finally, NHC-boryl azides are type III dipoles, where the HOMO of the dipole and the LUMO of the dipolarophile play the key role, indicating that such azides are of electron-donating nature.^26^ The latter is attributed to the σ-donating abilities of the carbene toward the *p*_*z*_-orbital of the nitrogen atoms in the azide moiety. Localization of IBO-charges on the nitrogen atoms (values in parentheses) in B–N(−0.37)-N(0.34)-N(−0.27) for **3a** and C–N(−0.27)-N(0.34)-N(−0.17) for the phenyl azide, respectively, also distinguishes the electronic character of **3a** from that of the conventional phenyl azide, with the electron cloud in the latter being more delocalized.

**Figure 8:**
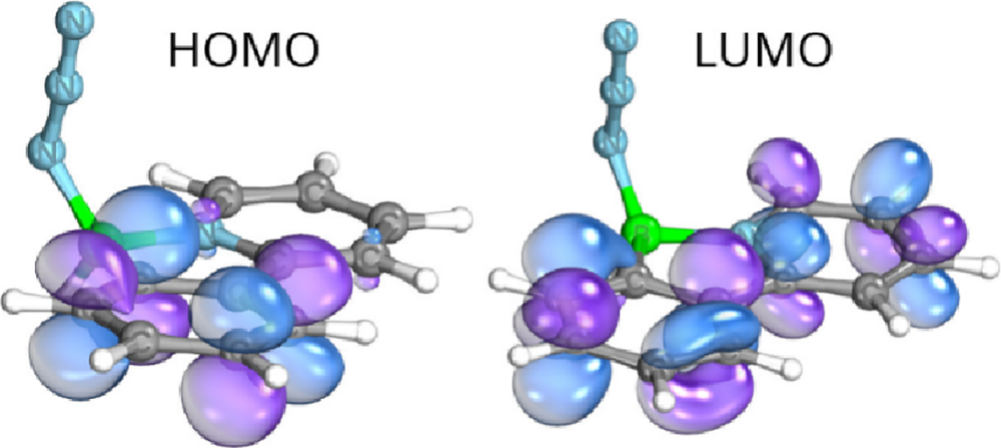
IBO-based frontier orbitals of **3a** showing no contribution from the N_3_ group.

**Figure 9:**
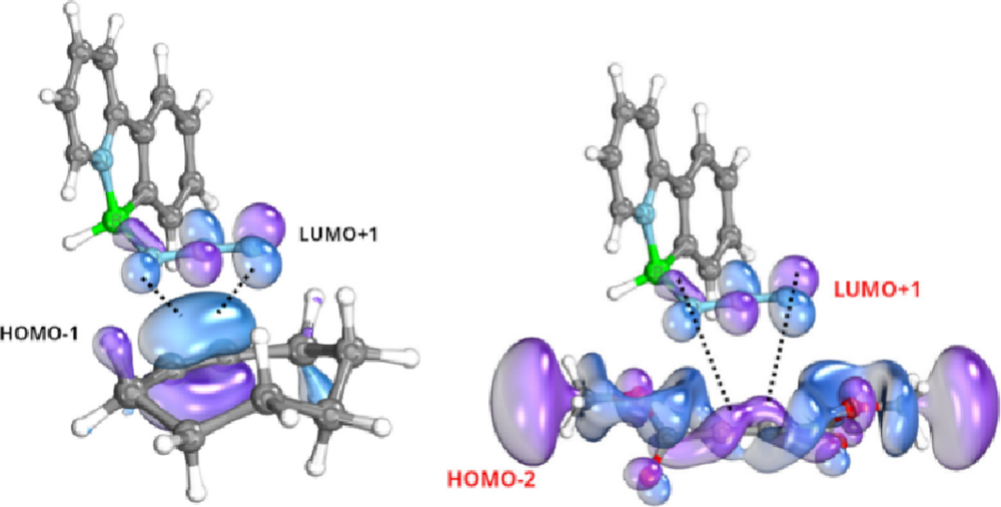
Overlaps between the suitably occupied Kohn–Sham frontier-like IBO orbitals indicating the initial attacks in the reactions of **3a** with cyclooctyne (left) and DMAD (right).

We further investigated the reaction pathways of 3 + 2 cycloaddition of **3a** with cyclooctyne and DMAD, respectively, at the B3LYP/6–31+G* level. Figure 10 illustrates the energetic profiles of both synchronous reactions, each involving a significant deformation of the almost linear N–N–N dipole in **3a**. As illustrated, the initial activation barriers are acceptably low, and both reactions are quite exothermic. These barrier values are, however, higher than those detected for the reaction of boryl azide with slightly different types of alkynes, e.g. 11.7 kcal/mol (an alkyne ester) and 9.1 kcal/mol (cyclooctyne, both at B3LYP/6–31G*).^26a^ Note that the transition state for the reaction of phenyl azide with cyclooctyne lies considerably lower, 8 kcal/mol, than that for **3a** with cyclooctyne. The point being that the repulsion between the outer nitrogen atoms in the phenyl azide is lower than in **3a** (see the above IBO charges), resulting in overcoming a greater repulsion in **3a** upon keeping the NNN angles in the corresponding transition states at almost the same magnitudes (143.3° for phenyl azide^29^ at B3LYP/6–31G* as qualitatively compared to 142.6° for **3a** at B3LYP/6–31+G*). The solid-state structure of **14a** (see Figure 7) offers comparison with that computed at the B3LYP/6–31+G* model chemistry. There is an overall agreement between the two types of molecular structures, e.g., the NNN bond angle in the crystal was refined to be 107.9(1)°, this internal coordinate being optimized to 108.3°. The remaining bond angles within the triazole ring do not significantly deviate from 108°, which is consistent with a regular pentagon. However, a direct comparison of the molecular geometries obtained in the solid state and *in silico* must be approached with caution.

**Figure 10:**
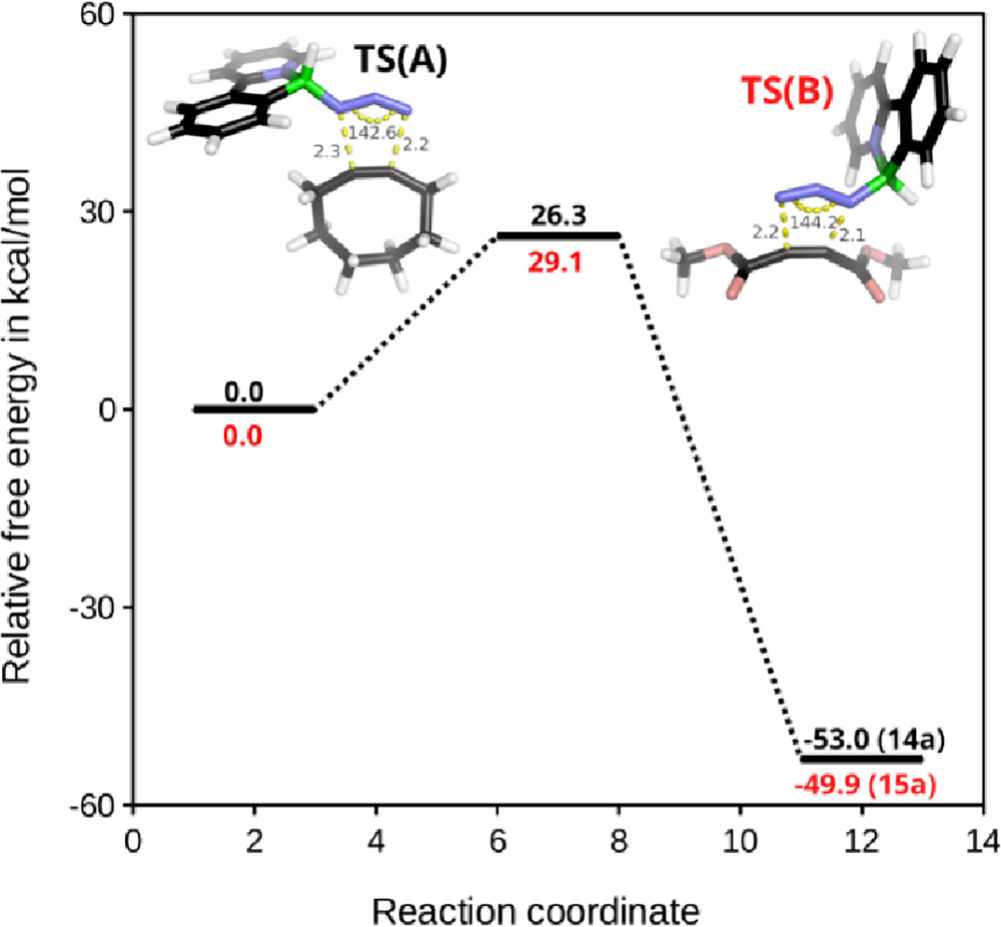
Reaction pathways of the reactions of **3a** with cycloctyne (black legends) and DMAD (red legends) as examined at the B3LYP/6–31+G* level of theory, TS is referring to the corresponding transition states characterized with imaginary frequencies of −362 cm^–1^ and −372 cm^–1^ for TS­(A) and TS­(B), respectively.

## Fluorescence Properties

Arylpyridine-chelated boranes represent a well-established structural motif in the field of luminophore design.^14^ While the nature of the substituents on the boron center typically exerts a marginal influence on the fluorescence emission, with these compounds generally emitting in the UV-blue region, more pronounced effects have been observed upon π-system extension^30^ or the introduction of weakly coordinating groups.^12^ Even in the present study, a series of compounds, including parent boron azides **3a**–**c**, exhibited weak UV-blue emission upon UV excitation, with fluorescence quantum yields (Φ_f_) below 0.01. However, a notable enhancement of fluorescence emission intensities were observed for boron triazoles **15a**–**c**, derived from DMAD, both in solution and solid state. In deaerated dichloromethane solution compounds **15a**–**c** displayed an absorption band in the UV-A region and a strong UV-blue fluorescence with the maxima at 375, 385, and 372 nm, and the corresponding quantum yields were 0.31, 0.74, and 0.32 respectively (Table 3 and Figure 11). Excitation spectra recorded at the emission maxima matched the absorption spectra, confirming that the observed emissions originated from the studied compounds. The higher quantum yield and longer fluorescence lifetime measured for bis-triazole **15b**, compared to the values obtained for **15a** and **15c**, may originate from the steric shielding of the boron atom, which leads to the stabilization of the excited singlet state against nonradiative deactivation. Similar effects were observed in our previous work.^12^

**Table 3:** Fluorescence Properties of **15a**–**c** in Dichloromethane, at Room Temperature^a^

	λ_max_/nm	λ_f_/nm	Φ_f_	τ_f_/ns
**15a**	321	375	0.31	4.1
**15b**	327	385	0.74	7.0
**15c**	317	372	0.32	4.2

a λ_max_ – absorption/excitation maximum; λ_f_ - fluorescence maximum (λ_exc_ = 300 nm); τ_f_ - fluorescence lifetime recorded at the maximum of emission (λ_exc_ = 340 nm); Φ_f_ fluorescence quantum yield (λ_exc_ = 320 nm, experimental error of Φ_f_ is ± 0.01).

**Figure 11:**
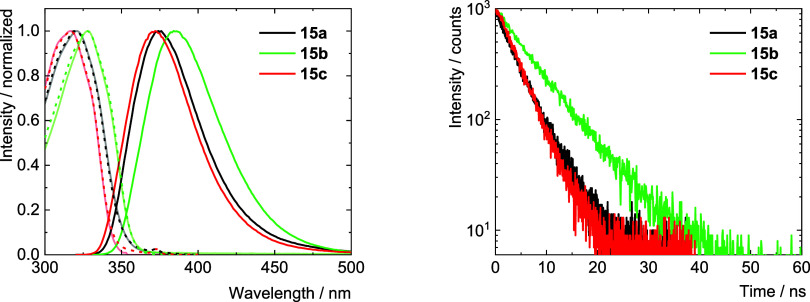
Photophysical properties of **15a**–**c** in dichloromethane at room temperature (left). Absorption spectra and fluorescence emission spectra were measured upon excitation at 300 nm, and excitation spectra were recorded at the emission maxima (dashed lines). Fluorescence decay kinetics were recorded at the emission maxima, excitation at 340 nm (right).

The fluorescence properties of **15a**–**c** were also studied in the solid state at room temperature. Upon excitation at 300 nm, strong deep blue fluorescence was observed with maxima at 439, 400, and 397 nm with associated quantum yields of 0.20, 0.48, and 0.29, respectively (Table 4 and Figure 12). Overall, the excitation edges and emission maxima were shifted to lower energies when compared to those measured in dichloromethane solutions, apparently as a result of intramolecular interactions and molecular packing in the solid state structure (for structure of **15b** see SI).

**Table 4:** Photophysical Properties of **15a**–**c** in the Solid State at Room Temperature^a^

	λ_max_/nm	λ_f_/nm	Φ_f_	τ_f_/ns
**15a**	386	439	0.20	3.1
**15b**	300	400	0.48	6.1
**15c**	333	397	0.29	5.7

a λ_max_ - excitation maximum; λ_f_ - fluorescence maximum (λ_exc_ = 300 nm); τ_f_ - fluorescence lifetime recorded at the emission maxima (λ_exc_ = 340 nm); Φ_f_ fluorescence quantum yield (λ_exc_ = 380, 300, and 330 nm, respectively, experimental error of Φ_f_ is ± 0.01).

**Figure 12:**
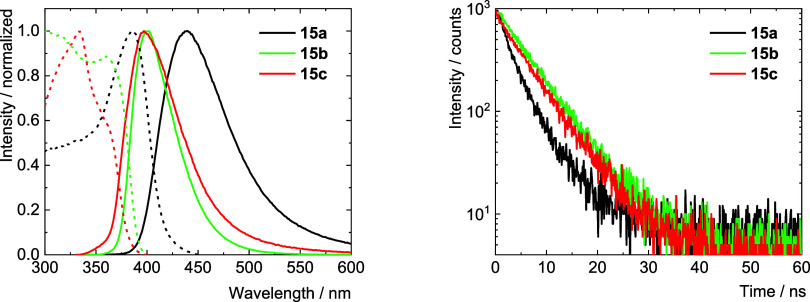
Photophysical properties of **15a**–**c** in the solid state, at room temperature (left). Fluorescence emission spectra upon excitation at 300 nm and excitation spectra were recorded at 460, 420, and 420 nm, respectively (dashed lines). Fluorescence decay kinetics recorded at the emission maxima, excitation at 340 nm (right).

Excited states in the simplest system **15a** were investigated computationally using the configuration interaction singles approach (CIS). The geometry used for these calculations was derived from the TS­(B) geometry (see Figure 10). Triplet states were excluded from further consideration, as their computed oscillator strengths were all zero. The detected singlet states with oscillator strength of 0.50 were found qualitatively consistent with the experimental λ_max_ of **15a** with the corresponding singlet electron excitation energy 4.25 eV (292 nm) at the SMD/CH_2_Cl_2_/CIS/6–311+G**//B3LYP/6–31+G* level of theory (see SI for details). This singlet excitation primarily corresponds to a HOMO→LUMO transition, with a wave function coefficient of 0.63, and to a lesser extent to a HOMO→(LUMO+1) transition (coefficient −0.10). Squaring these coefficients provides a measure of each configuratiońs contribution to the overall singlet state, indicating that the HOMO→LUMO transition is the dominant one. The monoexcitation is clearly localized within the phenylpyridine-boron fragment of the molecule; see Figure 13.

**Figure 13:**
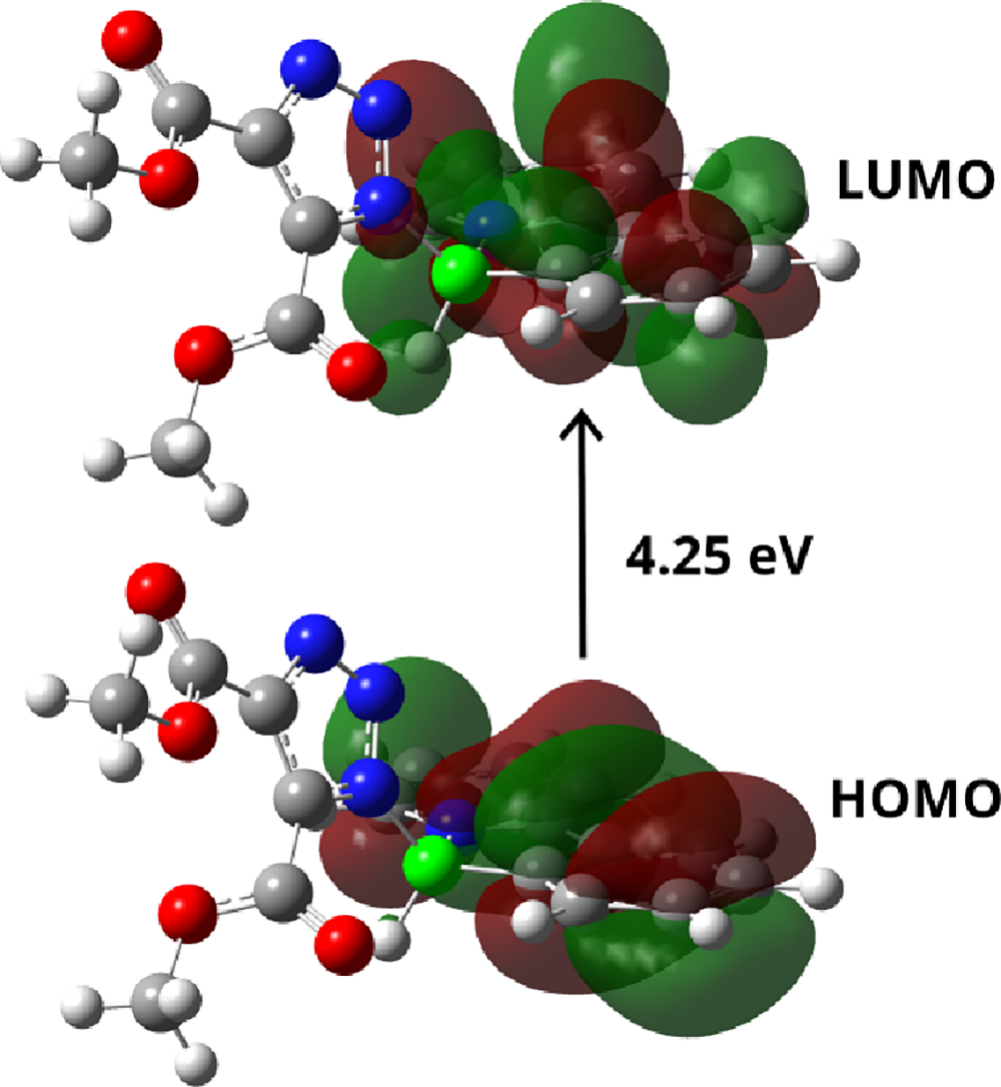
Most intensive monoexcitation from the ground state to the first singlet state of **15a** modeled with CIS-based frontier orbitals.

## Conclusions

In this study, we report a series of phenylpyridine-based boron azides and the exploration of their reactivity landscape. Three phenylpyridine-stabilized boron azides were synthesized: a hydridic monoazide, a sterically hindered cyclopentyl-substituted monoazide, and a boron diazide. These compounds are readily accessible via nucleophilic substitution from *in situ* generated borenium-type intermediates and exhibit notable photochemical and thermal stability.

Reactivity studies showed that the investigated boron azides react poorly with nucleophiles in phosphine-mediated Staudinger-type transformations, although small amounts of an unexpected dibenzo-1,3,2-diazaborinine were formed as byproduct. In reactions with IMes, the anticipated triazene products were not obtained; instead, either azide elimination occurred, yielding a boron-NHC adduct, or the compound decomposed. The reported borane azides readily form adducts with other boron-based Lewis acids, giving structures with a bridging azide motif. Attempts at reduction of the azide moiety did not afford the expected amines but instead triggered azide elimination, producing borinic acids. Kinetic and computational analyses revealed unexpectedly high activation barriers for [3 + 2] cycloadditions with activated alkynes and proceed via an inverse electron-demand pathway. Quantum chemical calculations corroborated these observations, showing that, unlike typical 1,3-dipoles, these boron azides interact with cyclooctyne and DMAD primarily through their higher-lying LUMO+1 orbital, indicating a deviation from a conventional HOMO–LUMO-driven reactivity pathway.

Additionally, selected cycloaddition products exhibited strong UV-A fluorescence in solution (λ_max_ 370–385 nm) and deep-blue fluorescence (400–440 nm) in the solid state, with quantum yields up to 0.74 (in dichloromethane solution) and 0.48 (in the solid state). These findings expand the understanding of boron azide reactivity in main-group chemistry and suggest potential applications of boron triazole derivatives as robust UV-A or deep-blue fluorophores.

## Supplementary Material



## Notes

Description of experimental procedures including complete characterization of prepared compounds, copies of the NMR spectra, crystallographic parameters, additional structure diagrams and further computational details (PDF)
