# Supplementary material for: Phenylpyridine-Based Boron Azides: Tuning Reactivity and Accessing Fluorescent Triazoles
Source: Inorg Chem. 2025 Sep 5;64(36):18556–66. doi: 10.1021/acs.inorgchem.5c03363 (PMC12442104; doi:10.1021/acs.inorgchem.5c03363)
Supplement: Supplementary file 1 [file ic5c03363_si_001.pdf]

Supporting Information

Phenylpyridine-Based Boron Azides: Tuning Reactivity and Accessing Fluorescent Triazoles

Karel Škoch[a*], Michaela Buziková[a], Drahomír Hnyk[a], Miroslava Litecká[a], Anna Vykydalová[a,b], Dmytro Bavol[a], Kamil Lang[a], Kaplan Kirakci[a]

[a]Institute of Inorganic Chemistry of Czech Academy of Sciences, Husinec-Řež 1001, 250 68, Czech Republic

[b] Polymer Institute, Slovak Academy of Sciences, Dúbravská cesta 9, 845 41 Bratislava, Slovakia

E-mail: skoch@iic.cas.cz

Table of contents

Experimental Part 2

Preparation of boron azides **3a-c** 4

Preparation of **4** 15

Thermal stability of **3a-c** 19

Reactivity of prepared azides towards phosphine nucleophiles, preparation of **6** and **7** 24

Reductive chemistry of boron azides, preparation of **8-13** 34

Cycloaddition chemistry, preparation of **14-16** 64

Computational details 91

Supporting Information – References 97

Experimental Part

Experiments were performed under dry argon atmosphere using standard Schlenk-type glassware and/or argon filled glovebox, unless stated otherwise. Reaction solvents hexane, toluene and tetrahydrofuran were dried by refluxing over sodium metal in the presence of benzophenone ketyl radical under argon atmosphere. Dichloromethane was purified by distillation from CaH_2 in argon atmosphere. Dry solvents were stored over activated 4Å molecular sieves. Solvents used for extractions and/or column chromatography were used as received. Deuterated solvents were distilled from CaH_2 and stored over 4Å molecular sieves under argon atmosphere. All chemicals were purchased from commercially available resources (Sigma-Aldrich, BLD-Pharm, TCI, ABCR) and used as received, unless stated otherwise. Compound **1, 2a**,[S1] $HB(C_6F_5)_2$,[S2] and cyclooctyne[S3] were prepared according to reported procedures.

CAUTION: Sodium azide and azide products employed in this work are toxic and potentially explosive. All procedures were therefore carried out in small scale in the glovebox or in well ventilated fume hood using appropriate protective equipment and following standard safety protocols. No explosive decomposition was observed in our experiments; however, strict safety measures should always be followed.

NMR spectra were recorded on JEOL Delta 600 spectrometer at 20 °C. ^{1}H and ^{13}C NMR chemical shifts (δ in ppm) are given relative to TMS and referenced to the residue solvent signal ($CDCl_3$: δ_H = 7.26, δ_C = 77.0, CD_2Cl_2: δ_H =5.32, δ_C = 53.8). ^{11}B, ^{19}F and ^{31}P are referenced according to the primary reference for the unified chemical shift scale following IUPAC recommendation [R.K. Harris. E.D. Becker, S.M. Cabral de Menezes, R. Goodfellow, P. Granger *Pure Appl. Chem.* **2001**, *73*, 1795-1818]. The multiplicity of the signals is indicated as s, d, t, q or m for singlets, doublets, triplets, quartets or multiplets, coupling constants *J* are given in Hertz as positive values regardless of their individual signs. Assignment of the peaks was supported by 2D experiments (COSY, HSQC and HMBC). MestreNova software package was used for analysing the spectra.

Elemental Analysis was performed on FlashSmart™ Elemental Analyzer. Tin capsules were filled with samples and sealed in glovebox atmosphere. Mass spectrometry measurements were performed on Orbitrap Exploris™ 120 instrument using electrospray ionization. Infrared spectra were collected on Nexus 670 FT-IR spectrometer. Air stable samples were analysed by ATR method, samples sensitive to ambient conditions were measured as KBr pellets prepared in glovebox atmosphere. Samples were measured in range 4000-400 cm^{-1} and the relative intensities are given in parentheses (s, m, w for strong, medium and weak). Simultaneous thermogravimetric and differential scanning calorimetry analysis (TGA/DSC) was performed as simultaneous thermal analysis (STA) on Netzsch STA449 F1 Jupiter.

UV-Vis absorption and fluorescent properties were recorded on Perkin Elmer Lambda 35 spectrometer and Fluorolog 3 spectrometer equipped with a cooled TBX-05-C photon detection module (Horiba Jobin Yvon). Fluorescence lifetime experiments were performed upon excitation at 340 nm and decay curves were fitted to exponential function by the iterative reconvolution of the DAS6 software. Fluorescence quantum yields were measured using Quantaurus QY C11347-1 spectrometer (Hamamatsu).

X-ray diffraction analysis were performed on Rigaku XtaLAB Synergy S diffractometer equipped with Mo (Mo/K_α radiation; λ = 0.71073 Å) and Cu (Cu/Kα radiation; λ = 1.54184 Å) with micro-focus X-ray source and Hybrid Pixel Array Detector (HyPix-6000HE). Oxford Cryosystems (Cryostream 800) cooling device was used for data collection at a temperature of 100 K and 150 K (KSK331-tw-aP_twin1_hklf4). CrysAlisPro software[S4] was used for data collection and cell refinement, data reduction and absorption correction. Data were corrected for absorption effects using empirical

absorption correction (spherical harmonics), implemented in SCALE3 ABSPACK scaling algorithm and numerical absorption correction based on a Gaussian integration over a multifaceted crystal model or analytical absorption correction (only KSK185_40_Cu_100-aa) was applied as described by Coppens *et al.*[S5] and North *et al.*[S6] The structures of prepared compounds were solved with the ShelXT[S7] structure solution program using Intrinsic Phasing and refined with the refinement package using SHELXL[S8] Least Squares minimisation implemented in Olex2[S9]. Anisotropic displacement parameters were refined for all non-H atoms. The hydrogen atoms were calculated to idealised positions or found in the the Furier map (Boronic hydrogens). For crystallographic data and structure refinement see ESI tables. Molecular graphic for all crystal structures was generated using DIAMOND software.[S10]

Crystallographic data for structural analysis has been deposited with the Cambridge Crystallographic Data Centre, CCDC nos. 2445679-2445691. Copies of this information may be obtained free of charge from The Director, CCDC, 12 Union Road, Cambridge CB2 1EY, UK (fax: +44-1223-336033; e-mail: deposit@ccdc.cam.ac.uk or www: http://www.ccdc.cam.ac.uk).

Preparation of boron azides **3a-c**

Preparation of **3a**

Method 1

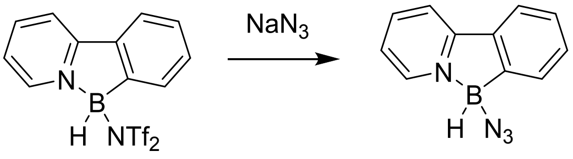

2a **3a**

Compound **2a** (223 mg, 0.500 mmol) and NaN_3 (97.5 mg, 1.50 mmol) were suspended in dry tetrahydrofuran (10 mL) and stirred overnight at room temperature. Reaction mixture was filtered and volatiles removed *in vacuo*. Oily residue was purified by column chromatography on silica gel and dichloromethane as eluent. Product **3a** was obtained as a colourless oil which gradually solidified (71.7 mg, 0.345 mmol, 79% yield).

Method 2

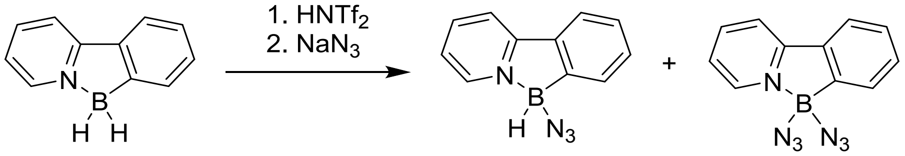

1 **3a** **3b**

Borane **1** (334 mg, 2.00 mmol) was dissolved in dry dichloromethane (5 mL) and subsequently a solution of triflimic acid (562 mg, 2.00 mmol) in dichloromethane (10 mL) was added portion wise. Effervescence occurred and the mixture was stirred for additional 30 minutes at room temperature. Volatiles were removed *in vacuo* and the oily residue was washed with hexane (2x5 mL), subsequently treated with NaN_3 (390 mg, 6.00 mmol). The mixture was then suspended in tetrahydrofuran (10 mL) and stirred overnight at room temperature. Reaction mixture was filtered and volatiles were removed *in vacuo*. The oily residue was purified by column chromatography on silica gel with dichloromethane as eluent. Boron azide **3a** was obtained as a colourless oil which gradually solidified (269 mg, 1.29 mmol, 65% yield). Additionally, fractions containing reaction by-product were isolated alongside. This by-product was identified as the boron diazide **3b** (35 mg, 0.14 mmol, 7% yield).

Characterization data for boron azide **3a**

Elemental analysis calculated for $C_{11}H_9N_4B$ (208.0): C 63.51, H 4.36, N 26.93; found C 63.31, H 4.34, N 26.76.

IR (KBr) ν: 2955 (w), 2925 (w), 2854 (w), **2393 (m, BH)**, **2096 (vs, N_3)**, 1622 (s), 1563 (w), 1486 (s), 1447 (m), 1329 (m), 1305 (s), 1286 (m), 1271 (m), 1167 (s), 1130 (m), 1109 (m), 1053 (s), 1006 (m), 963 (w), 948 (w), 878 (w), 843 (m), 800 (w), 766 (m), 738 (s), 665 (m), 634 (w), 586 (w), 559 (w), 466 (w), 413 (m) cm^{-1}.

^{1}H NMR (600 MHz, $CDCl_3$, 293 K): δ = [8.59 (d, $^3J_{HH}$ = 5.6 Hz), 8.08 (dd, $^3J_{HH} \approx {}^3J_{HH}$ = 7.6 Hz), 7.93 (d, $^3J_{HH}$ = 7.7 Hz), 7.45 (dd, $^3J_{HH}$ = 7.5 Hz, $^3J_{HH}$ = 5.6 Hz)](each 1H, pyridyl), [7.80 (d, $^3J_{HH}$ = 7.3 Hz),

7.78 (d, $^3J_{HH}$ = 7.1 Hz), 7.51 (dd, $^3J_{HH} \approx {}^3J_{HH}$ = 7.2 Hz), 7.39 (dd, $^3J_{HH} \approx {}^3J_{HH}$ = 7.2 Hz)](each 1H, C_6H_4), 3.92 (br, 1H, BH).

^{13}C{^{1}H} NMR (151 MHz, $CDCl_3$, 293 K): δ = [157.7 (*i*-C), 143.7 (CH), 142.0 (CH), 122.4 (CH), 118.1 (CH)](pyridyl), [152.8 (br, B-C), 136.4 (*i*-C), 131.6 (CH), 130.8 (CH), 127.5 (CH), 121.7 (CH)](C_6H_4).

^{11}B{^{1}H} NMR (193 MHz, $CDCl_3$, 293 K): δ = –0.4 ($\nu_{1/2} \approx$ 70 Hz).

^{11}B NMR (193 MHz, $CDCl_3$, 293 K): δ = –0.4 (d, $^1J_{BH}$ = 110 Hz).

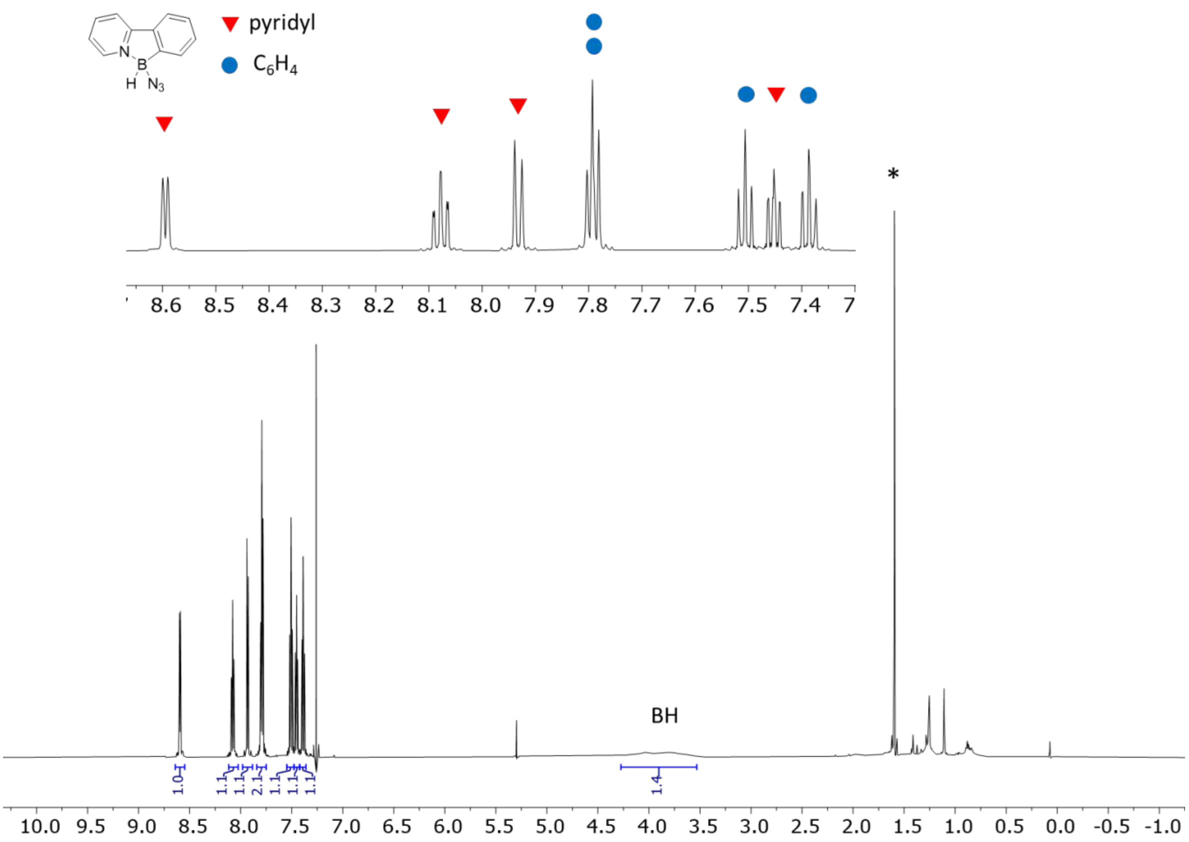

Figure S1: **^{1}H NMR** ($CDCl_3$, 600 MHz, 293 K) spectrum of compound **3a**. Asterisk denotes water residue present in the solvent.

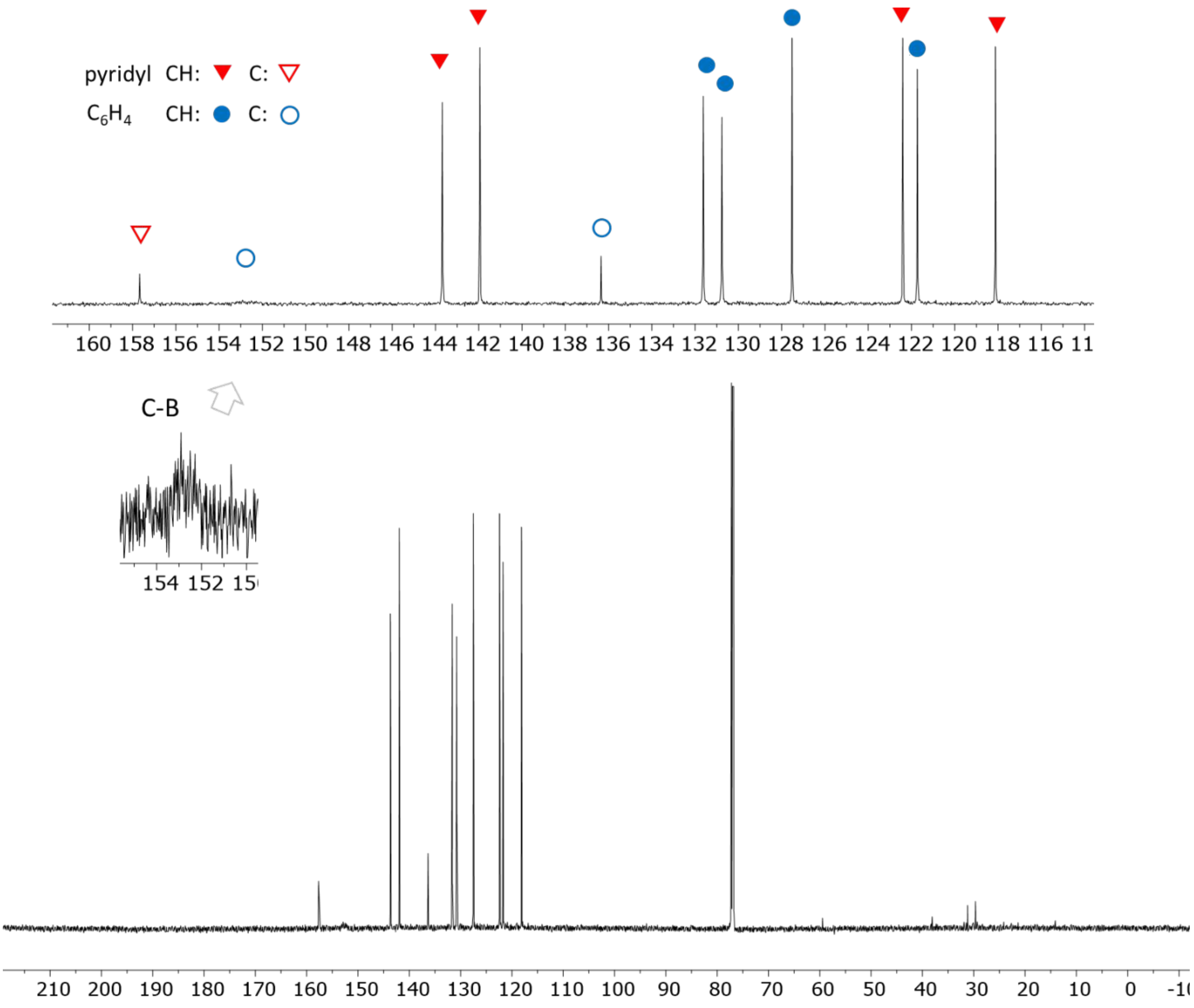

Figure S2: **$^{13}C\{^1H\}$ NMR** (151 MHz, $CDCl_3$, 293 K) spectrum of compound **3a**.

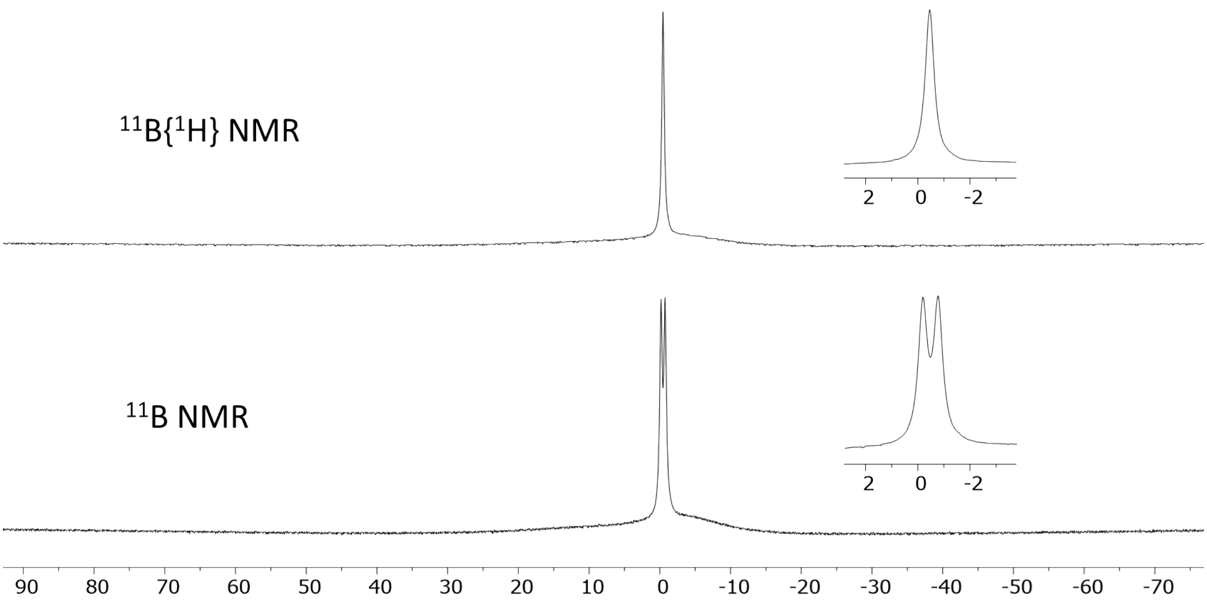

Figure S3: **^{11}B** and **$^{11}B\{^1H\}$ NMR** (193 MHz, $CDCl_3$, 293 K) spectra of compound **3a**.

Crystal structure determination **of 3a [KSK331-tw-aP_twin1_hklf4]**

A crystal suitable for SC-XRD structure determination was obtained by slow evaporation of solution of **3a** in dichloromethane:heptane mixture (ca. 1:1) under ambient conditions.

Crystal Data for $C_{11}H_9BN_4$ (M =208.03 g/mol): triclinic, space group P–1 (no. 2), a = 7.2300(3) Å, b = 9.9856(4) Å, c = 14.4866(3) Å, α = 90.039(3)°, β = 99.237(2)°, γ = 90.341(4)°, V = 1032.29(6) Å^3, Z = 4, T = 150.00(10) K, μ(Cu Kα) = 0.670 mm^{-1}, $Dcalc$ = 1.339 g/cm^3, 9406 reflections measured (6.182° ≤ 2Θ ≤ 160.916°), 9406 unique (R_{int} = ?, R_{sigma} = 0.0082) which were used in all calculations. The final R_1 was 0.0702 (I > 2σ(I)) and wR_2 was 0.2056 (all data). **CCDC: 2445683**

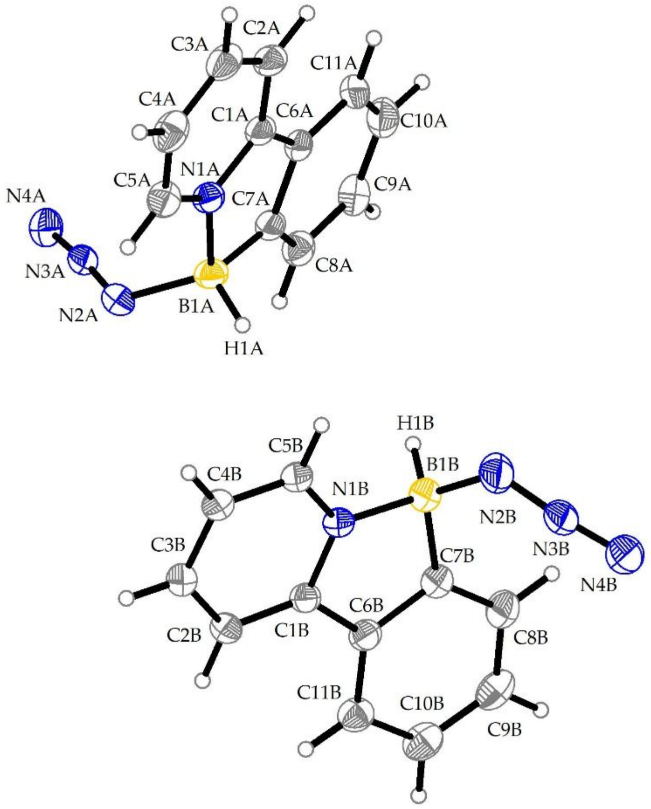

Figure S4: Crystal structure of **3a** (thermal ellipsoids shown at 30 % probability level).

Characterization data for boron diazide **3b**:

Elemental analysis calculated for $C_{11}H_8N_7B$ (249.0): C 53.05, H 3.24, N 39.37; found C 53.41, H 3.19, N 39.17

HRMS calculated for $C_{11}H_8BN_7Na^+$ $[M+Na]^+$: 272.0826, found 272.0815.

IR (KBr) ν: 2955 (w), 2924 (w), 2854 (w), **2094 (vs, N_3)**, 1623 (s), 1567 (w), 1488 (s), 1449 (m), 1439 (m), 1374 (w), 1321 (m), 1308 (m), 1290 (s), 1167 (s), 1132 (m), 1105 (w), 1082 (m), 1014 (w), 951 (w), 926 (s), 915 (s), 887 (s), 865 (s), 833 (m), 795 (w), 762 (s), 745 (m), 729 (s), 662 (m), 642 (m), 604 (w), 580 (m), 548 (w), 415 (m) cm^{-1}.

^{1}H NMR (600 MHz, $CDCl_3$, 293 K): δ = [8.48 (d, $^3J_{HH}$ = 5.7 Hz), 8.17 (dd, $^3J_{HH} \approx {}^3J_{HH}$ = 7.8 Hz), 7.95 (d, $^3J_{HH}$ = 7.8 Hz), 7.45 (dd, $^3J_{HH}$ = 7.8 Hz, $^3J_{HH}$ = 5.7 Hz)](each 1H, pyridyl), [7.82 (d, $^3J_{HH}$ = 7.5 Hz), 7.80 (d, $^3J_{HH}$ = 7.4 Hz), 7.58 (dd, $^3J_{HH} \approx {}^3J_{HH}$ = 7.6 Hz), 7.47 (dd, $^3J_{HH} \approx {}^3J_{HH}$ = 7.6 Hz)](each 1H, C_6H_4).

^{13}C{^{1}H} NMR (151 MHz, $CDCl_3$, 293 K): δ = [156.7 (*i*-C), 143.7 (CH), 142.2 (CH), 123.6 (CH), 118.4 (CH)](pyridyl), [145.3 (br, B-C), 136.1 (*i*-C), 132.6 (CH), 130.5 (CH), 129.2 (CH), 122.3 (CH)](C_6H_4).

^{11}B{^{1}H} NMR (193 MHz, $CDCl_3$, 293 K): δ = 3.5 ($\nu_{1/2} \approx$ 50 Hz).

^{11}B NMR (193 MHz, $CDCl_3$, 293 K): δ = 3.5 ($\nu_{1/2} \approx$ 50 Hz).

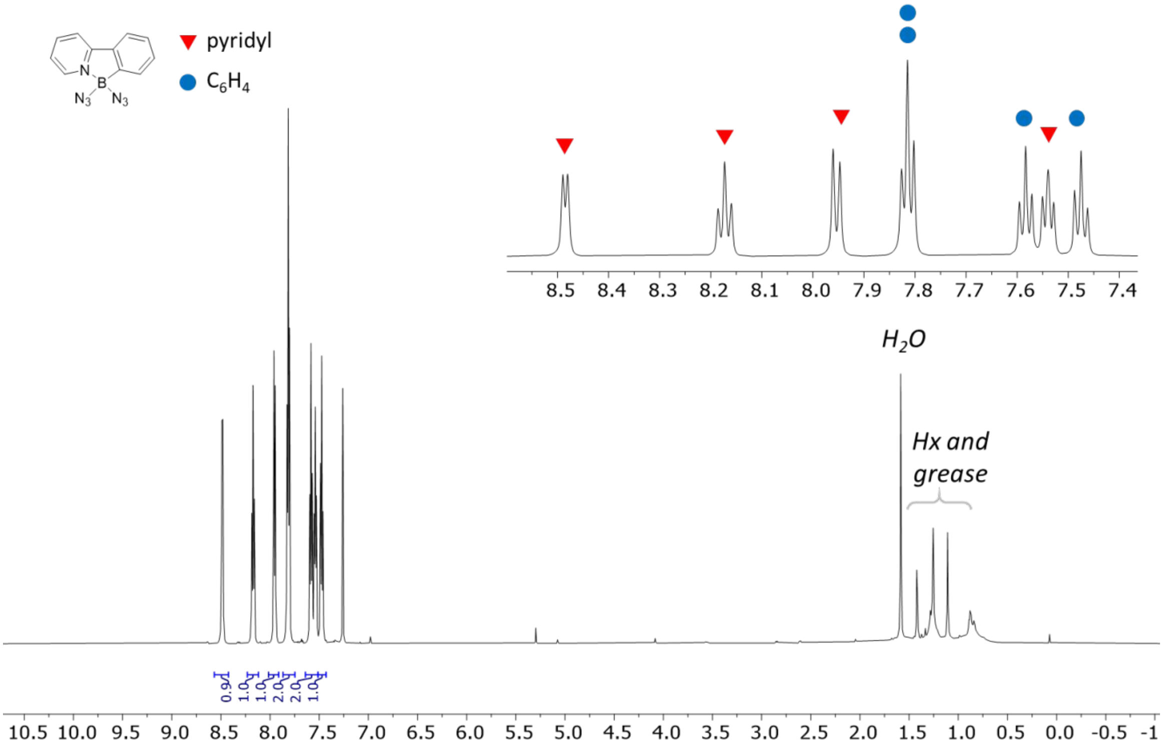

Figure S5: **^{1}H NMR** ($CDCl_3$, 600 MHz, 293 K) spectrum of compound **3b**, Hx denotes residual hexane.

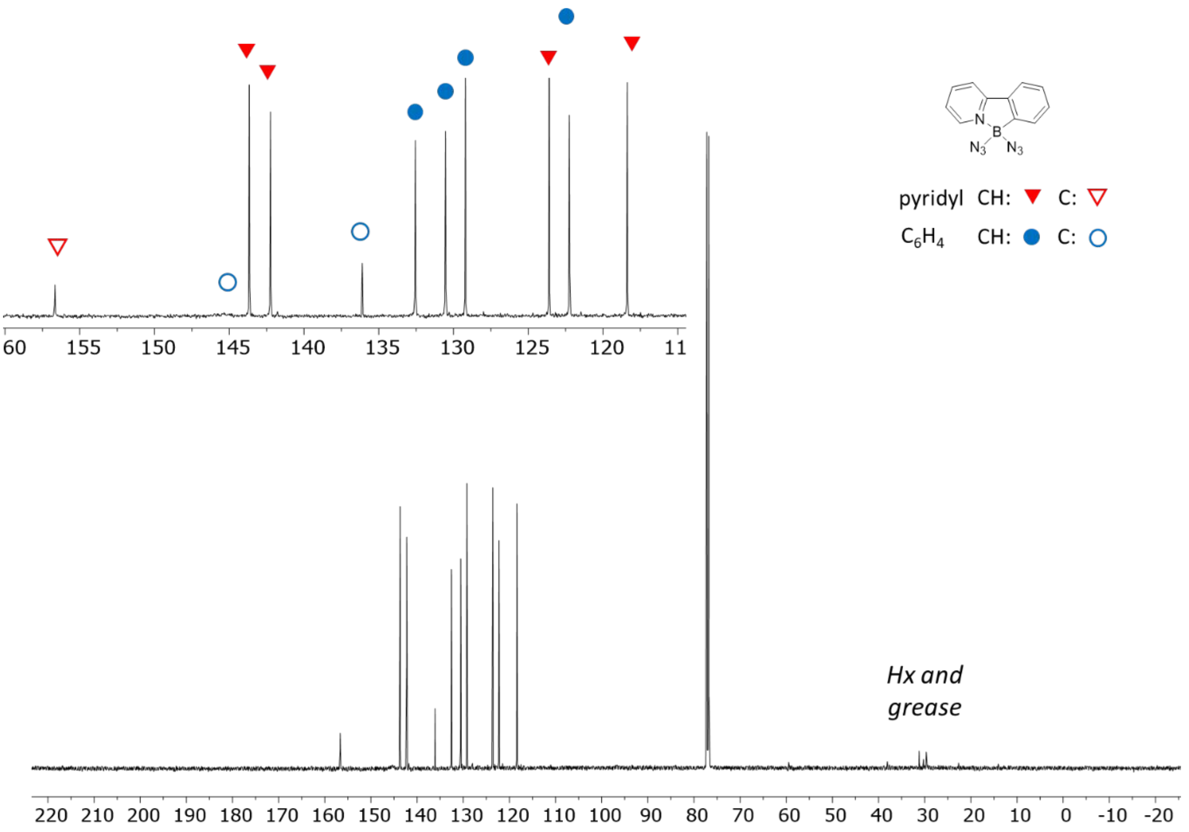

Figure S6: **^{13}C{^{1}H} NMR** (151 MHz, $CDCl_3$, 293 K) spectrum of compound **3b**, Hx denotes residual hexane.

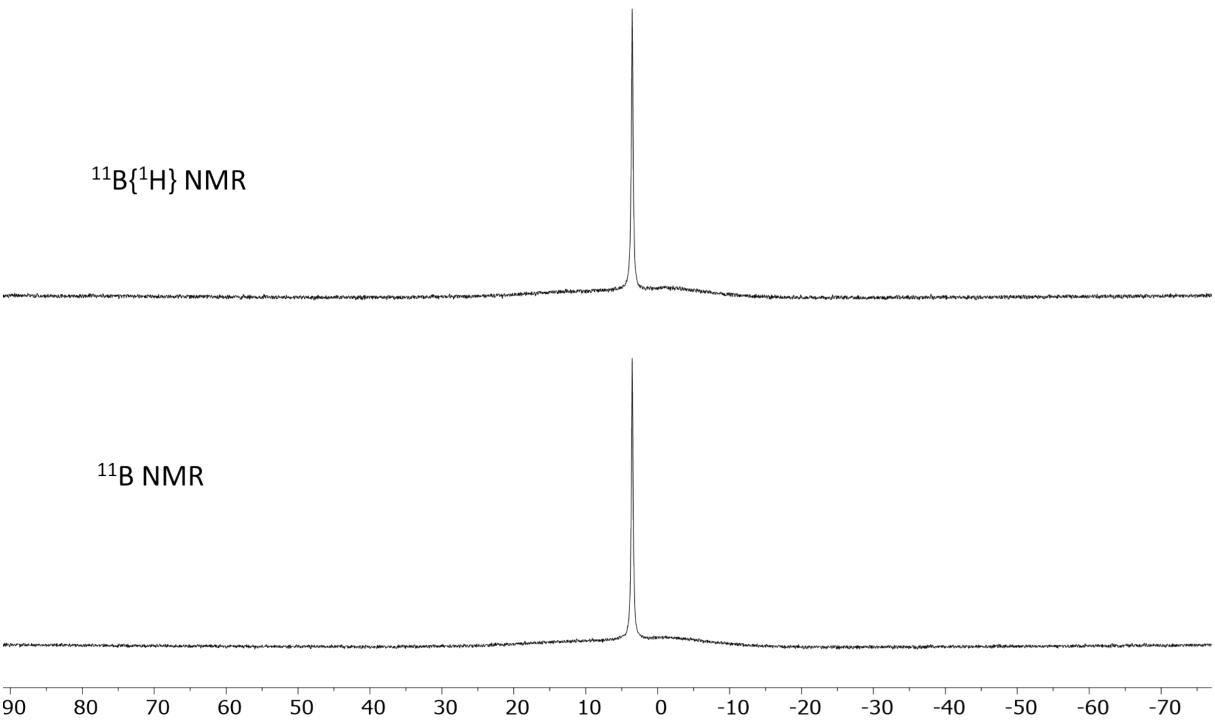

Figure S7: **^{11}B** and **^{11}B{^{1}H} NMR** (193 MHz, $CDCl_3$, 293 K) spectra of compound **3b**.

Crystal structure determination of **3b [KSK_BN3_2-gg]**

A crystal suitable for SC-XRD structure determination was obtained by slow evaporation of a solution of **3b** in dichloromethane:heptane mixture (ca. 1:1) under ambient conditions.

Crystal Data for $C_{11}H_8BN_7$ (M =249.05 g/mol): monoclinic, space group P2$_1$/c (no. 14), a = 8.7752(3) Å, b = 6.8782(3) Å, c = 19.0665(7) Å, β = 91.609(3)°, V = 1150.35(8) Å^3, Z = 4, T = 99.99(10) K, μ(Cu Kα) = 0.781 mm^{-1}, $Dcalc$ = 1.438 g/cm^3, 5021 reflections measured (9.28° ≤ 2Θ ≤ 133.194°), 2021 unique (R_{int} = 0.0373, R_{sigma} = 0.0479) which were used in all calculations. The final R_1 was 0.0365 (I > 2σ(I)) and wR_2 was 0.0992 (all data). **CCDC: 2445681**

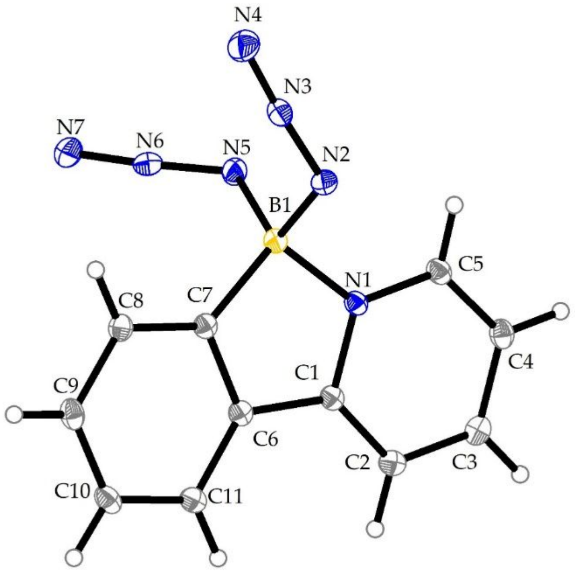

Figure S8: Crystal structure of **3b** (thermal ellipsoids shown at 30 % probability level).

Preparation of **3c**

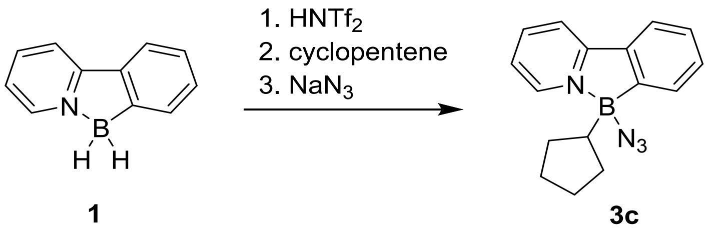

Borane **1** (668 mg, 4.00 mmol) was dissolved in dry dichloromethane (10 mL) and subsequently solution of triflimic acid (1.12 g, 4.00 mmol) in dichloromethane (20 mL) was added portion wise. Effervescence occurred and the mixture was stirred for 20 minutes at room temperature. Subsequently, excess of cyclopentene (0.5 g, ca 8 mm ol) was added. The mixture was stirred for additional 30 minutes and subsequently volatiles were removed *in vacuo*. The residue was washed with hexane (2x 5 mL) and subsequently treated with NaN_3 (780 mg, 12.0 mmol). The mixture was suspended in tetrahydrofuran (10 mL) and stirred overnight at room temperature. Reaction mixture was filtered and volatiles were removed *in vacuo*. The oily residue was purified by column chromatography on silica gel, using ethylacetate hexane 1:1 mixture as eluent. Product **3c** was obtained as a white solid (824 mg, 2.98 mmol, 75% yield).

Elemental analysis calculated for $C_{16}H_{17}N_4B$ (276.2): C 69.59, H 6.21, N 20.29; found C 69.96, H 6.36, N 20.56.

IR (ATR) ν: 2937 (w), 2860 (w), 2846 (w), 2832 (w), **2096 (vs, N_3)**, 1621 (s), 1572 (w), 1484 (s), 1446 (m), 1328 (w), 1308 (s), 1287 (m), 1272 (w), 1170 (w), 1158 (m), 1129 (m), 1098 (w), 1057 (m), 1012 (m), 987 (w), 959 (m), 887 (m), 865 (w), 802 (w), 762 (s), 745 (s), 735 (s), 641 (m), 633 (m), 590 (w), 553 (w), 500 (w), 460 (w), 420 (m) cm^{-1}.

^{1}H NMR (600 MHz, $CDCl_3$, 293 K): δ = [8.48 (d, $^3J_{HH}$ = 5.7 Hz), 8.06 (dd, $^3J_{HH} \approx {}^3J_{HH}$ = 7.8 Hz), 7.93 (d, $^3J_{HH}$ = 7.8 Hz), 7.43 (dd, $^3J_{HH}$ = 7.8 Hz, $^3J_{HH}$ = 5.7 Hz)](each 1H, pyridyl), [7.80 (d, $^3J_{HH}$ = 7.6 Hz), 7.77 (d, $^3J_{HH}$ = 7.3 Hz), 7.48 (dd, $^3J_{HH} \approx {}^3J_{HH}$ = 7.5 Hz), 7.38 (dd, $^3J_{HH} \approx {}^3J_{HH}$ = 7.5 Hz)](each 1H, C_6H_4), [1.87 (m, 1H), 1.51 (m, 3H), 1.32 (m, 2H), 1.16 (m, 2H), 0.62 (m, 1H)](cyclopentyl).

^{13}C{^{1}H} NMR (151 MHz, $CDCl_3$, 293 K): δ = [157.0 (*i*-C), 142.3 (CH), 141.7 (CH), 122.4 (CH), 118.0 (CH)](pyridyl), [153.5 (br, B-C), 136.4 (*i*-C), 131.2 (CH), 130.6 (CH), 127.4 (CH), 121.7 (CH)](C_6H_4), [33.5 (br), 28.7, 28.7, 27.0, 26.4](cyclopentyl).

^{11}B{^{1}H} NMR (193 MHz, $CDCl_3$, 293 K): δ = 4.6 ($\nu_{1/2} \approx$ 140 Hz).

^{11}B NMR (193 MHz, $CDCl_3$, 293 K): δ = 4.6 ($\nu_{1/2} \approx$ 150 Hz).

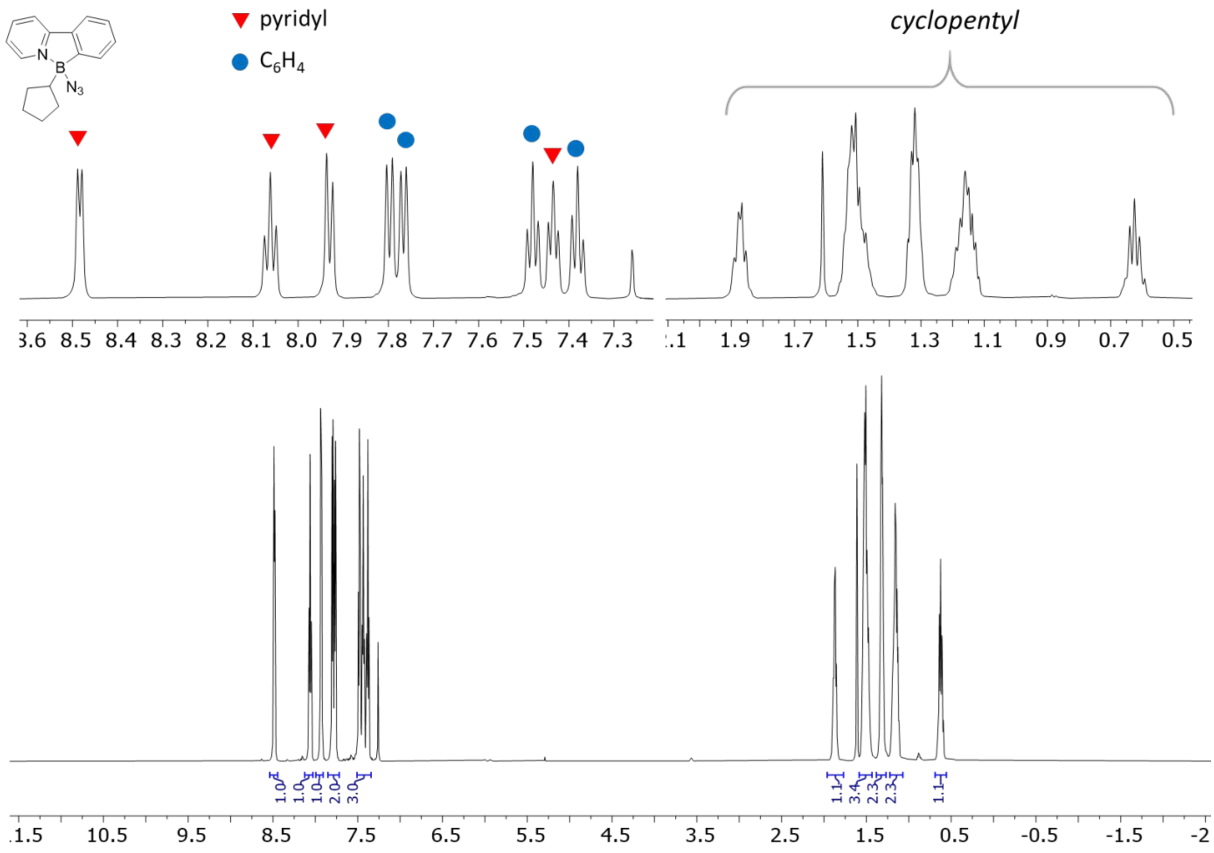

Figure S9: **^{1}H NMR** ($CDCl_3$, 600 MHz, 293 K) spectrum of compound **3c**.

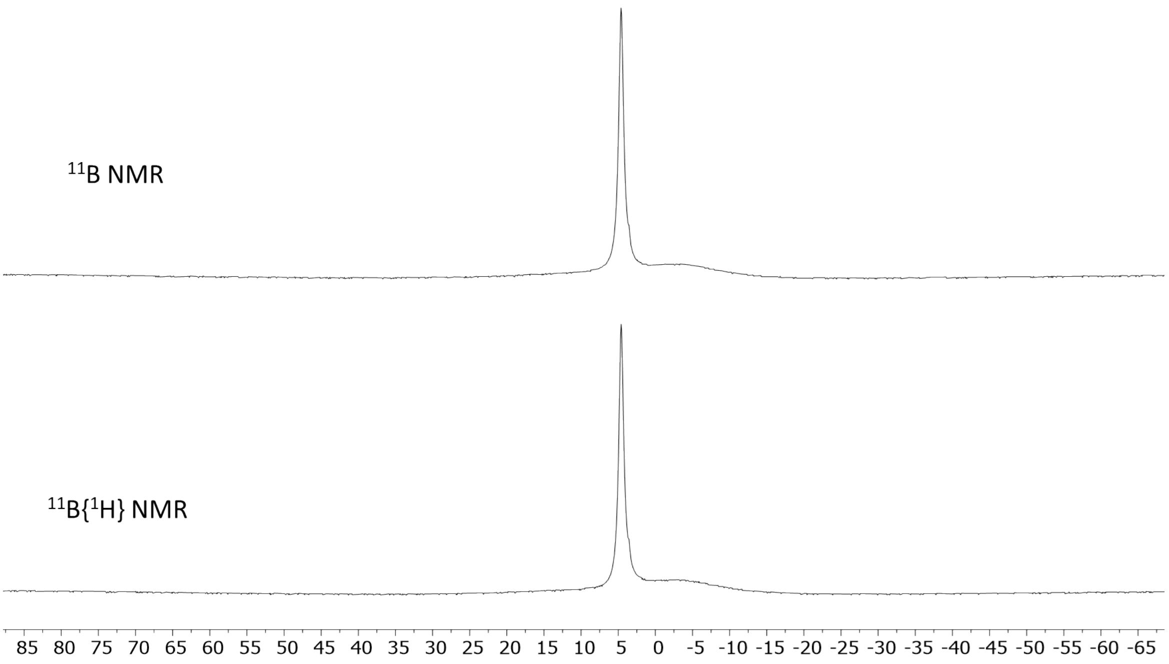

Figure S10: **^{11}B** and **^{11}B{^{1}H} NMR** (193 MHz, $CDCl_3$, 293 K) spectra of compound **3c**.

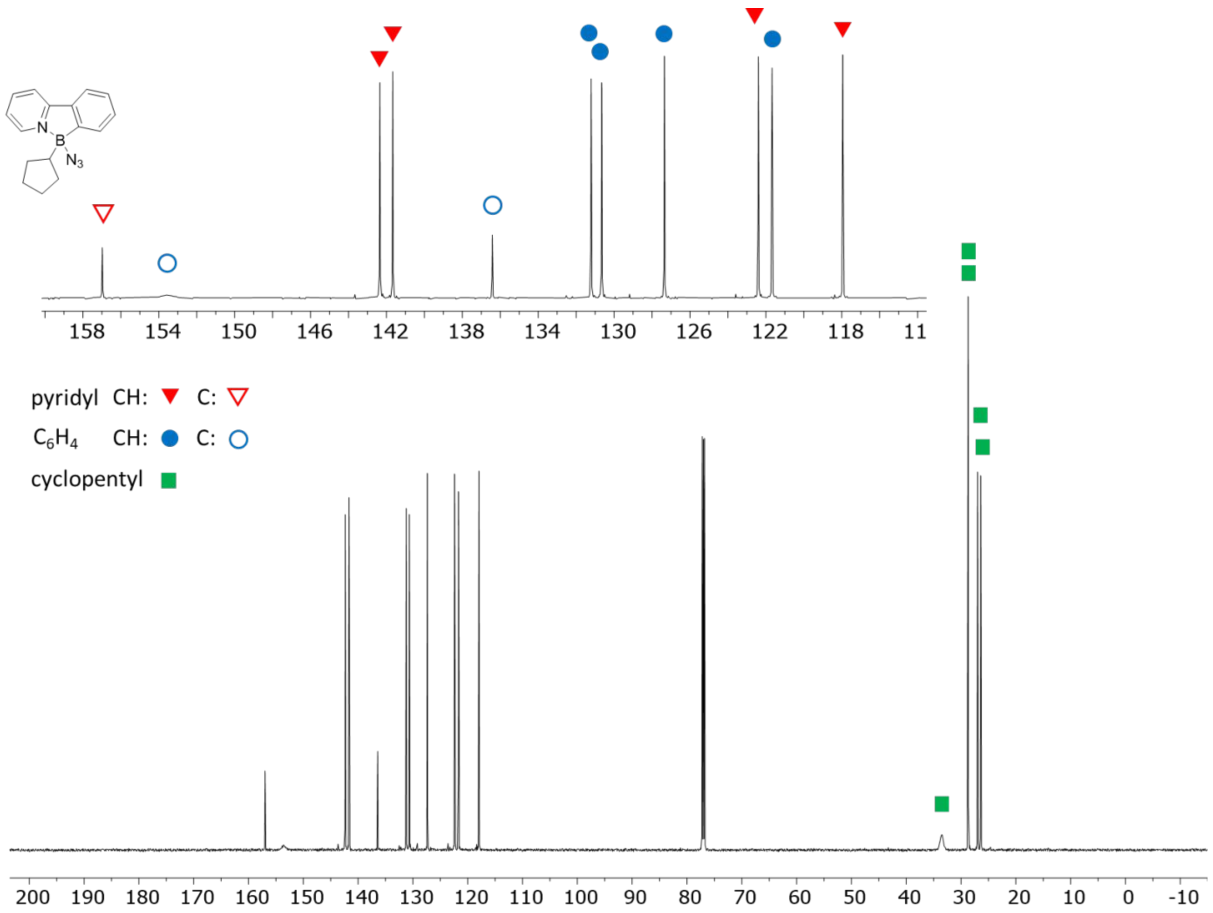

Figure S11: ^{13}**C{^{1}H} NMR** (151 MHz, $CDCl_3$, 293 K) spectrum of compound **3c**.

Crystal structure determination of 3c [KSK452-gg]

A crystal suitable for SC-XRD structure determination was obtained by slow diffusion of hexane into a solution of **3c** in dichloromethane under ambient conditions.

Crystal Data for $C_{16}H_{17}BN_4$ (M =276.14 g/mol): monoclinic, space group $P2_1/n$ (no. 14), a = 9.2863(2) Å, b = 13.9944(4) Å, c = 10.7641(3) Å, β = 92.895(2)°, V = 1397.09(6) Å^3, Z = 4, T = 100.00(10) K, μ(Cu Kα) = 0.625 mm^{-1}, $Dcalc$ = 1.313 g/cm^3, 17836 reflections measured (10.376° ≤ 2Θ ≤ 155.804°), 2882 unique (R_{int} = 0.0667, R_{sigma} = 0.0333) which were used in all calculations. The final R_1 was 0.0503 (I > 2σ(I)) and wR_2 was 0.1374 (all data). **CCDC: 2445685**

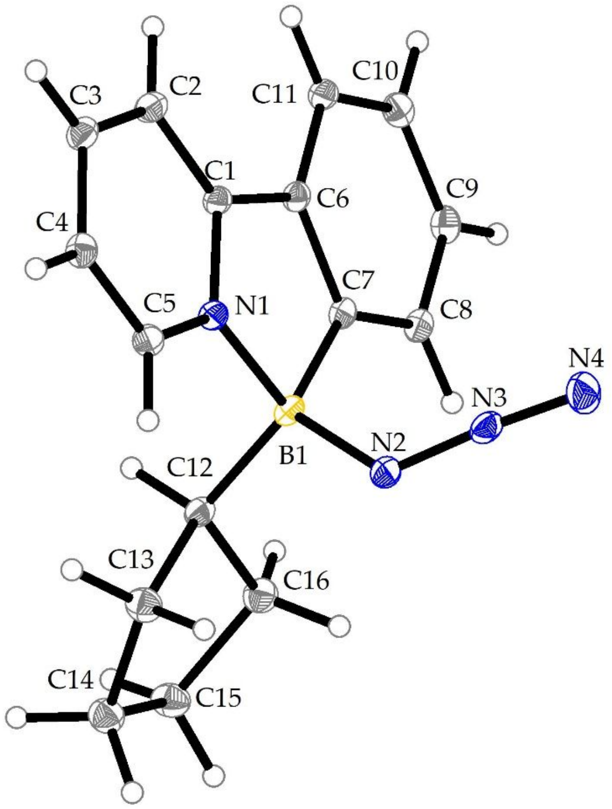

Figure S12: Crystal structure of **3c** (thermal ellipsoids shown at 30 % probability level).

Preparation of **4a**

Preparation using $TMSN_3$

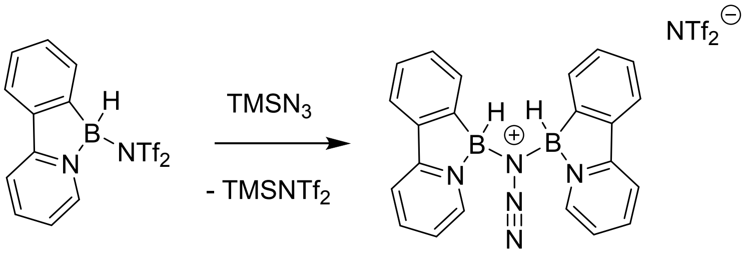

2a **4a**

Solution of **2a** (89.2 mg, 0.200 mmol) in 2 mL CH_2Cl_2 was treated with $TMSN_3$ (23.0 mg, 0.200 mmol) in 2 mL CH_2Cl_2. The mixture was stirred for 60 minutes at room temperature. Subsequently, volatiles were removed *in vacuo*. The oily residue was washed three times with CH_2Cl_2/Hx mixture (ca 5:1, 5 mL) to induce the solidification of the product. The solid residue was dried *in vacuo* to provide **4a** as a white solid (71.9 mg, 0.110 mmol, 55% yield).

Preparation by reaction of **2a** with **3a**

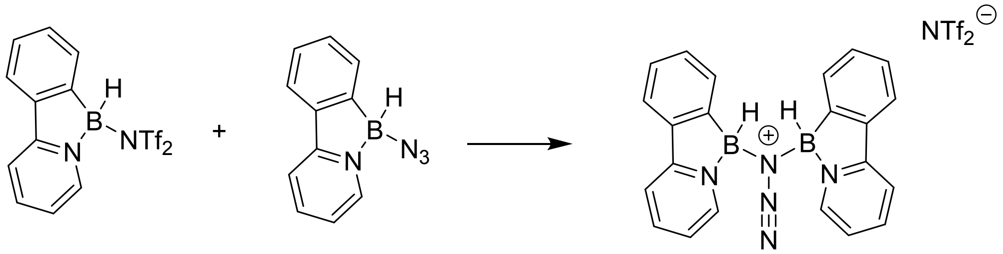

2a **3a** **4a**

Solution of **2a** (89.2 mg, 0.2 mmol) in 2 mL CH_2Cl_2 was added to solution of **3a** (41.6 mg, 0.2 mmol) in 1 mL CH_2Cl_2. The mixture was stirred for 30 minutes at room temperature, filtered and volatiles were removed *in vacuo*. The residue was washed with hexane and dried *in vacuo* to give **4a** as a white solid (90.5 mg, 0.138 mmol, 69 % yield).

Product consists of a mixture of diastereomers in ca 2:1 ratio (estimation based on ^{1}H NMR). Due to the gradual degradation of the compound in solution, satisfactory $^{13}C\{^1H\}$ spectra could not be obtained and 2D NMR experiments were not measured.

Elemental analysis calculated for $C_{24}H_{18}N_6B_2F_6O_4S_2$ (654.2): C 44.06, H 2.77, N 12.85; found C 43.81, H 2.86, N 12.78.

HRMS calculated for cation $C_{22}H_{18}B_2N_5^+$ 374.1743, found 374.1735 and anion $C_2NS_2O_4F_6^-$ calculated 279.9178 found 279.9170.

IR (KBr): 3132 (w), 3088 (w), 3066 (w), **2460 (m, B-H)**, **2446 (m, B-H)**, **2184 (vs, N_3)**, 1627 (s), 1574 (w), 1490 (s), 1451 (m), 1357 (s), 1334 (m), 1293 (w), **1190 (br s, NTf_2)**, 1132 (m), 1085 (w), 1057 (s), 1004 (m), 928 (w), 876 (w), 822 (w), 789 (w), 770 (m), 761 (m), 748 m), 735 (m), 668 (w), 612 (s), 588 (w), 569 (m), 514 (m) cm^{-1}.

^{1}H NMR (600 MHz, CD_2Cl_2, 293 K): *major isomer* δ = 8.67 (d, $^3J_{HH}$ = 5.7 Hz, 1H), 8.10 (dd, $^3J_{HH} \approx {}^3J_{HH} \approx$7.8 Hz, 1H), 7.61 (dd, $^3J_{HH}$ = 7.8 Hz, $^3J_{HH}$ = 5.7 Hz, 1H), 7.56 (d, $^3J_{HH}$ = 8.1 Hz, 1H), 7.37 (d, $^3J_{HH}$ = 7.7 Hz, 1H), 7.19 (dd, $^3J_{HH} \approx {}^3J_{HH} \approx$ 7.6 Hz, 1H), 7.00 (dd, $^3J_{HH} \approx {}^3J_{HH} \approx$ 7.2 Hz, 1H), 6.94 (d,

$^3J_{HH}$ = 7.2 Hz, 1H), 3.86 (br m, 1H, BH) ; *minor isomer* δ = 8.38 (d, $^3J_{HH}$ = 5.7 Hz, 1H), 8.17 (dd, $^3J_{HH}$ ≈ $^3J_{HH}$ ≈ 7.8 Hz, 1H), 7.85 (d, $^3J_{HH}$ = 8.0 Hz, 1H), 7.65 (d, $^3J_{HH}$ = 7.0 Hz, 1H), 7.50 (dd, $^3J_{HH}$ ≈ $^3J_{HH}$ ≈ 7.5 Hz, 1H), 7.46 (m, 1H), 7.43-7.39 (m, 2H), 3.86 (br m, 1H, BH).

^{11}B{^{1}H} NMR (193 MHz, CD_2Cl_2, 293 K): *major and minor isomer* δ = 0.7 ($\nu_{1/2}$ ≈ 250 Hz).

^{11}B NMR (193 MHz, CD_2Cl_2, 293 K): *major and minor isomer* δ = 0.7 ($\nu_{1/2}$ ≈ 330 Hz).

^{19}F NMR (564 MHz, CD_2Cl_2, 293 K): δ = –79.3 (s).

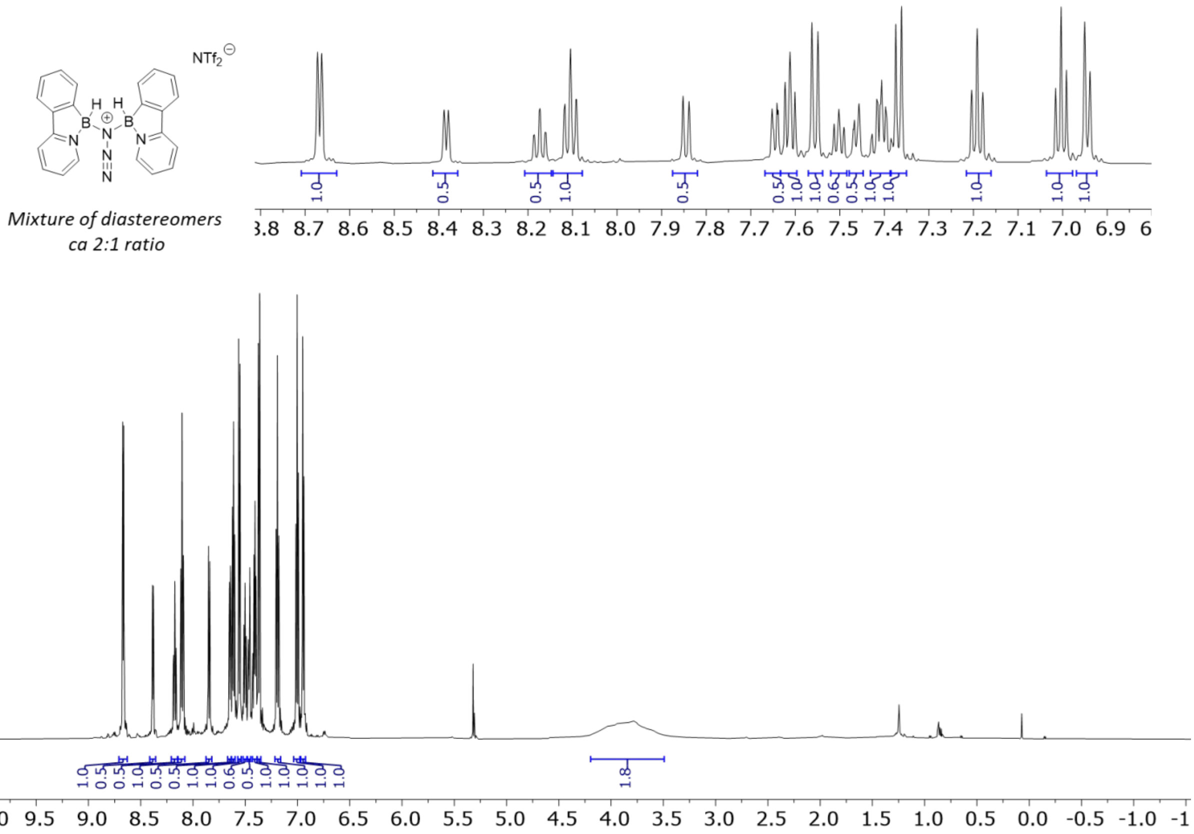

Figure S13: **^{1}H NMR** (CD_2Cl_2, 600 MHz, 293 K) spectrum of compound **4a**.

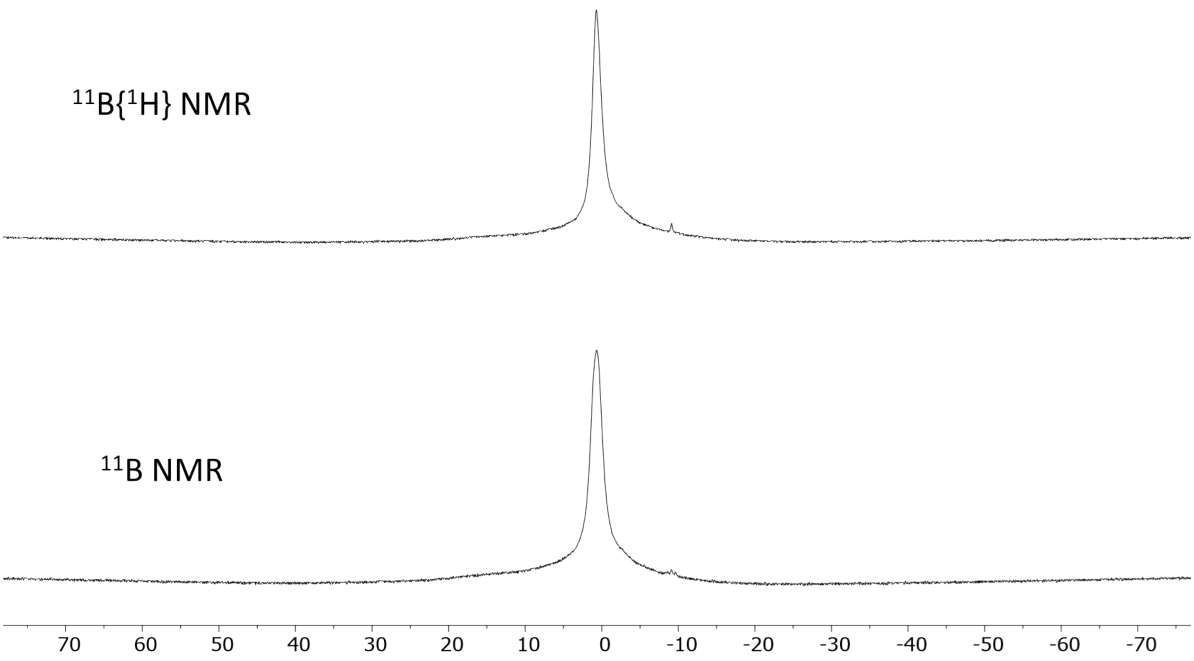

Figure S14: **^{11}B** and **^{11}B{^{1}H} NMR** (193 MHz, CD_2Cl_2, 293 K) spectra of compound **4a**.

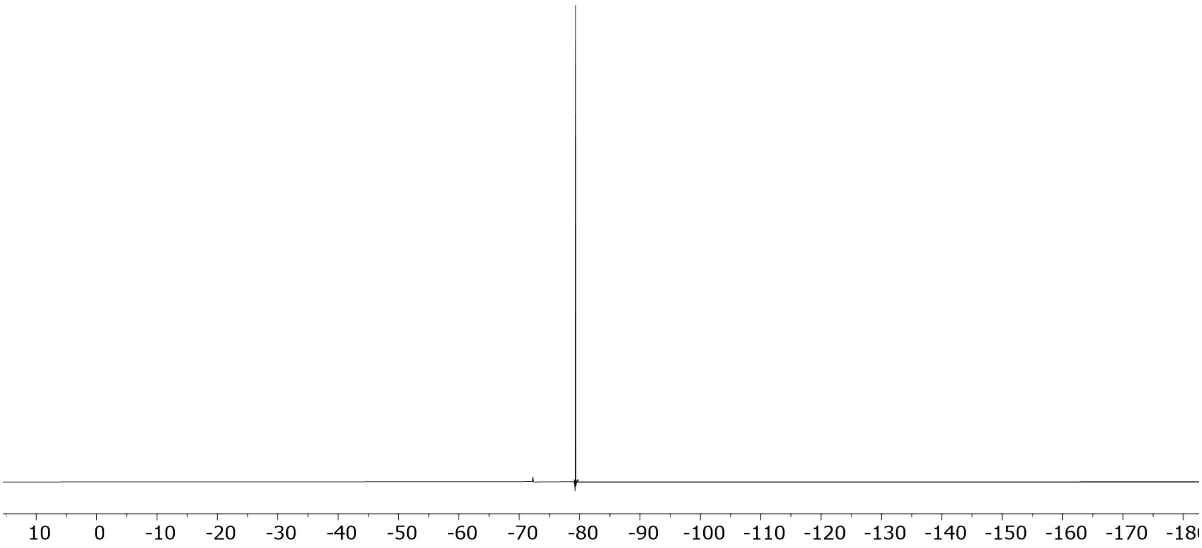

Figure S15: **^{19}F NMR** (564 MHz, CD_2Cl_2, 293 K) spectrum of compound **4a**.

Crystal structure determination of **4a [KSK185_40_Cu_100-aa]**

A crystal suitable for SC-XRD structure determination was obtained by slow diffusion of hexane into a solution of **4a** in dichloromethane in argon filled glovebox at –35 °C.

Crystal Data for $C_{24}H_{18}B_2F_6N_6O_4S_2$ (M =654.18 g/mol): triclinic, space group P1 (no. 1), a = 6.9650(2) Å, b = 10.3043(3) Å, c = 10.5014(3) Å, α = 78.684(2)°, β = 71.706(2)°, γ = 85.035(2)°, V = 701.42(4) Å^3, Z = 1, T = 100.00(10) K, μ(Cu Kα) = 2.486 mm^{-1}, $Dcalc$ = 1.549 g/cm^3, 16522 reflections measured (8.754° ≤ 2Θ ≤ 154.748°), 5198 unique (R_{int} = 0.0512, R_{sigma} = 0.0433) which were used in all calculations. The final R_1 was 0.0612 (I > 2σ(I)) and wR_2 was 0.1997 (all data). **CCDC: 2445687**

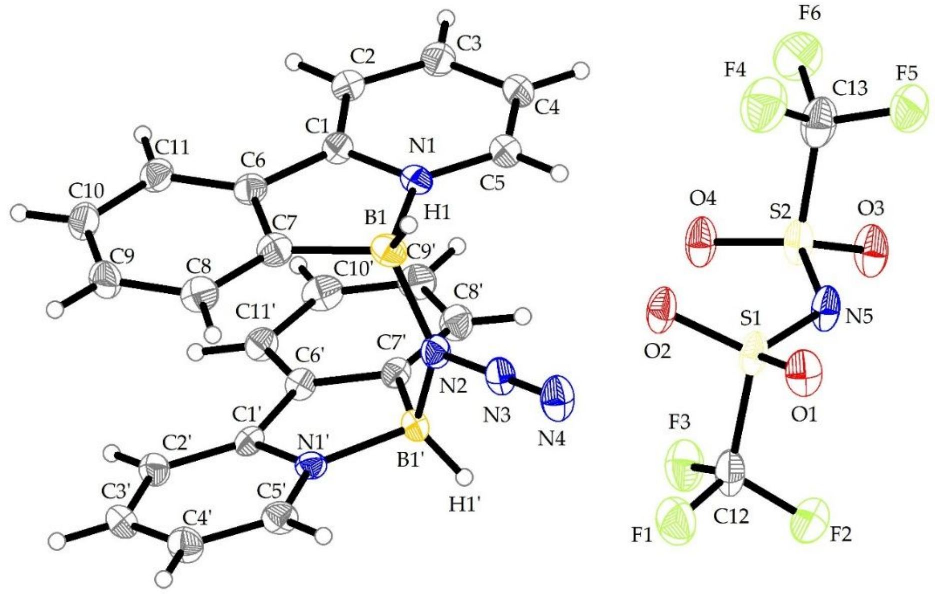

Figure S16: Crystal structure of **4a** (thermal ellipsoids shown at 30 % probability level).

Thermal and photochemical stability of boron azides

Thermolytic stability in solution

Approximately 10 mg of compound **3a**, **3b** or **3c** was dissolved in 0.6 mL of dry deareated mesitylene, transferred into a Young NMR tube, and placed in a preheated oil bath. The stability of the azides was monitored by $^{11}B\{^{1}H\}$ NMR spectroscopy (without lock or shim). Upon heating compound **3a** and **3b** at 140 °C for 20 hours, the solutions turned yellow, and copious beige precipitate formed. NMR analysis indicated a substantial decrease in the signal corresponding to compound **3a** (δ_B = -0.3) or 3b (δ_B = 4.4) giving rise to at least three different boron signals. In contrast, when compound **3c** was heated for 5 days at 160 °C, the sample remained clear, and NMR analysis showed only minimal decomposition of azide **3c** (δ_B = 4.5). See spectra below.

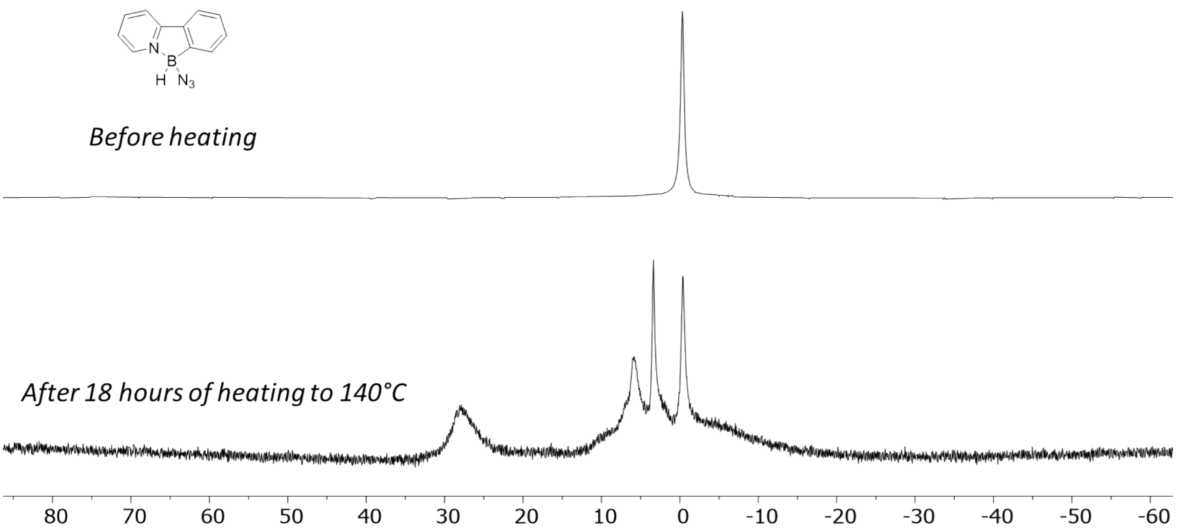

Figure S17: $^{11}B\{^{1}H\}$ NMR (192 MHz, mesitylene, 293K) spectra of sample **3a** before and after heating for 18h to 140°C showing non-selective decomposition **3a**.

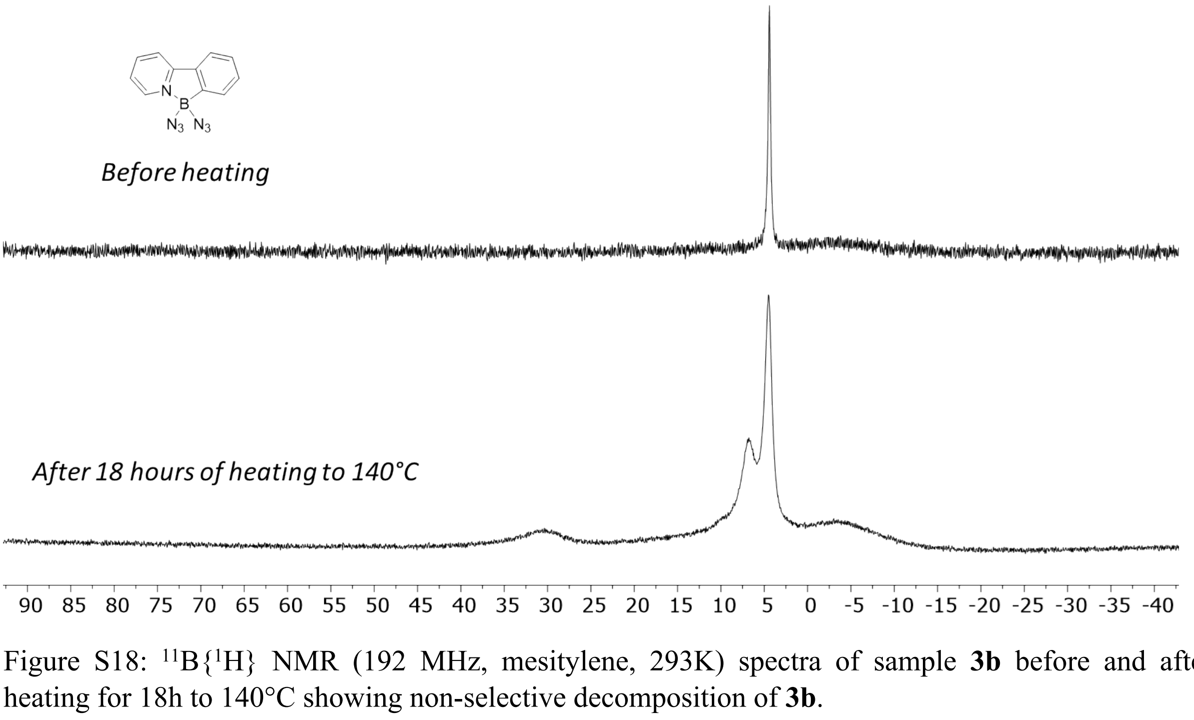

Figure S18: $^{11}B\{^{1}H\}$ NMR (192 MHz, mesitylene, 293K) spectra of sample **3b** before and after heating for 18h to 140°C showing non-selective decomposition of **3b**.

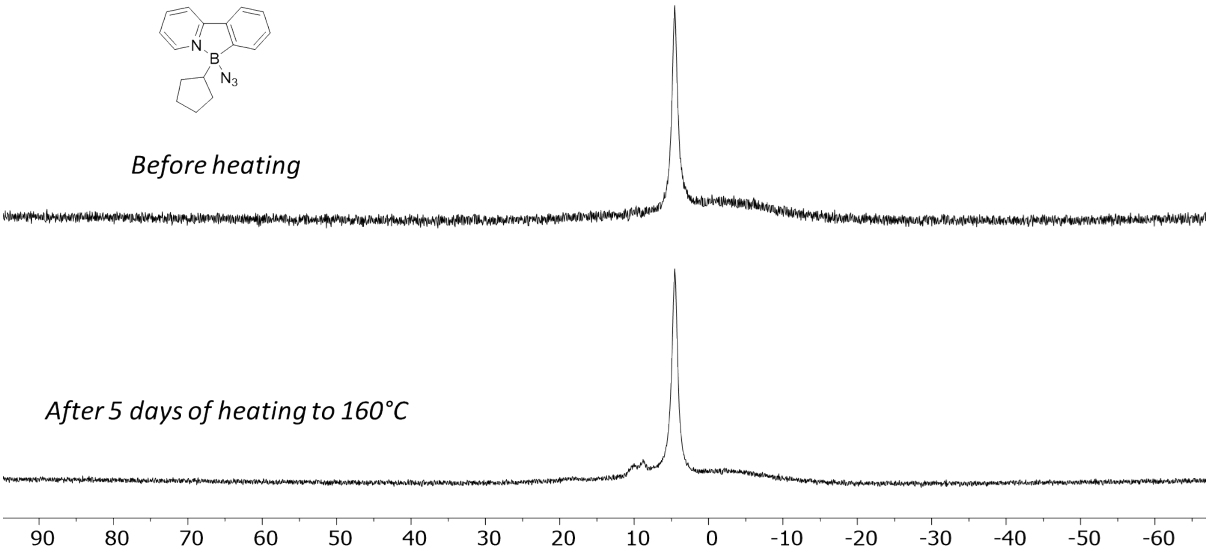

Figure S19: $^{11}B\{^{1}H\}$ NMR (192 MHz, mesitylene, 293K) spectra of sample **3c** before and after heating to 160°C for 5 days indicating thermal robustness of **3c**.

Thermolytic stability in solid state

Approximately 1.5 mg of the corresponding crystalline substance was heated in a nitrogen flow at a rate of 5 °C/min, using a simultaneous thermal analysis (STA) instrument and the substance's heat flow and mass loss were monitored. During the DSC measurement, endothermic events corresponding to the melting point were first observed (for compound **3a** at 100 °C, **3b** at 82 °C, and **3c** at 149 °C), followed by exothermic events corresponding to its decomposition. Only in the case of compound **3b**, the mass loss revealed by thermogravimetric analysis (TGA) roughly corresponded to the loss of two nitrogen molecules (see below).

Attempted thermolysis of **3c** by placing solid samples in an Argon-filled Schlenk flask for 3 hours in an oil bath preheated to 170 °C resulted only in melting of the sample, with no reaction occurring. Placing the flask in an oil bath preheated to 180 °C resulted in gradual darkening and gas evolution. No defined product was identified by NMR analysis of the thermolyzed sample.

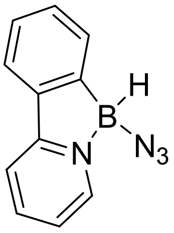

3a

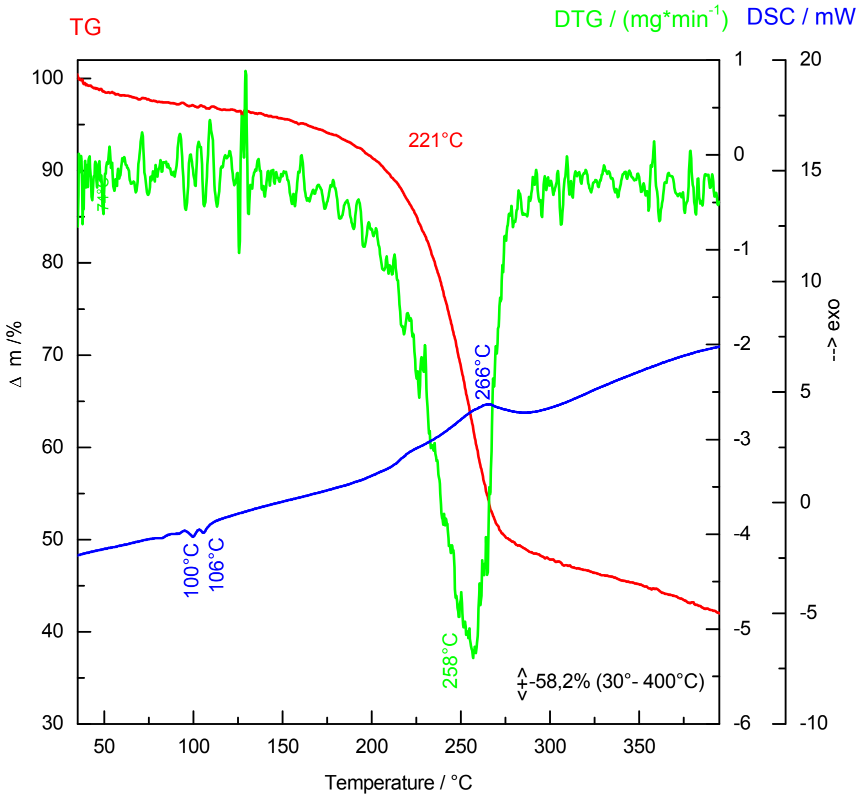

Figure S20: Simultaneous thermogravimetric and differential scanning calorimetry analysis (TGA/DSC) of compound **3a**.

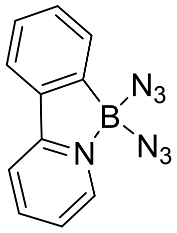

3b

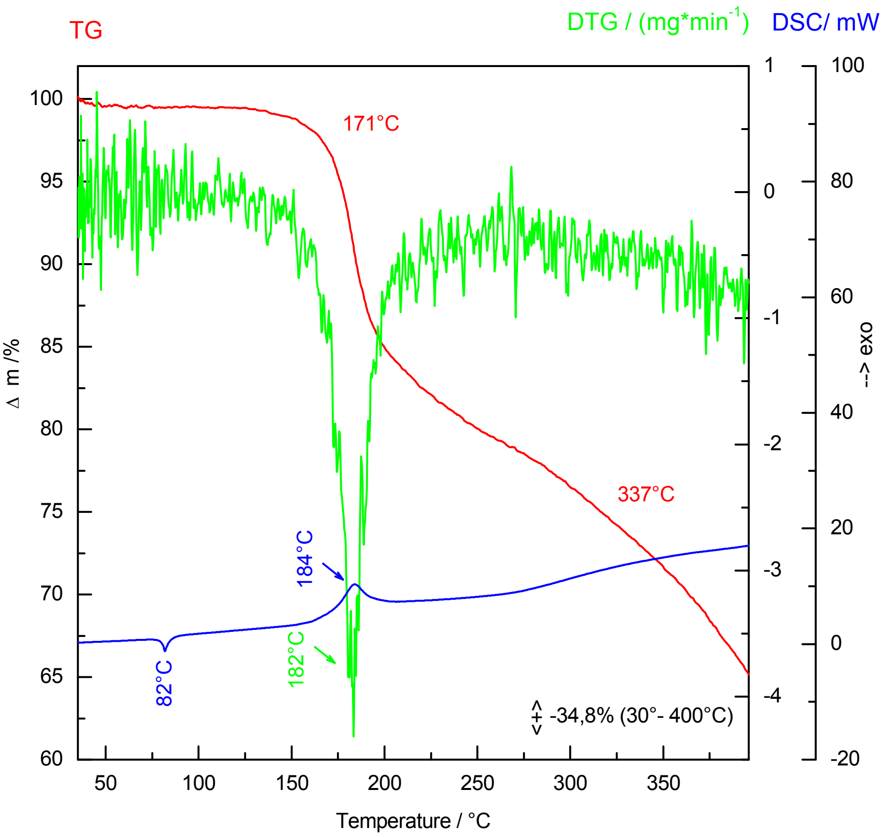

Figure S21: Simultaneous thermal analysis TGA/DSC of compound **3b**.

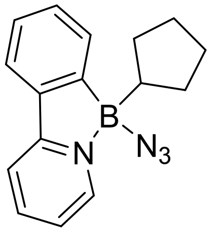

3c

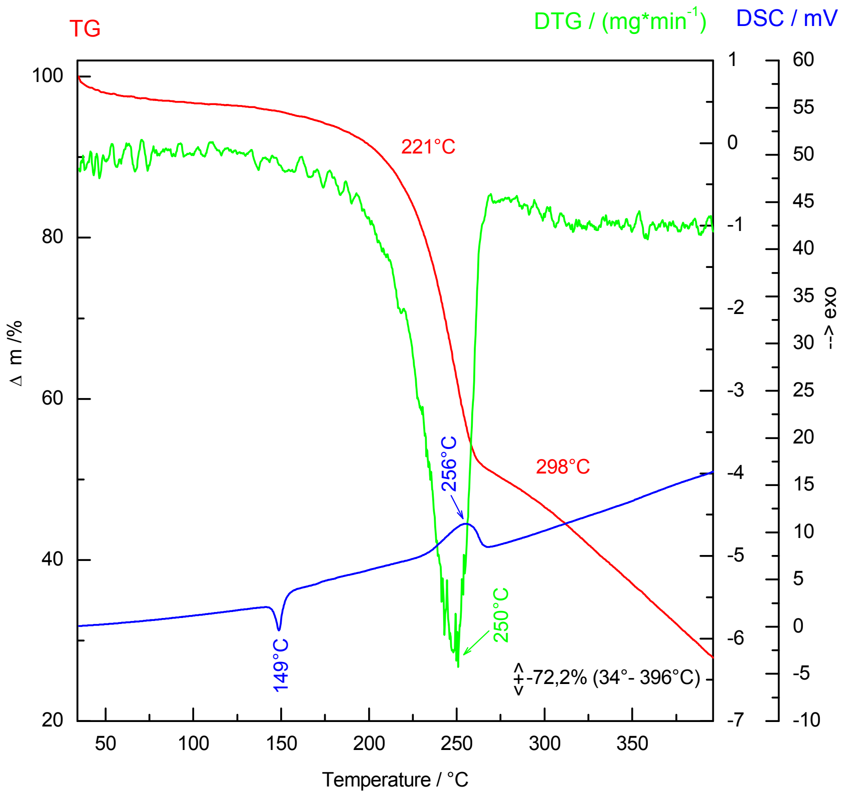

Figure S22: Simultaneous thermal analysis TGA/DSC of compound **3c**.

Photostability of **3a**

Representative experiment: 10 mg of **3a** was dissolved in 0.7 mL of C_6D_6 and placed into quartz NMR tube. NMR spectrum was collected and subsequently, the solution was continuously irradiated using an arc lamp source (Oriel, Newport) equipped with a 300 W ozone-free Xe lamp (Newport) for 6 h. The initially colourless sample turned pale yellow, but NMR measurement did not reveal any significant conversion of starting material (see spectra below).

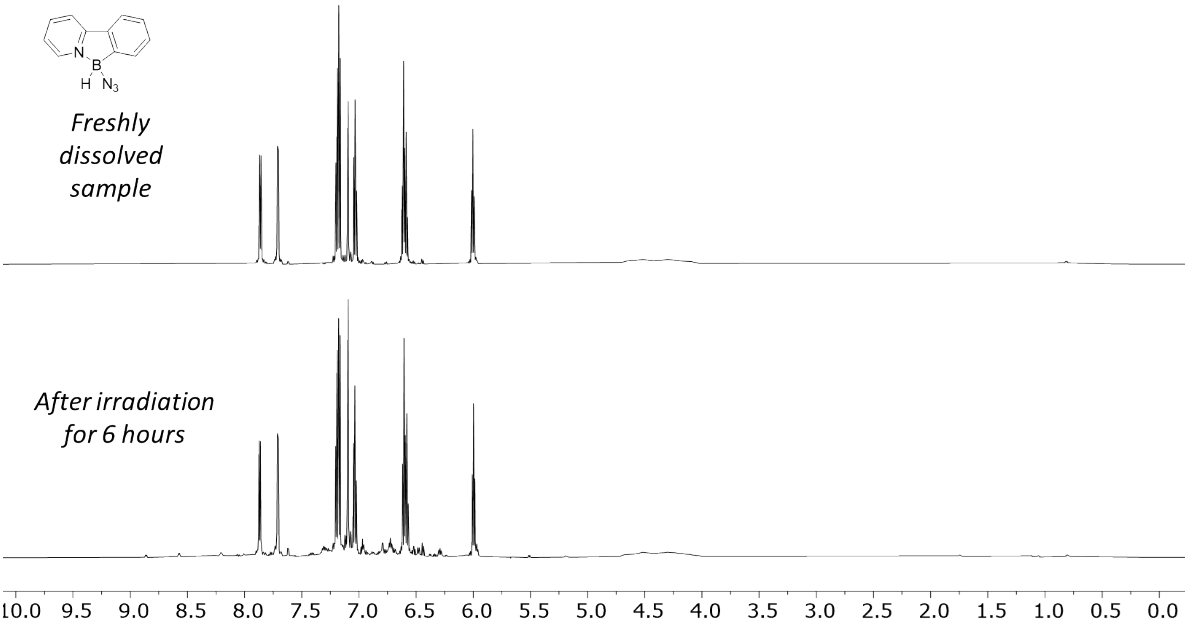

Figure S23: Comparison of **^{1}H NMR** (C_6D_6, 600 MHz, 293 K) spectra of **3a** before and after irradiation with enone lamp for 6 hours in quartz NMR tube.

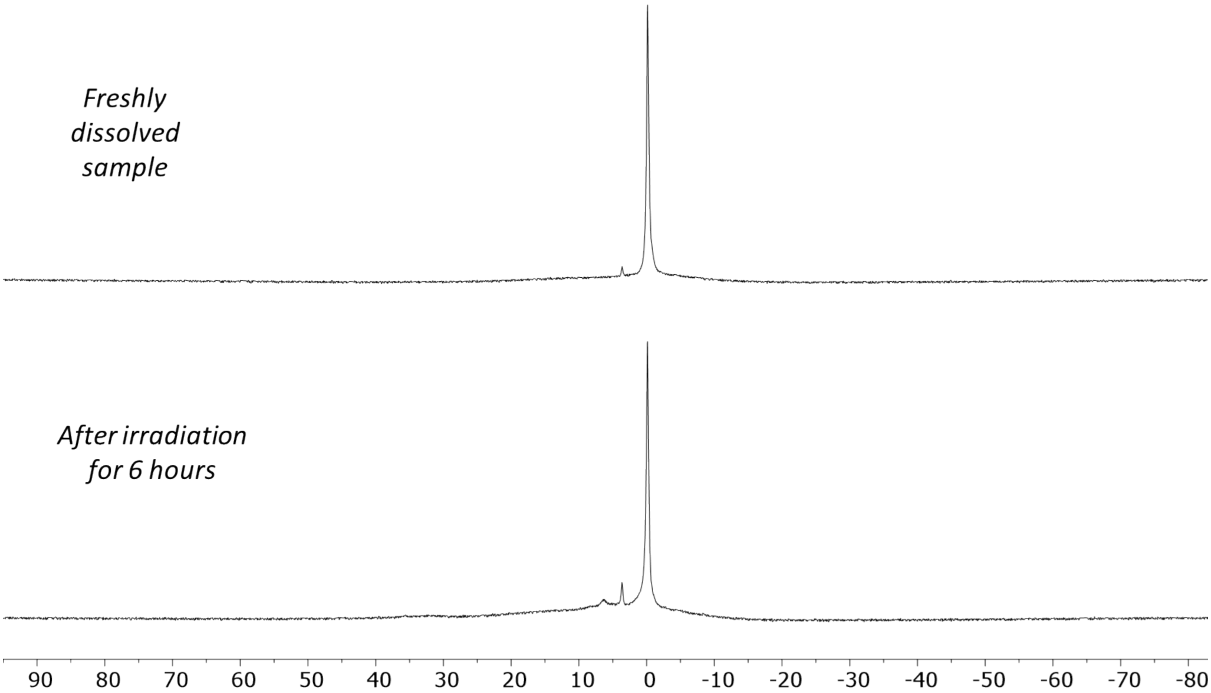

Figure S24: Comparison of **^{11}B{^{1}H} NMR** (C_6D_6, 193 MHz, 293 K) spectra of **3a** before and after irradiation with xenone lamp for 6 hours in quartz NMR tube.

Reactivity of prepared azides towards phosphine nucleophiles, preparation of **6c** and **7c**

In a typical experiment setup, 0.05 mmol of boron azide **3c** was treated with 0.05 mmol of corresponding phosphine (triphenylphosphine, tri-*n*-butylphosphine or triethyl phosphite) in 0.7 ml of deuterated toluene. The mixture was placed in an oil bath preheated to 100 °C and the reaction progress was monitored by ^{1}H and ^{31}P{^{1}H} NMR. After the designated period of time, an aliquot was taken from the reaction mixture and analyzed by HRMS.

Example 1

Reaction of **3c** with PPh_3

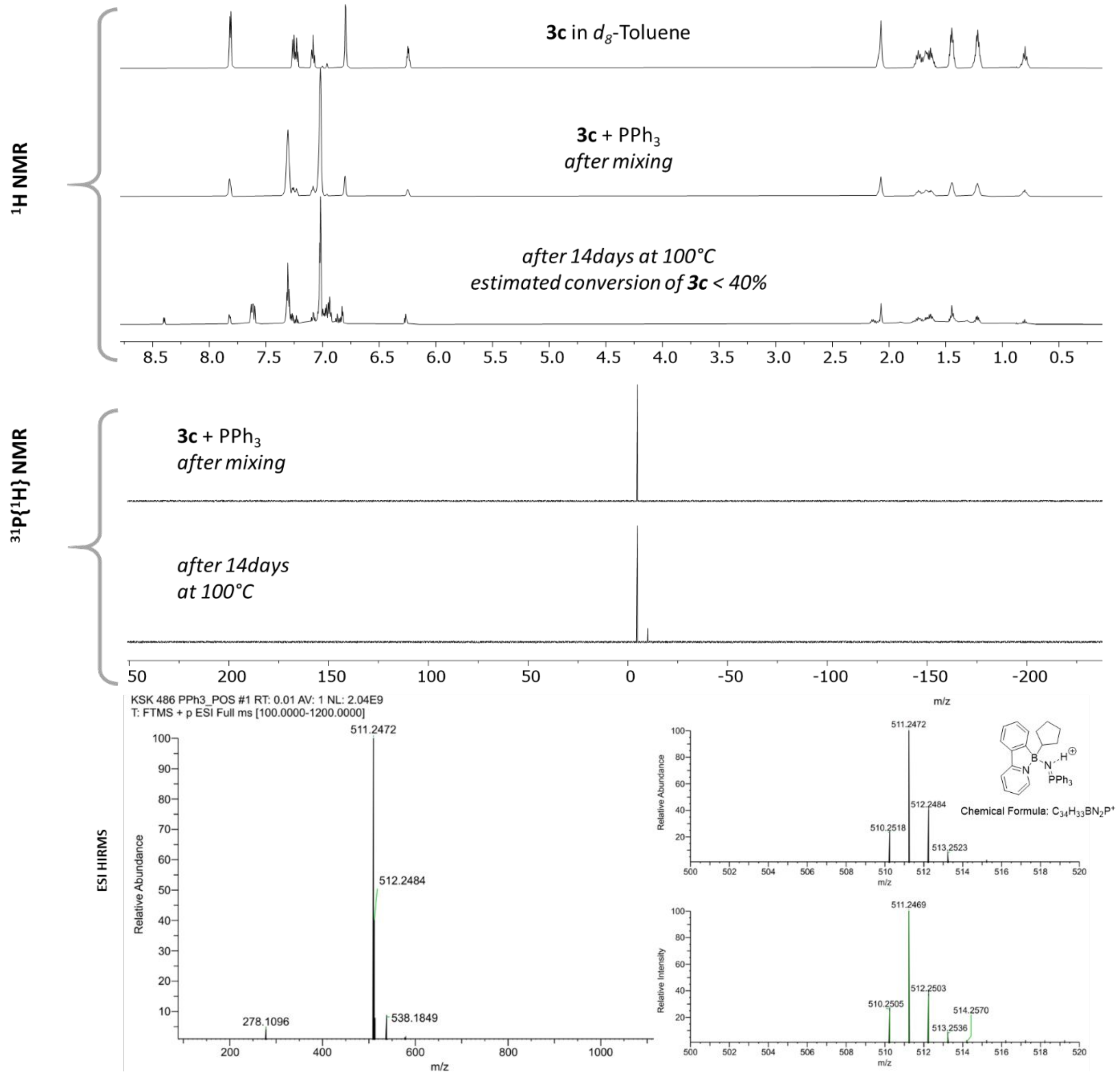

Figure S25: **^{1}H NMR** (Tol-d_8, 600 MHz, 293 K) spectra monitoring the progress of the reaction of **3c** with triphenylphosphine and HRMS analysis of the reaction mixture after the designated reaction time.

Example 2

Reaction of **3c** with *n*Bu_3P

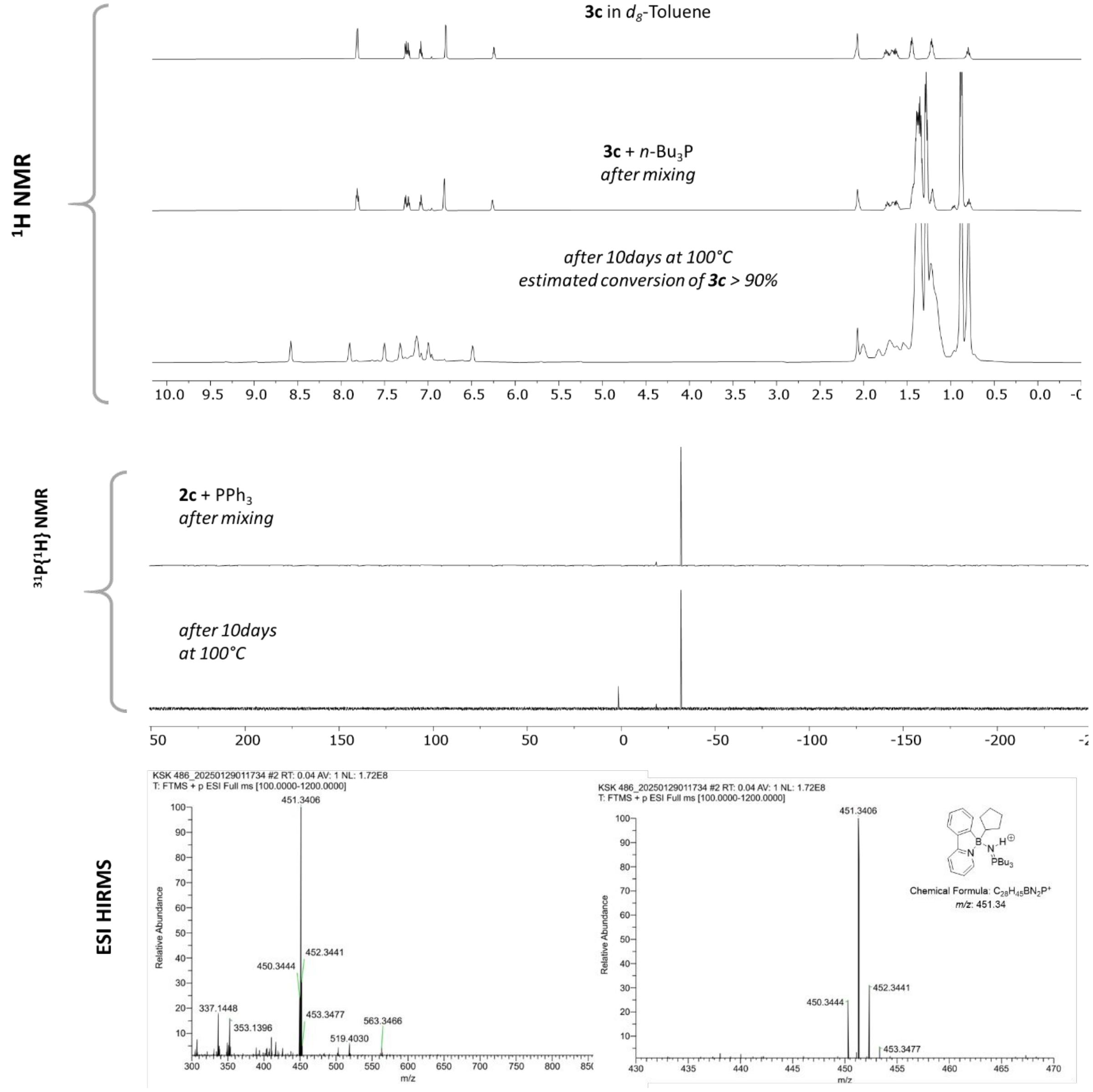

Figure S26: **¹H NMR** (Tol-d_8, 600 MHz, 293 K) spectra monitoring the progress of the reaction of **3c** with tri-*n*-butylphosphine and HRMS analysis of the reaction mixture after the designated reaction time.

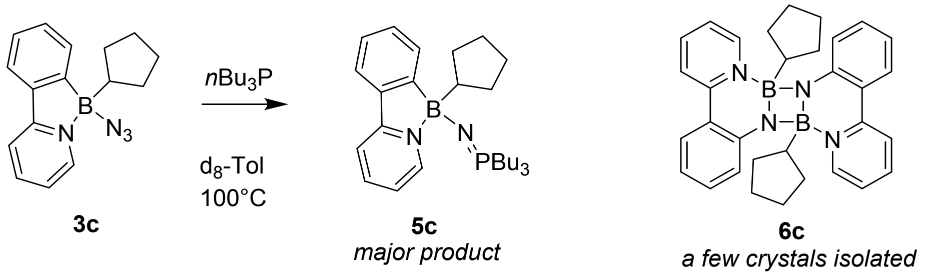

Boron azide **3c** (27.6 mg, 0.100 mmol) and tri-*n*-butylphosphine (10.1 mg, 0.100 mmol) were suspended in d8-toluene (0.7 mL) in Young NMR tube and placed to an oil bath preheated to 100 °C. The mixture was slowly turning brown and the reaction progress was periodically monitored by NMR. After 7 days, complete conversion of starting material was observed together with the formation of several red crystals in the deep brown solution. These were separated by flotation, washed with toluene, hexane and dried *in vacuo* to give compound **6c** as a red solid (3 mg, 0.006 mmol, 12% yield).

Comparison of the NMR spectra of the isolated compound **6c** and the crude reaction mixture (see below) confirmed that compound **6c** is a minor product of the reaction. Presence of compound **5c** in the mother liquor was further supported by HRMS analysis.

Characterization data of **6c**

HRMS for $C_{32}H_{35}N_4B_2^+$ $[M+H]^+$: calculated 497.3048, found 497.3042.

^{1}H NMR (600 MHz, $CDCl_3$, 293 K): δ = [8.74 (d, $^3J_{HH}$ = 5.8 Hz), 7.87 (m), 7.86 (m), 7.67 (dd, $^3J_{HH}$ = 8.1 Hz, $^4J_{HH}$ = 1.6 Hz), 7.41 (ddd, $^3J_{HH} \approx {}^3J_{HH} \approx$ 6.9 Hz, $^4J_{HH}$ = 1.7 Hz), 7.20 (ddd, $^3J_{HH} \approx {}^3J_{HH}$ = 7.7 Hz, $^4J_{HH}$ = 1.5 Hz), 7.00 (dd, $^3J_{HH}$ = 8.2 Hz, $^4J_{HH}$ = 1.4 Hz), 6.52 (ddd, $^3J_{HH} \approx {}^3J_{HH} \approx$ 8.0 Hz, $^4J_{HH}$ = 1.3 Hz)](each 1H, $C_6H_4C_5NH_4$), [1.35-0.68](m, 9H, C_5H_9).

^{11}B{^{1}H} NMR (193 MHz, $CDCl_3$, 293 K): δ = 6.4 ($\nu_{1/2} \approx$ 200 Hz).

^{11}B NMR (193 MHz, $CDCl_3$, 293 K): δ = 6.4 ($\nu_{1/2} \approx$ 200 Hz).

Due to limited solubility of compound **6c**, satisfactory $^{13}C\{^1H\}$ spectra could not be recorded and no 2D NMR experiments were measured.

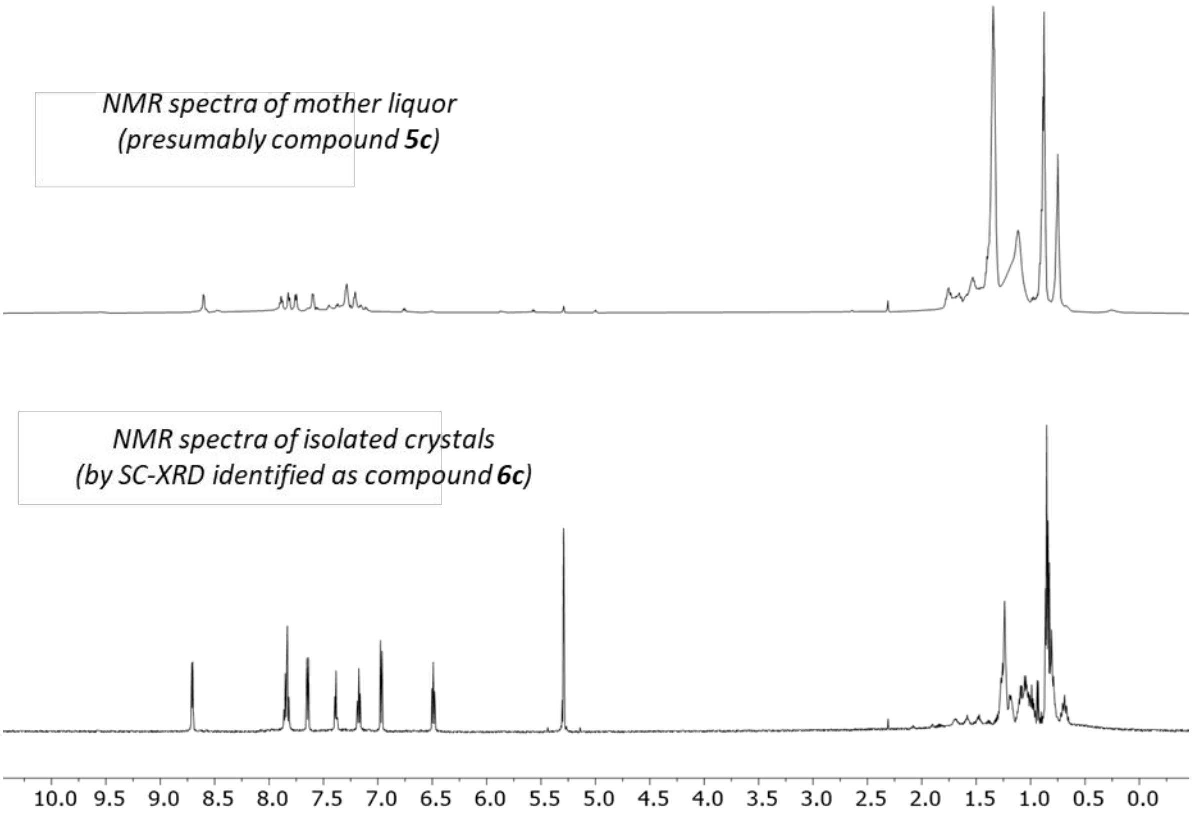

Figure S27: Comparison of **^{1}H NMR** (CD_2Cl_2, 600 MHz, 293 K) spectra of isolated compound **6c** and the mother liquor (presumably compound **5c**).

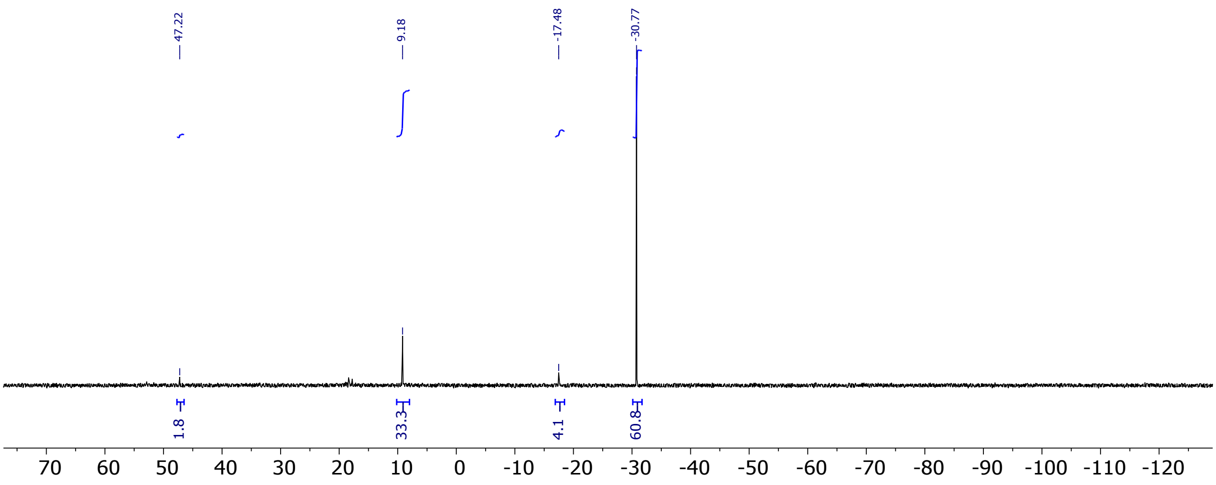

Figure S28: **^{31}P{^{1}H} NMR** (CD_2Cl_2, 243 MHz, 293 K) spectrum of the mother liquor.

Crystal structure determination of **6c** [KSK486-2-gg]

A crystal of **6c** suitable for SC-XRD structure determination was obtained directly during cooling of the reaction mixture (toluene) from 100 °C to room temperature.

Crystal Data for $C_{16}H_{17}BN_2$ (M =248.12 g/mol): triclinic, space group P-1 (no. 2), a = 8.1246(7) Å, b = 8.4458(7) Å, c = 9.5610(8) Å, α = 73.613(8)°, β = 84.074(7)°, γ = 86.031(7)°, V = 625.50(10) Å^3, Z = 2, T = 100.00(10) K, μ(Cu Kα) = 0.587 mm^{-1}, $Dcalc$ = 1.317 g/cm^3, 6352 reflections measured (9.68° ≤ 2Θ ≤ 152.776°), 2461 unique (R_{int} = 0.0350, R_{sigma} = 0.0400) which were used in all calculations. The final R_1 was 0.0436 (I > 2σ(I)) and wR_2 was 0.1234 (all data). **CCDC: 2445680**

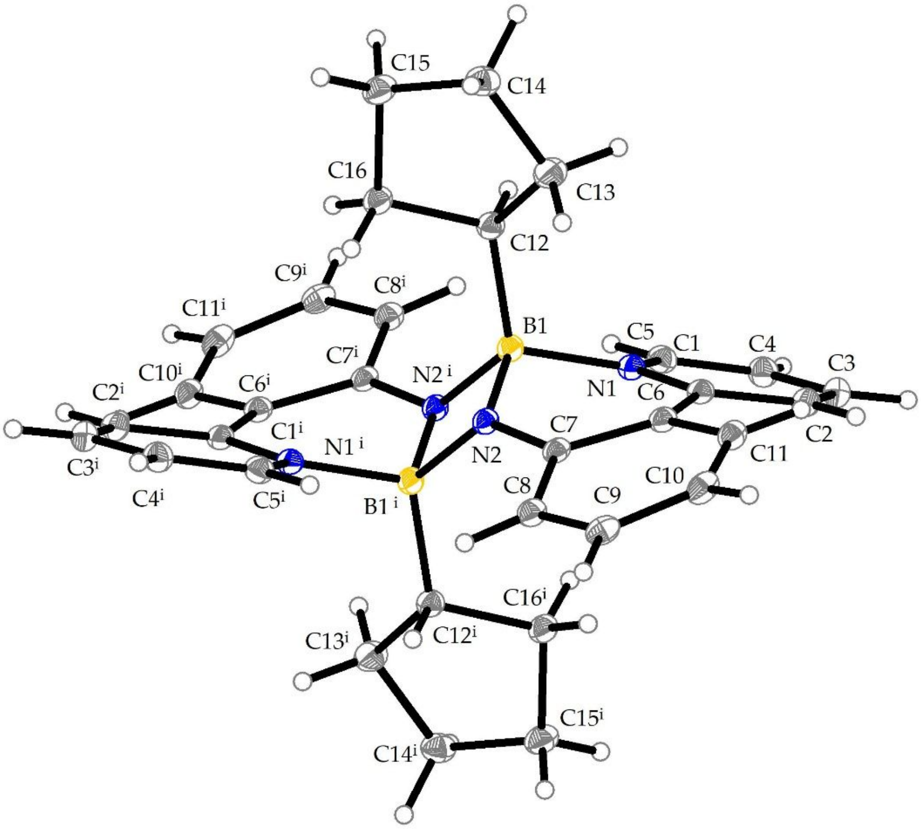

Figure S29: Crystal structure of **6c** (thermal ellipsoids shown at 30 % probability level).

Symmetry codes: i = 1 - x, - y, 2 - z.

Preparation of **7c**

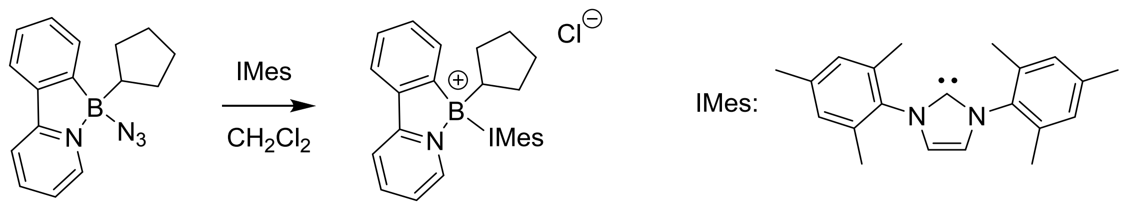

Boron azide **3c** (27.6 mg, 0.100 mmol) and IMes (1,3-dimesitylimidazol-2-ylidene, 30.4 mg, 0.100 mg) were combined in dichloromethane (3 mL) and allowed to react for 30 minutes at room temperature. Solvent was removed *in vacuo* and the oily residue was taken in dichloromethane (ca 1 mL), filtered and layered with hexane (ca 5 mL). After a week, the formed crystalline solid was carefully decanted, washed with hexane (2x2 mL) and dried *in vacuo* to give compound **7c** as a white solid (35.0 mg).

According to NMR, the product contains small amount of $IMesH^+$ cation (integration of ^1H NMR signals estimates molar ratio of cationic moiety $\mathbf{7c}^+$ and $IMesH^+$ cca 88:12). According to SC-XRD, the counter anion was identified as chloride, presumably originating from the solvent. The yield was not determined. Attempted reaction in benzene resulted in an immediate darkening and overall decomposition of the reaction mixture.

HRMS for $C_{37}H_{41}N_3B^+$ $[M]^+$: calculated 538.3394 found 538.3391.

IR (KBr) ν: 3161 (m), 2998 (m), 2929 (m), 2857 (m), 1620 (s), 1653 (m), 1484 (s), 1444 (s), 1408 (m), 1378 (m), 1331 (w), 1290 (w), 1258 (w), 1233 (m), 1222 (m), 1162 (m), 1135 (m), 1127 (m), 1071 (m), 1033 (m), 982 (w), 961 (w), 926 (w), 853 (m), 819 (w), 804 (m), 770 (s), 747 (s), 717 (w), 701 (w), 669 (w), 633 (w), 592 (m), 579 (m), 565 (w), 426 (w) cm^{-1}.

^{1}H NMR (600 MHz, $CDCl_3$, 293 K): δ = [8.22 (d, $^3J_{HH}$ = 5.9 Hz), 8.04 (dd, $^3J_{HH} \approx {}^3J_{HH}$ = 7.8 Hz), 7.61 (d, $^3J_{HH}$ = 8.2 Hz), 7.50 (m)](each 1H, pyridyl), [7.49, 7.48, 7.36, 7.35](each m, 1H, C_6H_4), [7.17 (s, 2H, CH^{Im}), 6.77 (m, 4H, CH^{Mes}), 2.29 (s, 3H, CH_3^{Mes}), 1.91 (s, 3H, CH_3^{Mes}), 1.81 (s, 3H, CH_3^{Mes})](IMes), [1.11, 0.95, 0.86, 0.82, 0.79, 0.79, 0.44, 0.41, -0.38](each m, 1H, C_5H_9).

^{13}C{^{1}H} NMR (151 MHz, $CDCl_3$, 293 K): δ = [158.4 (br, *i*-C)[a], 143.4 (CH), 142.6 (CH), 123.3 (CH), 118.9 (CH)](pyridyl), [153.2 (br, C-B)[a], 138.0 (*i*-C), 131.4 (CH), 131.2 (CH), 128.8 (CH), 122.4 (CH)](C_6H_4), [159.1 ($C^{carbene}$)[a], 141.2 (*i*-C), 135.2 (*i*-C), 134.7 (*i*-C), 129.7 (2xCH^{Mes}), 126.1 (CH^{Im}), 21.0 (CH_3), 18.5 (CH_3), 18.1 (CH_3)](IMes), [29.9 (CH_2), 29.0 (CH_2), 28.8 (br, B-CH), 27.3 (CH_2), 25.7 (CH_2)] (cyclopentyl), [a]based on gHMBC.

^{11}B{^{1}H} NMR (193 MHz, $CDCl_3$, 293 K): δ = –1.9 ($\nu_{1/2} \approx$ 250 Hz).

^{11}B NMR (193 MHz, $CDCl_3$, 293 K): δ = –1.9 ($\nu_{1/2} \approx$ 270 Hz).

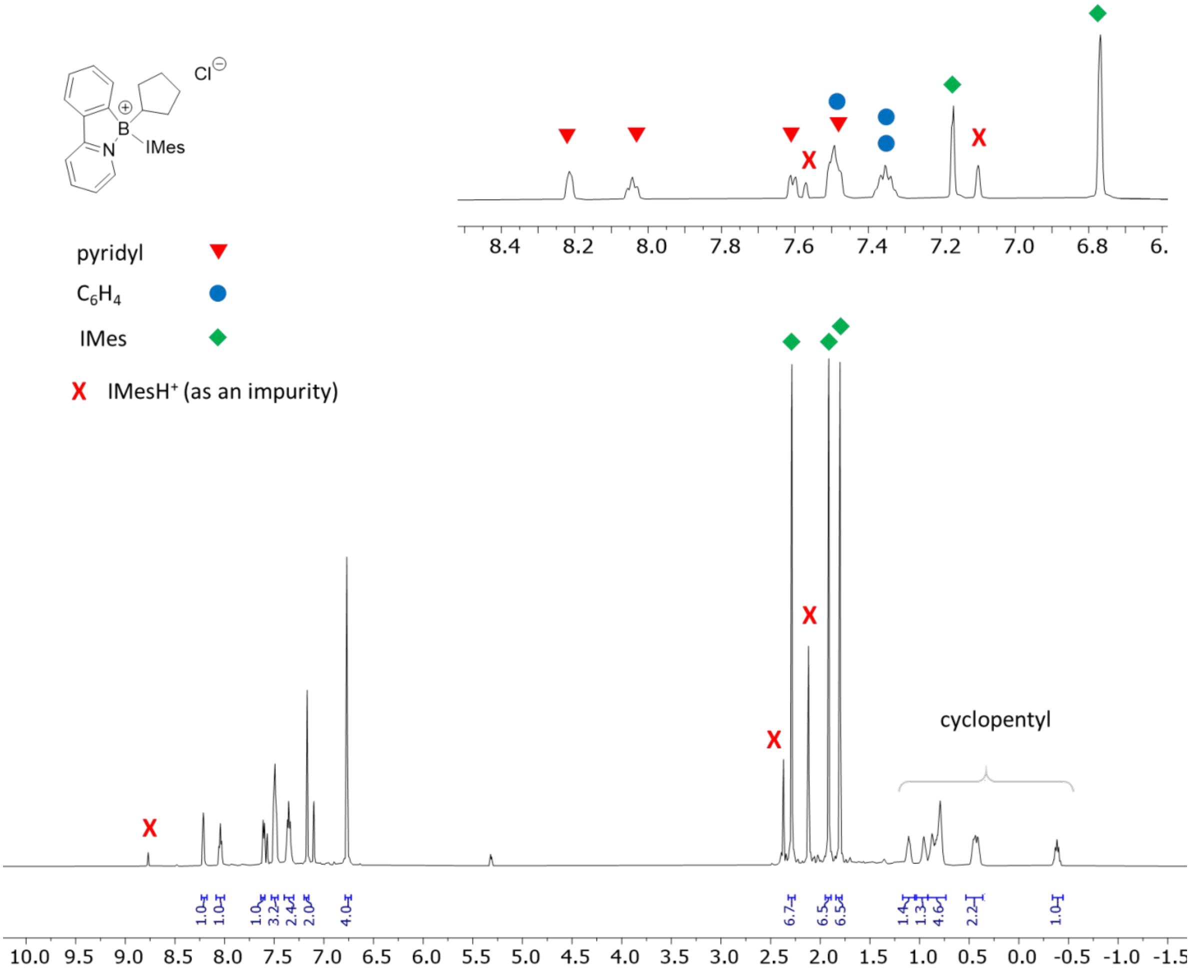

Figure S30: **^{1}H NMR** ($CDCl_3$, 600 MHz, 293 K) spectrum of compound **7c**.

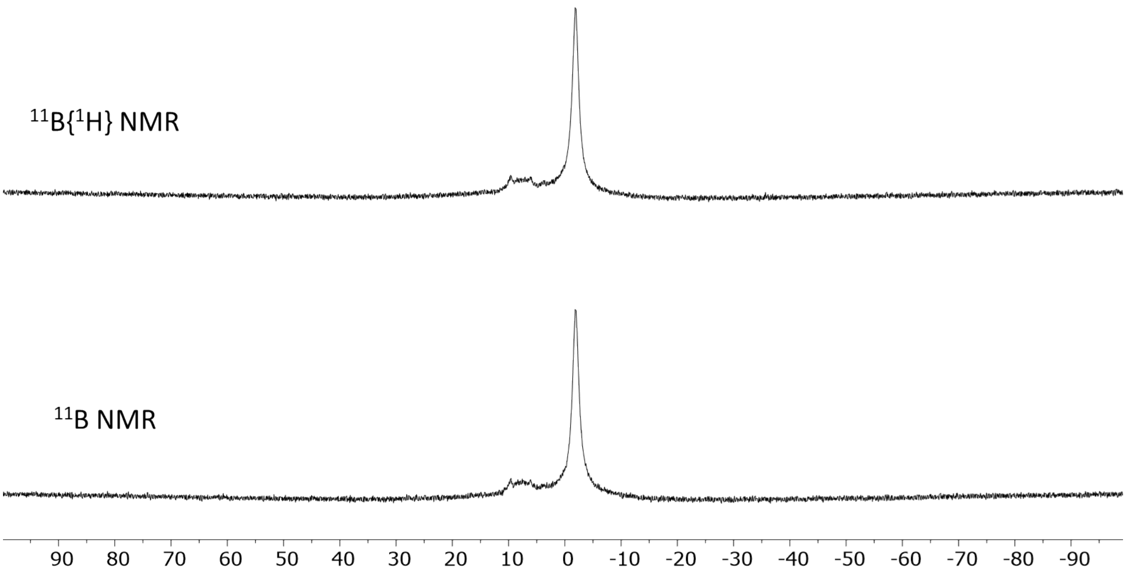

Figure S31: **^{11}B** and **^{11}B{^{1}H} NMR** (193 MHz, $CDCl_3$, 293 K) spectra of compound **7c**.

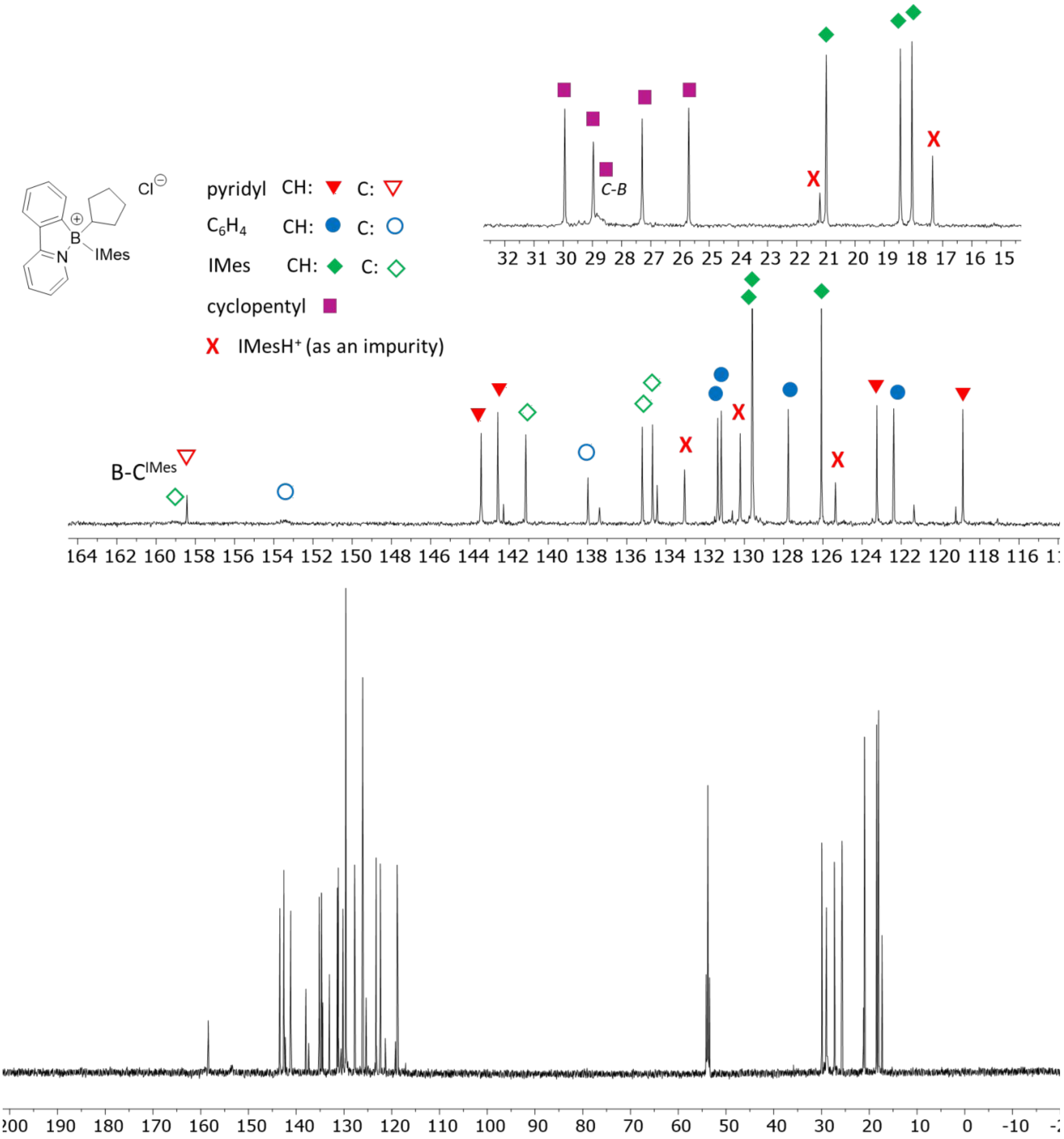

Figure S32: ^{13}**C{**^{1}**H} NMR** (151 MHz, $CDCl_3$, 293 K) spectrum of compound **7c**.

Crystal structure determination of **7c [KSK487-gg]**

A crystal suitable for SC-XRD structure determination was obtained by a slow diffusion of hexane into a solution of **7c** in dichloromethane in argon filled glovebox.

Crystal Data for $C_{37.5}H_{42.6}BCl_2N_3$ (M =617.05 g/mol): monoclinic, space group C2/c (no. 15), a = 37.2814(6) Å, b = 9.29980(10) Å, c = 19.3742(3) Å, β = 99.370(2)°, V = 6627.60(17) Å^3, Z = 8, T = 100.00(10) K, μ(Cu Kα) = 1.984 mm^{-1}, $Dcalc$ = 1.237 g/cm^3, 45838 reflections measured (9.252° ≤ 2Θ ≤ 153.762°), 6821 unique (R_{int} = 0.0377, R_{sigma} = 0.0234) which were used in all calculations. The final R_1 was 0.0854 (I > 2σ(I)) and wR_2 was 0.2297 (all data). **CCDC: 2445689**

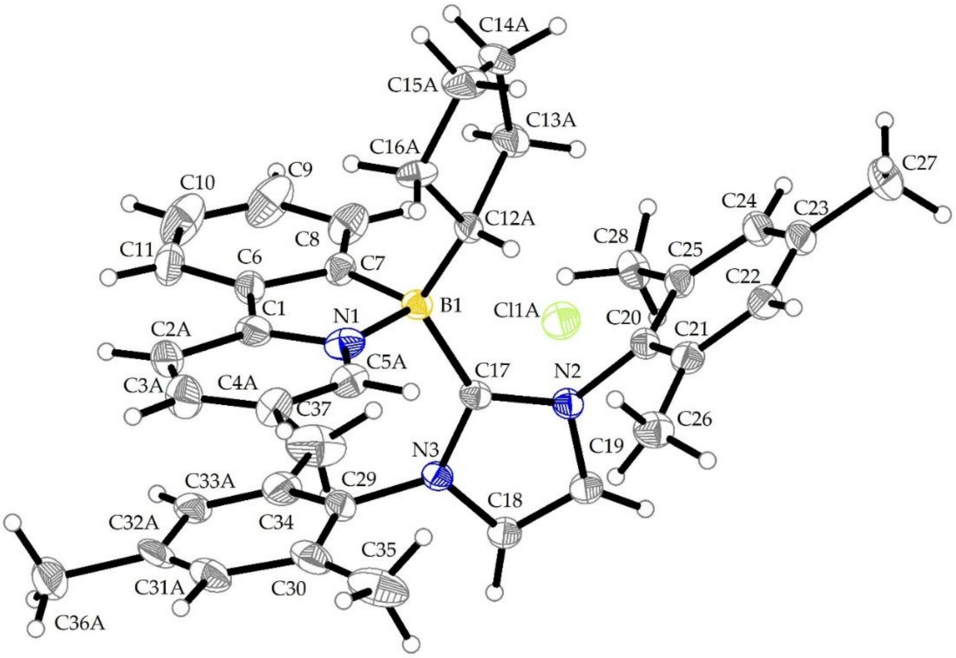

Figure S33: Crystal structure of **7c** (thermal ellipsoids shown at 30 % probability level). Some disordered atoms and dichloromethane are omitted for clarity.

NMR experiment

Boron azide **3c** (13.8 mg, 0.05 mmol) and IMes (15.2 mg, 0.05 mmol) were combined in Toluene-d_8 (ca 0.5 mL in total), mixture turned dark purple/black almost immediately and some precipitate was formed. Reaction mixture was directly analyzed with NMR revealing formation of complex product mixture.

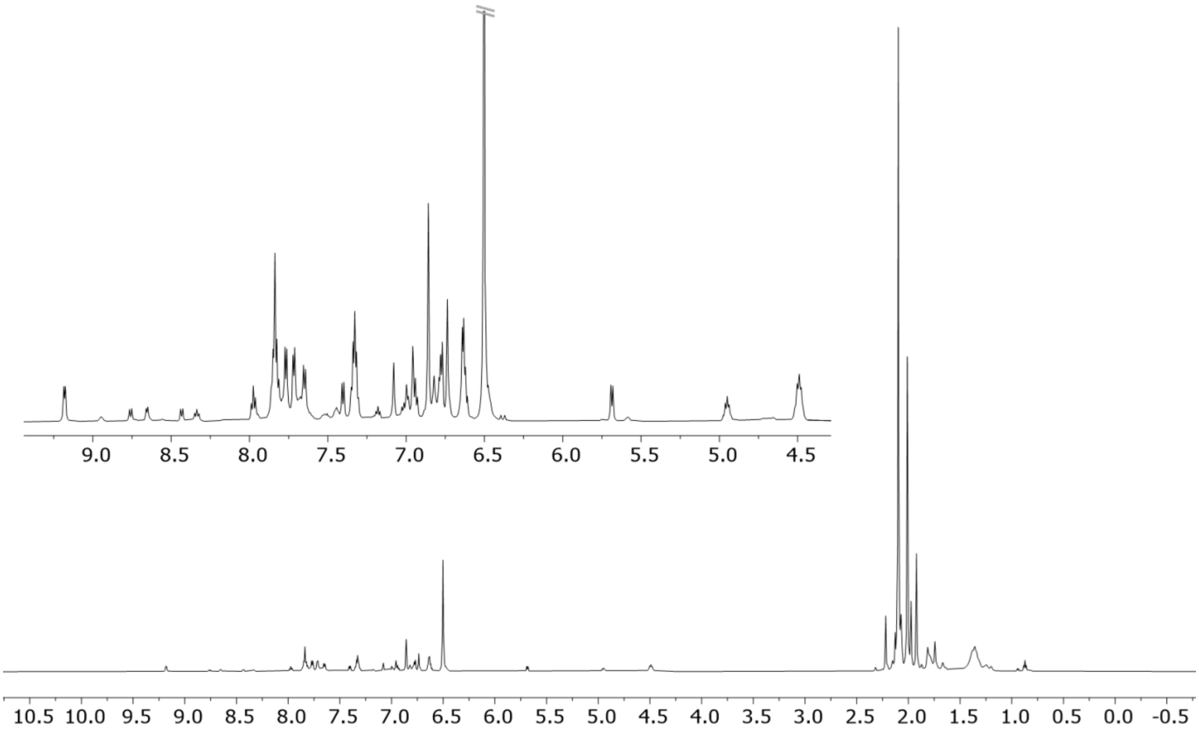

Figure S34: ^{1}**H NMR** (Tol-d_8, 600 MHz, 293 K) spectrum of reaction of **3c** with IMes.

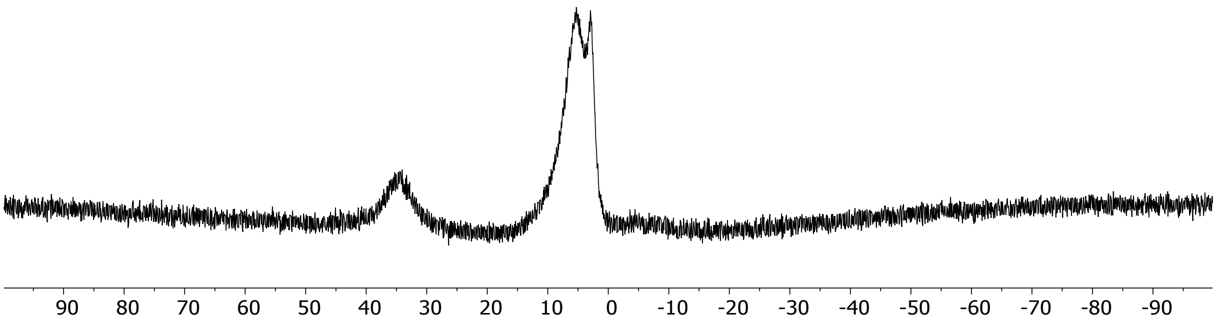

Figure S35: ^{11}**B{^{1}H} NMR** (Tol-d_8, 193 MHz, $CDCl_3$, 293 K) spectrum of reaction of **3c** with IMes.

Reductive chemistry of boron azides, preparation of **8-13**

Preparation of **8**

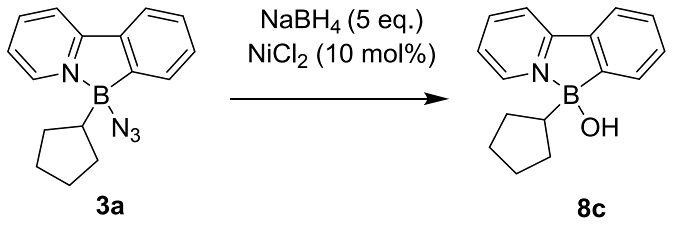

Azide **3a** (138 mg, 0.500 mmol) was dissolved in a mixture of tetrahydrofuran (5 mL) and deionized water (5 mL). $NaBH_4$ (95 mg, 2.5 mmol, 5 equiv.) was added, followed by $NiCl_2{\cdot}6H_2O$ (12 mg, 0.050 mmol, 0.1 equiv.). Upon addition of the nickel salt, a dark precipitate was formed and some effervescence occurred. The mixture was stirred for 3 hours at room temperature, then filtered and diluted with 20 mL of ethyl acetate. Organic phases were separated and the aqueous one was extracted with ethyl acetate (3x5 mL). Combined organic extracts were washed with brine and dried over $MgSO_4$. After filtration and removal of volatiles, the resulting oily mixture was purified by column chromatography on silica gel dichloromethane / methanol (10:1) mixture as eluent. Traces of unreacted azide alongside with minor impurities were eluted first, followed by compound **8c**. Borinic acid **8c** was obtained as thick colourless oil, which solidified to give an off-white solid upon standing (90 mg, 0.38 mmol, 76 % yield).

Elemental analysis calculated for $C_{16}H_{18}BNO$ (251.1) C 76.52, H 7.22, N 5.58; found C 76.18, H 7.02, N 5.09.

HRMS for $C_{16}H_{19}NBO^+$ [monomer+H]$^+$: calculated 252.1563 found 252.1554 and $C_{32}H_{35}N_2B_2O$ [dimer+H]$^+$ calculated 485.2946 found 485.2958

IR (KBr) ν: 3392 (br m, OH), 3055 (w), 2935 (m), 2854 (m), 1616 (s), 1572 (m), 1562 (w), 1482 (s), 1445 (m), 1327 (m), 1306 (w), 1287 (w), 1187 (m), 1158 (s), 1120 (m), 1052 (s), 1024 (s), 963 (w), 941 (m), 908 (m), 800 (w), 764 (s), 739 (s), 641 (w), 558 (w), 489 (w), 426 (m) cm^{-1}.

^{1}H NMR (600 MHz, $CDCl_3$, 293K): δ = [8.58 (d, $^3J_{HH}$ = 5.5 Hz), 7.96 (dd, $^3J_{HH} \approx {}^3J_{HH}$ = 7.8 Hz), 7.81 (d, , $^3J_{HH}$ = 7.8 Hz), 7.38 (dd, $^3J_{HH}$ = 5.5 Hz $^3J_{HH}$ = 7.8 Hz)](each 1H, pyridyl), [7.70 (d, $^3J_{HH}$ = 7.76 Hz), 7.60 (d, $^3J_{HH}$ = 7.3 Hz), 7.34 (dd, $^3J_{HH}$ = 7.3 Hz, $^3J_{HH}$ = 7.7 Hz), 7.29 (dd, $^3J_{HH} \approx {}^3J_{HH}$ = 7.6 Hz)](each 1H, C_6H_4), 4.11 (br s, 1H, OH), [1.72 (m, 1H), 1.38 (m, 3H), 1.20 (m, 2H), 1.03 (m, 1H), 0.94 (m, 1H), 0.40 (m, 1H)](cyclopentyl).

^{13}C{^{1}H} NMR (151 MHz, $CDCl_3$, 293K): δ = [155.5 (*i*-C), 142.7 (CH), 140.9 (CH), 122.4 (CH), 117.3 (CH)](pyridyl), [155.7 (br, C-B), 136.5 (*i*-C), 130.7 (CH), 130.6 (CH), 126.8 (CH), 121.1 (CH)](C_6H_4), [34.6 (br, C-B), 28.8 (CH_2), 28.7 (CH_2), 27.0 (CH_2), 26.4 (CH_2)](cyclopentyl).

^{11}B{^{1}H} NMR (193 MHz, $CDCl_3$, 293K): δ = 8.2 ($\nu_{1/2} \approx$ 300 Hz).

^{11}B NMR (193 MHz, $CDCl_3$, 293K): δ = 8.2 ($\nu_{1/2} \approx$ 320 Hz).

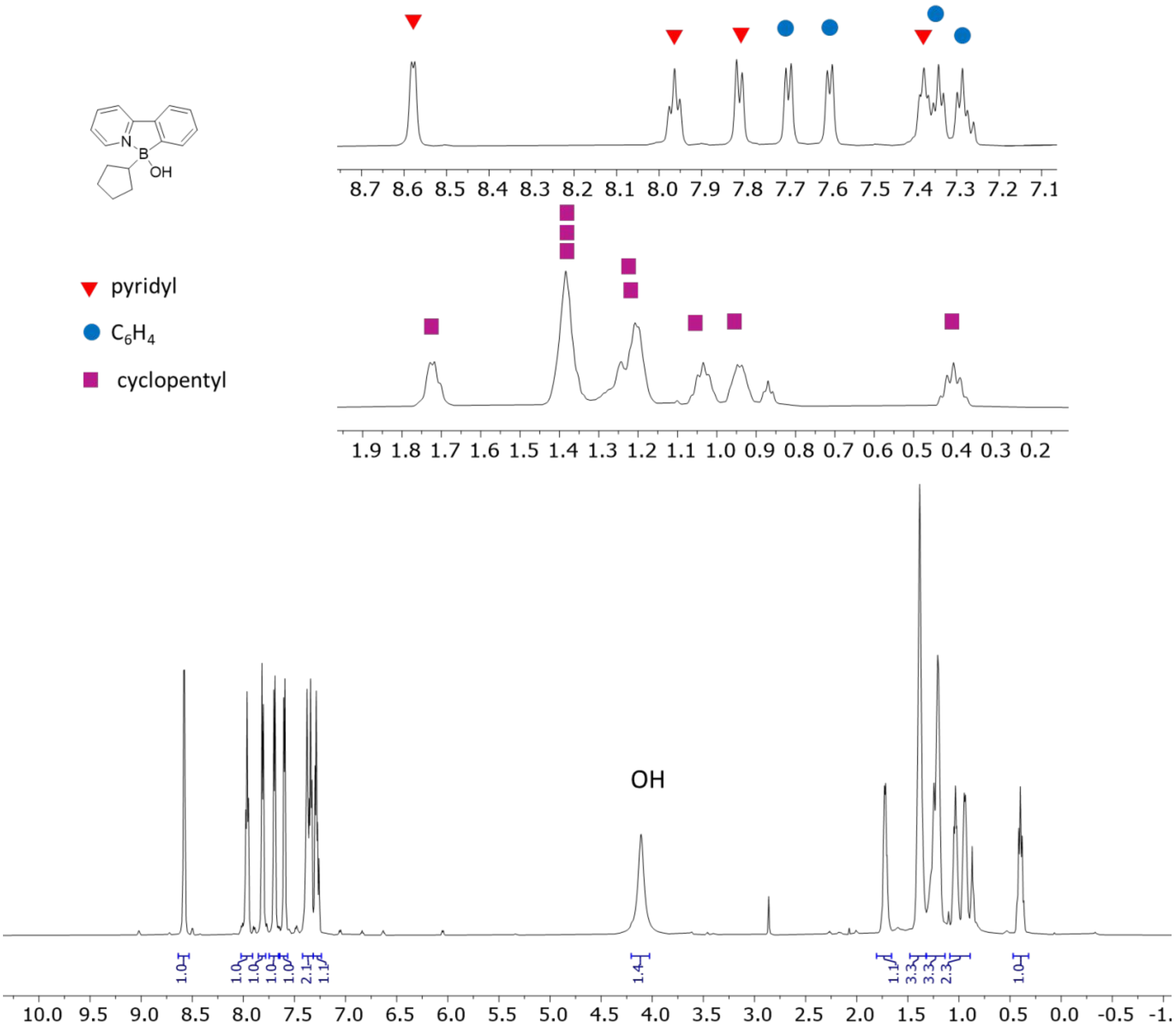

Figure S36: **^{1}H NMR** ($CDCl_3$, 600 MHz, 293 K) spectrum of compound **8c**.

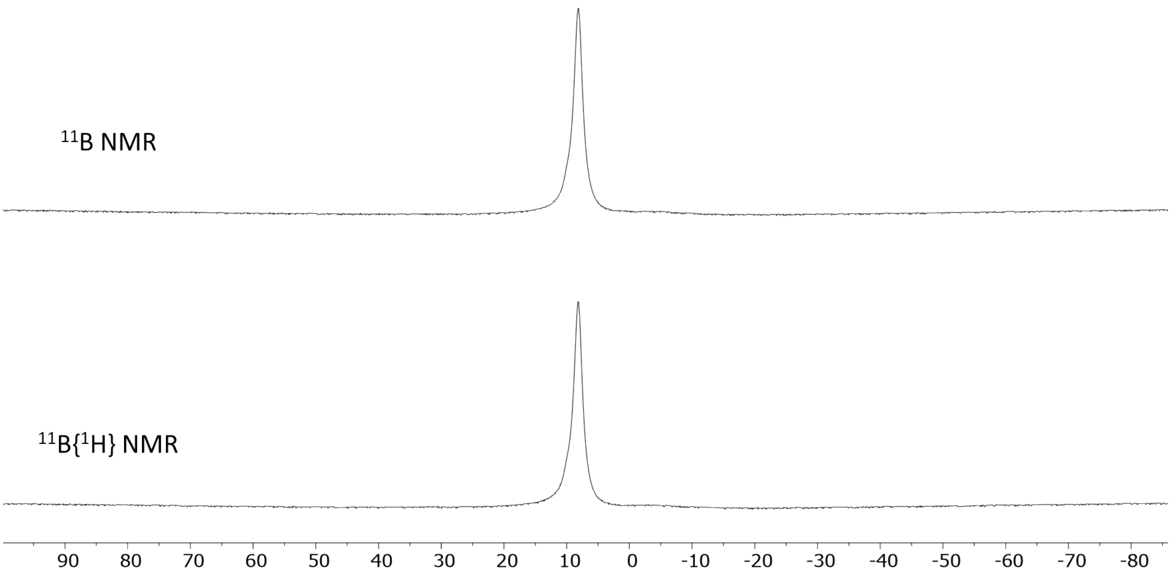

Figure S37: **^{11}B** and **^{11}B{^{1}H} NMR** (193 MHz, $CDCl_3$, 293 K) spectra of compound **8c**.

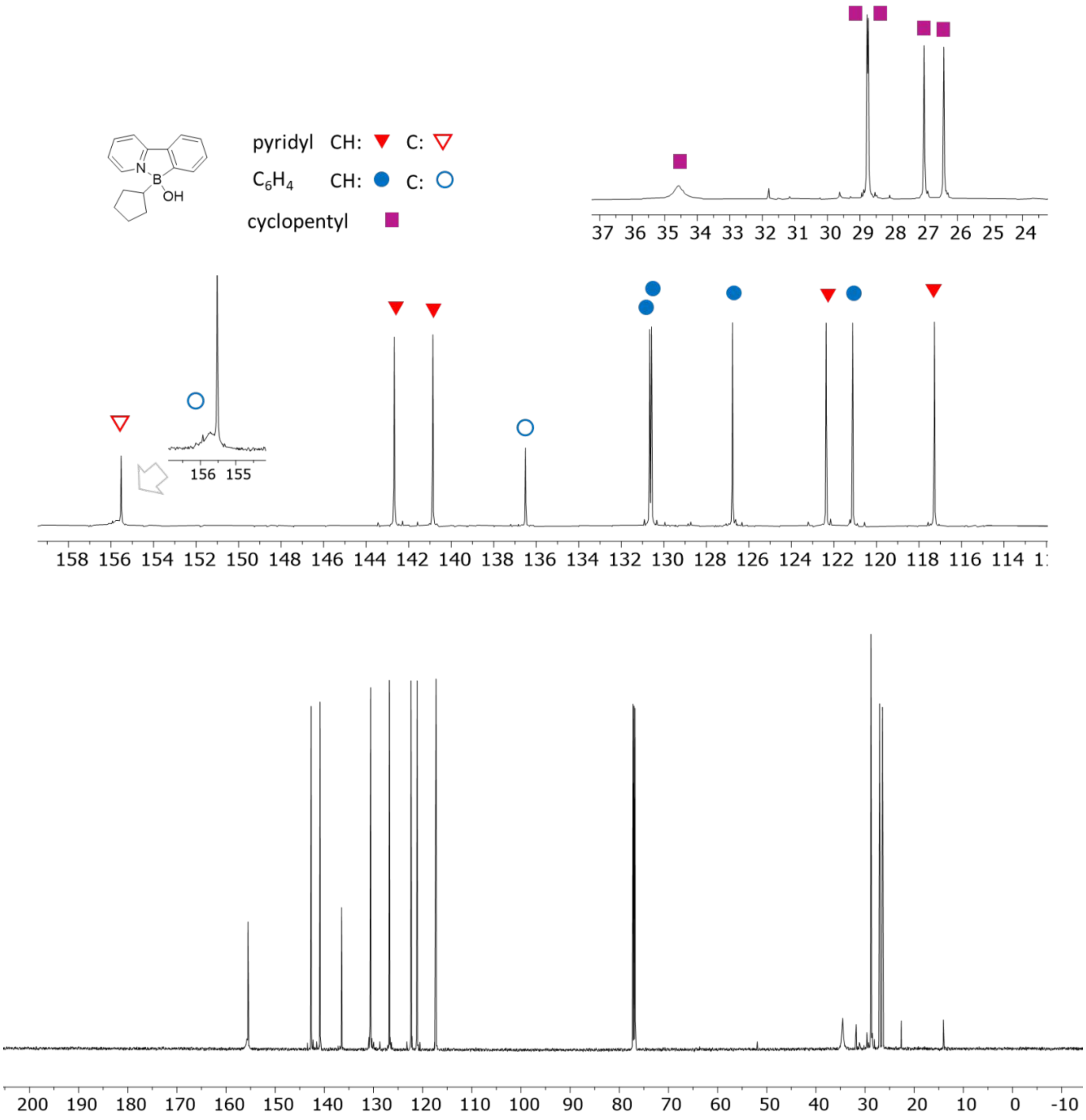

Figure S38: **^{13}C{^{1}H} NMR** (151 MHz, $CDCl_3$, 293 K) spectrum of compound **8c**.

Crystal structure determination of **8c** [KSK468-gg]

A crystal suitable for SC-XRD structure determination was obtained by slow evaporation of a solution of **8c** in a CH_2Cl_2/heptane mixture (ca 1:1 vol.) at ambient conditions.

Crystal Data for $C_{71}H_{88}B_4N_4O_4$ (M =1104.69 g/mol): orthorhombic, space group Pbca (no. 61), a = 28.0645(4) Å, b = 15.4730(2) Å, c = 29.2493(3) Å, V = 12701.3(3) Å^3, Z = 8, T = 100.00(10) K, μ(Cu Kα) = 0.536 mm^{-1}, $Dcalc$ = 1.155 g/cm^3, 50719 reflections measured (6.298° ≤ 2Θ ≤ 133.182°), 11215 unique (R_{int} = 0.0356, R_{sigma} = 0.0302) which were used in all calculations. The final R_1 was 0.0499 (I > 2σ(I)) and wR_2 was 0.1346 (all data). **CCDC: 2445691**

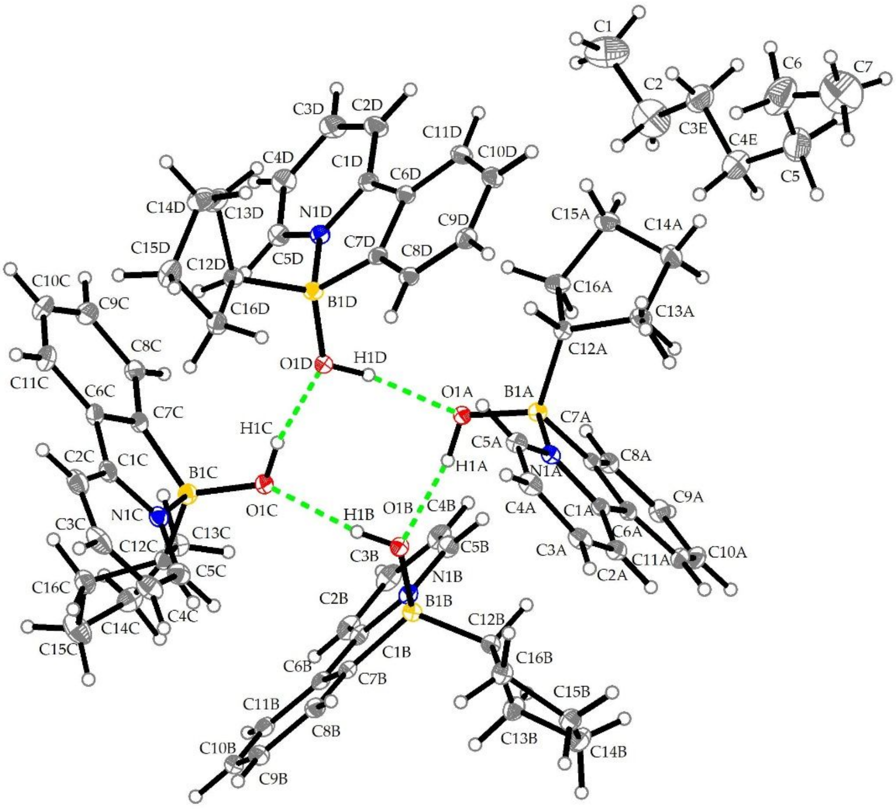

Figure S39: Crystal structure of **8c** (thermal ellipsoids shown at 30 % probability level). Some disordered atoms are omitted for clarity.

Preparation of **9c**

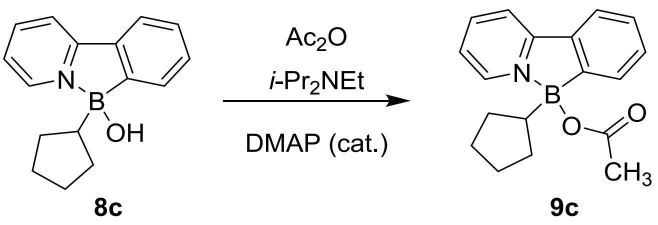

Borinic acid **8c** (87 mg, 0.35 mmol) and DMAP (4-dimethylaminopyridine, 4.4 mg, 0.035 mmol, 0.1 eq.) were dissolved in dry dichloromethane (5 mL) and dry *i*-Pr_2NEt (0.30 mL, 1.8 mmol, 5 equiv.) was added, followed by acetic anhydride (0.07 mL, 0.7 mmol, 2 equiv.). The mixture was stirred for four hours at room temperature and subsequently quenched by addition of saturated aqueous $NaHCO_3$ solution. Aqueous phase was separated and extracted with dichloromethane (2x5 mL). Combined organic fractions were combined, washed with brine and dried with $MgSO_4$. After filtration and removal of volatiles, the pale-yellow residue was purified by column chromatography on silica gel using AcOEt / Hx (1:1) as eluent. Product **9c** was obtained as a white solid (92 mg, 0.31 mmol, 90% yield).

Elemental analysis calculated for $C_{18}H_{20}BNO_2$ (293.2) C 73.14, H 6.88, N 4.78; found C 72.99, H 7.06, N 4.68.

IR (KBr) ν: 3197 (w), 3062 (w), 2935 (m), 2853 (m), 1666 **(s, CO)**, 1646 (m), 1625 (s), 1561 (s), 1515 (s), 1488 (m), 1467 (s), 1376 (s), 1304 (s), 1278 (s), 1208 (m), 1163 (m), 1090 (s), 1031 (w), 981 (s), 946 (s), 905 (s), 888 (s), 821 (w), 789 (m), 761 (s), 739 (s), 688 (w), 637 (w), 614 (m), 556 (m), 539 (m), 523 (m), 473 (w), 443 (w), 430 (w) cm^{-1}.

^{1}H NMR (600 MHz, $CDCl_3$, 293 K): δ = [8.64 (d, $^3J_{HH}$ = 5.6 Hz), 7.99 (dd, $^3J_{HH} \approx {}^3J_{HH}$ = 7.8 Hz), 7.90 (d, $^3J_{HH}$ = 7.8 Hz), 7.36 (dd, $^3J_{HH}$ = 7.8 Hz, $^3J_{HH}$ = 5.6 Hz)](each 1H, pyridyl), [7.77 (d, $^3J_{HH}$ = 7.6 Hz), 7.71 (d, $^3J_{HH}$ = 7.2 Hz), 7.42 (dd, $^3J_{HH} \approx {}^3J_{HH}$ = 7.5 Hz), 7.33 (dd, $^3J_{HH} \approx {}^3J_{HH}$ = 7.6 Hz)](each 1H, C_6H_4), 2.00 (s, 3H, $COCH_3$), [1.76 (m, 1H), 1.53-1.41 (m, 3H), 1.34-1.25 (m, 3H), 1.10 (m, 1H), 0.6 (m, 1H)](cyclopentyl).

^{13}C{^{1}H} NMR (151 MHz, $CDCl_3$, 293 K): δ = [173.2 (CO), 23.4(CH_3)](Ac), [157.0 (*i*-C), 142.5 (CH), 141.1 (CH), 121.9 (CH), 117.7 (CH)](pyridyl), [155.0 (br, C-B), 136.5 (*i*-C), 130.9 (CH), 130.1 (CH), 126.7 (CH), 121.3 (CH)](C_6H_4), [33.1 (br, C-B), 28.7 (CH_2), 28.2 (CH_2), 26.9 (CH_2), 26.4 (CH_2)](cyclopentyl).

^{11}B{^{1}H} NMR (193 MHz, $CDCl_3$, 293 K): δ = 6.7 ($\nu_{1/2} \approx$ 200 Hz).

^{11}B NMR (193 MHz, $CDCl_3$, 293 K): δ = 6.7 ($\nu_{1/2} \approx$ 200 Hz).

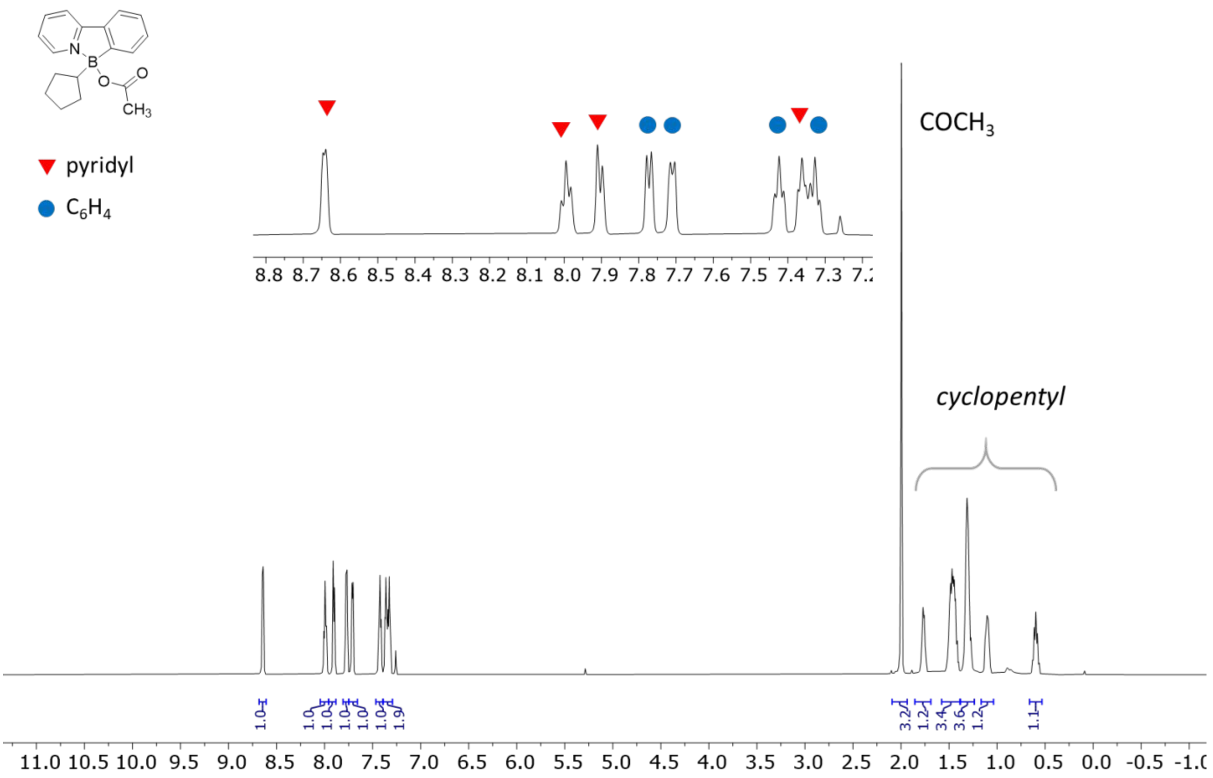

Figure S40: **^{1}H NMR** ($CDCl_3$, 600 MHz, 293 K) spectrum of compound **9c**.

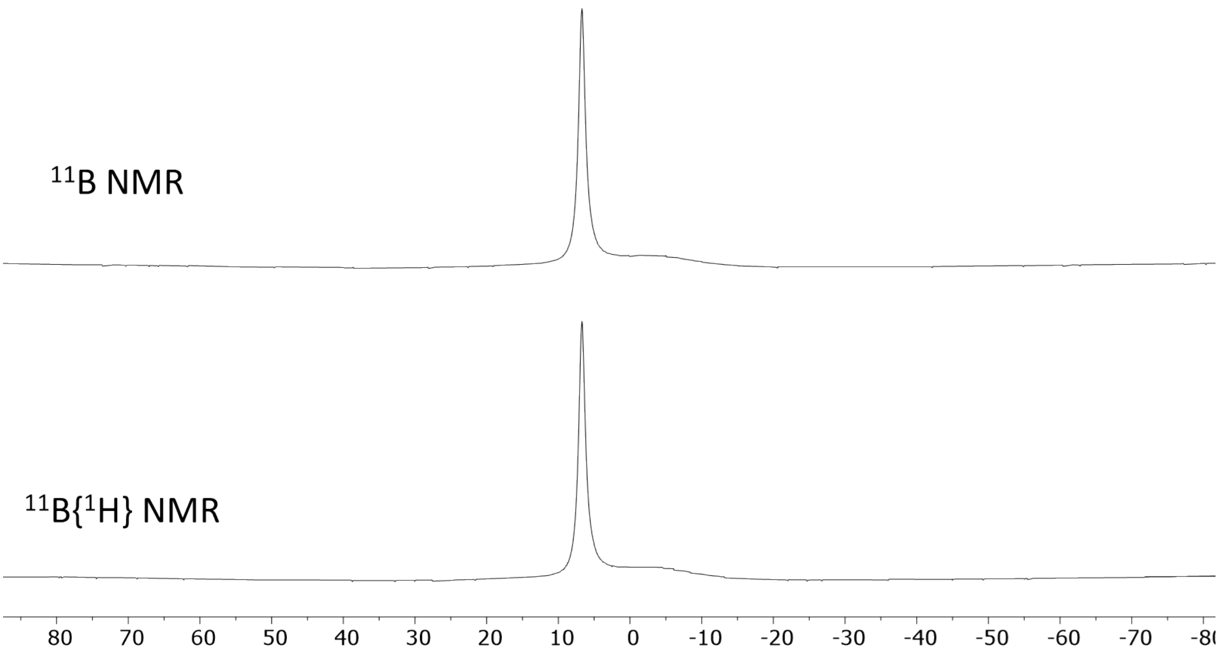

Figure S41: **^{11}B** and **^{11}B{^{1}H} NMR** (193 MHz, $CDCl_3$, 293 K) spectra of compound **9c**.

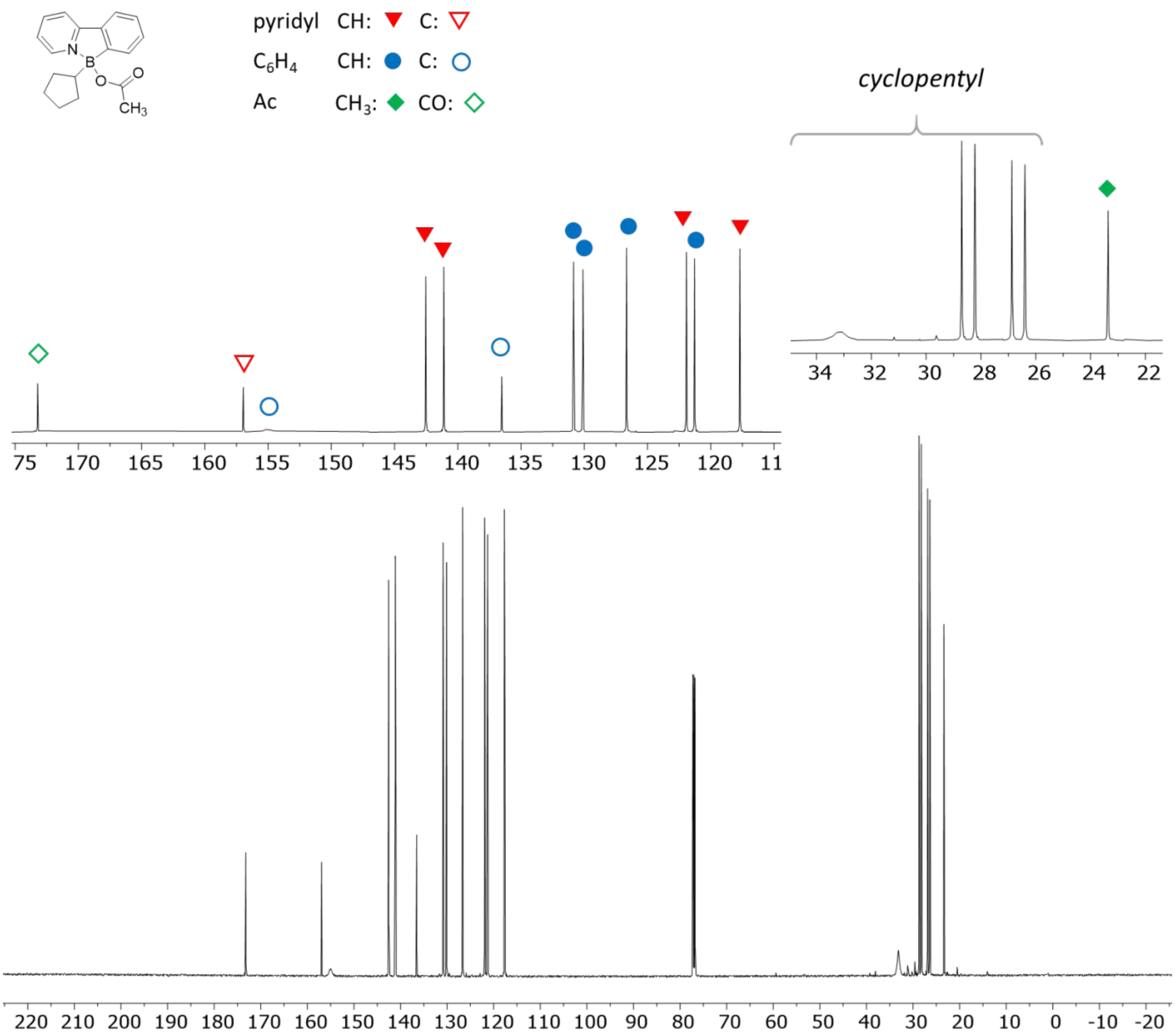

Figure S42: **^{13}C{^{1}H} NMR** (151 MHz, $CDCl_3$, 293 K) spectrum of compound **9c**.

Crystal structure determination of **9c** [KSK470-gg]

A crystal suitable for SC-XRD structure determination was obtained by slow evaporation of a solution of **9c** in a CH_2Cl_2/heptane mixture (cca 1:1 vol.) at ambient conditions.

Crystal Data for $C_{18}H_{20}BNO_2$ (M =293.16 g/mol): monoclinic, space group $P2_1/c$ (no. 14), a = 14.1610(3) Å, b = 8.86639(18) Å, c = 13.0386(3) Å, β = 99.638(2)°, V = 1613.98(6) Å^3, Z = 4, T = 100.00(10) K, μ(Cu Kα) = 0.608 mm^{-1}, $Dcalc$ = 1.206 g/cm^3, 18789 reflections measured (6.33° ≤ 2Θ ≤ 153.116°), 3311 unique (R_{int} = 0.0369, R_{sigma} = 0.0238) which were used in all calculations. The final R_1 was 0.0448 (I > 2σ(I)) and wR_2 was 0.1199 (all data). **CCDC: 2445688**

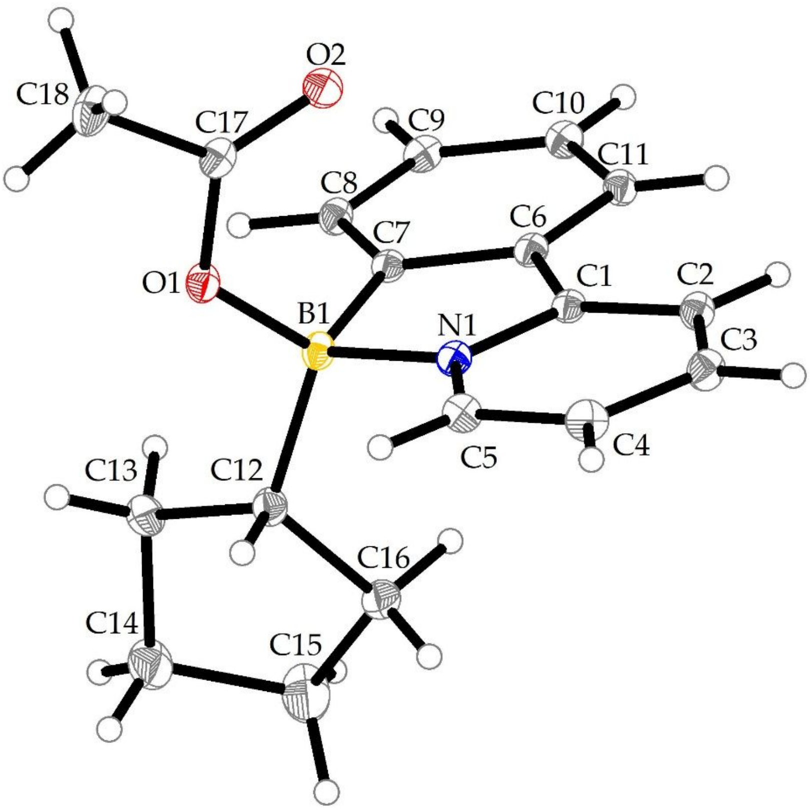

Figure S43: Crystal structure of **9c** (thermal ellipsoids shown at 30 % probability level).

Preparation of **10c**

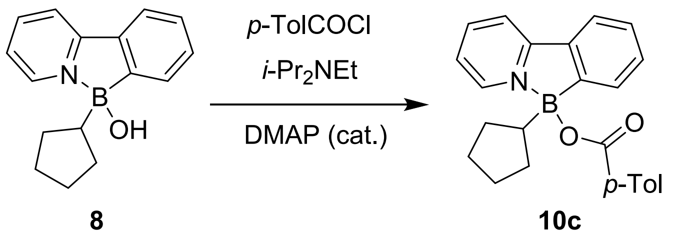

Borinic acid **8** (50 mg, 0.20 mmol) and 4-dimethylaminopyridine (2.4 mg, 0.020 mmol, 0.1 eq.) were dissolved in dry dichloromethane (5 mL) and dry *i*-Pr_2NEt (0.14 mL, 1.0 mmol, 5 eq.) was added, followed by 4-methylbenzoyl chloride (0.05 mL, 0.4 mmol, 2 equiv.). The mixture was stirred for four hours at room temperature and subsequently quenched by addition of saturated aqueous $NaHCO_3$ solution. Aqueous phase was separated and extracted with dichloromethane (2x5 mL). Organic fractions were combined, washed with brine and dried over $MgSO_4$. After filtration and removal of volatiles, the pale-yellow residue was purified by column chromatography on silica gel using AcOEt / Hx (1:1) as eluent. Product **10c** was obtained as a white solid (40 mg, 0.11 mmol, 55% yield).

Satisfactory elementary analysis was not obtained.

HRMS for $C_{24}H_{24}NBO_2Na^+$ $[M+Na]^+$: calculated 392.1805, found 392.1972.

IR (KBr) ν: 2940 (m), 2853 (m), **1672 (s, CO)**, 1621 (m), 1571 (w), 1486 (s), 1448 (m), 1408 (w), 1376 (w), 1327 (s), 1316 (s), 1292 (m), 1251 (w), 1176 (m), 1163 (w), 1135 (m) 1127 (m), 1111 (w), 1067 (m), 1015 (w), 961 (w), 942 (w), 922 (m), 889 (w), 840 (w), 794 (w), 758 (s), 734 (s), 692 (w), 649 (w), 574 (w), 491 (w), 425 (w) cm^{-1}.

^{1}H NMR (600 MHz, $CDCl_3$, 293 K): δ = [8.65 (d, $^3J_{HH}$ = 5.7 Hz), 8.02 (dd, $^3J_{HH} \approx {}^3J_{HH}$ = 7.8 Hz), 7.95 (m), 7.35 (m)](each 1H, pyridyl), [7.82 (d, $^3J_{HH}$ = 7.6 Hz), 7.71 (d, $^3J_{HH}$ = 7.2 Hz), 7.41 (dd, $^3J_{HH} \approx {}^3J_{HH}$ = 7.5 Hz), 7.35 (m)](each 1H, C_6H_4), [7.96 (m, 2H, CH), 7.18 (m, 2H, CH), 2.38 (s, 3H, CH_3)](*p*-Tol), [1.87, 1.59, 1.58, 1.50, 1.39, 1.39, 1.32, 1.25, 0.80](each m, 1H, cyclopentyl, estimated from gHSQC).

^{13}C{^{1}H} NMR (151 MHz, $CDCl_3$, 293 K): δ = [167.9 (CO), 142.1 (*i*-C), 130.9 (*i*-C), 129.8 (CH), 128.7 (CH), 21.5 (CH_3)](*p*-TolCO), [157.1 (*i*-C), 142.4 (CH), 141.2 (CH), 122.0 (CH), 117.9 (CH)](pyridyl), [155.3 (br, C-B), 136.5 (*i*-C), 130.9 (CH), 130.1 (CH), 126.7 (CH), 121.4 (CH)](C_6H_4), [33.8 (br, C-B), 28.8 (CH_2), 28.4 (CH_2), 27.0 (CH_2), 26.6 (CH_2)](cyclopentyl).

^{11}B{^{1}H} NMR (193 MHz, $CDCl_3$, 293 K): δ = 6.8 ($\nu_{1/2} \approx$ 250 Hz).

^{11}B NMR (193 MHz, $CDCl_3$, 293 K): δ = 6.8 ($\nu_{1/2} \approx$ 250 Hz).

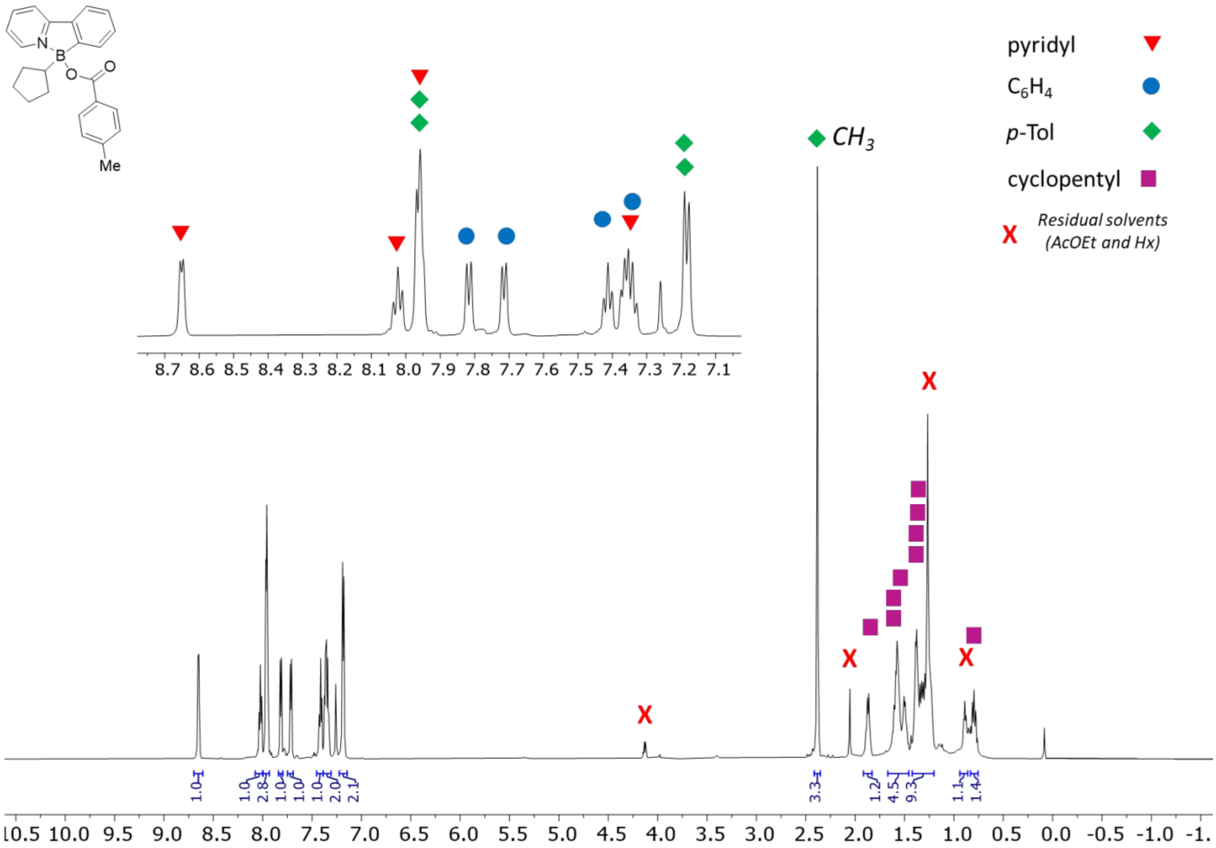

Figure S44: **^{1}H NMR** ($CDCl_3$, 600 MHz, 293 K) spectrum of compound **10c**, Hx denotes residual hexane.

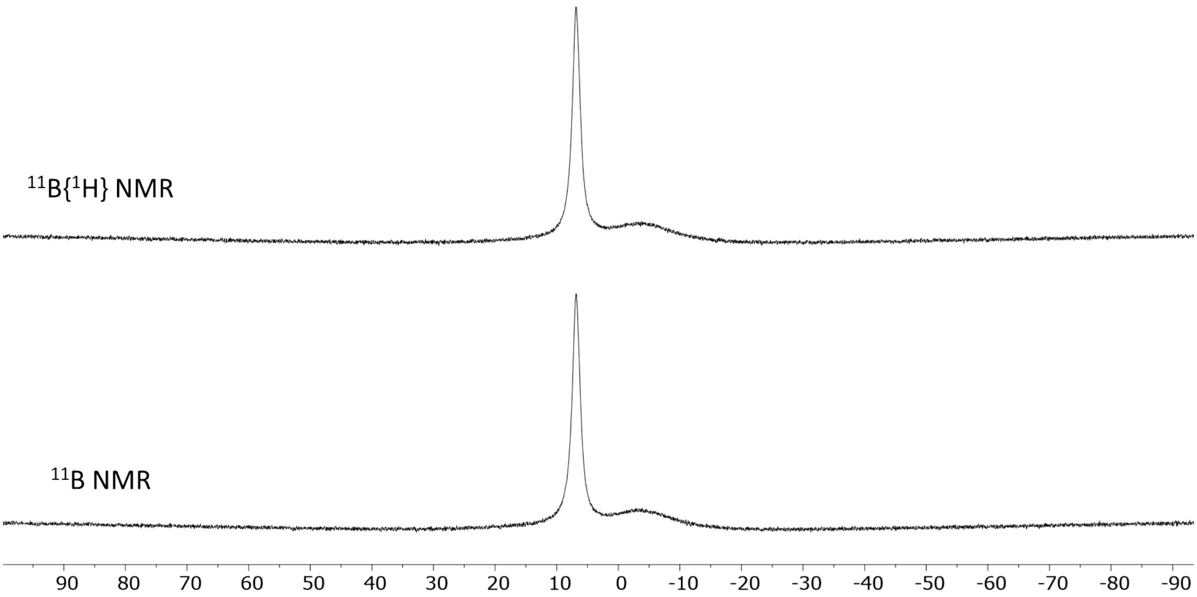

Figure S45: **^{11}B** and **^{11}B{^{1}H} NMR** (193 MHz, $CDCl_3$, 293 K) spectra of compound **10c**.

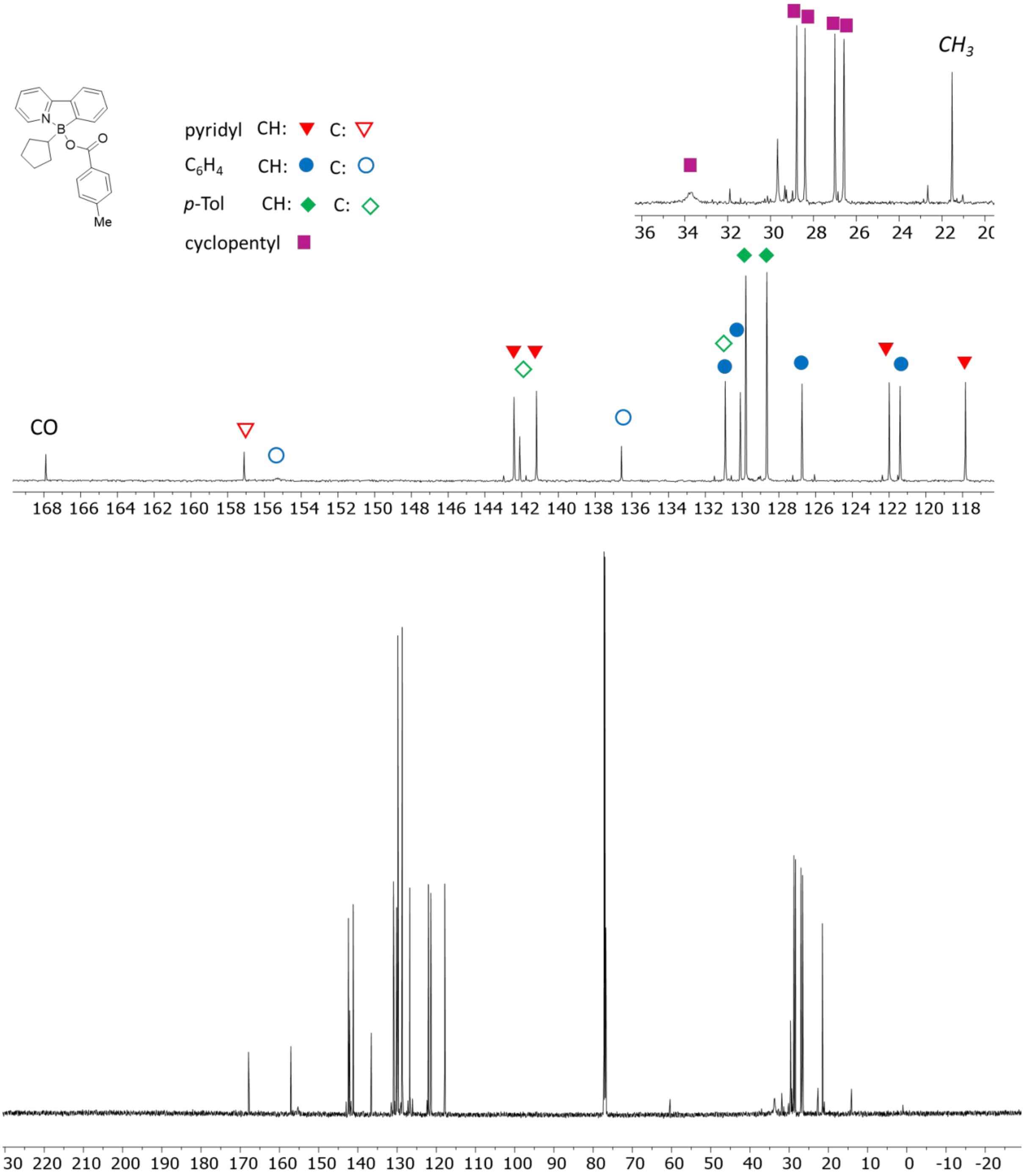

Figure S46: **^{13}C{^{1}H} NMR** (151 MHz, $CDCl_3$, 293 K) spectrum of compound **10c**.

Preparation of **11a**

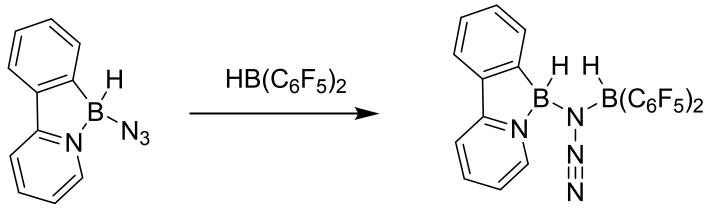

1 **3a** **11a**

Boron azide **3a** (62.4 mg, 0.300 mmol) and $HB(C_6F_5)_2$ (103 mg, 0.300 mmol) were combined in dichloromethane (5 mL) and stirred for 30 minutes. The reaction mixture was then filtered and concentrated *in vacuo*. The solid residue was washed with dichloromethane / hexane mixture (ca 5 mL of ca 4:1) three times and subsequently dried *in vacuo* to yield compound **11a** as a white solid (155 mg, 0.280 mmol, 93% yield).

Elemental analysis calculated for $C_{23}H_{10}N_4B_2F_{10}$ (554.1): C 49.87, H 1.82, N 10.11; found C 50.03, H 1.78, N 9.65

IR (ATR): 3086 (w), 3065 (w), **2451 (m, B-H), 2418 (m, B-H), 2164 (vs, N_3)**, 1648 (s), 1626 (s), 1564 (m), 1573 (m), 1517 (s), 1472 (s), 1395 (m), 1380 (m), 1330 (m), 1310 (w), 1289 (s), 1232 (s), 1174 (m), 1130 (m), 1083 (s), 1006 (m), 974 (s), 938 (m), 910 (m), 872 (m), 851 (w), 824 (w), 797 (m), 771 (s), 749 (w), 682 (m), 636 (w), 618 (m), 602 (w), 572 (w), 554 (w) cm^{-1}.

^{1}H NMR (600 MHz, CD_2Cl_2, 293 K): δ = [8.72 (d, $^3J_{HH}$ = 5.8 Hz), 8.22 (dd, $^3J_{HH} \approx {}^3J_{HH} \approx$ 8.0 Hz), 8.01 (d, $^3J_{HH}$ = 8.1 Hz), 7.57 (m)](each 1H, pyridyl), [7.82 (m), 7.54 (m), 7.43 (m), 7.42 (m)](each 1H, C_6H_4), 3.92 (br m, 1H, B-H), 3.37 (br m, 1H, B-H).

^{13}C{^{1}H} NMR (151 MHz, CD_2Cl_2, 293 K): δ = [158.7 (*i*-C), 144.7 (CH), 144.2 (CH), 123.5 (CH), 119.0 (CH)](pyridyl), [147.3 (br, C-B), 137.6 (*i*-C), 132.0 (CH), 131.3 (CH), 129.1 (CH), 122.4 (CH)](C_6H_4), [148.5 (dm, $^1J_{FC} \approx$ 240 Hz), 140.1 (dm, $^1J_{FC} \approx$ 250 Hz), 137.2 (dm, $^1J_{FC} \approx$ 250 Hz), 116 (br m, C-B)](C_6F_5).

^{11}B{^{1}H} NMR (193 MHz, CD_2Cl_2, 293 K): δ = 0.8 ($\nu_{1/2} \approx$ 200 Hz), –12.0 ($\nu_{1/2} \approx$ 200 Hz).

^{11}B NMR (193 MHz, CD_2Cl_2, 293 K): δ = 0.8 ($\nu_{1/2} \approx$ 300 Hz), –12.0 ($\nu_{1/2} \approx$ 300 Hz).

^{19}F NMR (564 MHz, CD_2Cl_2, 293 K): δ = [–134.0, –134.7](each m, 2F, *o*-C_6F_5), [–158.5, –158.7](each m, 1F, *p*-C_6F_5), [–164.7, –165.0](each m, 2F, *m*-C_6F_5).

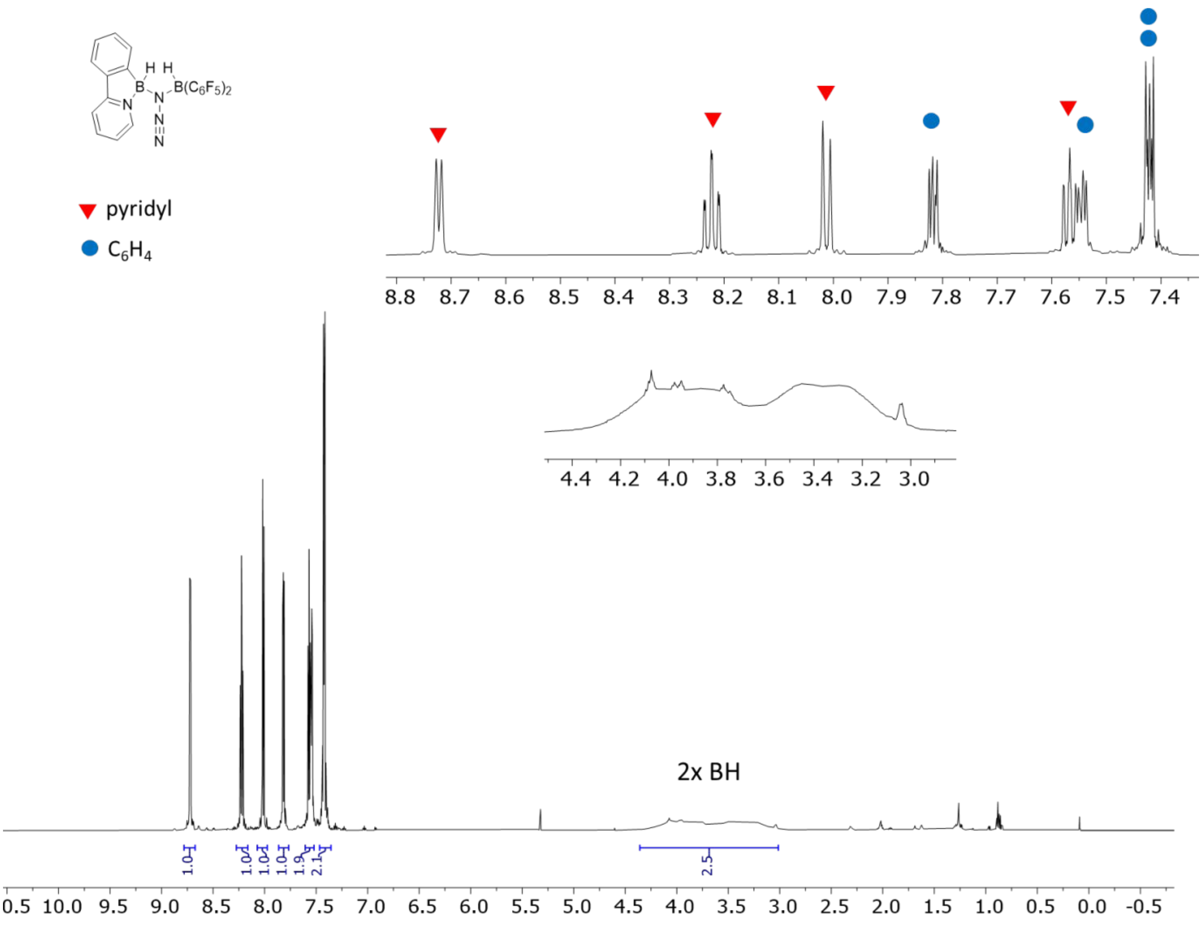

Figure S47: **^{1}H NMR** (CD_2Cl_2, 600 MHz, 293 K) spectrum of compound **11a**.

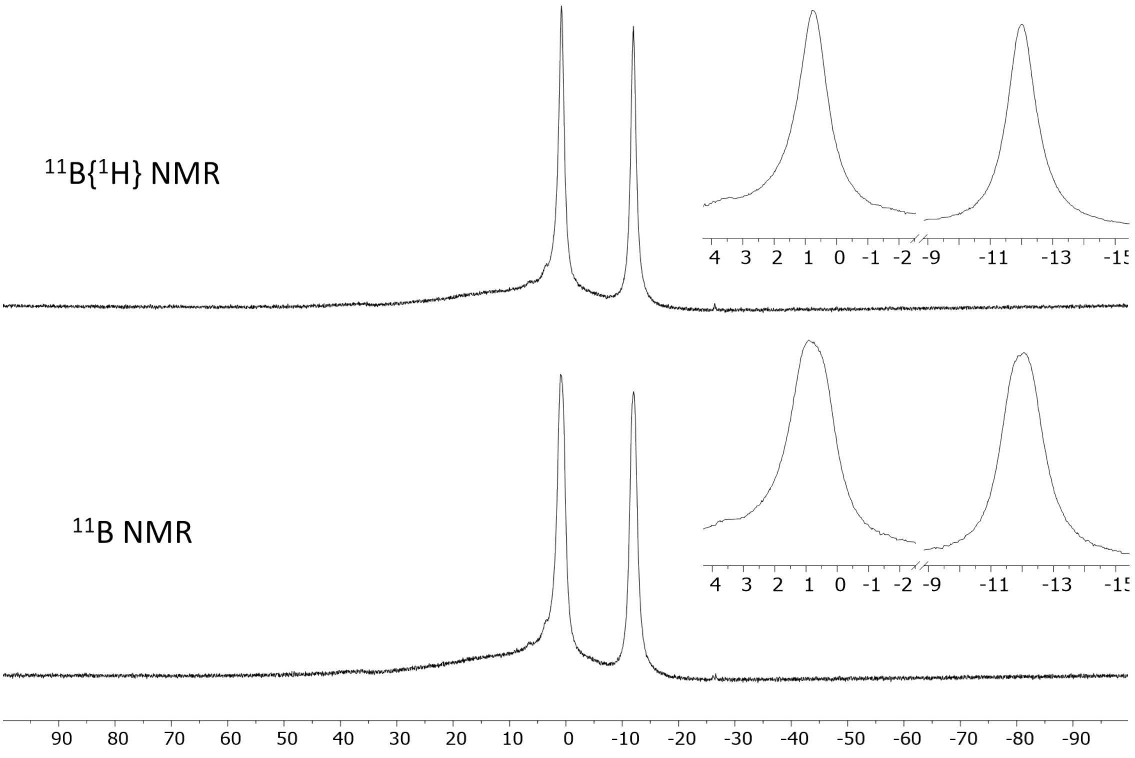

Figure S48: **^{11}B** and **^{11}B{^{1}H} NMR** (193 MHz, CD_2Cl_2, 293 K) spectra of compound **11a**.

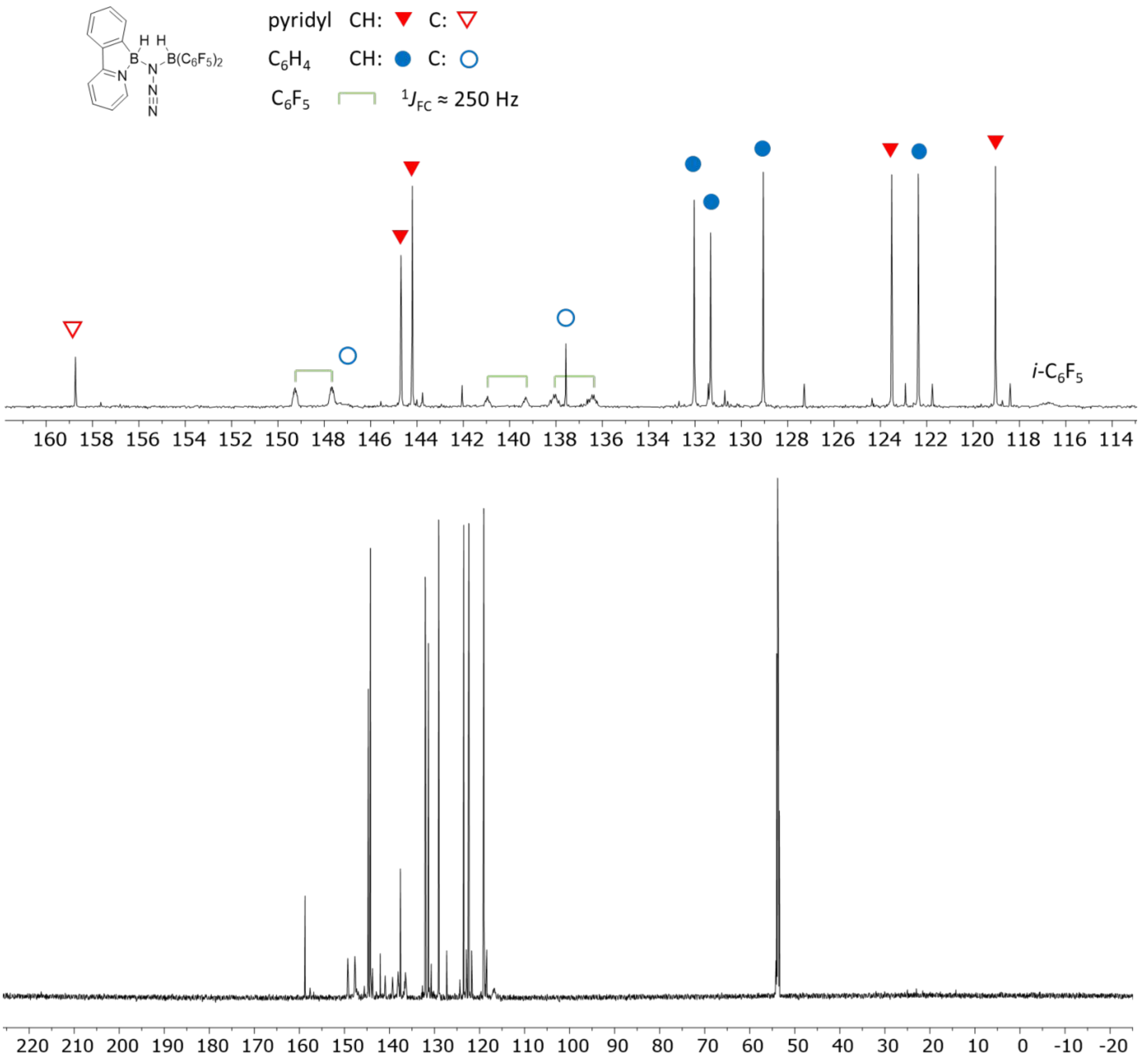

Figure S49: **^{13}C{^{1}H} NMR** (151 MHz, CD_2Cl_2, 293 K) spectrum of compound **11a**.

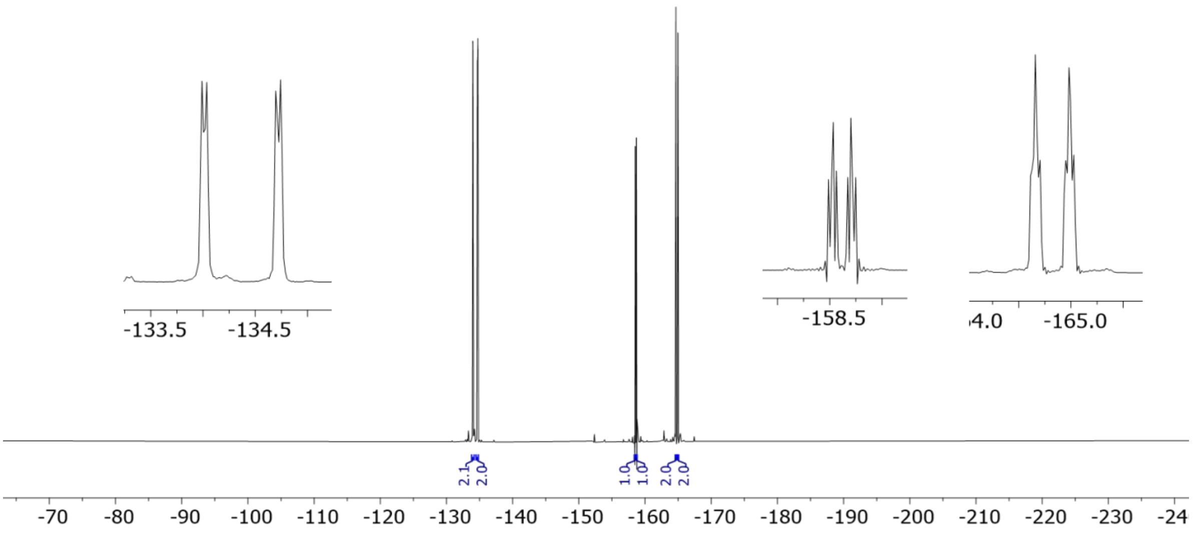

Figure S50: **^{19}F NMR** (CD_2Cl_2, 564 MHz, 293 K) spectrum of compound **11a**.

Crystal structure determination of **11a [KSK386-gg]**

A crystal suitable for SC-XRD structure determination was obtained by slow diffusion of hexane into a solution of **11a** in dichloromethane in argon filled glovebox.

Crystal Data for $C_{23}H_{10}B_2F_{10}N_4$ (M =553.97 g/mol): triclinic, space group P-1 (no. 2), a = 7.6334(6) Å, b = 11.6560(6) Å, c = 13.2648(7) Å, α = 102.241(4)°, β = 96.193(6)°, γ = 98.807(6)°, V = 1127.71(12) Å^3, Z = 2, T = 100.00(10) K, μ(Cu Kα) = 1.389 mm^{-1}, $Dcalc$ = 1.631 g/cm^3, 10681 reflections measured (6.892° ≤ 2Θ ≤ 133.202°), 3916 unique (R_{int} = 0.0434, R_{sigma} = 0.0425) which were used in all calculations. The final R_1 was 0.0574 (I > 2σ(I)) and wR_2 was 0.1726 (all data). **CCDC: 2445684**

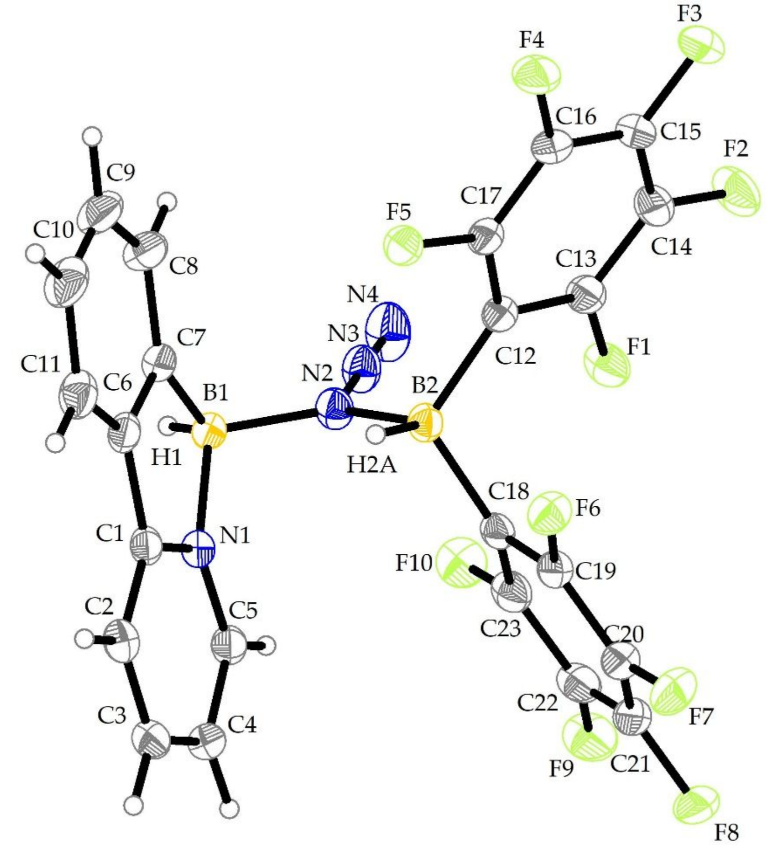

Figure S51: Crystal structure of **11a** (thermal ellipsoids shown at 30 % probability level).

Preparation of **12a**

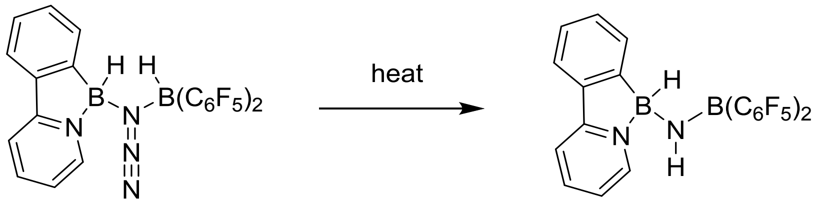

11a **12a**

Boron azide **3a** (20.8 mg, 0.100 mmol) and $HB(C_6F_5)_2$ (34.6 mg, 0.100 mmol) were combined in C_6D_6 (0.6 mL) and placed into J. Young NMR tube. The mixture was vigorously shaken and NMR spectra were recorded after 15 minutes revealing the formation of **11a**. The NMR tube was placed into an oil bath preheated to 100 °C. After 60 minutes, NMR spectra were recorded again to show a complete consumption of **11a** and formation of **12a** alongside with some impurities. Compound **12a** was characterized *in situ* as its workup and any attempt of isolation resulted only in deterioration of its purity. Yield was not determined.

^{1}H NMR (600 MHz, C_6D_6, 293 K): δ = [8.09 (d, $^{3}J_{HH}$ = 5.6 Hz), 7.93 ($^{3}J_{HH}$ = 7.3 Hz), 7.30 (dd, $^{3}J_{HH} \approx$ $^{3}J_{HH}$ = 7.5 Hz), 7.26 ($^{3}J_{HH}$ = 7.7 Hz), 7.12 (dd, $^{3}J_{HH} \approx$ $^{3}J_{HH}$ = 7.5 Hz), 6.80 (m), 6.79 (m), 6.24 (dd, $^{3}J_{HH}$ = 7.5 Hz, $^{3}J_{HH}$ = 5.6 Hz)](each 1H, CH^{Ar}), 5.13 (s, 1H, NH), 4.01 (br, 1H, BH).

^{11}B{^{1}H} NMR (193 MHz, C_6D_6, 293 K): δ = 35.7 ($\nu_{1/2} \approx$ 500 Hz, N-$B(C_6F_5)_2$), –1.5 ($\nu_{1/2} \approx$ 180 Hz, BH).

^{11}B NMR (193 MHz, C_6D_6, 293 K): δ = 35.7 ($\nu_{1/2} \approx$ 500 Hz, N-$B(C_6F_5)_2$), –1.5 ($\nu_{1/2} \approx$ 280 Hz, BH).

^{19}F NMR (564 MHz, C_6D_6, 293 K): δ = [–132.9, –133.5](each m, 2F, *o*-C_6F_5), [–153.2, –155.4](each m, 1F, *p*-C_6F_5), [–162.4, –162.9](each m, 2F, *m*-C_6F_5).

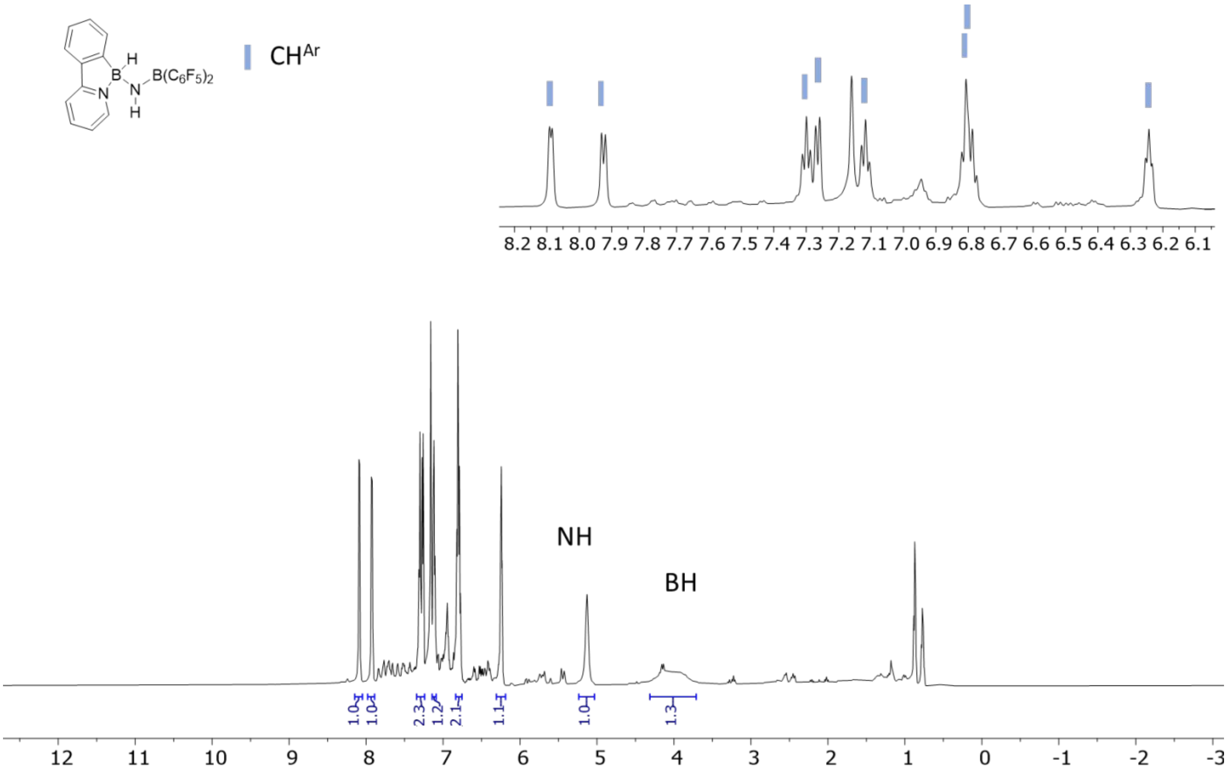

Figure S52: **^{1}H NMR** (C_6D_6, 600 MHz, 293 K) spectrum recorded for the *in situ* generated compound **12a**.

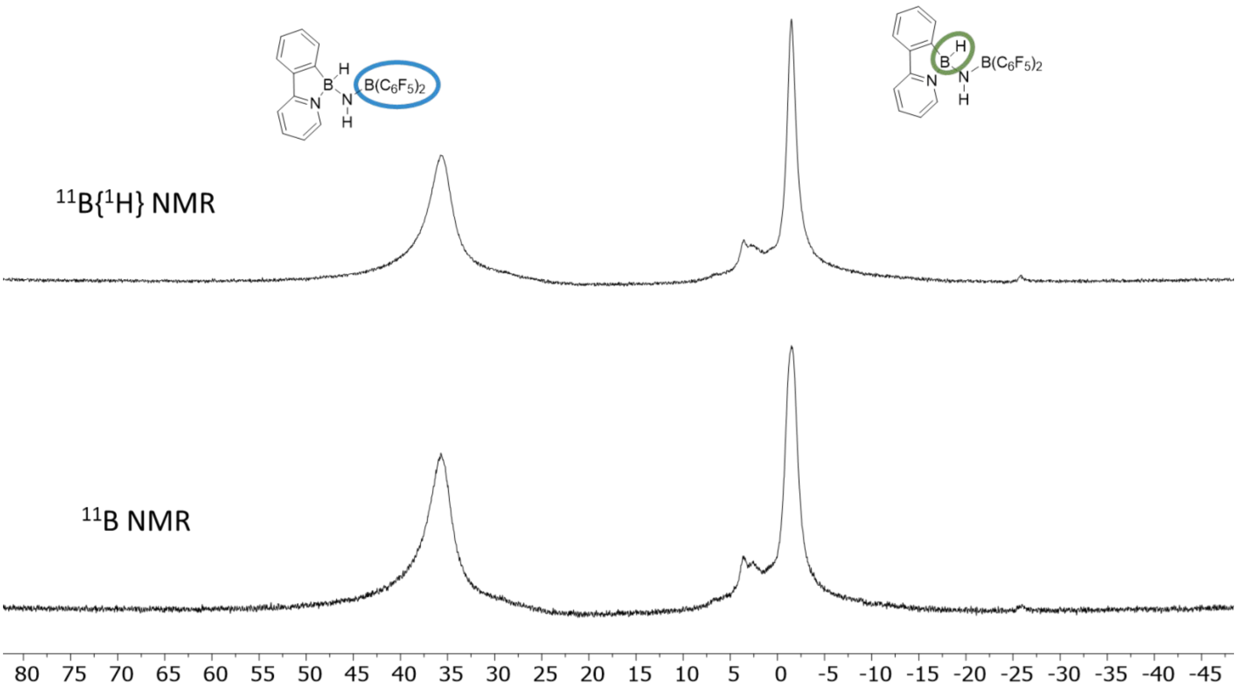

Figure S53: **^{11}B** and **^{11}B{^{1}H} NMR** (193 MHz, C_6D_6, 293 K) spectra recorded for the *in situ* generated compound **12a**.

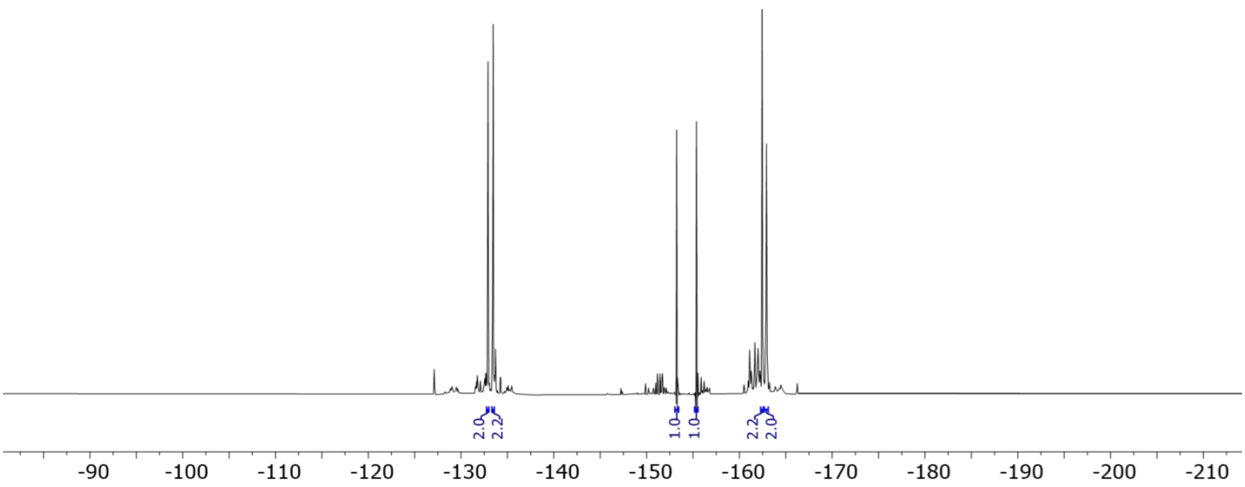

Figure S54: [19]**F NMR** (564 MHz, C_6D_6, 293 K) spectrum recorded for the *in situ* generated compound **12a**.

Preparation of **12c**

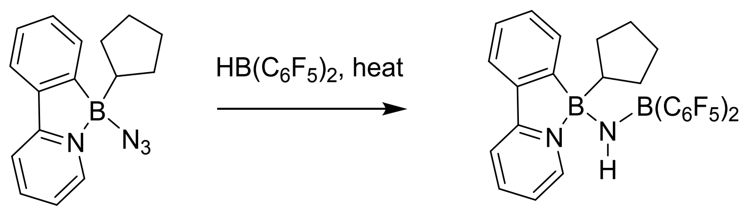

3c **12c**

Boron azide **3c** (138 mg, 0.500 mmol) and $HB(C_6F_5)_2$ (173 mg, 0.500 mmol) were dissolved in toluene (5 mL) and heated to 100 °C for one hour. After cooling to room temperature, the mixture was filtered via cannula, and volatiles removed *in vacuo*, yielding thick yellow oil. Hexane (5 mL) was added and the resulting emulsion was vigorously stirred overnight to precipitate the impurities. On the following day, the mixture was carefully decanted and the solid residue extracted with hexane (2x 3 mL). Organic fractions were combined and volatiles removed *in vacuo*, providing compound **12c** as a white solid (246 mg, 0.415 mmol, 83% yield).

Elemental analysis calculated for $C_{28}H_{18}N_2B_2F_{10}$ (594.1): C 56.61, H 3.05, N 4.47; found C 56.29, H 3.02, N 4.12.

IR (KBr): 3380 (m, N-H), 3070 (w), 2950 (m), 2910 (m), 2864 (m), 1647 (s), 1621 (s), 1572 (m), 1479 (br s), 1400 (s), 1303 (s), 1252 (m), 1175 (s), 1129 (s), 1088 (s), 1064 (m), 977 (s), 918 (m), 856 (m), 795 (s), 762 (s), 742 (s), 694 (m), 636 (m), 557 (w), 543 (w), 422 (w) cm^{-1}.

^{1}H NMR (600 MHz, CD_2Cl_2, 293 K): δ = [8.46 (d, $^3J_{HH}$ = 5.7 Hz), 8.08 (dd, $^3J_{HH} \approx {}^3J_{HH} \approx$ 7.8 Hz), 7.81 (d, $^3J_{HH}$ = 7.9 Hz), 7.47 (dd, $^3J_{HH}$ = 7.9 Hz, $^3J_{HH}$ = 5.7 Hz)](each 1H, pyridyl), [7.55 (m), 7.55 (m), 7.31 (dd, $^3J_{HH} \approx {}^3J_{HH} \approx$ 7.6 Hz), 7.20 (dd, $^3J_{HH} \approx {}^3J_{HH} \approx$ 7.6 Hz)](each 1H, C_6H_4), 5.98 (br, 1H, NH), [1.81, 1.49, 1.47, 1.42, 1.30, 1.29, 1.08, 1.08, 0.51](each m, 1H, cyclopentyl).

^{13}C{^{1}H} NMR (151 MHz, CD_2Cl_2, 293 K): δ = [157.6 (br, C-B), 136.4 (*i*-C), 130.8 (CH), 130.6 (CH), 126.7 (CH), 121.0 (CH)](C_6H_4), [156.7 (*i*-C), 143.0 (CH), 141.7 (CH), 122.9 (CH), 117.7 (CH)](pyridyl), [148.4, 145.0, 141.9, 140.1, 137.5, 136.3](each dm, $^1J_{FC} \approx$ 250 Hz, C-F of C_6F_5; *i*-C of C_6F_5 cannot be unambiguously assigned), [36.6 (br), 29.1, 28.6, 27.4, 26.8](cyclopentyl).

^{11}B{^{1}H} NMR (193 MHz, CD_2Cl_2, 293 K): δ = 34.5 ($\nu_{1/2} \approx$ 400 Hz, N-B(C_6F_5)$_2$), 2.9 ($\nu_{1/2} \approx$ 180 Hz, Phpy-B(cypent)).

^{11}B NMR (193 MHz, CD_2Cl_2, 293 K): δ = 34.5 ($\nu_{1/2} \approx$ 400 Hz, N-B(C_6F_5)$_2$), 2.9 ($\nu_{1/2} \approx$ 180 Hz, Phpy-B(cypent)).

^{19}F NMR (564 MHz, CD_2Cl_2, 293 K): δ = [–130.0 (1F), –133.8 (2F), –135.0 (1F)](each m, *o*-C_6F_5), [–153.7, –157.5](each m, 1F, *p*-C_6F_5), [–163.3 (2F), –164.0 (1F), –165.2(1F)](each m, *m*-C_6F_5).

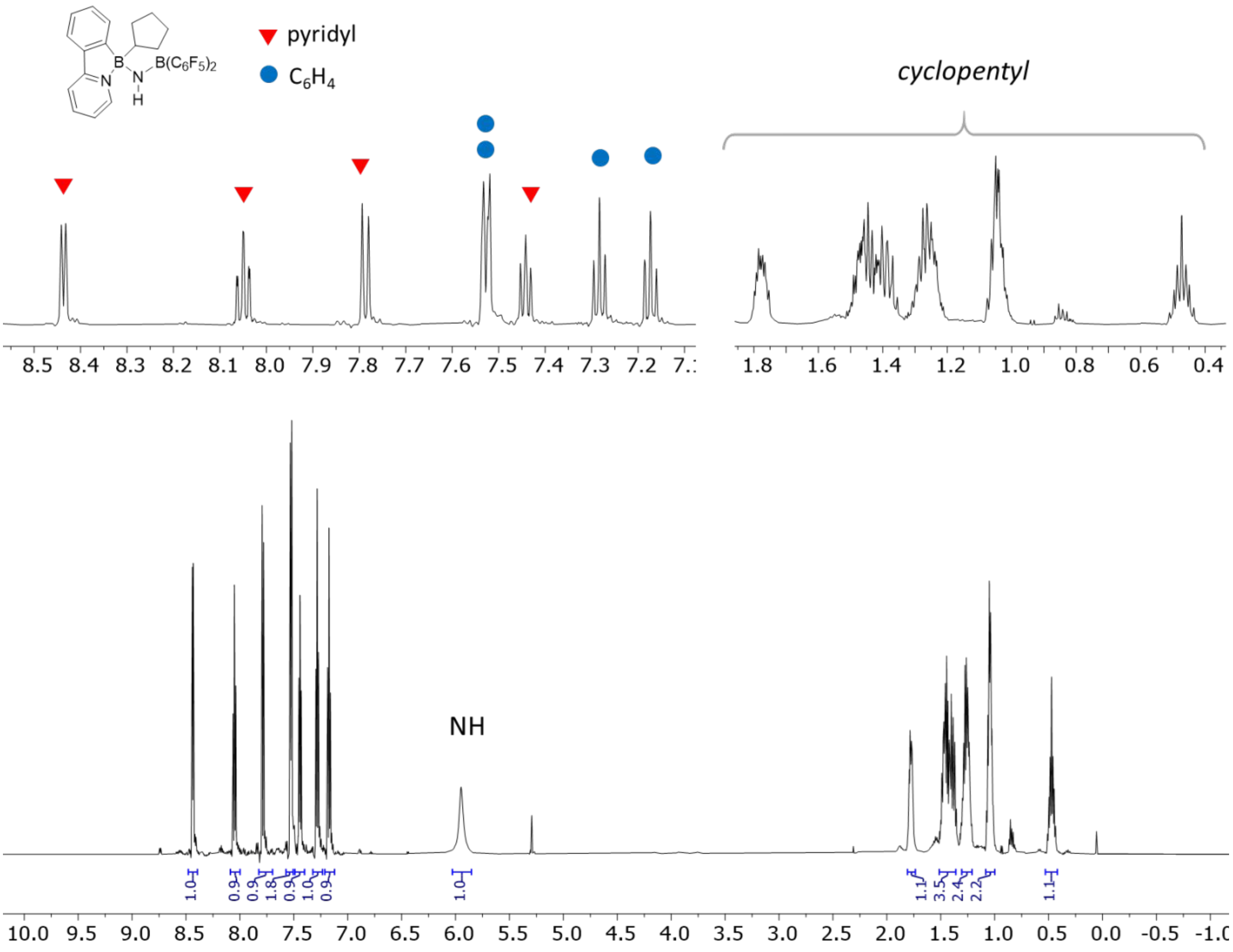

Figure S55: **^{1}H NMR** (CD_2Cl_2, 600 MHz, 293 K) spectrum of compound **12c**.

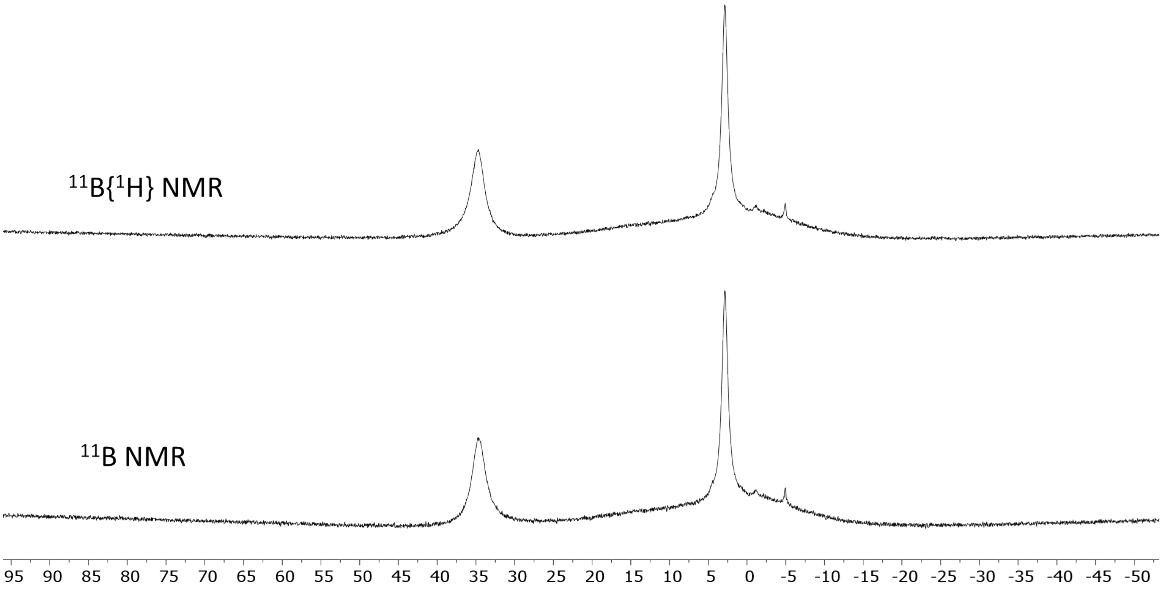

Figure S561: **^{11}B** and **^{11}B{^{1}H} NMR** (193 MHz, CD_2Cl_2, 293 K) spectra of compound **12c**.

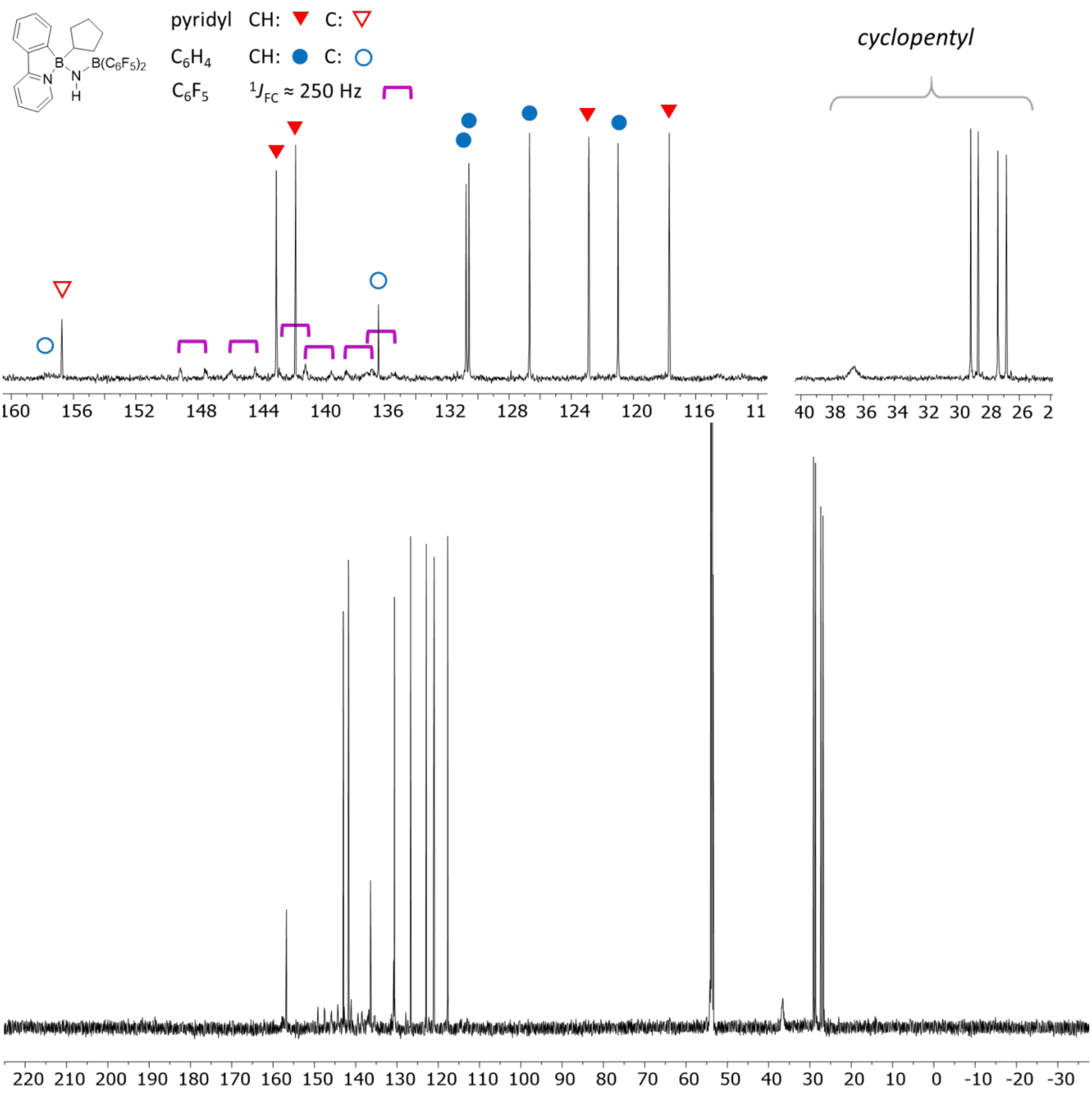

Figure S57: **$^{13}C\{^1H\}$ NMR** (151 MHz, CD_2Cl_2, 293 K) spectrum of compound **12c**.

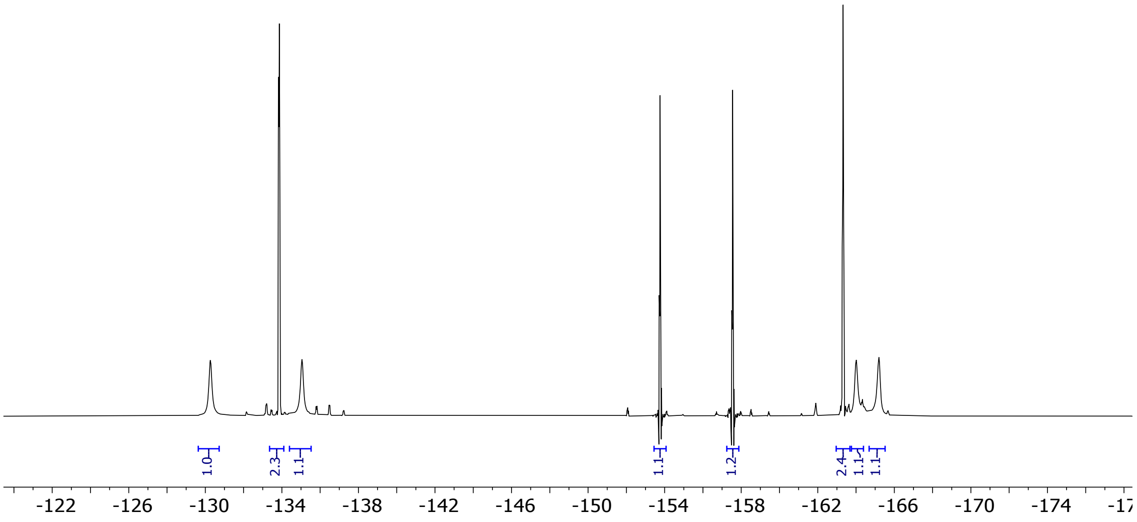

Figure S58: **^{19}F NMR** (CD_2Cl_2, 564 MHz, 293 K) spectrum of compound **12c**.

Preparation of **13a**

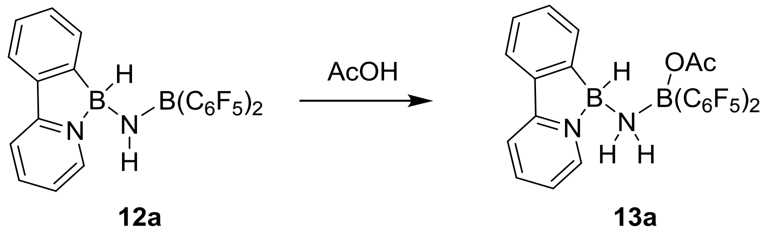

Diboron amine **12a** (52.6 mg, 0.100 mmol) was dissolved in dichloromethane (3 mL) and treated with excess of acetic acid (0.1 mL). The bright yellow reaction mixture was allowed to stir for 30 minutes at room temperature. The mixture was then exposed to ambient atmosphere, volatiles were removed *in vacuo* and the yellow residue purified by short column chromatography on silica gel using ethyl acetate / hexane (1:1) as mobile phase. Yellow band containing impurities was eluted first, followed by the band corresponding to the product. Volatiles were removed *in vacuo* to give compound **13a** as a white solid (28.6 mg, 0.0488 mmol, 49% yield).

Compound **13a** gradually decomposes both in solution and solid state, satisfactory elementary analysis was not obtained.

HRMS calculated for anion $C_{25}H_{13}N_2B_2F_{10}O_2^-$ [M-H]$^-$: 585.1012, found 585.1014.

^{1}H NMR (600 MHz, $CDCl_3$, 293 K): δ = [8.61 (d, $^3J_{HH}$ = 5.7 Hz), 8.14 (dd, $^3J_{HH} \approx {}^3J_{HH} \approx$ 8.0 Hz), 7.97 (d, $^3J_{HH}$ = 8.1 Hz), 7.44 (dd, $^3J_{HH}$ = 7.9 Hz, $^3J_{HH}$ = 5.7)](each 1H, pyridyl), [7.75 (d, $^3J_{HH}$ = 7.4 Hz), 7.38 (m), 7.38 (m), 7.37 (m)](each 1H, C_6H_4), [4.71 (br, 1H), 4.63 (br, 1H)](NH_2), 3.55 (br, 1H, BH), 1.94 (s, 3H, OAc).

^{13}C{^{1}H} NMR (151 MHz, $CDCl_3$, 293 K): δ = [175.6 (CO), 23.4 (CH_3)](OAc), [159.0 (*i*-C), 144.3 (CH), 142.8 (CH), 122.3 (CH), 118.5 (CH)](pyridyl), [151.6 (br, C-B)[a], 136.5 (*i*-C), 131.7 (CH), 130.8 (CH), 128.0 (CH), 121.8 (CH)](C_6H_4), [148.5 (dm, $^1J_{FC} \approx$ 240 Hz), 140.1 (dm, $^1J_{FC} \approx$ 250 Hz), 137.2 (dm, $^1J_{FC} \approx$ 250 Hz), 116 (br m, C-B)](C_6F_5), [a]based on gHMBC experiment, signals of $B(C_6F_5)_2$ are not listed.

^{11}B{^{1}H} NMR (193 MHz, $CDCl_3$, 293 K): δ = –1.2 ($\nu_{1/2} \approx$ 80 Hz), –2.6 ($\nu_{1/2} \approx$ 200 Hz).

^{11}B NMR (193 MHz, $CDCl_3$, 293 K): δ = –1.2 ($\nu_{1/2} \approx$ 80 Hz), –2.6 (d, $^1J_{BH} \approx$ 100 Hz).

^{19}F NMR (564 MHz, $CDCl_3$, 293 K): δ = –135.7 (m, 2F, *o*-C_6F_5), –157.5 (m, 1F, *o*-C_6F_5), –163.5 (m, 2F, *m*-C_6F_5).

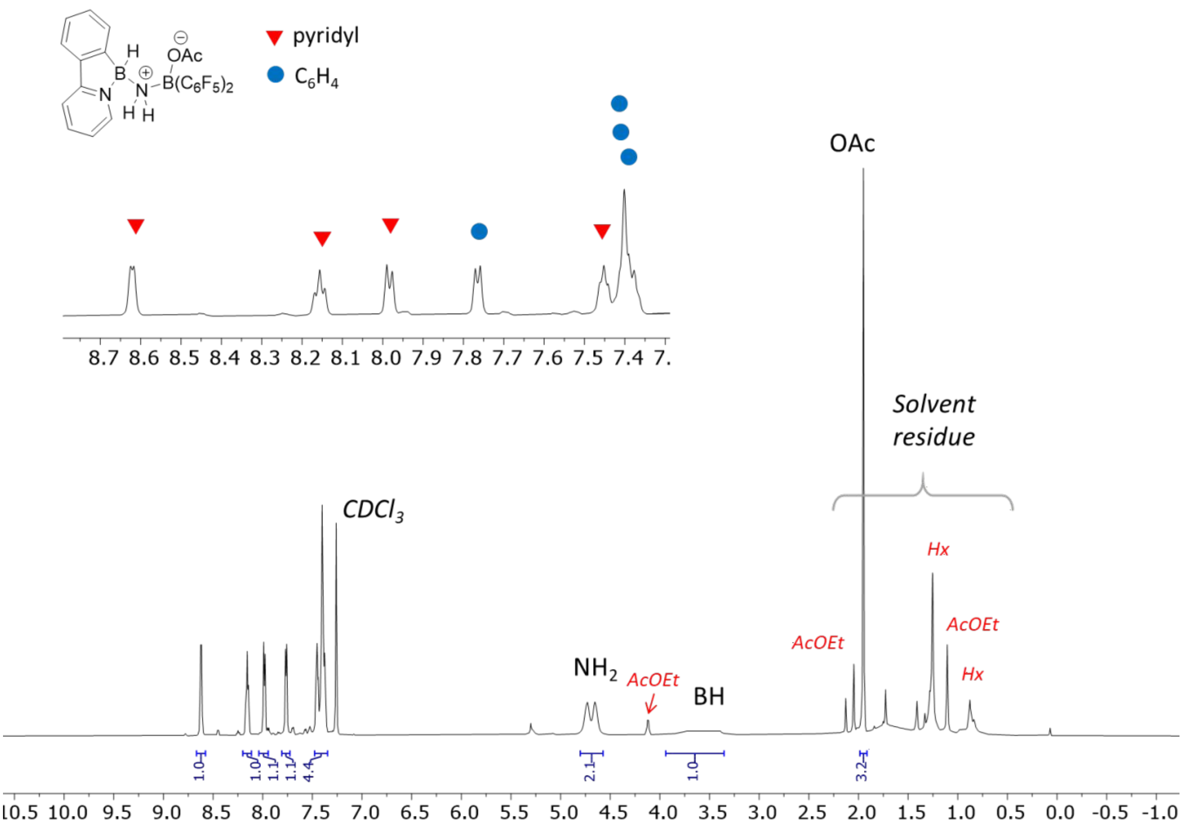

Figure S59: **^{1}H NMR** ($CDCl_3$, 600 MHz, 293 K) spectrum of compound **13a**, Hx and AcOEt denotes solvent residues.

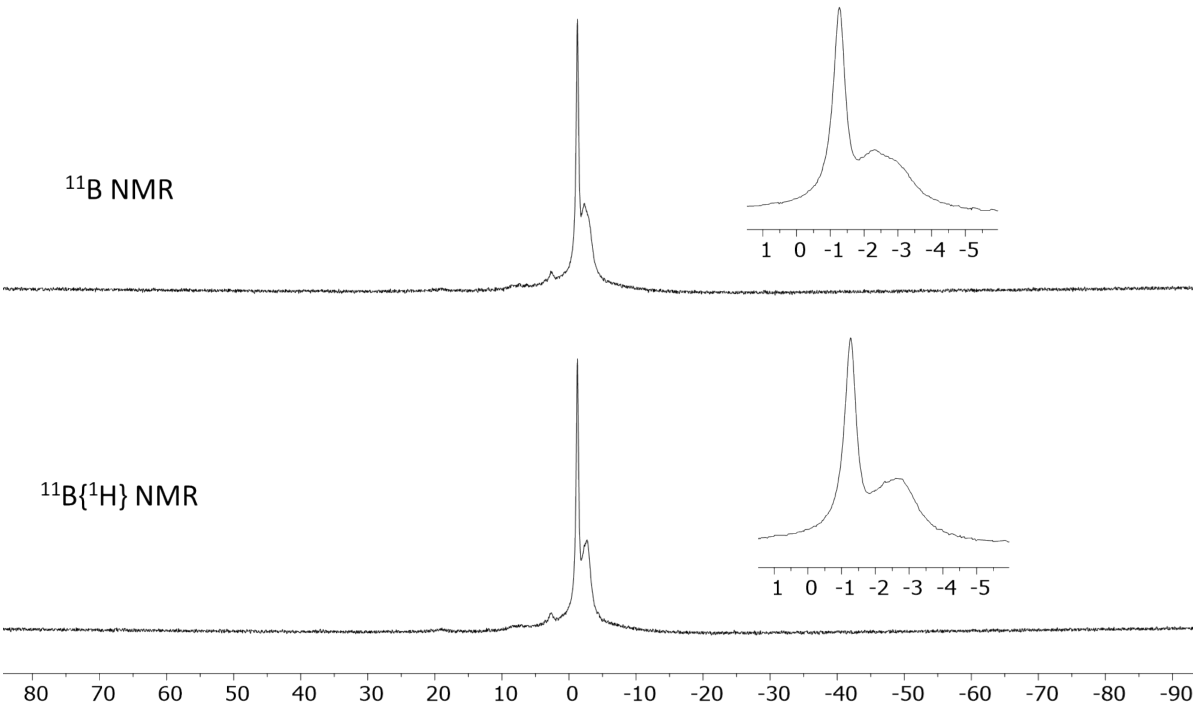

Figure S60: **^{11}B** and **^{11}B{^{1}H} NMR** (193 MHz, $CDCl_3$, 293 K) spectra of compound **13a**.

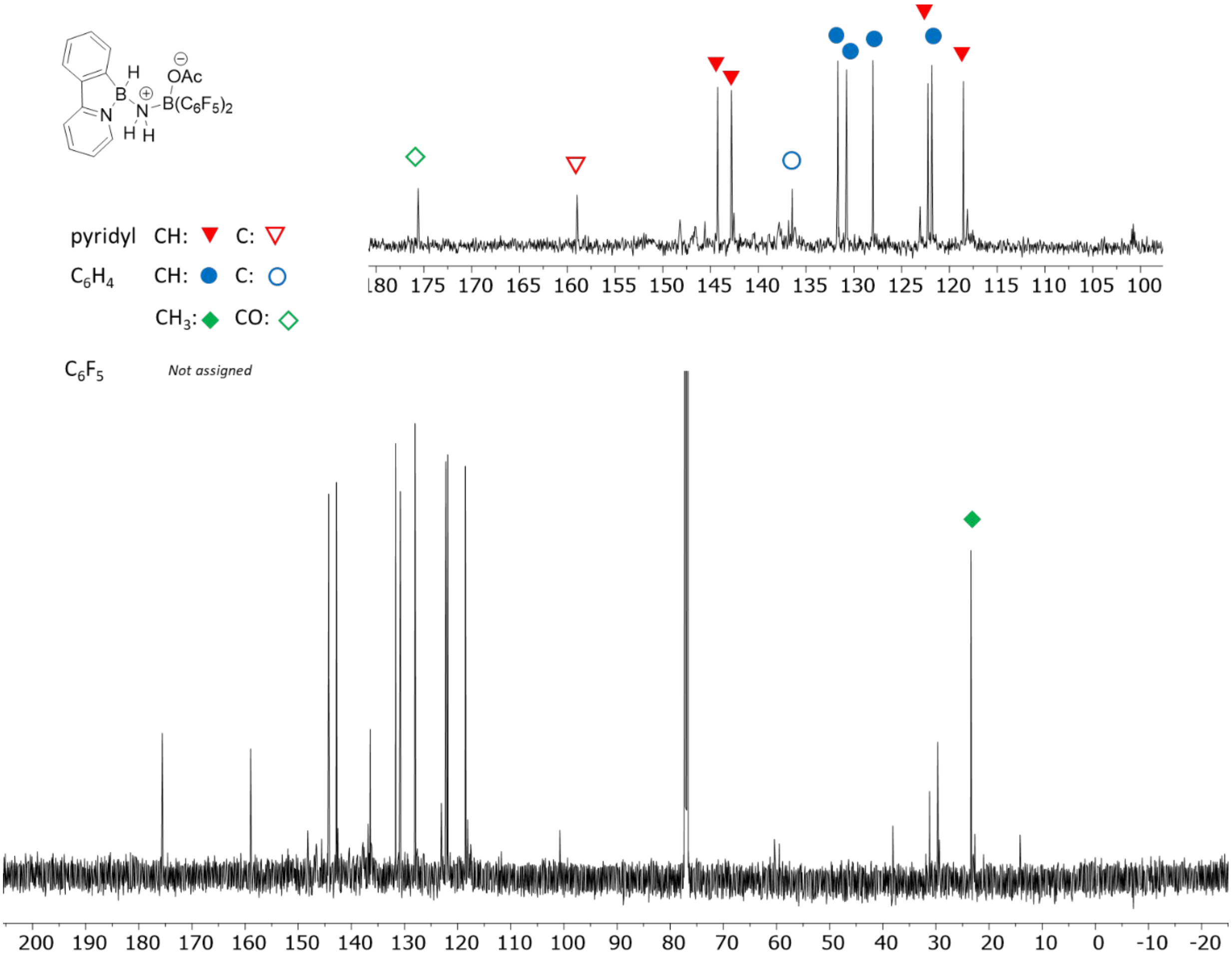

Figure S61: $^{13}C\{^{1}H\}$ **NMR** (151 MHz, $CDCl_3$, 293 K) spectrum of compound **13a**.

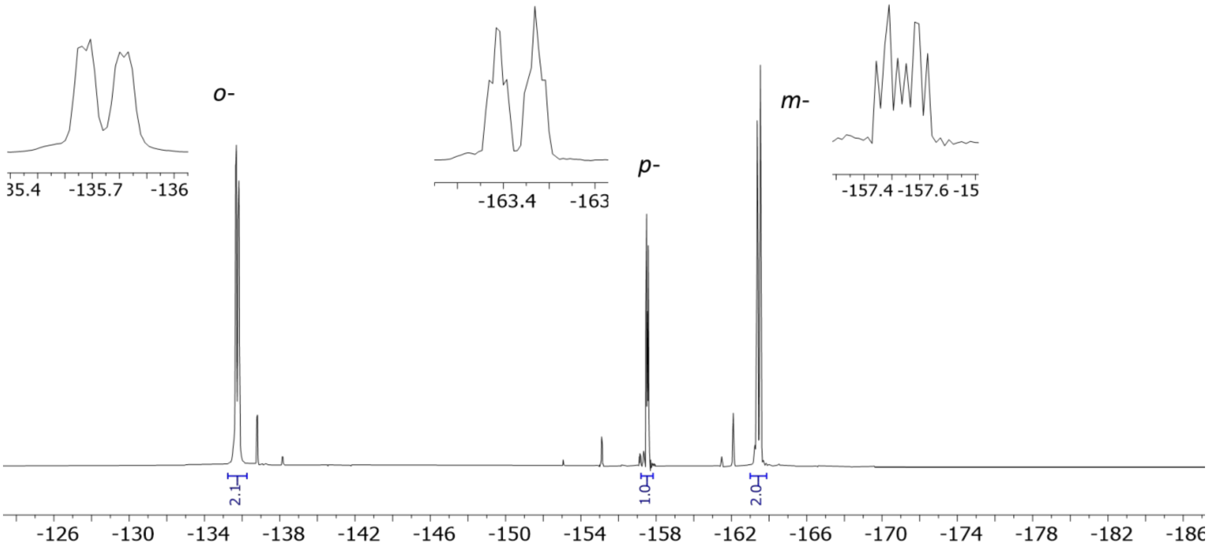

Figure S62: ^{19}F **NMR** ($CDCl_3$, 564 MHz, 293 K) spectrum of compound **13a**.

Preparation of **13c**

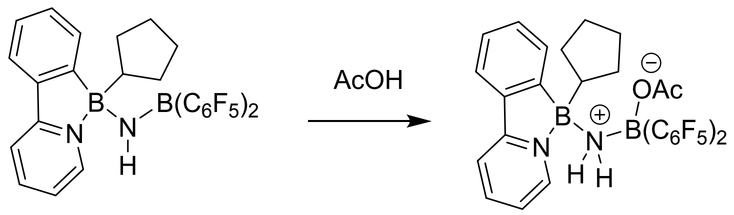

12c **13c**

Diboron amine **12c** (59.4 mg, 0.100 mmol) was dissolved in dichloromethane (3 mL) and treated with excess of acetic acid (0.1 mL). The bright yellow reaction mixture was allowed to stir for 30 minutes at room temperature. The mixture was then exposed to ambient atmosphere, volatiles were removed *in vacuo* and the yellow oily residue was purified by short column chromatography on silica gel using ethylacetate / hexane (1:3) as mobile phase. The yellow band containing impurities was eluted first, followed by the band corresponding to the product. Volatiles were removed *in vacuo*, yielding compound **13c** as a rapidly crystallizing white solid (54.6 mg, 0.0834 mmol, 83% yield).

HRMS calculated for anion $C_{30}H_{21}N_2B_2F_{10}O_2^-$ $[M\text{-}H]^-$: 653.1640, found 653.1645.

Elemental analysis calculated for $C_{30}H_{22}N_2B_2F_{10}O_2$ (654.1): C 55.09, H 3.39, N 4.28; found C 55.45, H 3.46, N 4.65.

IR (KBr): 3314 (w, N-H), 3207 (m, N-H), 2953 (m), 2936 (m), 2866 (w), 2816 (w), 1686 (CO, s), 1646 (m), 1625 (m), 1560 (m), 1515 (s), 1486 (m), 1468 (s), 1450 (s), 1375 (s), 1304 (s), 1277 (s), 1262 (s), 1205 (m), 1169 (m), 1161 (m), 1110 (w), 1090 (s), 1029 (m), 984 (m), 963 (m), 931 (m), 908 (m), 892 (m), 818 (w), 789 (m), 760 (s), 734 (s), 686 (m), 637 (w), 629 (w), 614 (m), 556 (m), 537 (m), 521 (m), 474 (w), 431 (m) cm^{-1}.

^{1}H NMR (600 MHz, $CDCl_3$, 293 K): δ = [8.39 (d, $^3J_{HH}$ = 5.9 Hz), 8.08 (dd, $^3J_{HH} \approx {}^3J_{HH} \approx$ 7.7 Hz), 7.88 (d, $^3J_{HH}$ = 7.9 Hz), 7.43 (dd, $^3J_{HH}$ = 7.7 Hz, $^3J_{HH}$ = 5.9 Hz)](each 1H, pyridyl), [7.61 (d, $^3J_{HH}$ = 7.6 Hz), 7.48 (d, $^3J_{HH}$ = 7.6 Hz), 7.32 (dd, $^3J_{HH} \approx {}^3J_{HH} \approx$ 7.6 Hz), 7.28 (dd, $^3J_{HH} \approx {}^3J_{HH} \approx$ 7.6 Hz)](each 1H, C_6H_4), [5.10, 4.43](each br, H, NH), 1.76 (s, 3H, OAc), [1.93, 1.57, 1.56, 1.48, 1.31, 1.30, 1.21, 1.02, 0.37, 0.51](each m, 1H, cyclopentyl).

^{13}C{^{1}H} NMR (151 MHz, $CDCl_3$, 293 K): δ = [175.7 (CO), 23.7 (CH_3)](OAc), [158.5 (*i*-C), 143.1 (CH), 142.6 (CH), 121.9 (CH), 118.2 (CH)](pyridyl), [157.6 (br, C-B)[a], 136.4 (*i*-C), 131.2 (CH), 130.0 (CH), 127.6 (CH), 121.4 (CH)](C_6H_4), [36.6 (br), 29.1, 28.6, 27.4, 26.8](cyclopentyl), signals of C_6F_5 cannot be unambiguously assigned), [a]based on gHMBC.

^{11}B{^{1}H} NMR (193 MHz, $CDCl_3$, 293 K): δ = 2.6 ($\nu_{1/2} \approx$ 400 Hz), –1.6 ($\nu_{1/2} \approx$ 75 Hz).

^{11}B NMR (193 MHz, $CDCl_3$, 293 K): δ = 2.6 ($\nu_{1/2} \approx$ 400 Hz), –1.6 ($\nu_{1/2} \approx$ 75 Hz).

^{19}F NMR (564 MHz, $CDCl_3$, 293 K): δ = –135.7 (br, 4F, *o*-C_6F_5), [–153.8, –158.3](each m, 1F, *p*-C_6F_5), [–163.6, –164.1](each m, 2F, *m*-C_6F_5).

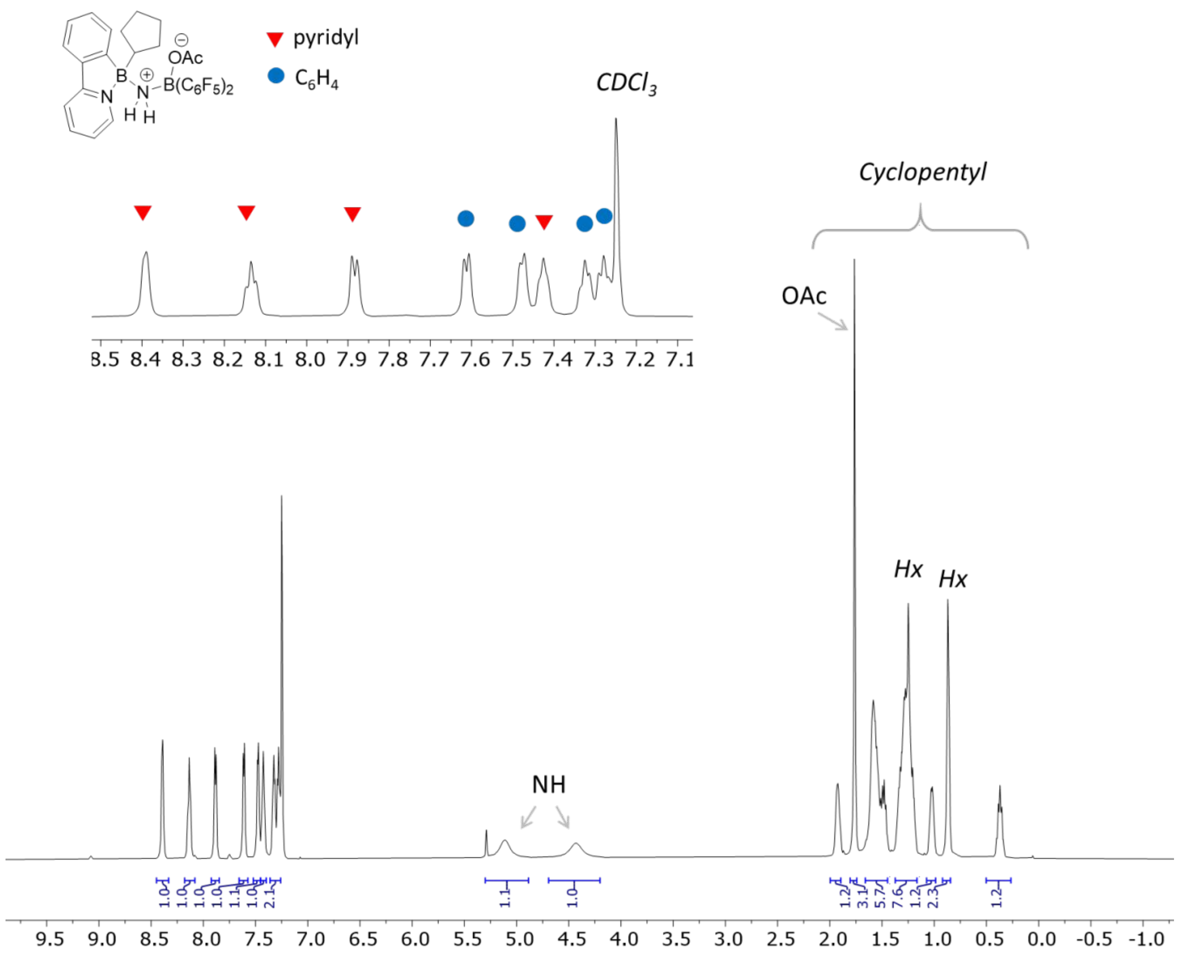

Figure S63: **^{1}H NMR** ($CDCl_3$, 600 MHz, 293 K) spectrum of compound **13c**. Hx denotes residual hexane.

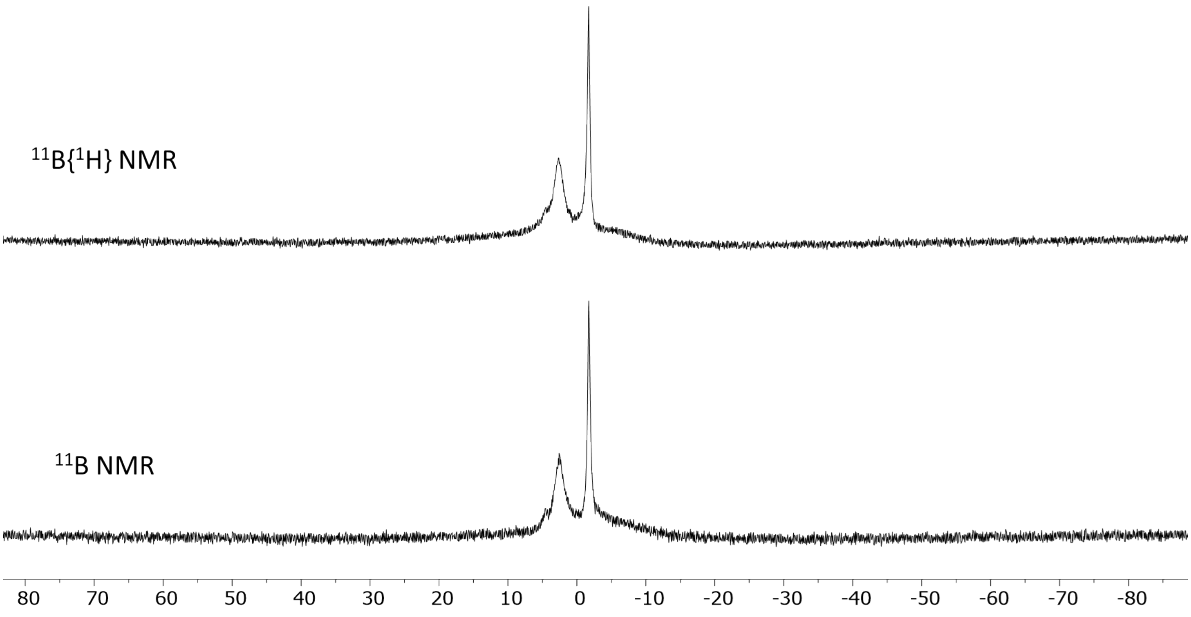

Figure S64: **^{11}B** and **^{11}B{^{1}H} NMR** (193 MHz, $CDCl_3$, 293 K) spectra of compound **13c**.

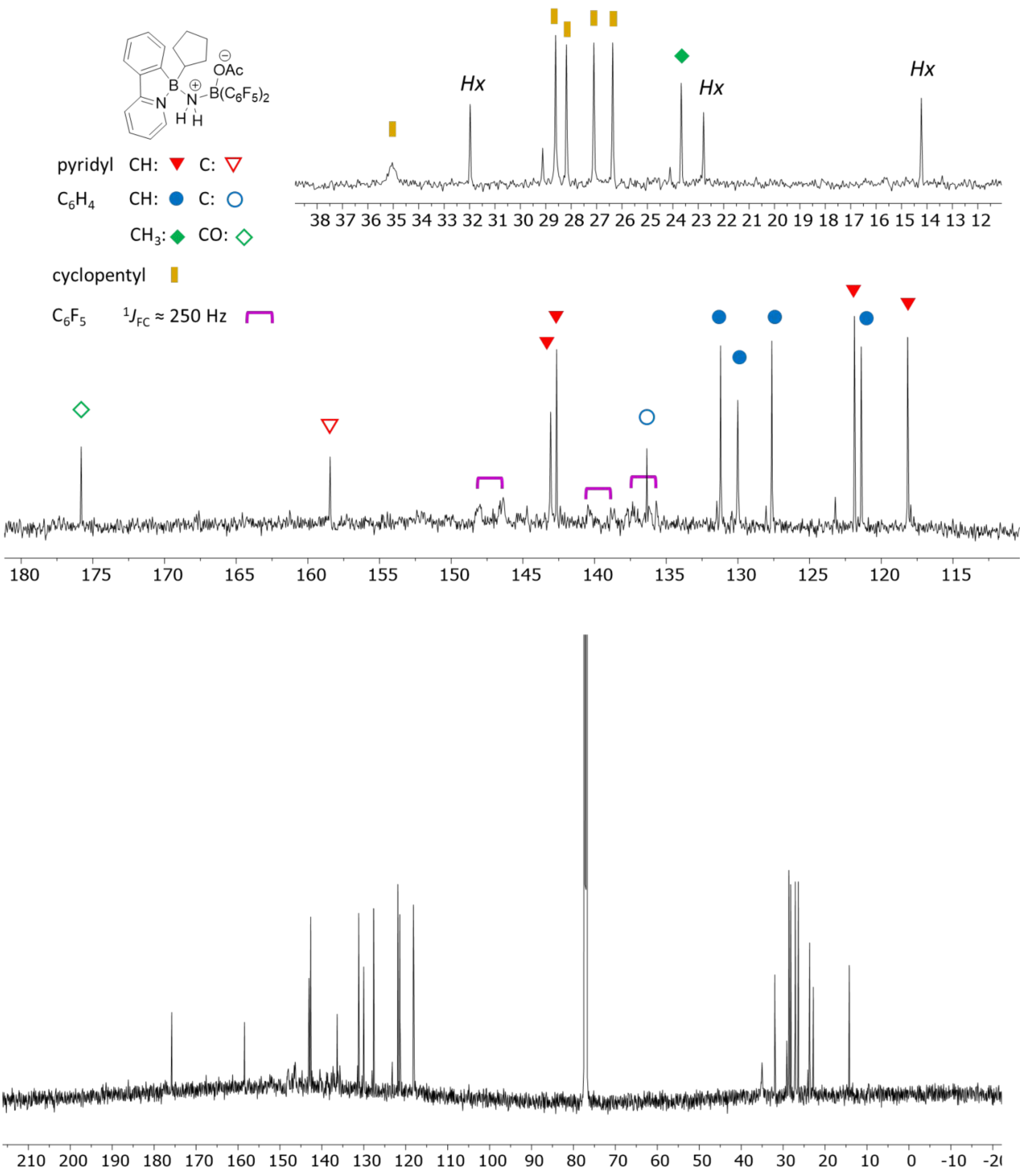

Figure S65: **^{13}C{^{1}H} NMR** (151 MHz, $CDCl_3$, 293 K) spectrum of compound **13c**.

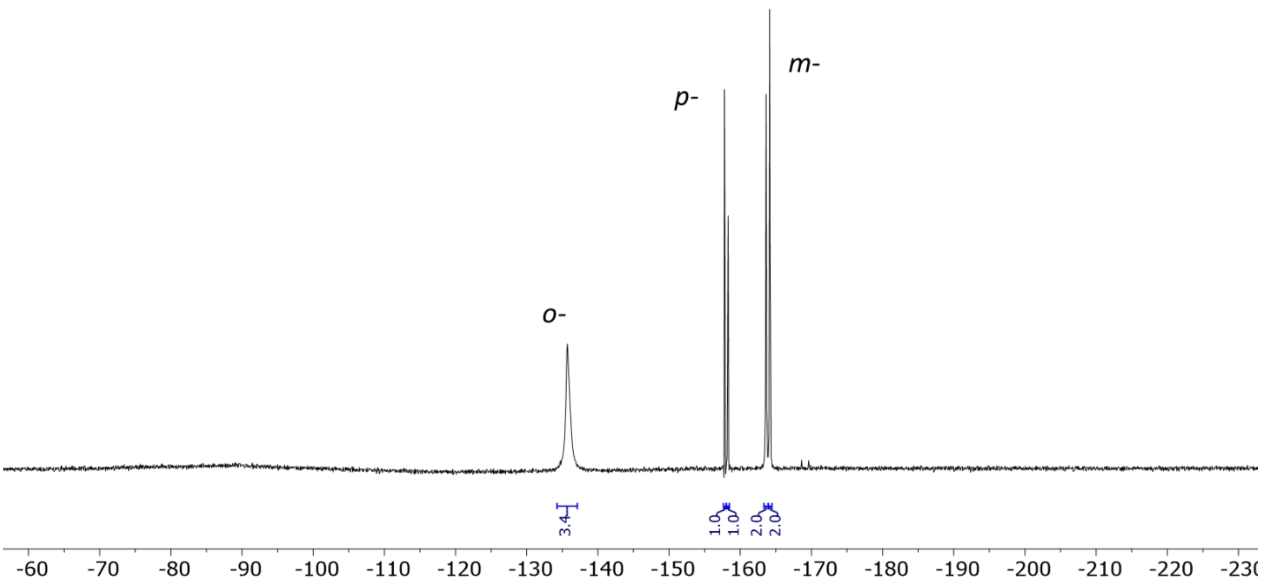

Figure S66: **^{19}F NMR** ($CDCl_3$, 564 MHz, 293 K) spectrum of compound **13c**.

Crystal structure determination of **13c** [KSK475-gg]

A crystal suitable for SC-XRD structure determination was obtained by slow evaporation of a solution of **13c** in a dichloromethane:heptane mixture (ca 1:1) under ambient conditions.

Crystal Data for $C_{30}H_{22}B_2F_{10}N_2O_2$ (M =654.11 g/mol): triclinic, space group P-1 (no. 2), a = 10.26571(19) Å, b = 10.7516(2) Å, c = 14.95180(14) Å, α = 87.1421(11)°, β = 84.7058(12)°, γ = 69.8797(17)°, V = 1542.66(5) Å^3, Z = 2, T = 99.99(10) K, μ(Cu Kα) = 1.132 mm^{-1}, $Dcalc$ = 1.408 g/cm^3, 45357 reflections measured (5.938° ≤ 2Θ ≤ 154.476°), 6157 unique (R_{int} = 0.0338, R_{sigma} = 0.0169) which were used in all calculations. The final R_1 was 0.0335 (I > 2σ(I)) and wR_2 was 0.0932 (all data). **CCDC: 2445690**

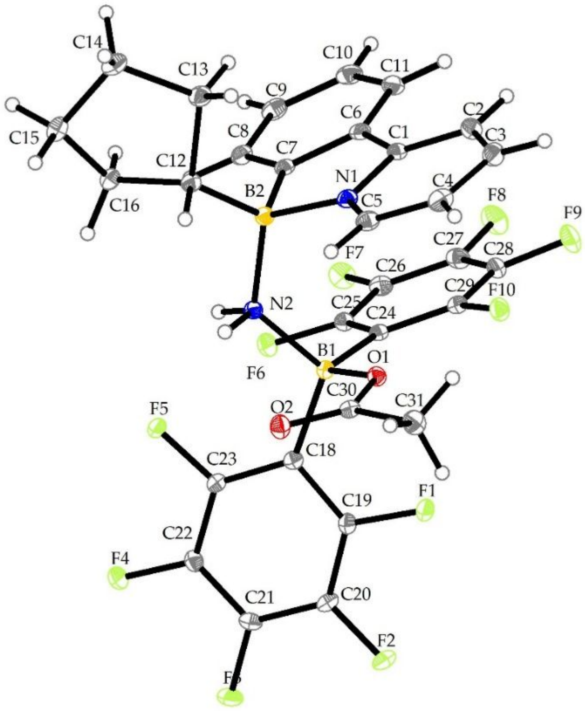

Figure S67: Crystal structure of **13c** (thermal ellipsoids shown at 30 % probability level).

Preparation of compound **8c** by hydrolysis of **12c**

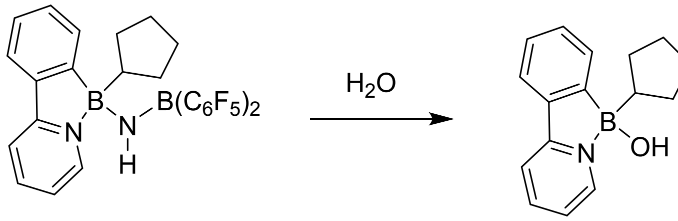

12c **8c**

Diboron amine **12c** (29.7 mg, 0.0500 mmol) was dissolved in tetrahydrofuran and two drops of distilled water were added. The mixture was stirred for 30 minutes at room temperature upon which $MgSO_4$ (ca 200 mg) was added, the mixture was filtered and filter paper was extensively washed with tetrahydrofuran. Volatiles were removed *in vacuo* to give colourless oil. NMR analysis of the crude mixture revealed a nearly complete conversion to borinic acid **8c**. The mixture was passed through a short silica gel column using dichloromethane / methanol 10:1 mixture as eluent to provide borinic acid **8c** in the form of a colourless oil (11.2 mg, 0.0446 mmol, 89% yield.)

NMR characterization data correspond to those obtained for **8c** prepared by reduction using the $NiCl_2/NaBH_4$ system (see above).

Reaction of **12c** with triflimic acid

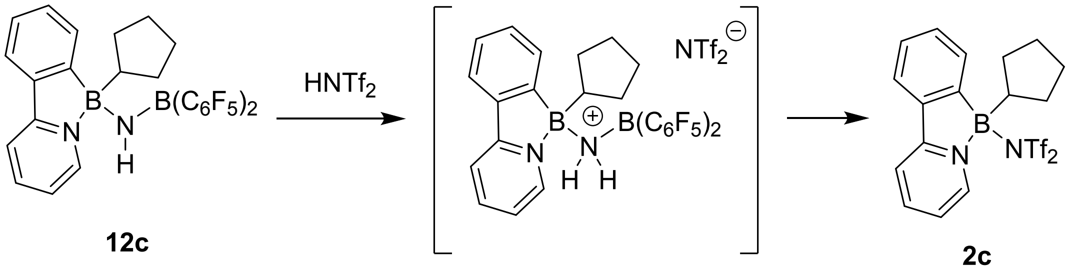

Diboron amine **12c** (29.7 mg, 0.0500 mmol) and triflimic acid (14.1 mg, 0.0500 mmol) were combined in CD_2Cl_2 (0.6 mL) and NMR spectra were recorded after 10 minutes and then one hour (see below). The NMR measurement likely indicated protonation of the amino group, followed by formation of compound **2c**. The identity of this product was confirmed by comparison with an authentic sample of **2c**.

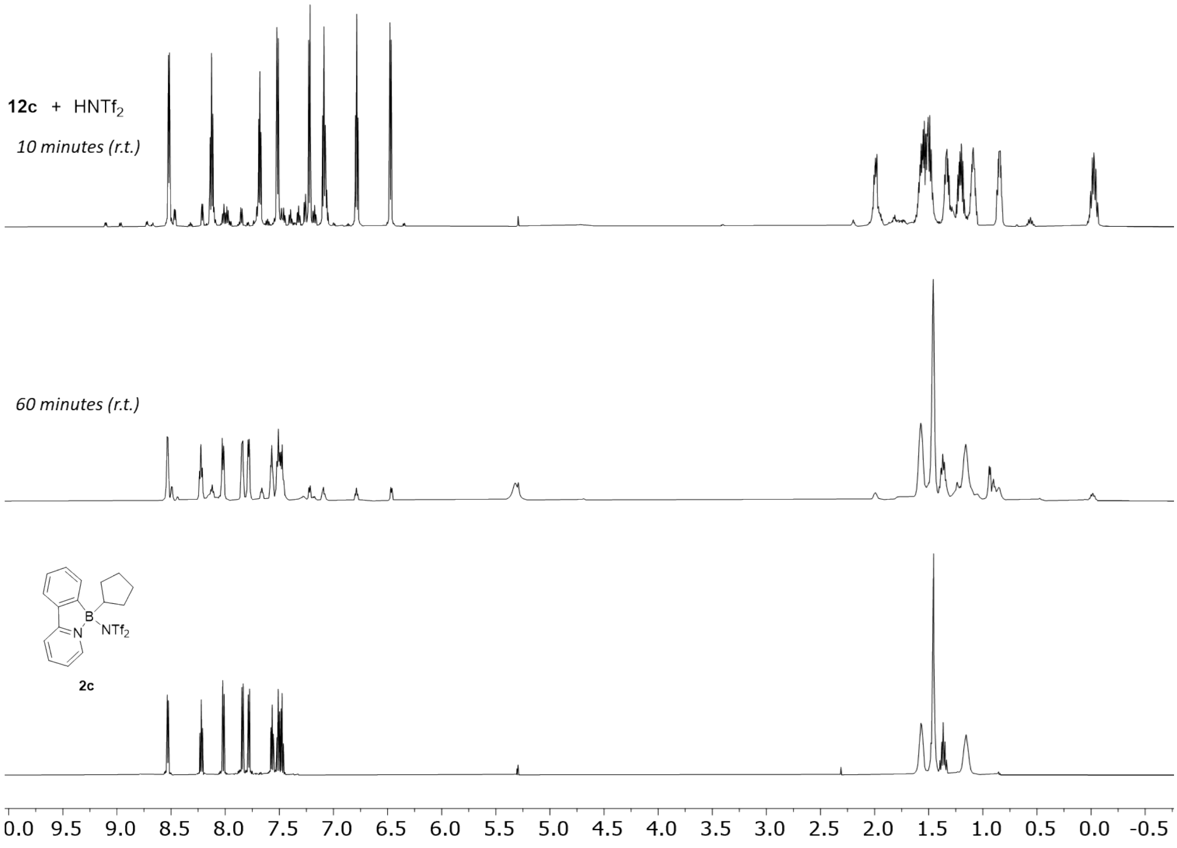

Figure S68: **^{1}H NMR** (CD_2Cl_2, 600 MHz, 293 K) spectra monitoring the reaction of **12c** with triflimic acid and the comparison with an authentic sample of **2c**.

Cycloaddition chemistry, preparation of **14-16**

Preparation of compound **14a**

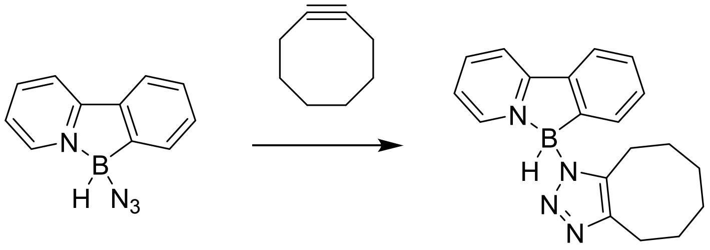

3a 14a

Boron azide **3a** (41.6 mg, 0.200 mmol) and cyclooctyne (33 mg, 0.30 mmol) were combined in of chloroform (4 mL) and the resulting mixture was stirred at 60 °C overnight. After cooling to room temperature, reaction mixture was transferred on a top of a short silica gel column which was eluted by dichloromethane to wash out the unreacted cyclooctyne. Subsequently, the polarity of the mobile phase was increased to dichloromethane / methanol (10:1) mixture for the elution of the triazole product. After removal of volatiles, product **14a** was obtained as a colourless oil which rapidly crystalized (59.5 mg, 0.188 mmol, 94% yield).

Elemental analysis calculated for $C_{19}H_{21}N_4B$ (316.2): C 72.17, H 6.69, N 17.72; found C 72.12, H 6.79, N 18.08.

HRMS calculated for $C_{19}H_{22}BN_4Na^+$ $[M+H]^+$: 317.1932, found 317.1926.

IR (ATR) ν: 3067 (w), 3011 (w), 2923 (m), 2850 (m), **2418 (m, B-H)**, 1622 (s), 1565 (m), 1486 (s), 1442 (m), 1368 (w), 13651 (w), 1329 (w), 1313 (m), 1289 (w), 1280 (w), 1263 (m), 1231 (m), 1216 (m), 1203 (m), 1173 (m), 1156 (s), 1134 (w), 1122 (m), 1109 (w), 1096 (m), 1064 (m), 1037 (s), 1006 (s), 942 (w), 874 (w), 864 (w), 803 (w), 783 (m), 767 (m), 746 (s), 730 (s), 690 (m), 634 (m), 571 (w), 550 (w), 518 (w), 451 (w), 415 (m) cm^{-1}.

^{1}H NMR (600 MHz, $CDCl_3$, 293 K): δ = [8.24 (d, $^3J_{HH}$ = 5.7 Hz), 8.07 (dd, $^3J_{HH} \approx {}^3J_{HH}$ = 7.8 Hz), 8.01 (d, $^3J_{HH}$ = 7.8 Hz), 7.34 (dd, $^3J_{HH}$ = 7.8 Hz, $^3J_{HH}$ = 5.7 Hz)](each 1H, pyridyl), [7.86 (d, $^3J_{HH}$ = 7.6 Hz), 7.60 (d, $^3J_{HH}$ = 7.2 Hz), 7.45 (dd, $^3J_{HH} \approx {}^3J_{HH}$ = 7.5 Hz), 7.39 (dd, $^3J_{HH} \approx {}^3J_{HH}$ = 7.5 Hz)](each 1H, C_6H_4), 4.64 (br, 1H, BH), [2.85, 2.85, 2.50, 2.50, 1.72, 1.68, 1.48, 1.39, 1.39, 1.38, 1.29, 1.28](each m, 1H, $(CH_2)_6$, peak assignment based on gHSQC).

^{13}C{^{1}H} NMR (151 MHz, $CDCl_3$, 293 K): δ = [157.8 (*i*-C), 143.3 (CH), 142.0 (CH), 122.0 (CH), 118.4 (CH)](pyridyl), [153.4 (br, B-C), 136.4 (*i*-C), 131.4 (CH), 130.6 (CH), 127.3 (CH), 121.4 (CH)](C_6H_4), [144.5, 137.4]($C^{triazole}$), [29.1, 27.5, 25.6, 25.4, 24.5, 21.8](($(CH_2)_6$)).

^{11}B{^{1}H} NMR (193 MHz, $CDCl_3$, 293 K): δ = –1.9 ($\nu_{1/2} \approx$ 250 Hz).

^{11}B NMR (193 MHz, $CDCl_3$, 293 K): δ = –1.9 ($\nu_{1/2} \approx$ 320 Hz).

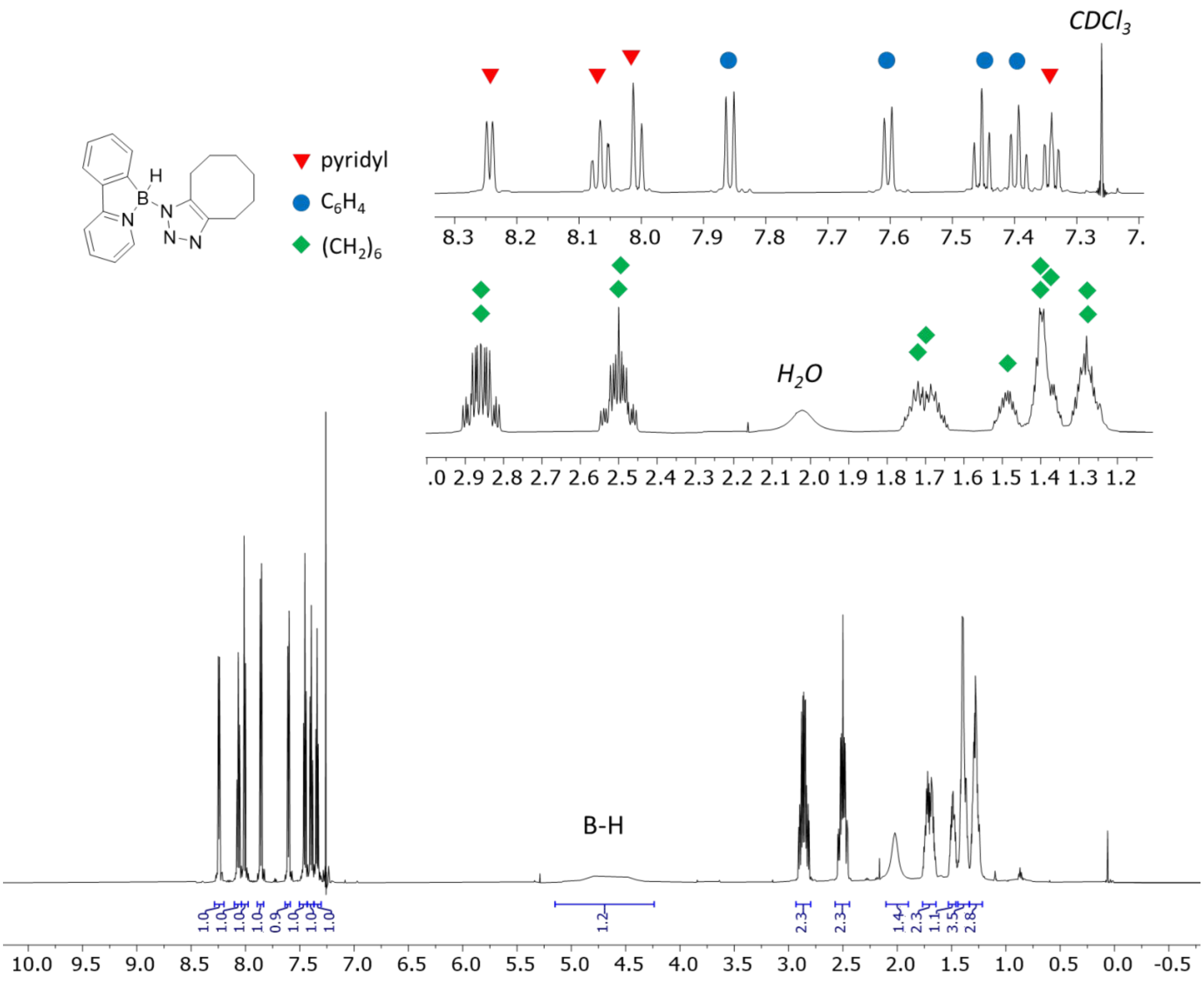

Figure S69: [1]**H NMR** ($CDCl_3$, 600 MHz, 293 K) spectrum of compound **14a.**

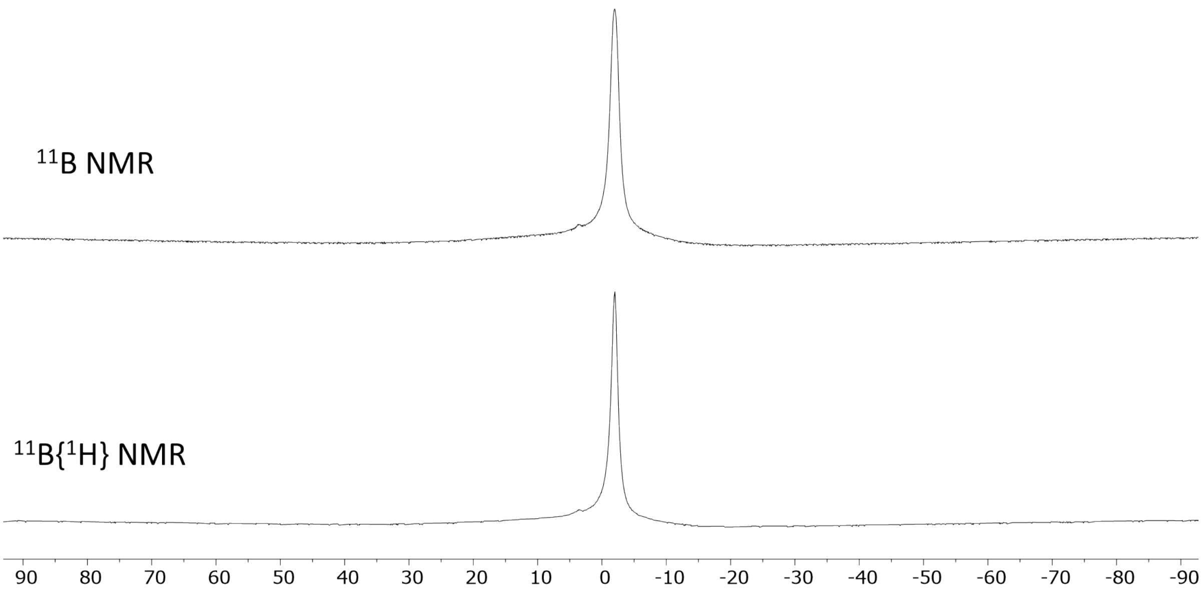

Figure S70: [11]**B** and [11]**B{^{1}H} NMR** (193 MHz, $CDCl_3$, 293 K) spectra of compound **14a**.

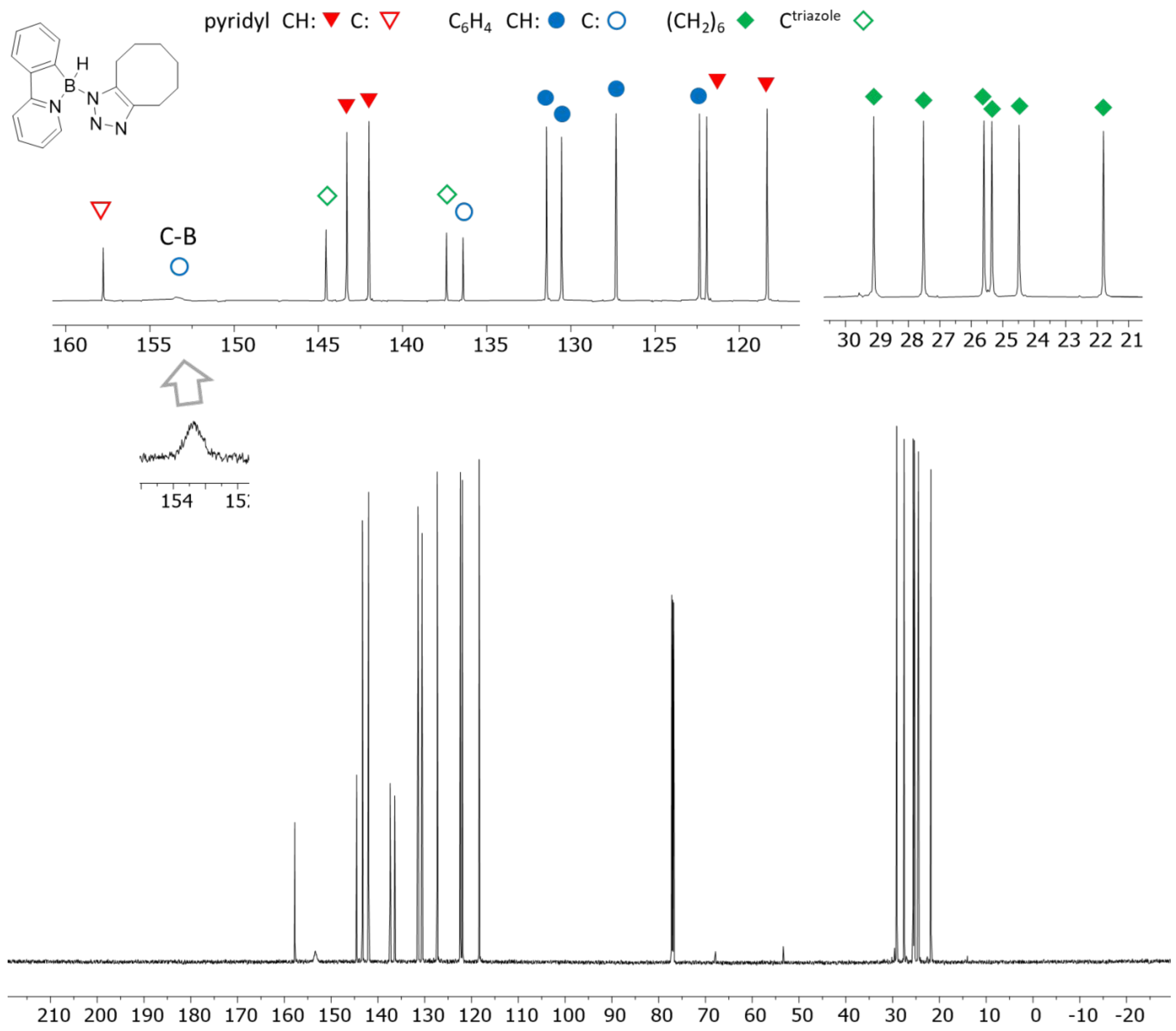

Figure S71: **^{13}C{^{1}H} NMR** (151 MHz, $CDCl_3$, 293 K) spectrum of compound **14a**.

Crystal structure determination of 14a [KSK478-gg]

A crystal suitable for SC-XRD structure determination was obtained by slow evaporation of a solution of **14a** in a dichloromethane:heptane mixture (ca. 1:1) under ambient conditions.

Crystal Data for $C_{19}H_{21}BN_4$ (M =316.21 g/mol): orthorhombic, space group Pbca (no. 61), a = 11.6685(2) Å, b = 10.9963(2) Å, c = 26.3151(6) Å, V = 3376.50(11) Å^3, Z = 8, T = 99.99(10) K, μ(Cu Kα) = 0.582 mm^{-1}, $Dcalc$ = 1.244 g/cm^3, 13397 reflections measured (6.718° ≤ 2Θ ≤ 152.804°), 3313 unique (R_{int} = 0.0343, R_{sigma} = 0.0315) which were used in all calculations. The final R_1 was 0.0427 (I > 2σ(I)) and wR_2 was 0.1152 (all data). **CCDC: 2445682**

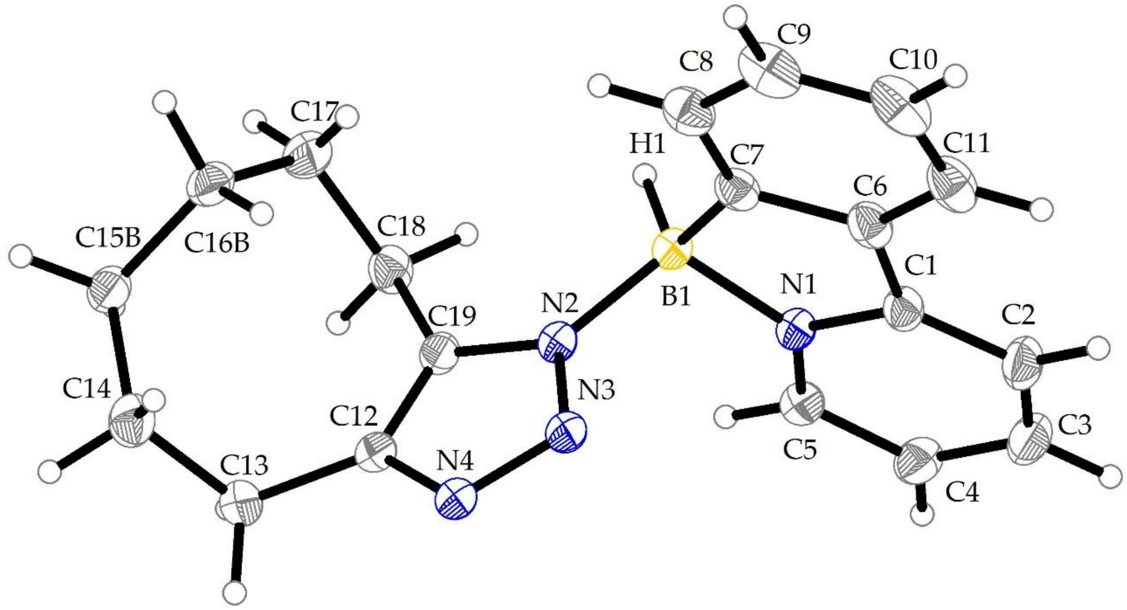

Figure S72: Crystal structure of **14a** (thermal ellipsoids shown at 30 % probability level). Some disordered atoms are omitted for clarity.

Preparation of compound **14b**

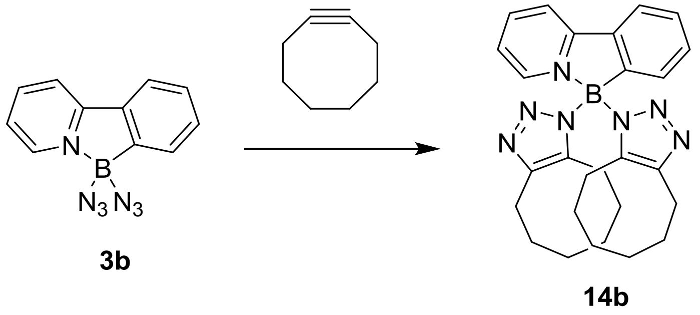

Boron diazide **3b** (24.9 mg, 0.100 mmol) and cyclooctyne (33 mg, 0.300 mmol) were combined in of chloroform (4 mL) and the resulting mixture was stirred at 60 °C for 40 hours. After cooling to room temperature, reaction mixture was transferred on top of a short silica gel column and eluted by dichlormethane to wash out the unreacted cyclooctyne. Subsequently, the polarity of the mobile phase was increased to dichloromethane / methanol (10:1) mixture for the elution of the bistriazole product **14b**. After the removal of volatiles, **14b** was obtained as a colourless oil (41.8 mg, 0.0898 mmol, 90% yield).

Elemental analysis calculated for $C_{27}H_{32}N_7B$ (465.4): C 69.68, H 6.93, N 21.07; found C 69.30, H 7.13, N 20.78.

HRMS calculated for $C_{27}H_{32}BN_7Na^+$ $[M+Na]^+$: 488.2708, found 488.2694.

IR (ATR) ν: 3057 (w), 2922 (m), 2852 (m), 1624 (s), 1555 (w), 1489 (s), 1444 (s), 1368 (w), 1348 (w), 1332 (w), 1310 (w), 1262 (w), 1236 (m), 1202 (w), 1165 (m), 1129 (w), 1101 (w), 1077 (m), 1003 (w), 950 (w), 898 (s), 869 (s), 850 (s), 821 (s), 769 (s), 743 (s), 664 (w), 636 (w), 551 (w), 418 (m) cm^{-1}.

^{1}H NMR (600 MHz, $CDCl_3$, 293 K): δ = [8.91 (dd, $^3J_{HH}$ = 5.8 Hz, $^4J_{HH}$ = 1.4 Hz), 8.05 (ddd, $^3J_{HH} \approx {}^3J_{HH}$ = 7.8 Hz, $^4J_{HH}$ = 1.5 Hz), 7.92 (d, $^3J_{HH}$ = 7.9 Hz), 7.37 (dd, $^3J_{HH}$ = 7.8 Hz, $^3J_{HH}$ = 5.7 Hz)](each 1H, pyridyl), [7.78 (d, $^3J_{HH}$ = 7.8 Hz), 7.77 (d, $^3J_{HH}$ = 7.8 Hz), 7.45 (dd, $^3J_{HH} \approx {}^3J_{HH}$ = 7.5 Hz), 7.42 (dd, $^3J_{HH} \approx {}^3J_{HH}$ = 7.5 Hz)](each 1H, C_6H_4), [2.86 (m, 2H), 2.77 (m, 2H), 2.43 (m, 4H), 1.67 (m, 4H), 1.34-1.46 (m, 8H), 1.26-1.33 (m, 4H)](($CH_2)_6$).

^{13}C{^{1}H} NMR (151 MHz, $CDCl_3$, 293 K): δ = [156.7 (*i*-C), 146.4 (CH), 144.0 (CH), 123.2 (CH), 118.0 (CH)](pyridyl), [147.0 (br, B-C), 137.2 (*i*-C), 131.7 (CH), 131.1 (CH), 129.3 (CH), 122.6 (CH)](C_6H_4), [145.4, 137.3]($C^{triazole}$), [28.7, 27.6, 25.5, 25.3, 24.1, 22.7]$(CH_2)_6$.

^{11}B{^{1}H} NMR (193 MHz, $CDCl_3$, 293 K): δ = 3.6 ($\nu_{1/2} \approx$ 200 Hz).

^{11}B NMR (193 MHz, $CDCl_3$, 293 K): δ = 3.6 ($\nu_{1/2} \approx$ 220 Hz).

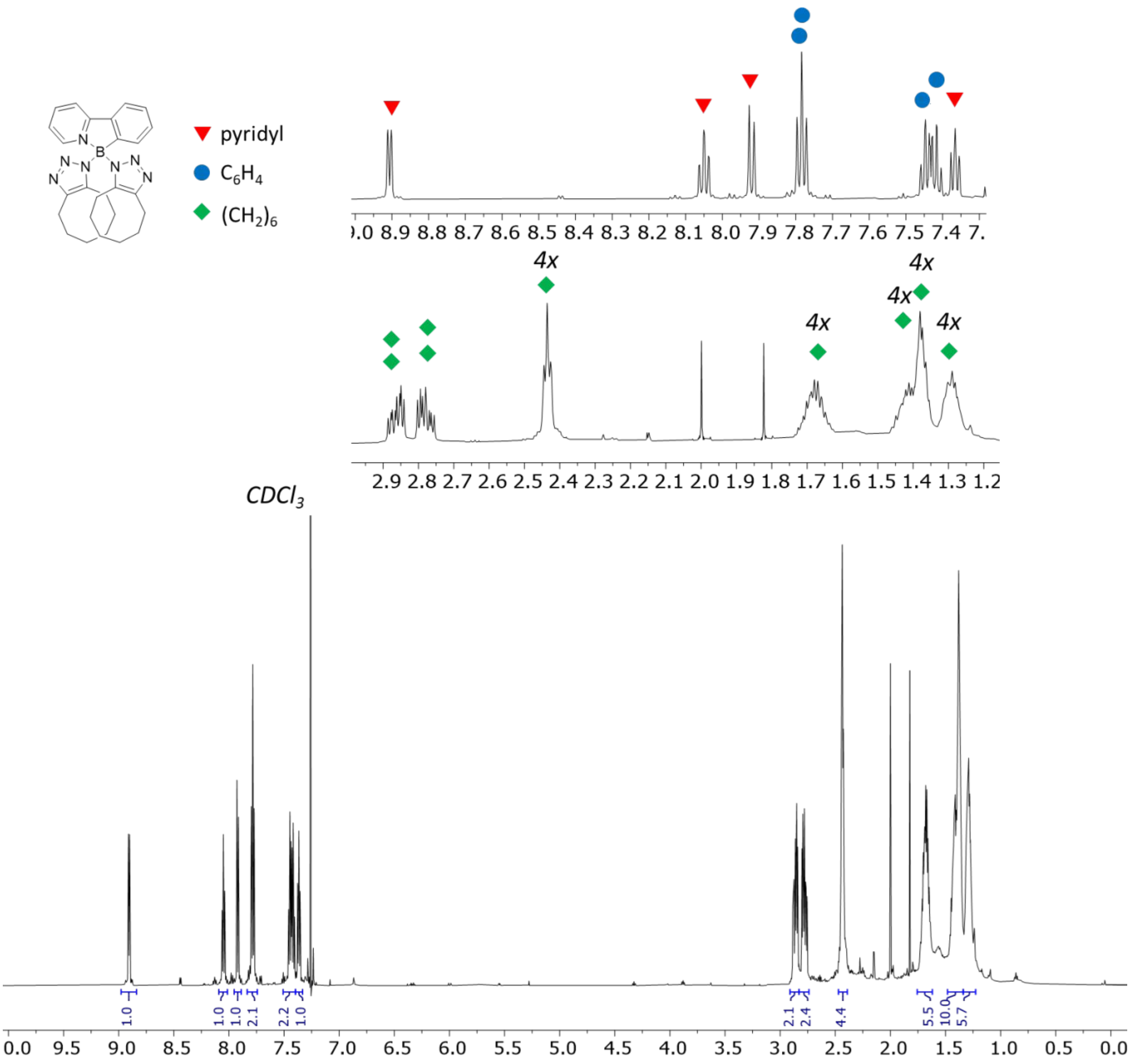

Figure S73: **^{1}H NMR** ($CDCl_3$, 600 MHz, 293 K) spectrum of compound **14b**

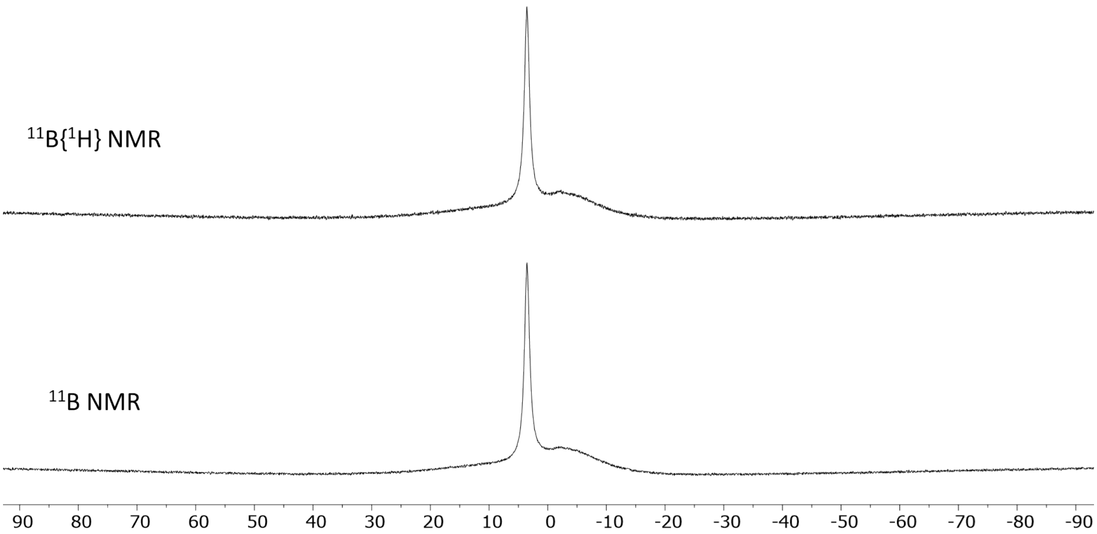

Figure S74: **^{11}B** and **^{11}B{^{1}H} NMR** (193 MHz, $CDCl_3$, 293 K) spectra of compound **14b**.

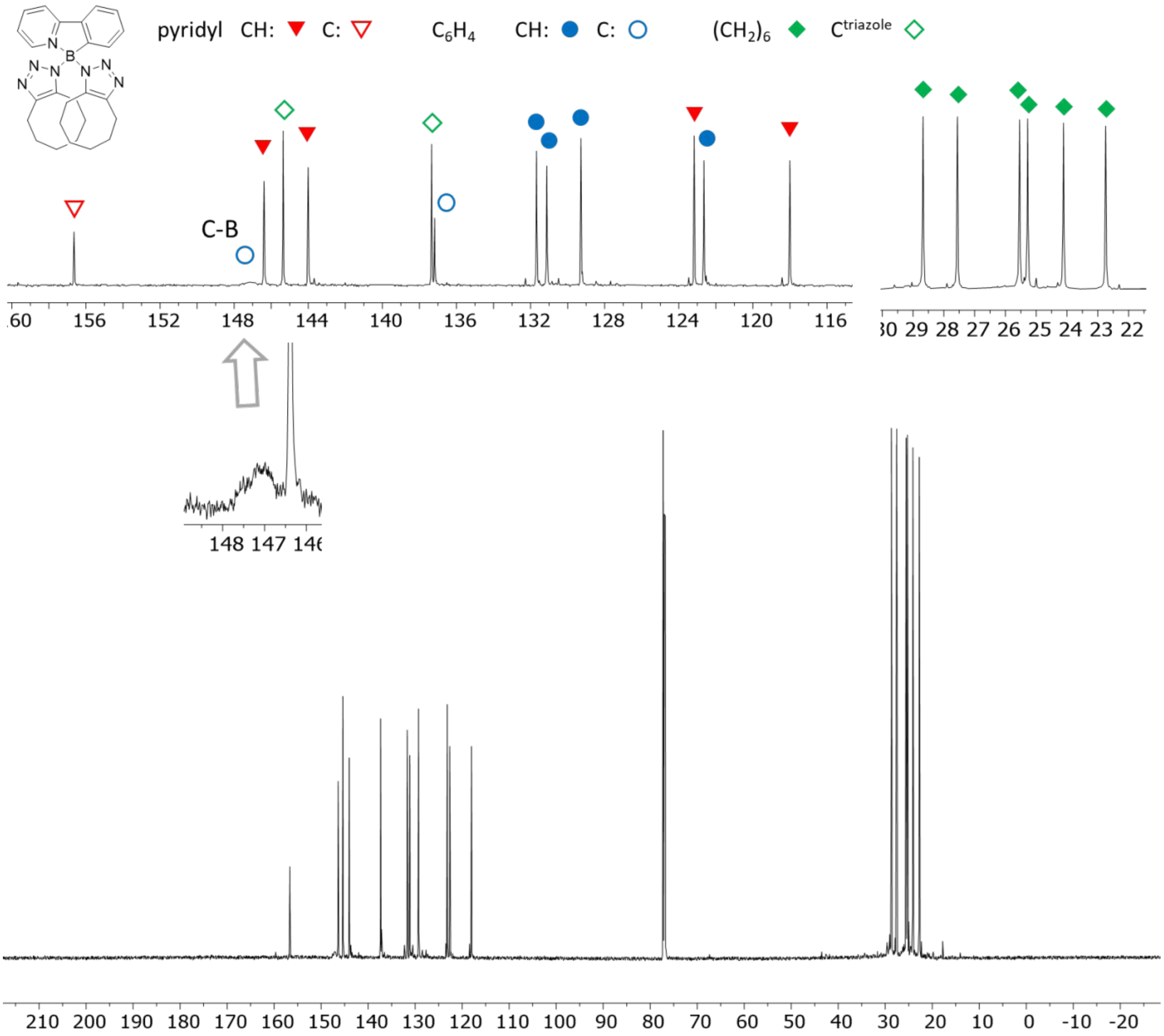

Figure S75: **^{13}C{^{1}H} NMR** (151 MHz, $CDCl_3$, 293 K) spectrum of compound **14b**.

Crystal structure determination of **14b [KSK480-gg]**

A crystal suitable for SC-XRD structure determination was obtained by slow evaporation of a solution of **14b** in a dichloromethane:heptane mixture (ca 1:1) under ambient conditions.

Crystal Data for $C_{27}H_{32}BN_7$ (M =465.40 g/mol): triclinic, space group P-1 (no. 2), a = 10.6172(4) Å, b = 11.8715(3) Å, c = 11.9587(4) Å, α = 111.862(3)°, β = 112.454(3)°, γ = 101.463(3)°, V = 1186.32(8) Å^3, Z = 2, T = 100.00(10) K, μ(Cu Kα) = 0.624 mm^{-1}, $Dcalc$ = 1.303 g/cm^3, 14067 reflections measured (8.746° ≤ 2Θ ≤ 152.39°), 4641 unique (R_{int} = 0.0350, R_{sigma} = 0.0353) which were used in all calculations. The final R_1 was 0.0436 (I > 2σ(I)) and wR_2 was 0.1208 (all data). **CCDC: 2445686**

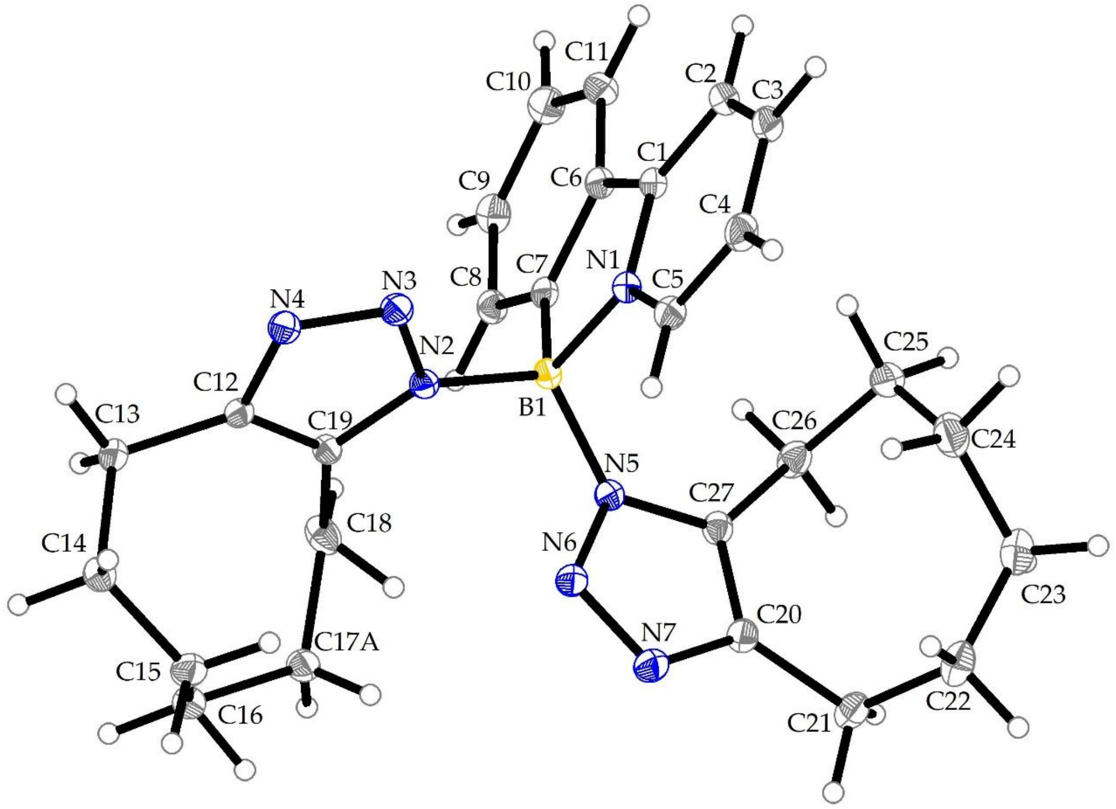

Figure S76: Crystal structure of **14b** (thermal ellipsoids shown at 30 % probability level).

Preparation of compound **14c**

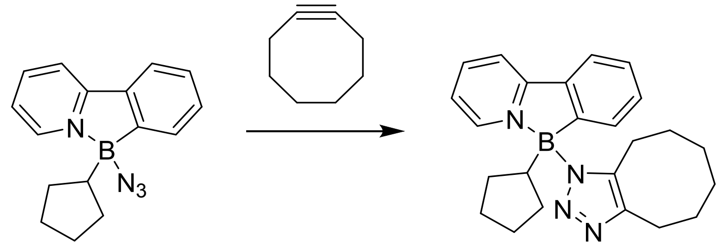

3c **14c**

Boron azide **3c** (27.6 mg, 0.100 mmol) and cyclooctyne (17 mg, 0.15 mmol) were combined in of chloroform (4 mL) and the resulting mixture was stirred at 60 °C overnight. After cooling to room temperature, reaction mixture was transferred on top of a short silica gel column and eluted with dichloromethane to wash out the unreacted cyclooctyne. Subsequently, the polarity of the mobile phase was increased to dichloromethane / methanol (10:1) mixture and the bistriazole product was eluted. After the removal of volatiles, product **14c** was obtained as a colourless oil, which rapidly solidified (35.5 mg, 0.0924 mmol, 92% yield).

Elemental analysis calculated for $C_{24}H_{29}N_4B$ (384.3): C 75.00, H 7.61, N 14.58; found C 74.96, H 7.81, N 14.35.

HRMS calculated for $C_{24}H_{29}BN_4Na^+$ $[M+H]^+$: 385.2558, found 385.2549.

IR (ATR) ν: 3024 (w), 2924 (m), 2853 (m), 1620 (s), 1571 (m), 1485 (s), 1447 (m), 1358 (w), 1309 (w), 1253 (m), 1236 (m), 1194 (m), 1173 (m), 1124 (m), 1090 (w), 1067 (m), 1041 (w), 1003 (m), 957 (w), 885 (w), 849 (m), 764 (s), 750 (s), 738 (s), 675 (w), 552 (w), 432 (m) cm^{-1}.

^{1}H NMR (600 MHz, $CDCl_3$, 293 K): δ = [8.35 (d, $^3J_{HH}$ = 5.7 Hz), 8.07 (dd, $^3J_{HH} \approx {}^3J_{HH}$ = 7.7 Hz), 8.00 (d, $^3J_{HH}$ = 7.9 Hz), 7.35 (dd, $^3J_{HH}$ = 7.6 Hz, $^3J_{HH}$ = 5.7 Hz)](each 1H, pyridyl), [7.88 (d, $^3J_{HH}$ = 7.6 Hz), 7.67 (d, $^3J_{HH}$ = 7.2 Hz), 7.45 (m), 7.42 (m)](each 1H, C_6H_4), [2.77, 2.72, 1.90, 1.78, 1.64, 1.55, 1.21, 1.18, 1.13, 1.03, 0.92, 0.57](each m, 1H, $(CH_2)_6$), [2.22, 2.11, 1.52, 1.46, 1.36, 1.25, 1.17, 0.81, 0.16](each m, 1H, cyclopentyl), peak assignment based on gHSQC and ^{1}H TOCSY experiments.

^{13}C{^{1}H} NMR (151 MHz, $CDCl_3$, 293 K): δ = [156.3 (*i*-C), 142.7 (CH), 141.6 (CH), 122.7 (CH), 117.8 (CH)](pyridyl), [154.3 (br, B-C), 136.7 (*i*-C), 131.0 (CH), 131.0 (CH), 127.3 (CH), 121.7 (CH)](C_6H_4), [144.9, 137.1]($C^{triazole}$), [33.4 (br, B-CH), 29.4, 29.2, 28.2, 26.3](cyclopentyl) [27.3, 26.9, 25.5, 25.4, 24.5, 21.7]($(CH_2)_6$).

^{11}B{^{1}H} NMR (193 MHz, $CDCl_3$, 293 K): δ = 3.9 ($\nu_{1/2} \approx$ 250 Hz).

^{11}B NMR (193 MHz, $CDCl_3$, 293 K): δ = 3.9 ($\nu_{1/2} \approx$ 260 Hz).

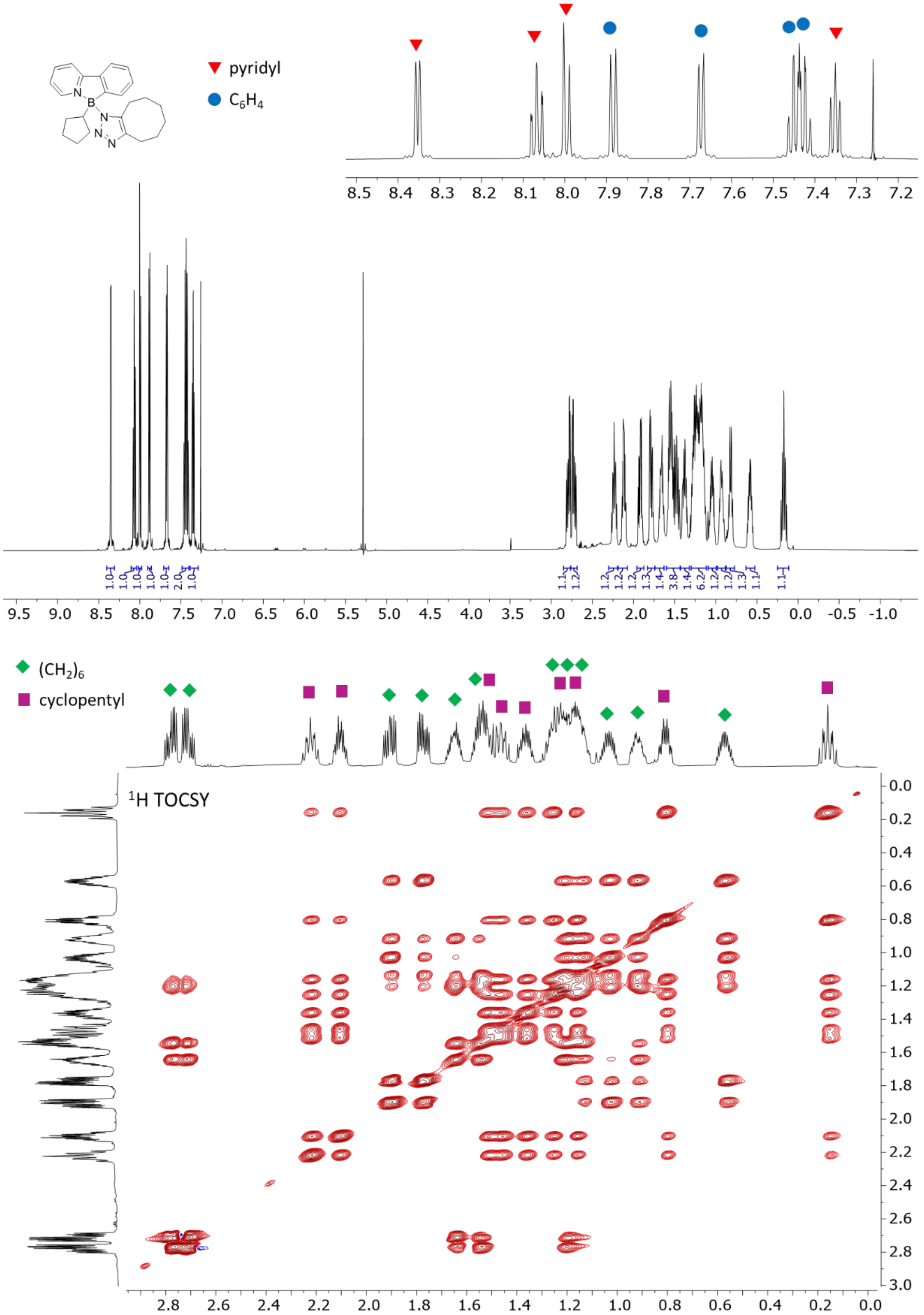

Figure S77: **¹H NMR** (600 MHz, $CDCl_3$, 293 K) spectrum of compound **14c** and excerpt from **¹H TOCSY** (600 MHz, $CDCl_3$, 293 K).

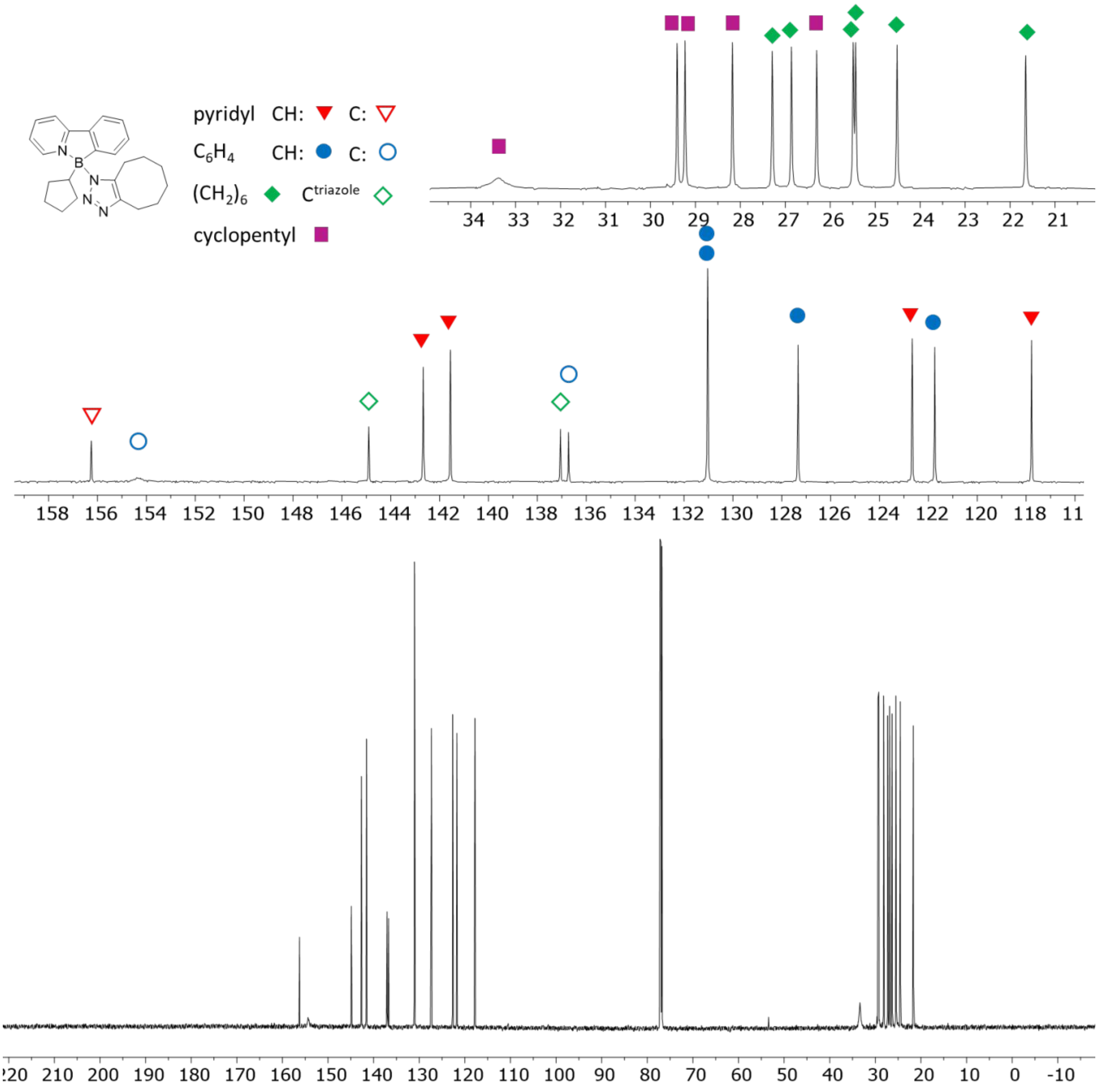

Figure S78: **$^{13}C\{^1H\}$ NMR** (151 MHz, $CDCl_3$, 293 K) spectrum of compound **14c**.

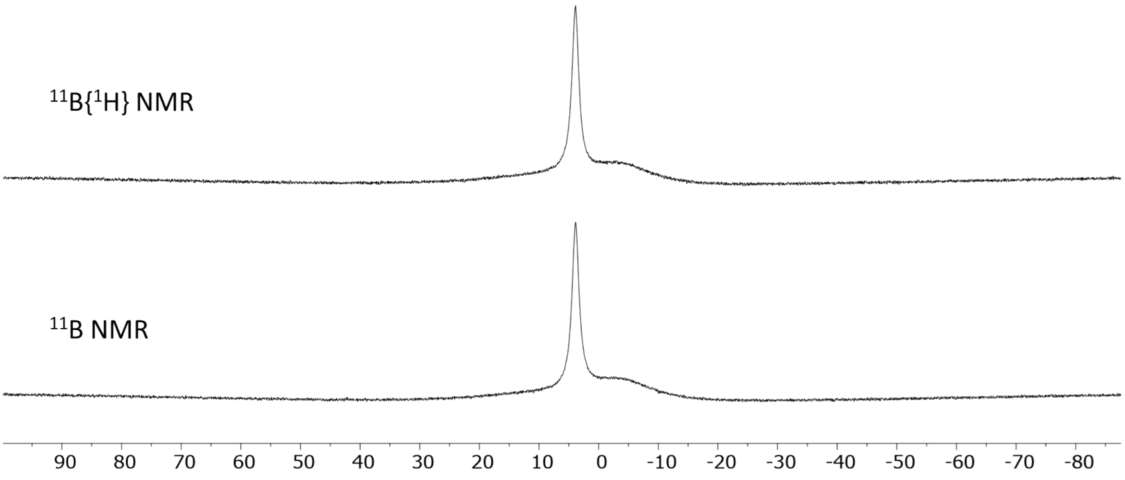

Figure S79: **^{11}B** and **$^{11}B\{^1H\}$ NMR** (193 MHz, $CDCl_3$, 293 K) spectra of compound **14c**.

Crystal structure determination of **14c [KSK480-gg]**

A crystal suitable for SC-XRD structure determination was obtained by slow evaporation of a solution of **14c** in a dichloromethane:heptane mixture (ca 1:1) under ambient conditions.

Crystal Data for $C_{24}H_{29}BN_4$ (M =384.32 g/mol): orthorhombic, space group Pbca (no. 61), a = 14.0592(2) Å, b = 15.0588(2) Å, c = 20.2961(3) Å, V = 4296.98(11) Å^3, Z = 8, T = 100.00(10) K, μ(Cu Kα) = 0.542 mm^{-1}, $Dcalc$ = 1.188 g/cm^3, 26440 reflections measured (8.714° ≤ 2Θ ≤ 133.2°), 3795 unique (R_{int} = 0.0469, R_{sigma} = 0.0333) which were used in all calculations. The final R_1 was 0.0445 (I > 2σ(I)) and wR_2 was 0.1255 (all data). **CCDC: 2451303**.

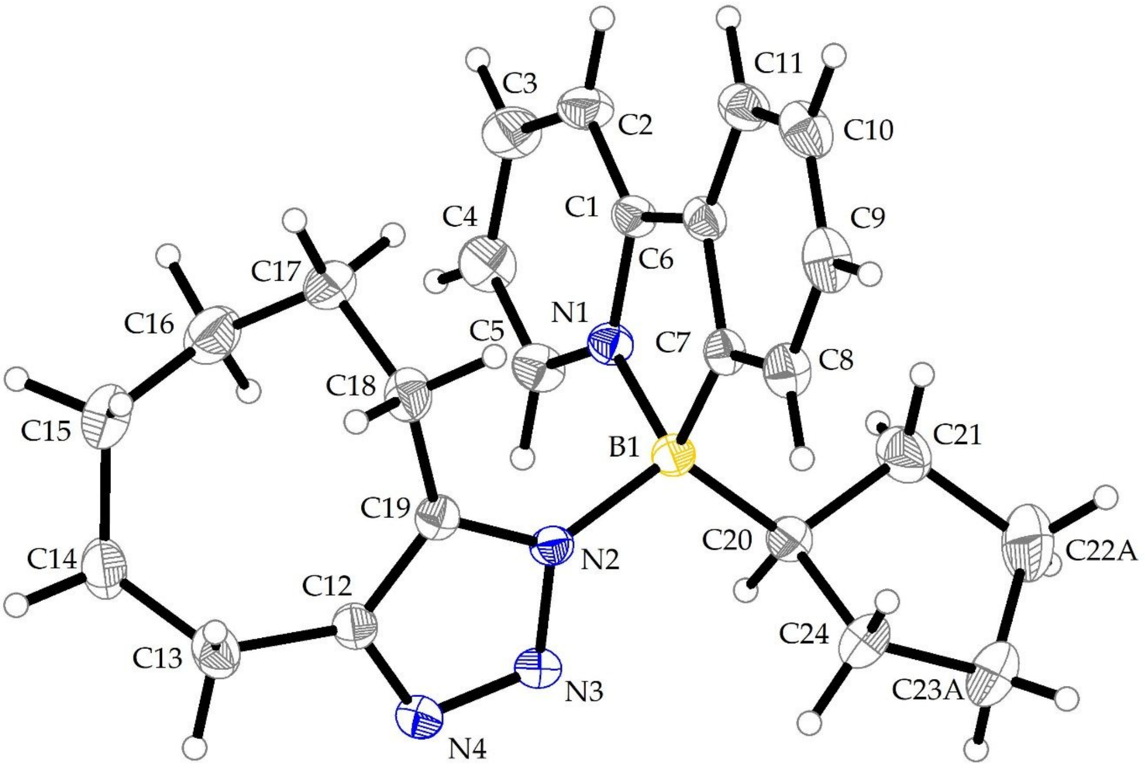

Figure S80: Crystal structure of **14c** (thermal ellipsoids shown at 30 % probability level). Some disordered atoms are omitted for clarity.

Preparation of compound **15a**

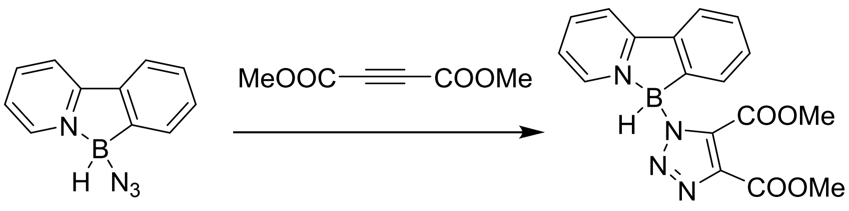

3a **15a**

Boron azide **3a** (104 mg, 0.500 mmol) in 5 mL of chloroform was treated with dimethyl acetylenedicarboxylate (DMAD, 78 mg, 0.55 mmol) in 5 mL of chloroform and the resulting mixture was stirred at 60 °C overnight. On the following day, the reaction mixture was directly loaded onto a chromatographic column packed with silica gel in dichloromethane. The column was first eluted with dichloromethane to remove impurities and subsequently the polarity of the mobile phase was increased to dichloromethane / methanol (10:1) mixture to elute the product. Compound **15a** was obtained as a white foamy solid (158 mg, 0.451 mmol, 90% yield) which exhibited purple-blue luminescence under irradiation with UV lamp.

Elemental analysis calculated for $C_{17}H_{15}N_4BO_4$ (350.1): C 58.31, H 4.32, N 16.00; found C 58.56, H 4.50, N 15.65.

HRMS calculated for $C_{17}H_{16}N_4BO_4^+$ $[M+H]^+$: 351.1260, found 351.1256.

IR (ATR) ν: 3090 (w), 3048 (w), 2954 (w), **2445 (m, B-H)**, **1723 (vs, C=O)**, 1626 (m), 1573 (w), 1541 (w), 1490 (m), 1449 (s), 1350 (m), 1335 (w), 1290 (w), 1252 (w), 1121 (s), 1179 (s), 1144 (s), 1086 (s), 1069 (m), 1055 (m), 1029 (s), 1009 (m), 968 (w), 947 (w), 836 (w), 811 (w), 768 (m), 746 (s), 734 (s), 693 (m), 635 (w), 568 (w), 458 (w) cm^{-1}.

^{1}H NMR (600 MHz, CDCl$_3$, 293 K): δ = [8.53 (d, $^3J_{HH}$ = 5.8 Hz), 8.10 (dd, $^3J_{HH} \approx {}^3J_{HH}$ = 7.8 Hz), 8.01 (d, $^3J_{HH}$ = 7.8 Hz), 7.39 (dd, $^3J_{HH}$ = 7.5 Hz, $^3J_{HH}$ = 5.8 Hz)](each 1H, pyridyl), [7.85 (d, $^3J_{HH}$ = 7.6 Hz), 7.60 (d, $^3J_{HH}$ = 7.2 Hz), 7.47 (dd, $^3J_{HH} \approx {}^3J_{HH}$ = 7.4 Hz), 7.40 (dd, $^3J_{HH} \approx {}^3J_{HH}$ = 7.4 Hz)](each 1H, C_6H_4), 4.55 (br m, 1H, BH), [3.91, 3.65](each s, 3H, COOMe).

^{13}C{^{1}H} NMR (151 MHz, CDCl$_3$, 293 K): δ = [162.4 (CO), 161.2 (CO), 137.8 (C triazole), 137.3 (C triazole), 53.0 (Me), 52.2 (Me)], [158.2 (ipso-C), 143.8 (CH), 142.6 (CH), 122.7 (CH), 118.5 (CH)](pyridyl), [150.5 (br, C-B), 137.2 (ipso-C), 131.7 (CH), 131.1 (CH), 128.0 (CH), 121.8 (CH)](C_6H_4).

^{11}B{^{1}H} NMR (193 MHz, CDCl$_3$, 293 K): δ = –1.8 ($\nu_{1/2} \approx$ 250 Hz).

^{11}B NMR (193 MHz, CDCl$_3$, 293 K): δ = –1.8 ($\nu_{1/2} \approx$ 330 Hz).

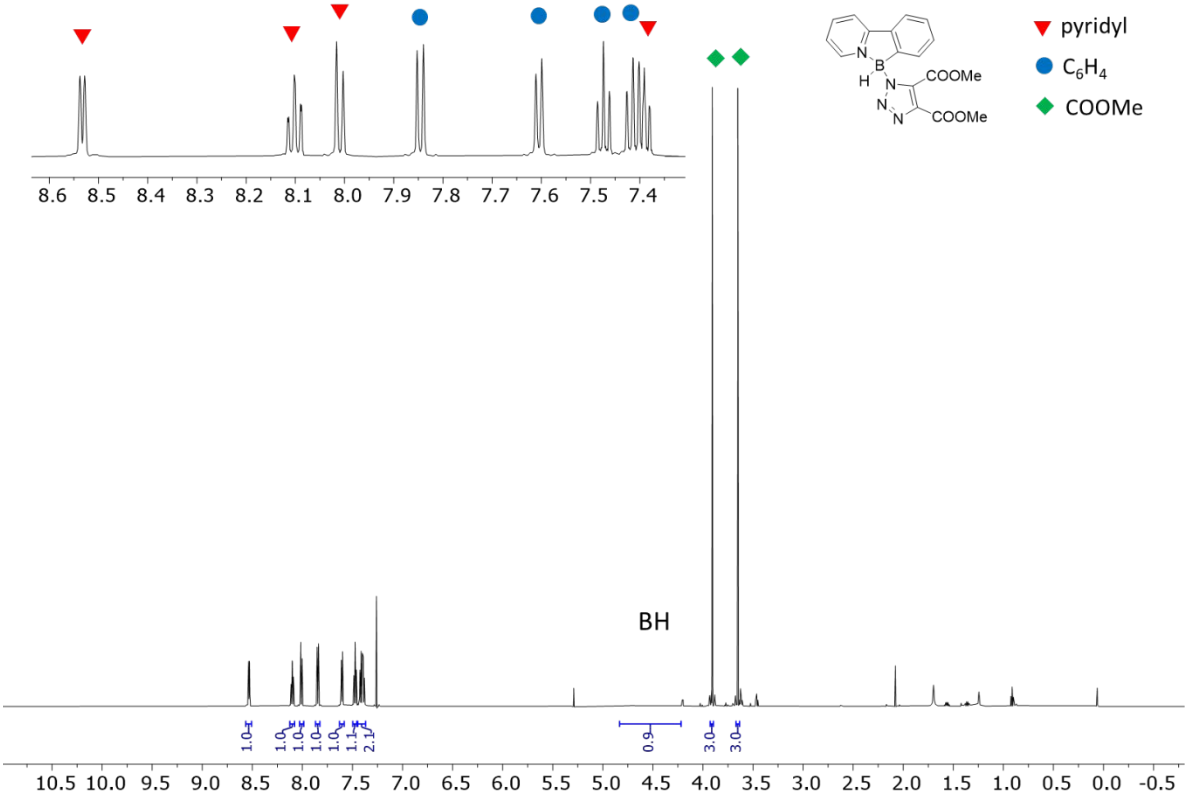

Figure S81: **¹H NMR** (600 MHz, $CDCl_3$, 293 K) spectrum of compound **15a**.

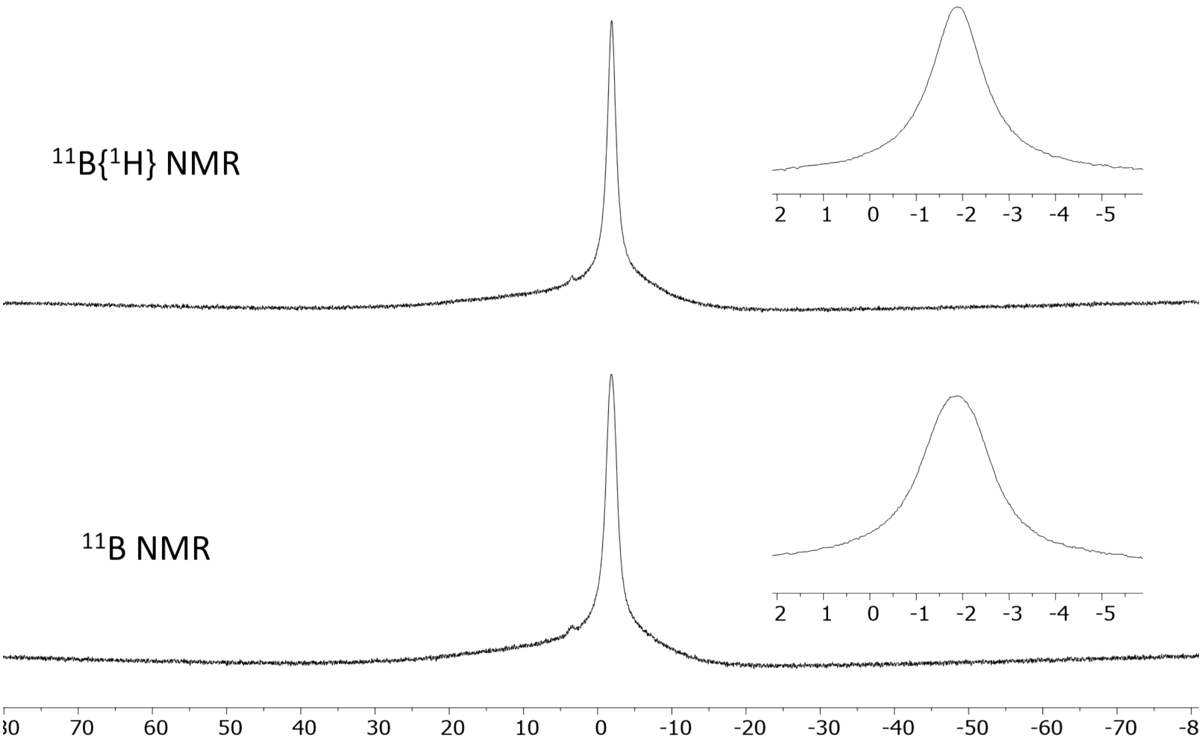

Figure S82: **¹¹B** and **¹¹B{¹H} NMR** (193 MHz, $CDCl_3$, 293 K) spectra of compound **15a**.

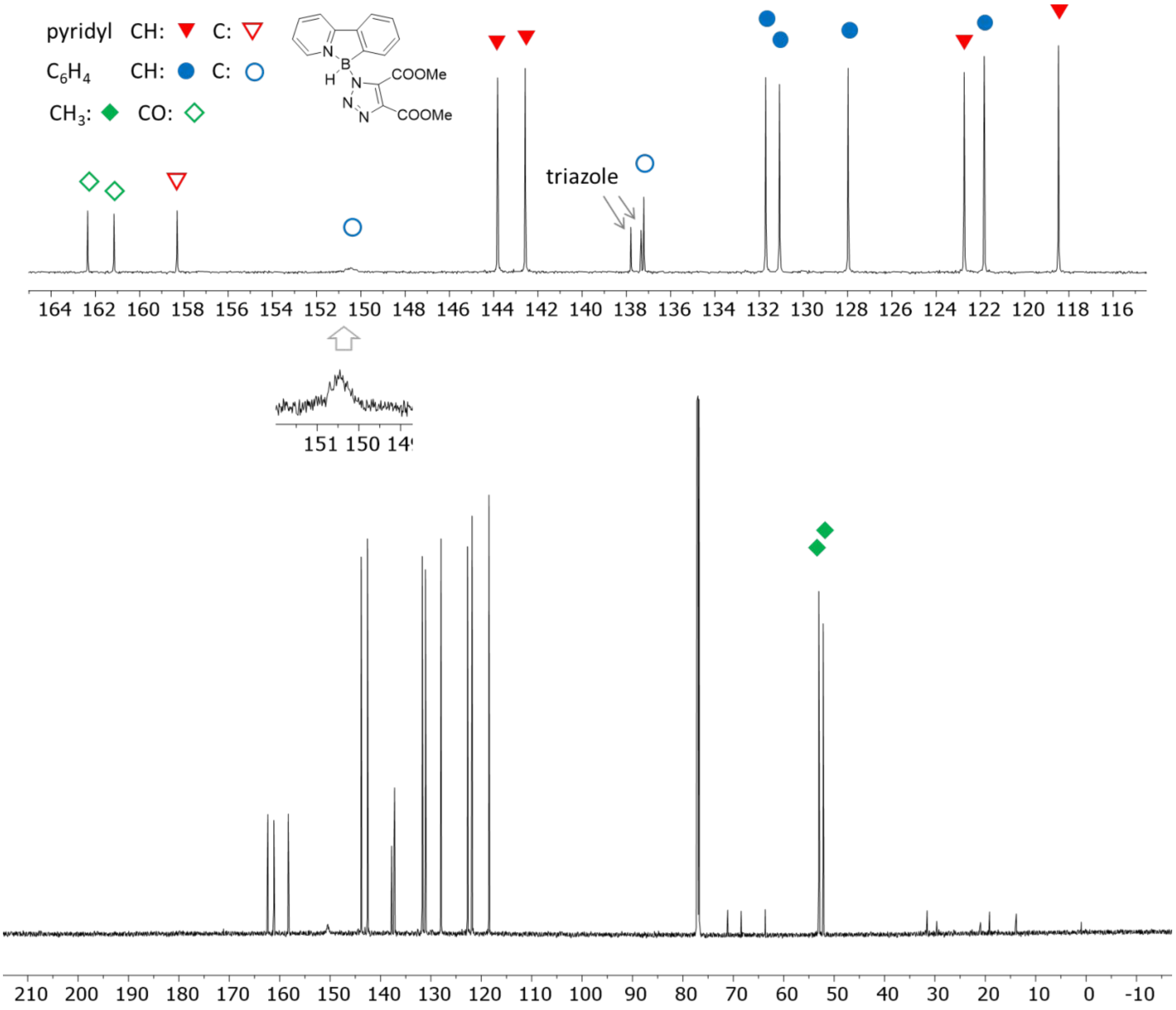

Figure S83: $^{13}C\{^{1}H\}$ **NMR** (151 MHz, $CDCl_3$, 293 K) spectrum of compound **15a**.

Preparation of compound **15b**

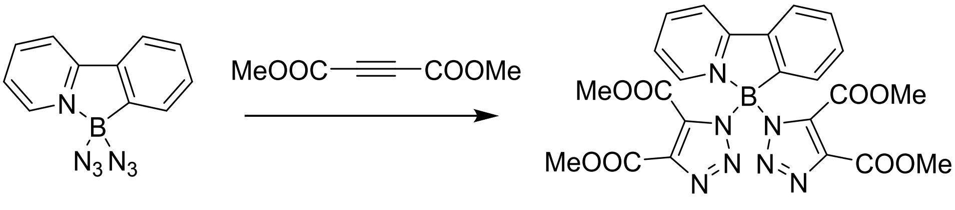

3b **15b**

Boron diazide **3b** (24.9 mg, 0.100 mmol) and dimethylacetylene dicarboxylate (DMAD, 42.6 mg, 0.300 mmol) were dissolved in $CDCl_3$ and placed in an NMR tube which was then sealed and placed to an oil bath preheated to 60 °C. Reaction progress was monitored by NMR. After 3 days, the NMR showed a complete conversion of the boron diazide, therefore the mixture was placed directly on top of a short silica gel column which was first eluted with dichloromethane to wash out the unreacted diester. Subsequently, the polarity of the mobile phase was increased to dichloromethane / methanol (10:1) mixture to elute the product, bistriazole **15b**, which was obtained as an oil that gradually solidified to give an off-white solid (48.8 mg, 0.0916 mmol, 92% isolated yield).

Elemental analysis calculated for $C_{23}H_{20}N_7BO_8$ (533.3): C 51.80, H 3.78, N 18.39; found C 51.86, H 3.76, N 18.79.

HRMS calculated for $C_{23}H_{20}N_7BO_8Na^+$ $[M+Na]^+$: 556.1359, found 556.1343.

IR (ATR) ν: 3089 (w), 2950 (m), **1730 (vs, CO)**, 1626 (s), 1533 (w), 1493 (m), 1451 (m), 1435 (m¨), 1365 (w), 1350 (w), 1272 (m), 1212 (s), 1178 (s), 1163 (s), 1143 (s), 1103 (m), 1076 (s), 1044 (m), 969 (w), 946 (w), 918 (w), 898 (m), 895 (s), 835 (s), 799 (m), 772 (s), 753 (s), 723 (w), 693 (w), 635 (w), 585 (w), 556 (m), 460 (w), 417 (m) cm^{-1}.

^{1}H NMR (600 MHz, $CDCl_3$, 293 K): δ = [8.62 (d, $^3J_{HH}$ = 5.8 Hz), 8.22 (dd, $^3J_{HH} \approx {}^3J_{HH}$ = 7.8 Hz), 8.06 (d, $^3J_{HH}$ = 7.8 Hz), 7.49 (dd, (dd, $^3J_{HH}$ = 7.9 Hz, $^3J_{HH}$ = 5.8 Hz)](each 1H, pyridyl), [8.02 (m), 7.84 (m), 7.47 (m), 7.46 (m)](each 1H, C_6H_4), [3.87, 3.48](each s, 6H, COOMe).

^{13}C{^{1}H} NMR (151 MHz, $CDCl_3$, 293 K): δ = [160.7 (CO), 160.7 (CO), 138.8 (C triazole), 136.5 (C triazole), 52.9 (Me), 52.3 (Me)], [156.8 (*i*-C), 145.3 (CH), 144.7 (CH), 123.9 (CH), 118.8 (CH)](pyridyl), [143.4 (br, C-B), 137.9 (*i*-C), 132.4 (CH), 132.2 (CH), 130.2 (CH), 122.5 (CH)](C_6H_4).

^{11}B{^{1}H} NMR (193 MHz, $CDCl_3$, 293 K): δ = 3.7 ($\nu_{1/2} \approx$ 280 Hz).

^{11}B NMR (193 MHz, $CDCl_3$, 293 K): δ = 3.7 ($\nu_{1/2} \approx$ 290 Hz).

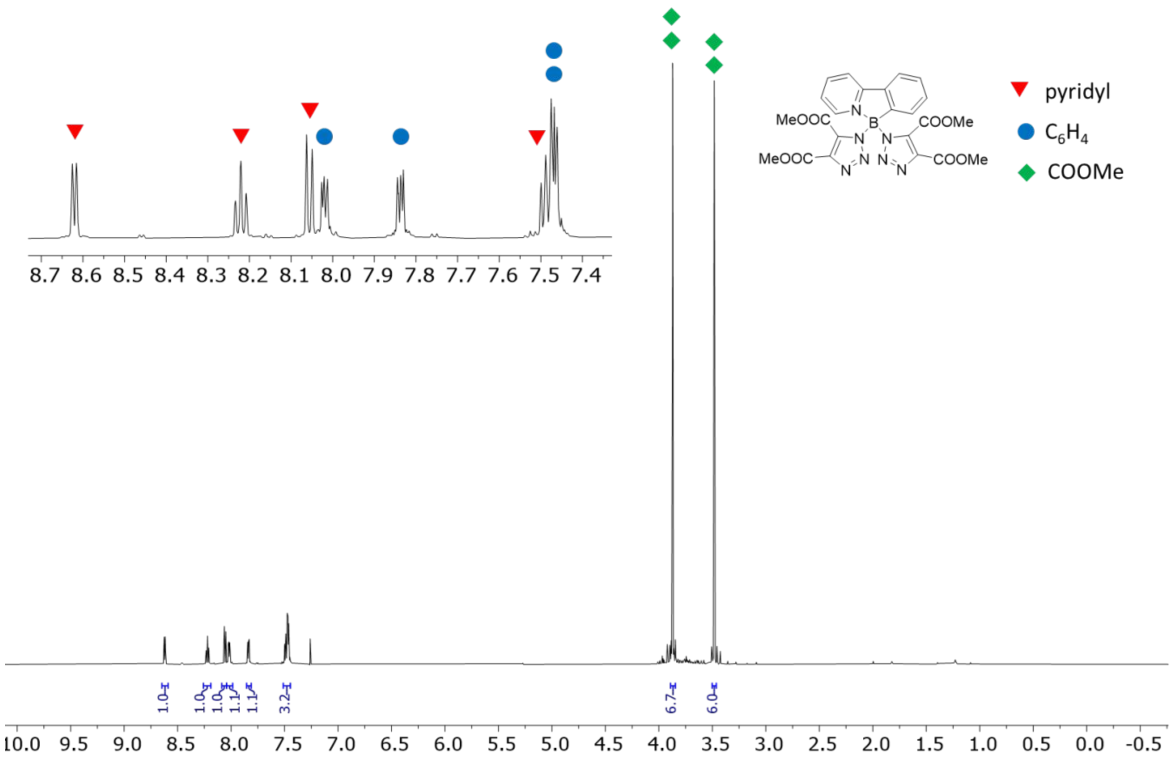

Figure S84: **^{1}H NMR** (600 MHz, $CDCl_3$, 293 K) spectrum of compound **15b**.

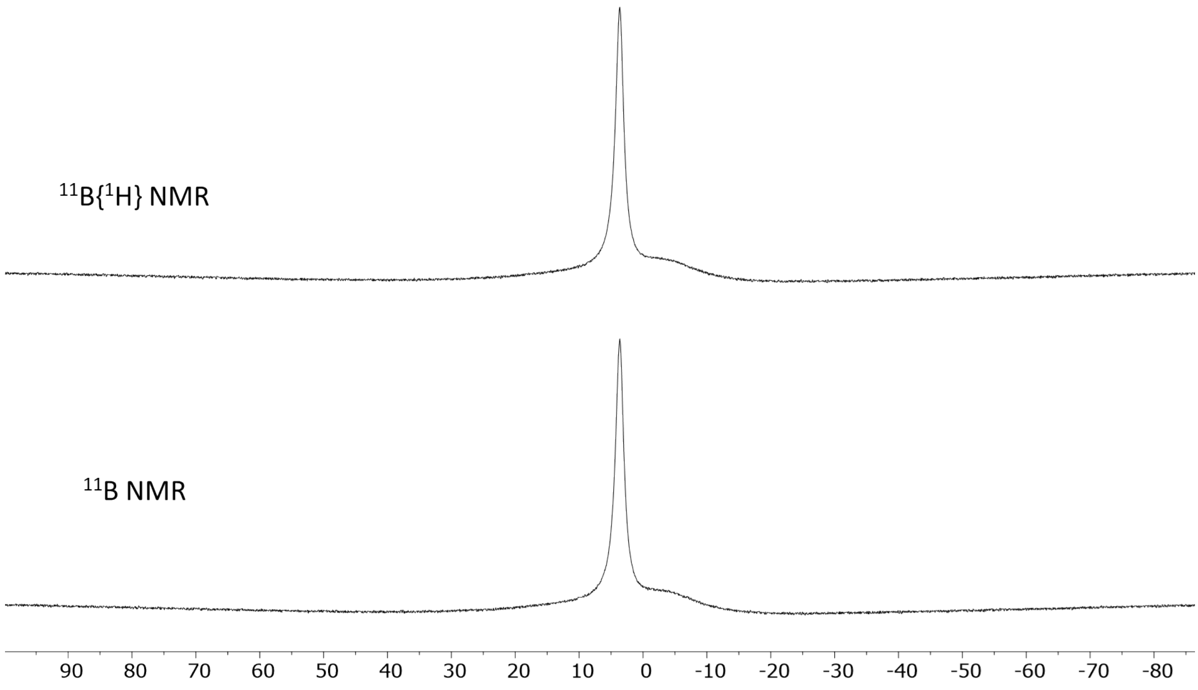

Figure S85: **^{11}B** and **^{11}B{^{1}H} NMR** (193 MHz, $CDCl_3$, 293 K) spectra of compound **15b.**

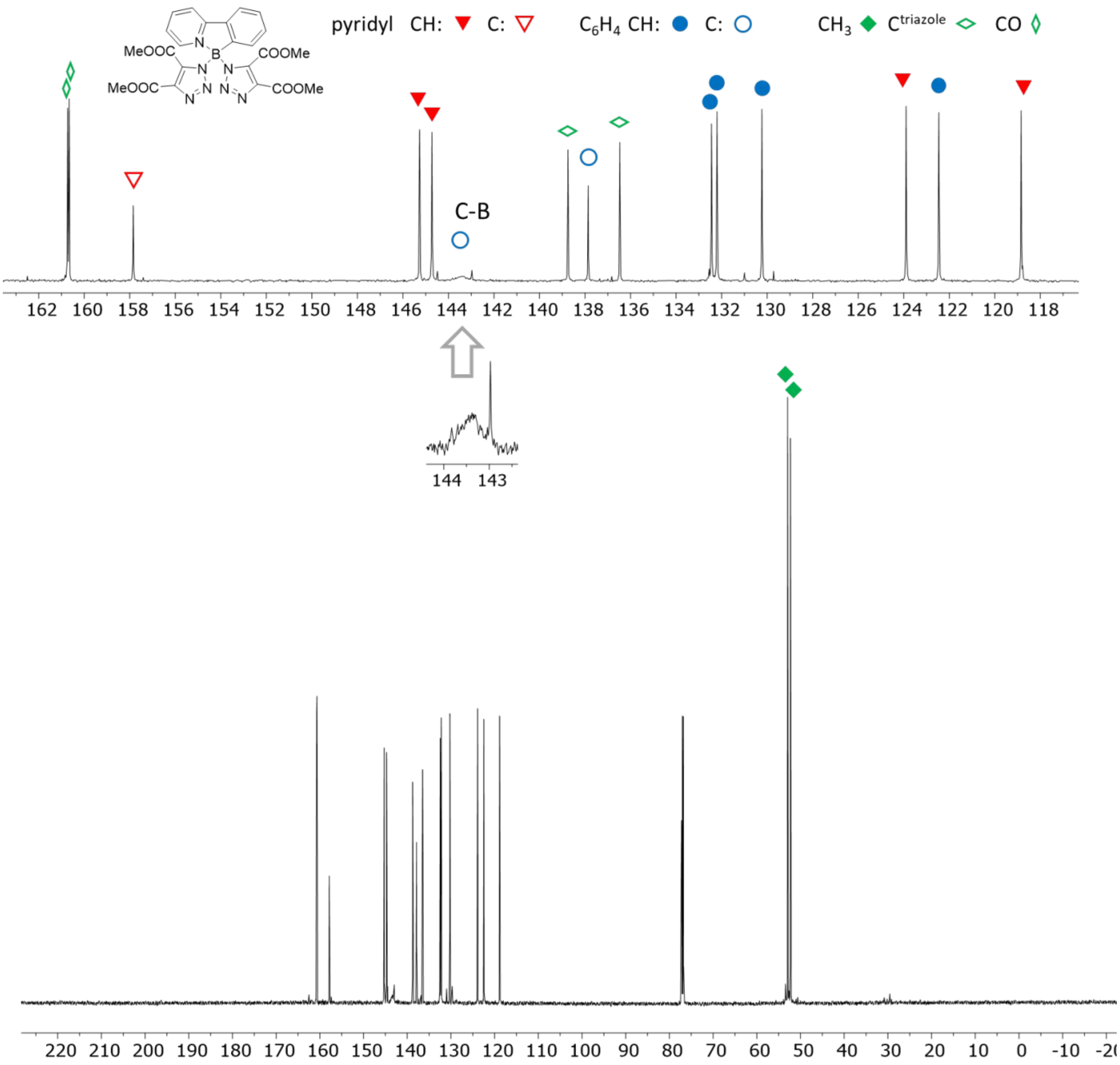

Figure S86: $^{13}C\{^{1}H\}$ **NMR** (151 MHz, $CDCl_3$, 293 K) spectrum of compound **15b**.

Crystal structure determination of **15b** [KSK481-3tw_twin1_hklf4]

A crystal suitable for SC-XRD structure determination was obtained by slow evaporation of a solution of **15b** in a dichloromethane:heptane mixture (ca. 1:1) under ambient conditions.

Crystal Data for $C_{23}H_{20}BN_7O_8$ (M =533.27 g/mol): monoclinic, space group P2$_1$/c (no. 14), a = 15.4095(5) Å, b = 6.5272(2) Å, c = 24.6241(6) Å, β = 90.226(3)°, V = 2476.69(13) Å^3, Z = 4, T = 100.00(10) K, μ(Cu Kα) = 0.931 mm^{-1}, $Dcalc$ = 1.430 g/cm^3, 4345 reflections measured (5.736° ≤ 2Θ ≤ 133.2°), 4345 unique (R_{int} = ?, R_{sigma} = 0.0215) which were used in all calculations. The final R_1 was 0.0677 (I > 2σ(I)) and wR_2 was 0.2236 (all data). **CCDC: 2445679**

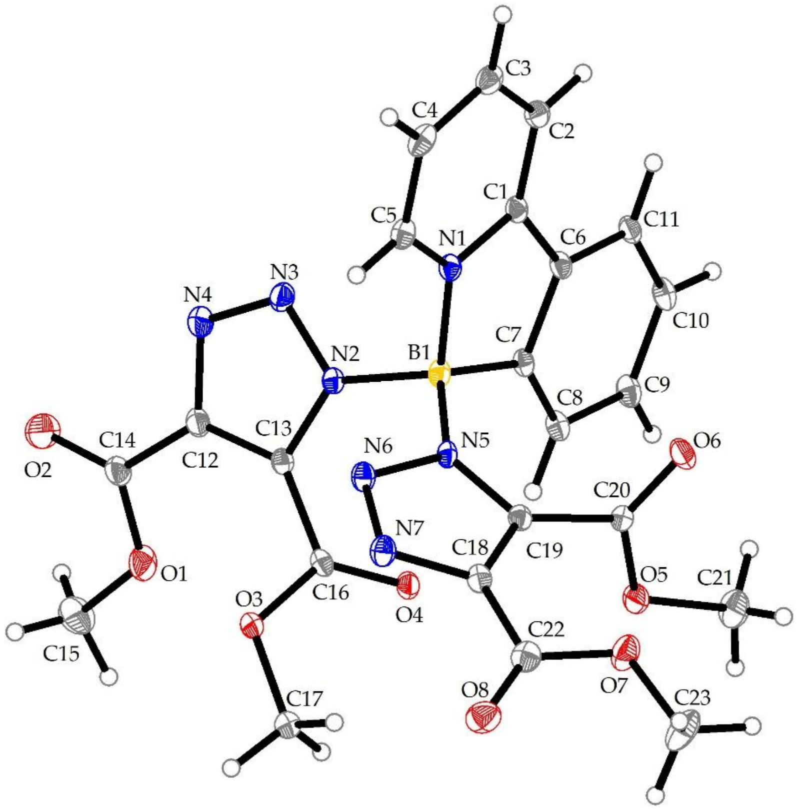

Figure S87: Crystal structure of **15b** (thermal ellipsoids shown at 30 % probability level).

Preparation of compound **15c**

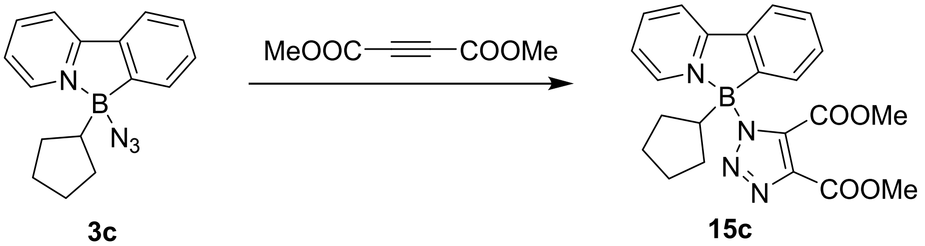

Boron azide **3c** (138 mg, 0.500 mmol) dissolved in 5 mL of chloroform was treated with dimethyl acetylenedicarboxylate (78 mg, 0.55 mmol) in 5 mL of chloroform and the resulting mixture was left stirring at 60 °C overnight. On the following day, the reaction mixture was directly loaded onto a chromatographic column packed with silica gel and dichloromethane. Impurities were eluted first using dichloromethane and, subsequently, the polarity of the mobile phase was increased to dichloromethane / methanol (10:1) mixture to elute the product. Compound **15c** was obtained as a white foamy solid (201 mg, 0.48 mmol, 96% yield, which exhibited purple-blue luminescence under irradiation with UV lamp.

Elemental analysis calculated for $C_{22}H_{23}N_4BO_4$ (418.2): C 63.18, H 5.54, N 13.40; found C 62.88, H 5.56, N 13.15.

HRMS calculated for $C_{22}H_2N_4BO_4^+$ $[M+H]^+$: 419.1885, found 419.1876.

IR (ATR) ν: 3039 (w), 2938 (m), 2864 (w), **1728 (vs, C=O)**, 1623 (m), 1544 (m), 1490 (s), 1448 (s), 1433 (m), 1419 (m), 1350 (m), 1334 (w), 1282 (m), 1253 (m), 1205 (s), 1177 (s), 1127 (m), 1077 (m), 1057 (m), 1005 (w), 970 (w), 951 (w), 887 (w), 849 (m), 821 (w), 797 (m), 771 (s), 752 (s), 694 (w), 677 (w), 637 (w), 555 (m), 469 (w) cm^{-1}.

^{1}H NMR (600 MHz, $CDCl_3$, 293 K): δ = [8.50 (dd, $^3J_{HH}$ = 5.9 Hz, $^4J_{HH}$ = 1.3 Hz), 8.10 (m), 8.00 (d, $^3J_{HH}$ = 7.8 Hz), 7.41 (m)](each 1H, pyridyl), [7.87 (dd, $^3J_{HH}$ = 7.6 Hz, $^4J_{HH}$ = 1.5 Hz), 7.61 (d, $^3J_{HH}$ = 7.2 Hz), 7.47 (m), 7.45 (m)](each 1H, C_6H_4), [3.83, 3.14](each s, 3H, COOMe), [2.21, 1.94, 1.52, 1.43, 1.43, 1.29, 1.20, 0.89, 0.21](each m, 1H, cyclopentyl).

^{13}C{^{1}H} NMR (151 MHz, $CDCl_3$, 293 K): δ = [162.2 (CO), 161.2 (CO), 137.2 (C triazole), 137.0 (C triazole), 52.3 (Me), 51.9 (Me)], [156.7 (*i*-C), 142.5 (CH), 142.2 (CH), 123.0 (CH), 118.1 (CH)](pyridyl), [150.6 (br, C-B), 137.9 (*i*-C), 131.4 (CH), 130.9 (CH), 127.9 (CH), 121.6 (CH)](C_6H_4), [32.5 (br), 128.9, 28.1, 26.9, 26.4](cyclopentyl).

^{11}B{^{1}H} NMR (193 MHz, $CDCl_3$, 293 K): δ = 4.0 ($\nu_{1/2} \approx$ 350 Hz).

^{11}B NMR (193 MHz, $CDCl_3$, 293 K): δ = 4.0 ($\nu_{1/2} \approx$ 360 Hz).

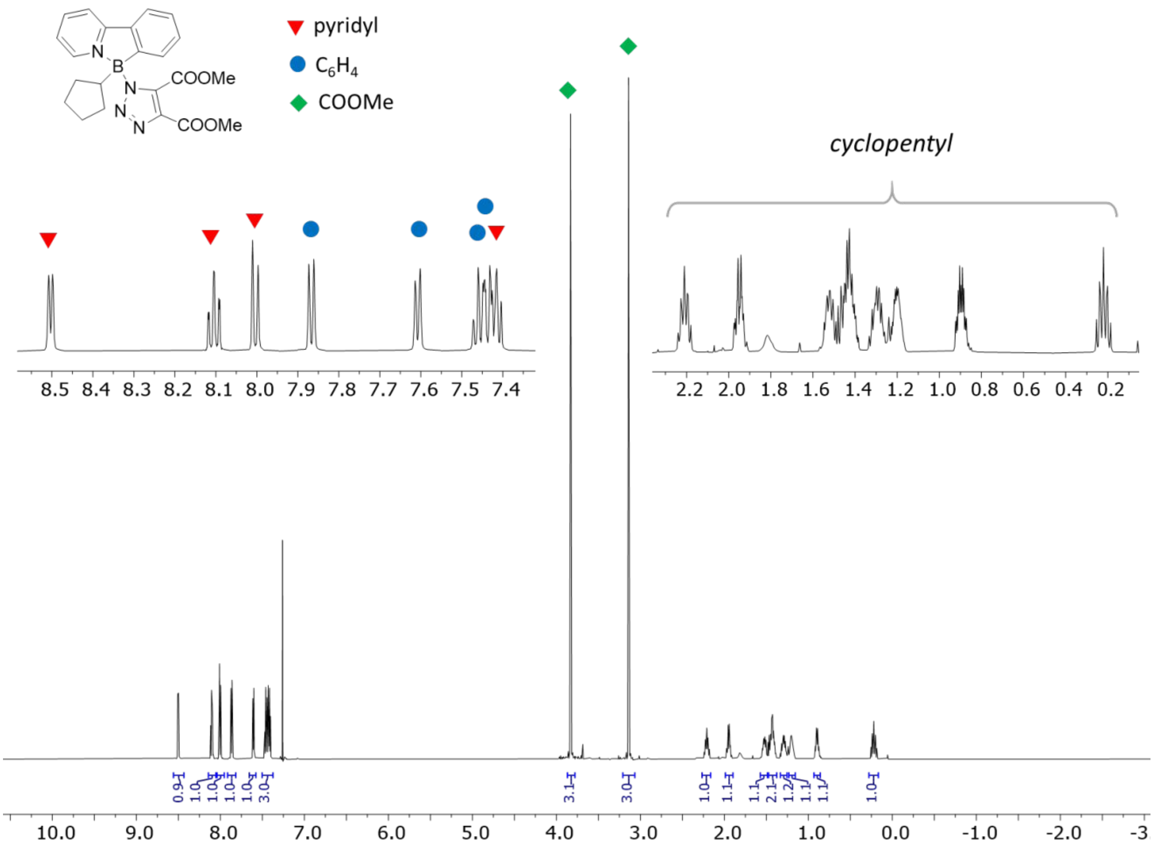

Figure S88: **^{1}H NMR** (600 MHz, $CDCl_3$, 293 K) spectrum of compound **15c**.

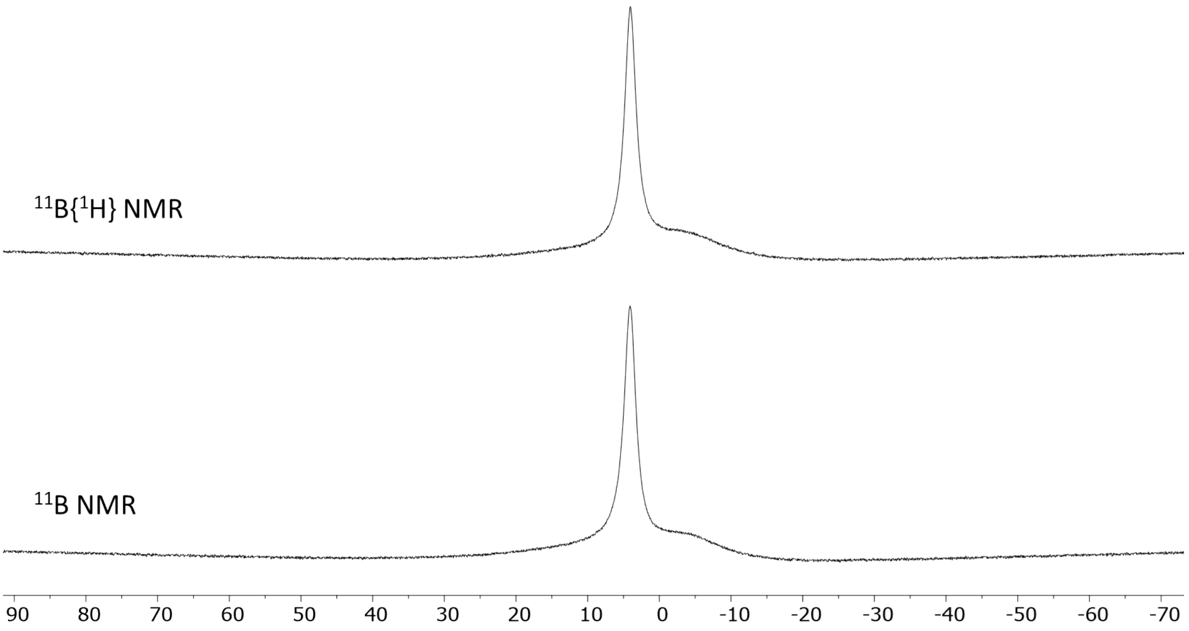

Figure S89: **^{11}B** and **$^{11}B\{^{1}H\}$ NMR** (193 MHz, $CDCl_3$, 293 K) spectra of compound **15c.**

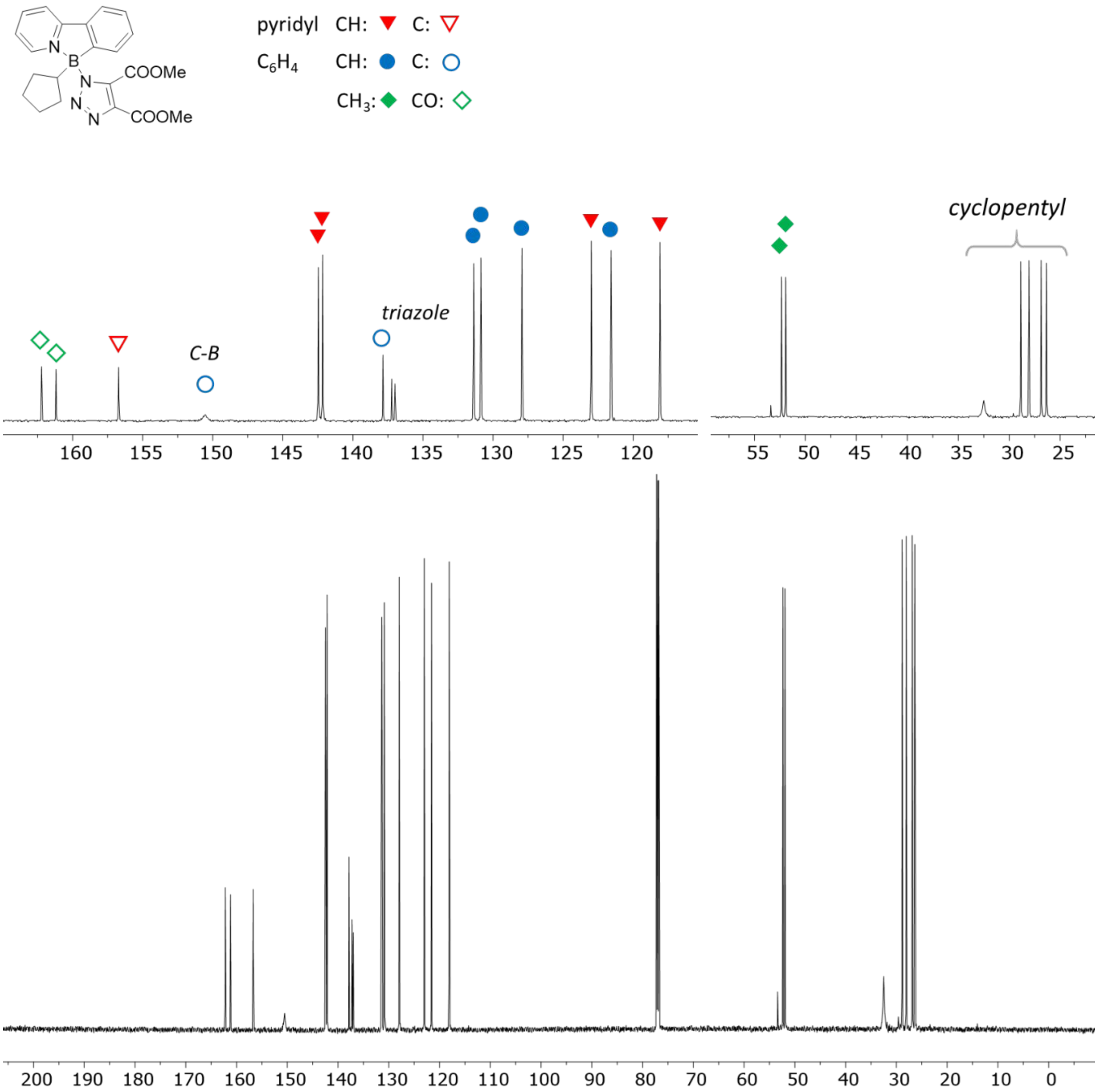

Figure S90: **$^{13}C\{^{1}H\}$ NMR** (151 MHz, $CDCl_3$, 293 K) spectrum of compound **15c**.

Preparation of compound **16**

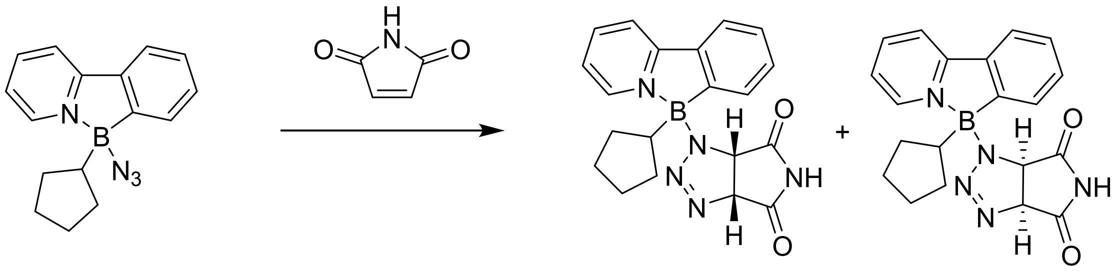

Boron azide **3c** (138 mg, 0.500 mmol) and maleimide (58.2 mg, 0.600 mmol) were suspended in dry toluene (5 mL) and heated to 60 °C for 18 h. On the following day, the volatiles were removed *in vacuo* to give a colourless oily residue, which was purified by column chromatography on silica gel using a mixture of AcOEt/Hx (1:1) as the eluent. Compound **16** was obtained as a white foamy semi-solid (172 mg, 0.461 mmol, 92% yield). According to ^{1}H NMR, the product consisted of two isomers in an approximate ratio of 2:1. The attempts to separate the isomers by repeated column chromatography or crystallization were not successful.

Satisfactory elementary analysis was not obtained.

HRMS calculated for $C_{22}H_{21}N_5BO_2^+$ [M+H]$^+$: 374.1792 found 374.1814.

IR (KBr) ν: 3059 (w), 2939 (m), 2858 (m), 2752 (w), 1781 (w), **1712 (vs, CO)**, 1620 (s), 1573 (w), 1486 (s), 1426 (m), 1332 (m), 1299 (w), 1274 (m), 1180 (s), 1114 (m), 1060 (m), 999 (w), 906 (m), 821 (w), 801 (w), 759 (m), 725 (s), 646 (w), 629 (w), 602 (m), 565 (m), 519 (w), 432 (w) cm^{-1}.

^{1}H NMR (600 MHz, CDCl$_3$, 293 K): major isomer: δ = 8.64 (br, 1H, NH), [8.33, 8.00, 7.93, 7.86, 7.56, 7.42, 7.39, 7.30] (each m, 1H, $C_6H_4C_5H_4N$), [5.11 (d, $^{3}J_{cisHH}$ = 10.9 Hz), 4.54 (d, $^{3}J_{cisHH}$ = 10.9 Hz)](both 1H, triazoline); minor isomer: δ = 9.08 (br, 1H, NH), [8.72, 7.99, 7.92, 7.82, 7.70, 7.42, 7.39, 7.34](each m, 1H, $C_6H_4C_5H_4N$), [5.13 (d, $^{3}J_{cisHH}$ = 11.0 Hz), 4.54 (d, $^{3}J_{cisHH}$ = 11.0 Hz)](both 1H, triazoline); *signals of cyclopentyl fragment for major and minor isomer were not unambiguously distingushed:* [2.07, 1.95-1.80 (m), 1.61-1.35 (m), 1.28-1.15 (m), 0.87 (m), 0.23 (m)].

^{13}C{^{1}H} NMR (151 MHz, CDCl$_3$, 293 K): major isomer: δ = [174.2, 172.7](CO), [156.8 (*i*-C), 152.5 (br, C-B), 141.7 (CH), 141.6 (CH), 138.1 (*i*-C), 130.8 (CH), 130.3 (CH), 127.5 (CH), 122.5 (CH), 121.8 (CH)]($C_6H_4C_5H_4N$), [79.7, 60.5](triazoline), [32.8 (br, C-B), 29.2 (CH_2), 28.6 (CH_2), 26.9 (CH_2), 26.3 (CH_2)](cyclopentyl); minor isomer: δ = [176.5, 172.7](CO), [156.8 (*i*-C), 153.7 (br, C-B), 143.6 (CH), 141.4 (CH), 136.8 (*i*-C), 131.5 (CH), 130.9 (CH), 127.1 (CH), 122.7 (CH), 121.3 (CH), 117.7 (CH)]($C_6H_4C_5H_4N$), [80.0, 60.6](triazoline), [30.8 (br, C-B), 28.6 (CH_2), 28.6 (CH_2), 27.2 (CH_2), 26.4 (CH_2)](cyclopentyl).

^{11}B{^{1}H} NMR (193 MHz, CDCl$_3$, 293 K): δ = 4.6 ($\nu_{1/2} \approx$ 600 Hz) *overlapping signal of both isomers.*

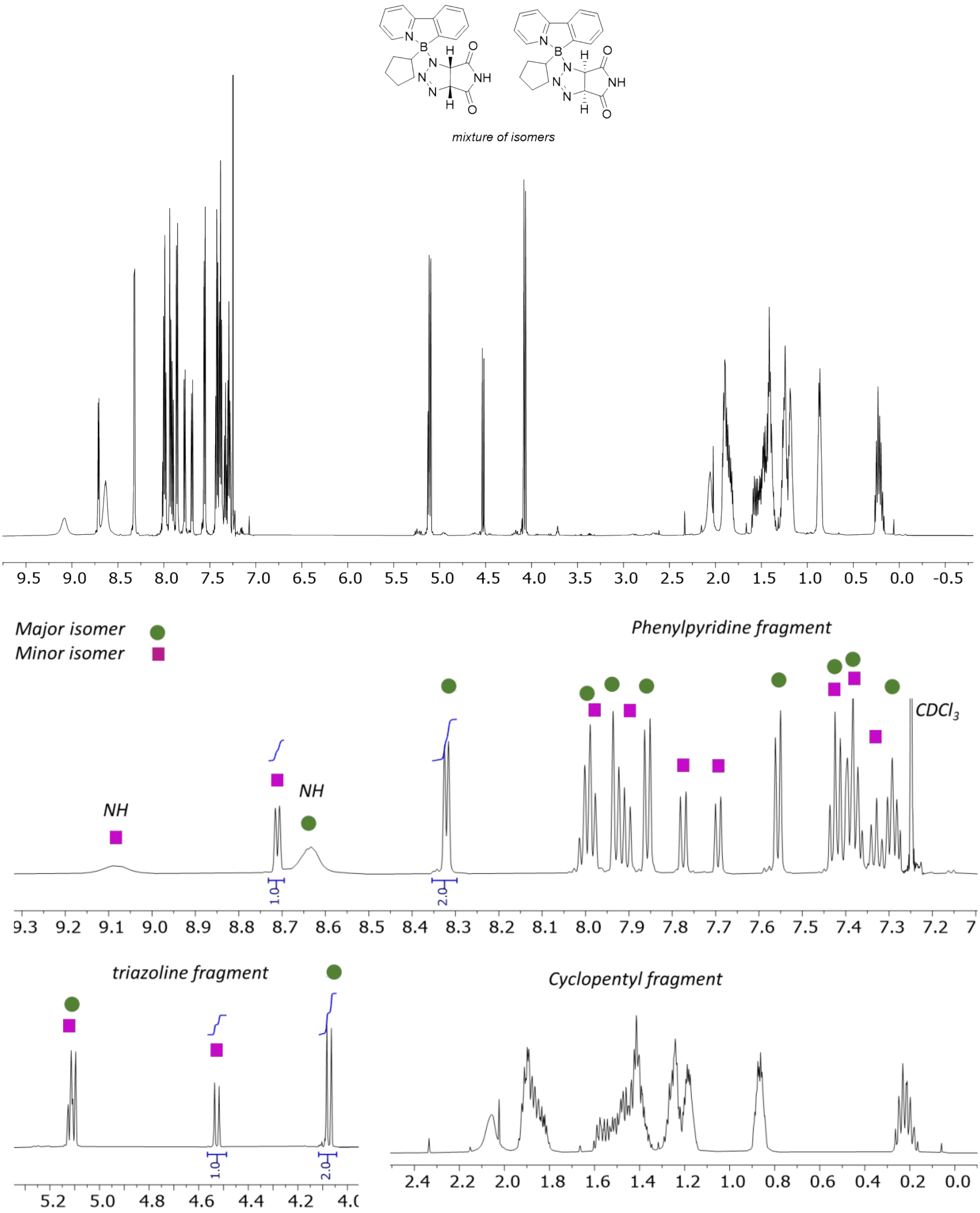

Figure S91: **^{1}H NMR** ($CDCl_3$, 600 MHz, 293 K) spectrum of compound **16**.

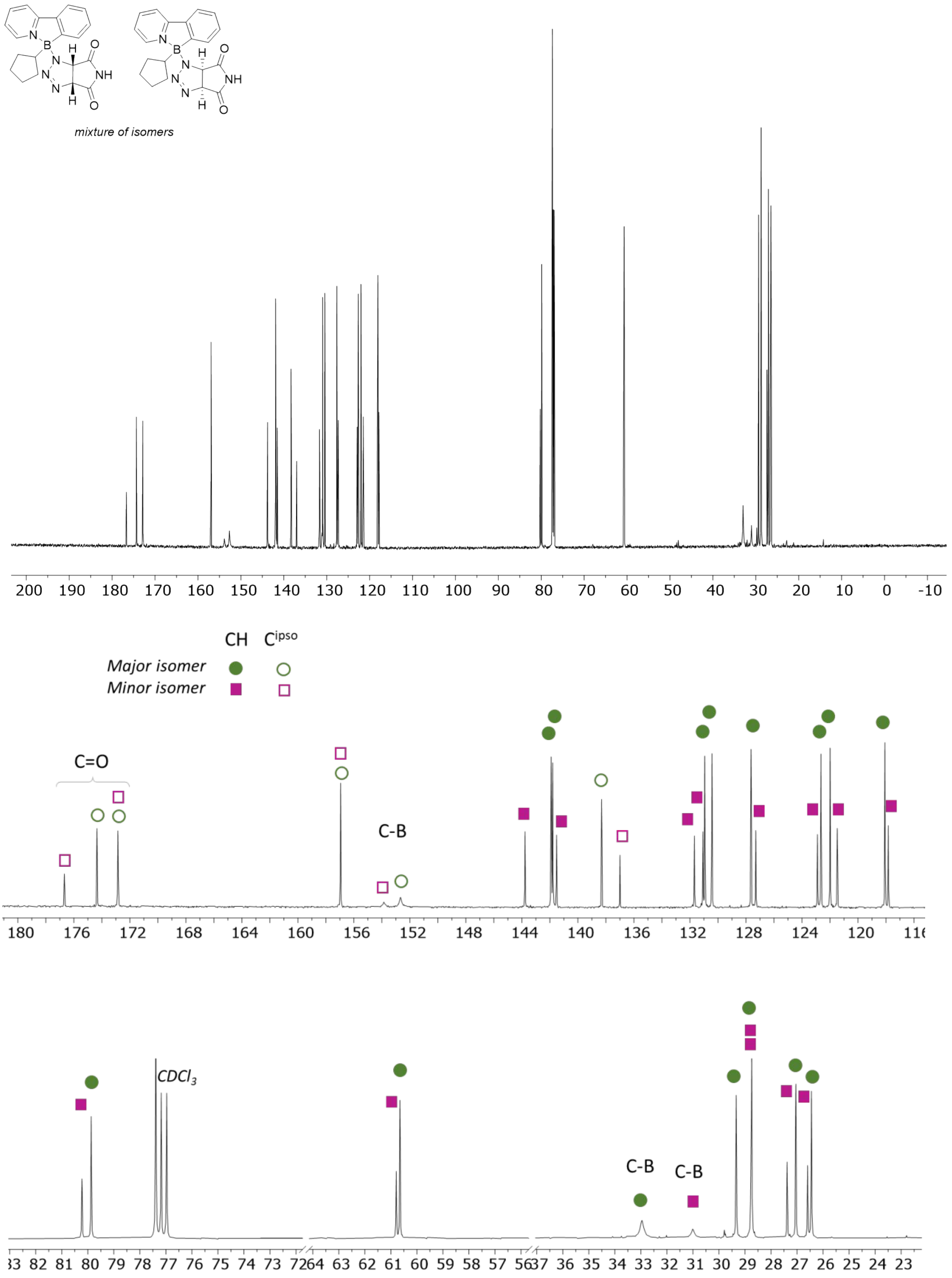

Figure S92: **^{13}C{^{1}H} NMR** (151 MHz, $CDCl_3$, 293 K) spectrum of compound **16**.

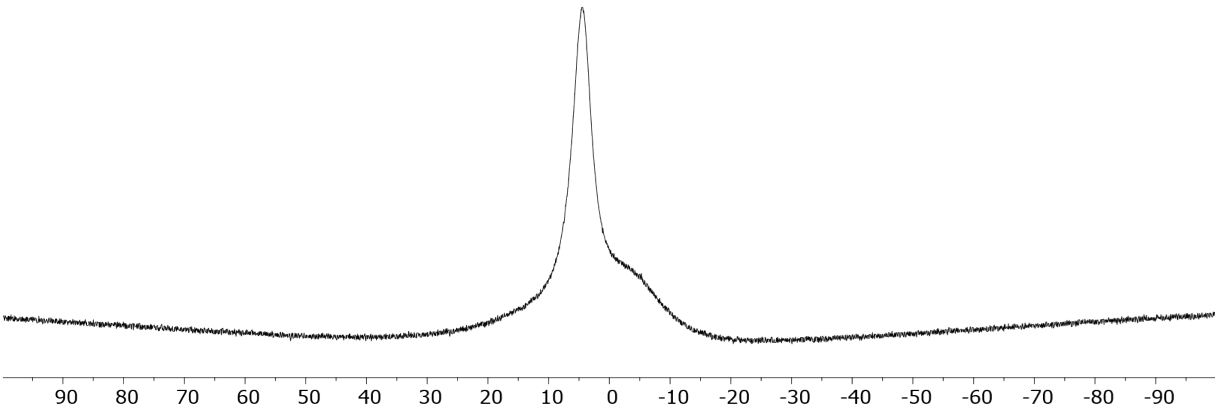

Figure S93: **^{11}B{^{1}H} NMR** (193 MHz, $CDCl_3$, 293 K) spectrum of compound **16**.

Kinetic investigation of triazole formation

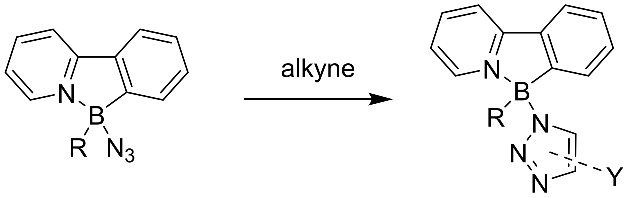

General procedure: Boron azide (0.5 mmol) and the corresponding alkyne (0.75 mmol, cyclooctyne or dimethyl acetylenedicarboxylate, DMAD) were combined in $CDCl_3$ (0.7 mL) and placed into an oil bath preheated to 60 °C. Reaction progress was periodically monitored by ^{1}H NMR.

Table S1: Kinetic investigation of reactivity of boron azides towards cyclooctyne and DMAD.

$c_{0(\text{azide})}$ = 0.071 M

**14a**	H/cyklooctyne		
t(min)	yield of **14a** (%)	**3a** conversion (%)	ln[azide]
0	0	100	-2,64
15	20	80	-2,86
30	33	67	-3,04
60	52	48	-3,37
90	63	37	-3,63
120	70	30	-3,84
150	75	25	-4,03
180	78	22	-4,15
210	82	18	-4,35

**14c**	pentyl/cyclooctyne		
t(min)	yield of **14c** (%)	**3c** conversion (%)	ln[azide]
0	0	100	-2,64
15	7	93	-2,71
30	13	87	-2,78
60	23	77	-2,90
90	32	68	-3,02
120	38	62	-3,12
150	44	56	-3,22
180	49	51	-3,31
210	52	48	-3,37

**15a**	H/DMAD		
t(min)	yield of **15a** (%)	**3a** conversion (%)	ln[azide]
0	0	100	-2,64
15	67	33	-3,75
30	84	16	-4,47
45	93	7	-5,30
60	96	4	-5,86
75	98	2	-6,55
90	99	1	-7,24

**15c**	pentyl/DMAD		
t(min)	yield of **15c** (%)	**3c** conversion(%)	ln[azide]
0	0	100	-2,64
15	38	62	-3,12
30	56	44	-3,46
45	68	32	-3,78
60	77	23	-4,11
75	84	16	-4,47
90	88	12	-4,76
120	93	7	-5,30
150	96	4	-5,86

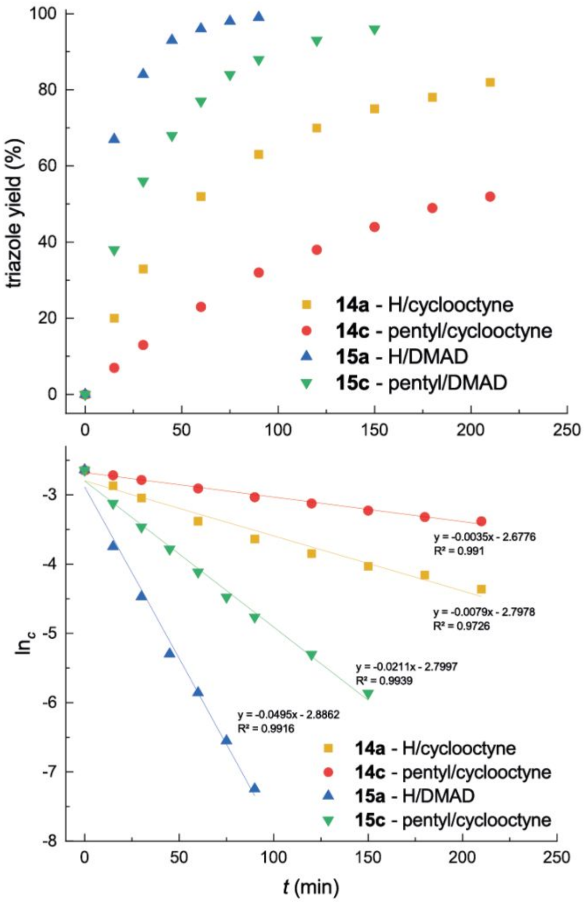

Computational details

Geometry optimizations and examination of the reaction pathways were carried out at the B3LYP/6-31+G* level. The same applies to zero-point, heat-capacity, and entropy corrections with frequencies. The excited states were computed at the CIS/6-311+G** level using the B3LYP/6-31+G* geometry, in which the presence of dichloromethane was considered in terms of applying the SMD approach. All these computations were performed using Gaussian16.[S11] The IAO/IBO method[S12] was used to connect quantitative SCF wave functions to a qualitative chemical picture, the nature of the orbitals naturally emerges. The IBOview program was used. The corresponding input files for the latter were generated at the B3LYP/def2-TZVP//B3LYP/6-31+G* level using the Turbomole6.6 program package.[S13]

Table S2: Cartesian coordinates of TS(A) derived at the B3LYP/6-31+G* level

C	-4.971902	0.456850	-1.315895
C	-3.933911	1.596907	-1.535675
C	-2.880326	-0.790895	0.546365
C	-2.503530	1.215386	-1.987662
C	-1.981181	-0.095756	0.053229
C	-1.478357	0.938250	-0.863564
H	-4.722400	-0.395328	-1.965931
H	-3.878376	2.221851	-0.632046
H	-2.556420	0.335451	-2.644105
H	-5.935349	0.833896	-1.684690
H	-4.343666	2.248972	-2.318691
H	-2.098388	2.033493	-2.600577
H	-0.519895	0.648219	-1.311879
H	-1.280195	1.864267	-0.304736
C	-5.224607	-0.050948	0.123628
H	-6.257190	-0.424341	0.184075
H	-5.163337	0.796565	0.820719
C	-4.300347	-1.179663	0.630012
H	-4.574658	-1.446668	1.658078
H	-4.463274	-2.087157	0.029148
N	-0.820225	-1.598505	1.925218
N	-1.903350	-2.024325	2.066581
N	-0.160157	-0.776627	1.264612

B	1.328594	-0.522979	1.398441
C	1.830333	0.964613	1.059720
N	2.156581	-1.283593	0.199520
C	2.790280	0.941616	0.023773
C	1.522539	2.199062	1.639126
C	2.967105	-0.422210	-0.475307
C	2.114842	-2.588529	-0.102881
C	3.439859	2.099383	-0.425903
C	3.800705	-0.904694	-1.492177
C	3.768091	-2.259323	-1.807856
C	2.909332	-3.119922	-1.110042
H	4.454572	-0.222505	-2.025215
H	4.405506	-2.648009	-2.597424
H	1.427348	-3.189661	0.483521
H	2.857468	-4.178446	-1.340543
C	3.113321	3.317145	0.168887
H	0.786219	2.256073	2.437857
C	2.155910	3.364733	1.194589
H	4.181151	2.062113	-1.221269
H	3.599616	4.230782	-0.162787
H	1.907005	4.321653	1.647834
H	1.792411	-0.957017	2.430728

Table S3: Cartesian coordinates of TS(B) derived at the B3LYP/6-31+G* level

C	-2.821734	-0.666839	-0.013907
C	-2.106740	-0.132678	-0.880764
C	-1.929767	0.651474	-2.097462
O	-2.076418	0.231389	-3.224795
O	-1.585366	1.933813	-1.810761
O	-4.031346	-1.075519	0.668687
O	-4.298418	-0.267024	1.721195
O	-4.720714	-2.023347	0.346675

N	-0.432968	-1.417892	0.529569
N	-1.311101	-1.797307	1.197952
N	-0.150318	-0.758861	-0.485019
B	1.185931	-0.080625	-0.798214
C	2.513812	-0.967117	-0.645906
N	1.542466	1.037722	0.331705
C	3.384225	-0.376693	0.296755
C	2.927705	-2.140471	-1.281171
C	2.777308	0.825486	0.868765
C	0.776269	2.073195	0.711285
C	4.637724	-0.922558	0.603610
C	3.275838	1.710797	1.831492
C	2.488990	2.785895	2.233271
C	1.218847	2.973291	1.671436
H	4.260665	1.549040	2.256592
H	2.860163	3.478294	2.983955
H	-0.188654	2.153901	0.220709
H	0.583334	3.799542	1.970714
H	5.027597	-2.094019	-0.044031
H	2.281360	-2.623750	-2.010251
C	4.174284	-2.699998	-0.978981
H	5.298476	-0.454549	1.329858
H	5.993456	-2.540023	0.177535
H	4.489028	-3.615604	-1.473996
H	-1.396785	2.788974	-2.956954
C	-5.475392	-0.609595	2.476790
H	-2.315358	2.840379	-3.546892
H	-1.147249	3.768389	-2.547247
H	-0.583399	2.410150	-3.581195
H	-5.380922	-1.616973	2.891089
H	-5.528497	0.132429	3.274149
H	-6.364903	-0.560703	1.842788

H	1.078602	0.496555	-1.852186

Table S4: Cartesian coordinates of **14a** derived at the B3LYP/6-31+G* level

C	-4.747149	1.091278	-0.937303
C	-3.382970	1.794558	-1.061350
C	-2.535338	-1.157295	0.443844
C	-2.295496	1.001238	-1.833344
C	-1.411313	-0.402486	0.137039
C	-1.096526	0.548139	-0.984727
H	-5.049345	0.734368	-1.934539
H	-3.014395	2.051848	-0.057974
H	-2.744239	0.135886	-2.335715
H	-5.496927	1.841636	-0.648492
H	-3.548225	2.751787	-1.573007
H	-1.891253	1.628971	-2.637847
H	-0.352595	0.087293	-1.653192
H	-0.606875	1.438976	-0.573276
C	-4.848224	-0.070559	0.064403
H	-5.883228	-0.438912	0.033161
H	-4.689000	0.305878	1.084297
C	-3.905288	-1.276519	-0.168051
H	-4.354182	-2.158926	0.301682
H	-3.846799	-1.498307	-1.242078
N	-1.040568	-1.635248	1.962273
N	-2.253304	-1.890410	1.566670
N	-0.496490	-0.718988	1.112017
B	0.979638	-0.362086	1.317406
C	1.545734	1.089808	0.935620
N	1.895683	-1.223908	0.289640
C	2.614323	0.963669	0.018271
C	1.238521	2.369450	1.409265
C	2.801764	-0.438207	-0.356062

C	1.851811	-2.552324	0.105484
C	3.356820	2.066098	-0.426603
C	3.728169	-1.023478	-1.227814
C	3.691814	-2.400335	-1.426579
C	2.739522	-3.181580	-0.756769
H	4.457466	-0.403997	-1.739043
H	4.402022	-2.868975	-2.102411
H	1.089843	-3.081006	0.670377
H	2.688897	-4.255864	-0.896349
C	3.020448	3.331169	0.052265
H	0.430874	2.506037	2.125311
C	1.965207	3.479593	0.966273
H	4.180150	1.949317	-1.127907
H	3.578482	4.202910	-0.278901
H	1.714653	4.471322	1.335685
H	1.308262	-0.703236	2.425824

Table S5: Cartesian coordinates of **15a** derived at the B3LYP/6-31+G* level

C	2.193624	0.889394	-0.836971
C	1.515773	0.119301	0.105883
C	1.976380	-0.883570	1.100994
O	1.550190	-2.019914	1.168995
O	2.908081	-0.374340	1.923841
C	3.629457	0.964601	-1.177906
O	4.294478	-0.137525	-0.748088
O	4.159462	1.876194	-1.777002
N	0.085944	1.331446	-1.065094
N	1.269857	1.614145	-1.527416
N	0.205525	0.420060	-0.065594
B	-1.047261	-0.112604	0.691594
C	-1.865730	-1.286898	-0.029590
N	-2.177655	1.023132	0.646878

C	-3.174446	-0.840444	-0.315167
C	-1.524684	-2.599340	-0.365557
C	-3.335202	0.550869	0.101799
C	-2.074574	2.285693	1.090089
C	-4.133447	-1.665046	-0.917629
C	-4.452070	1.390204	0.015115
C	-4.353351	2.698983	0.474101
C	-3.147382	3.160959	1.019508
H	-5.375247	1.015623	-0.413703
H	-5.210305	3.363593	0.406862
H	-1.109776	2.572879	1.493306
H	-3.039391	4.178823	1.377177
C	-3.768834	-2.969466	-1.248664
H	-0.526316	-2.970825	-0.150472
C	-2.471856	-3.430632	-0.974866
H	-5.138738	-1.307806	-1.129295
H	-4.491184	-3.630360	-1.720419
H	-2.202084	-4.450374	-1.239557
C	3.465545	-1.293054	2.884081
C	5.707786	-0.150273	-1.013635
H	3.948039	-2.128225	2.369448
H	4.195212	-0.710551	3.446703
H	2.681787	-1.675418	3.543088
H	6.196495	0.694245	-0.519701
H	6.069158	-1.096516	-0.609345
H	5.893584	-0.094863	-2.089603
H	-0.760245	-0.321691	1.844630

Supporting Information – References

[S1] Škoch, K.; Buziková, M.; Hnyk, D.; Litecká, M.; Kloda, M.; Kirakci, K.; Lang, K. Preparation, Structure, Reactivity, Lewis Acidic and Fluorescence Properties of Arylpyridine Based Boron C,N-Chelates Featuring Weakly Coordinating Anions. *Chemistry – A European Journal*, **2024**, *30*, e202403263.
https://doi.org/10.1002/chem.202403263

[S2] Parks, D.J.; Piers, W.E.; Yap G.P.A. Synthesis, Properties, and Hydroboration Activity of the Highly Electrophilic Borane Bis(pentafluorophenyl)borane, $HB(C_6F_5)_2$. *Organometallics*, **1998**, *27*, 5492-5503. https://doi.org/10.1021/om980673e

[S3] Brandsma, L.; Verkruijsse, H.D An Improved Synthesis of Cyclooctyne. *Synthesis* **1987**, 290.

DOI: 10.1055/s-1978-24725

[S4] Rigaku OD. (2023). CrysAlisPro. Retrieved from https://rigaku.com/products/crystallography/x-ray-diffraction/crysalispro

[S5] Coppens, P.; Leiserowitz, L.; Rabinovich, D. Absorption corrections in X-ray crystallography. *Acta Crystallographica*, **1965**, *18*, 1035–1038. https://doi.org/10.1107/S0365110X65003047

[S6] North, A. C. T.; Phillips, D. C.; Mathews, F. S. A semi-empirical method of absorption correction. *Acta Crystallographica Section A* **1968**, *24*, 351–359. https://doi.org/10.1107/S0567739468000707

[S7] Sheldrick, G. M. SHELXT – Integrated space-group and crystal-structure determination. *Acta Crystallographica Section A: Foundations and Advances*, **2015**, 71, 3–8. https://doi.org/10.1107/S2053273314026370

[S8] Sheldrick, G. M. Crystal structure refinement with SHELXL. *Acta Crystallographica Section C: Structural Chemistry*, **2015**, 71, 3–8. https://doi.org/10.1107/S2053229614024218

[S9] Dolomanov, O. V.; Bourhis, L. J.; Gildea, R. J.; Howard, J.A.K.; Puschmann, H. OLEX2: a complete structure solution, refinement and analysis program. *Journal of Applied Crystallography*, **2009**, 42, 339–341. https://doi.org/10.1107/S0021889808042726

[S10] Diamond, v4.6.3. (2020). Diamond—Crystal and Molecular Structure Visualization. Crystal Impact. http://www.crystalimpact.com/diamond

[S11] M. J. Frisch, G. W. Trucks, H. B. Schlegel, G. E. Scuseria, M. A. Robb, J. R. Cheeseman, G. Scalmani, V. Barone, G. A. Petersson, H. Nakatsuji, X. Li, M. Caricato, A. V. Marenich, J. Bloino, B. G. Janesko, R. Gomperts, B. Mennucci, H. P. Hratchian, J. V. Ortiz, A. F. Izmaylov, J. L. Sonnenberg, D. Williams-Young, F. Ding, F. Lipparini, F. Egidi, J. Goings, B. Peng, A. Petrone, T. Henderson, D. Ranasinghe, V. G. Zakrzewski, J. Gao, N. Rega, G. Zheng, W. Liang, M. Hada, M. Ehara, K. Toyota, R. Fukuda, J. Hasegawa, M. Ishida, T. Nakajima, Y. Honda, O. Kitao, H. Nakai, T. Vreven, K. Throssell, J. A. Montgomery, Jr., J. E. Peralta, F. Ogliaro, M. J. Bearpark, J. J. Heyd, E. N. Brothers, K. N. Kudin, V. N. Staroverov, T. A. Keith, R. Kobayashi, J. Normand, K. Raghavachari, A. P. Rendell, J. C. Burant, S. S. Iyengar, J. Tomasi, M. Cossi, J. M. Millam, M. Klene, C. Adamo, R. Cammi, J. W. Ochterski, R. L. Martin, K. Morokuma, O. Farkas, J. B. Foresman, and D. J. Fox, Gaussian 16, Revision C.01, Gaussian, Inc., Wallingford CT, USA, 2016.

[S12] G. Knizia, Intrinsic Atomic Orbitals: An Unbiased Bridge between Quantum Theory and Chemical ConceptsClick to copy article link. *J. Chem. Theory Comput.,* **2013**, *9*, 4834

[S13] *TURBOMOLE*, Version 7.3, 2018, a development of University of Karlsruhe and Forschungszentrum Karlsruhe GmbH, 1989-2017, TURBOMOLE GmbH, 2007.
